# In planta high levels of hydrolysable tannins inhibit peroxidase mediated anthocyanin degradation and maintain abaxially red leaves of *Excoecaria Cochinchinensis*

**DOI:** 10.1186/s12870-019-1903-y

**Published:** 2019-07-15

**Authors:** Honghui Luo, Wenjun Li, Xin Zhang, Shuangfan Deng, Qiuchan Xu, Ting Hou, Xuequn Pang, Zhaoqi Zhang, Xuelian Zhang

**Affiliations:** 1 State Key Laboratory for Conservation and Utilization of Subtropical Agro-bioresources/ Guangdong Provincial Key Laboratory of Postharvest Science of Fruits and Vegetables/ College of Horticulture, South China Agricultural University, Guangzhou, 510642 China; 2 College of Life Sciences, South China Agricultural University, Guangzhou, 510642 China

**Keywords:** Galloylglucoses/Ellagitannins, Anthocyanin maintenance, Peroxidase, Abaxially red leaves, *Excoecaria cochinchinensis*

## Abstract

**Background:**

Abaxially anthocyanic leaves of deeply-shaded understorey plants play important ecological significance for the environmental adaption. In contrast to the transient pigmentation in other plants, anthocyanins are permanently presented in these abaxially red leaves, however, the mechanism for the pigment maintenance remains unclear. In the present study, we investigated phenolic metabolites that may affect pigment stability and degradation in *Excoecaria cochinchinensis* (a bush of permanently abaxial-red leaves), via a comparison with *Osmanthus fragrans* (a bush of transiently red leaves).

**Results:**

High levels of galloylated anthocyanins were identified in the *Excoecaria* but not in the *Osmanthus* plants. The galloylated anthocyanin showed slightly higher stability than two non-galloylated anthocyanins, while all the 3 pigments were rapidly degraded by peroxidase (POD) in vitro. High levels of hydrolysable tannins [mainly galloylglucoses/ellagitannins (GGs/ETs)] were identified in *Excoecaria* but none in *Osmanthus*. GGs/ETs showed inhibition effect on POD, with IC50 ranged from 35.55 to 83.27 μM, correlated to the markedly lower POD activities detected in *Excoecaria* than in *Osmanthus*. Strong copigmentation was observed for GGs/ETs and anthocyanins, with more than 30% increase in the red intensity of non-galloylated anthocyanin solutions. In the leaf tissue, the hydrolysable tannins were observed to be co-localized with anthocyanins at the abaxial layer of the *Excoecaria* leaves, correlated to the low POD activity, more acidity and increased red intensity of the tissue.

**Conclusion:**

The results suggest that the *Excoecaria* leaves accumulate a distinct group of phenolic metabolites, mainly GGs/ETs, at the abaxial layer, which prevent anthocyanin degradation and increase the pigment stability, and consequently lead to the permanent maintenance of the red leaves.

**Electronic supplementary material:**

The online version of this article (10.1186/s12870-019-1903-y) contains supplementary material, which is available to authorized users.

## Background

Anthocyanins are the major pigments in plant kingdom, acting as protectors of photosynthetic tissues against damaging light, as signals for flowers and fruits to attract pollinators and seed dispersers [1–4]. In general, the pigments are transiently accumulated and degraded after their roles have been accomplished [5, 6], however, permanent presence of the pigments in the abaxially red leaves are found for many understorey plants. In contrast to intensive studies of ecological significance of the abaxial anthocyanins in the deeply-shaded plants, the mechanism for the pigment maintenance has rarely been dissected.

Anthocyanins accumulation usually occurs in the juvenile leaves of many plants, where the photosynthetic apparatus is not fully functional, and light in excess of photosynthetic capacity can lead to cellular damage [7, 8]. Predominantly located in the upper and/or lower epidermis of leaves, anthocyanins act as an efficient sunscreen to protect photosynthetic mesophyll cells [9]. Accordingly, under nature light, less photoinhibition of leaves, as represented by lower non-photochemical quenching (NPQ) and total xanthophyll to chlorophyll (VAZ/Chl) values, was found for many anthocyanic (red) species than anthocyanin-less ones [10–14]. In addition, anthocyanin biosynthesis in plant leaves is upregulated in response to various stresses, such as high UV radiation, low temperature [15], which is part of a mechanism to mitigate the effects of stress and enhance plant tolerance [16]. As the leaves mature and develop protective waxes, or are transferred to shade or the environmental stresses disappear, anthocyanins decrease rapidly, and the leaves turn completely green [1, 6, 17]. The decrease of anthocyanins presumably corresponds to a decreased need for photoprotection, to allow leaves to utilize higher light intensities [1, 18, 19].

Alternatively, anthocyanin presence in leaves may be permanent [20], and anthocyanins in these leaves are most commonly abaxial [20, 21]. The plants of permanently pigmented leaves are usually originated from the forest understoreys, have different color patterns on the surface, known as leaf variegation and can be popular as ornamental plants, for example, *Cyclamen purpurascens* [22], *Begonia fimbristipula* [23] and *Excoecaria cochinchinensis* Lour. Abaxial anthocyanins in these plants were proposed to weaken the green light to protect photosynthetic mesophyll cells during intermittent exposure to high-intensity sunlight (i.e. sun-patches) [23–25]. This photoprotective function of the abaxial anthocyanins is supported by the higher chlorophyll content and lower Chl a/b ratios in the anthocyanic (red) versus acyanic (non-red) leaves of *Begonia heracleifolia* (Cham. and Schltdl.) [24]. In contrast to the intensive studies of the ecological relevance of abaxially red leaves, the mechanism that anthocyanins are maintained and avoided from shade-promoted anthocyanin degradation, has been interested by researchers for many years, but has not yet been clarified [26, 27].

The concentration of anthocyanins in plant tissues is determined by the biosynthesis, degradation and stability of pigments in plants. In contrast to the deep knowledge on their biosynthesis [28, 29], anthocyanins degradation and stability in plant tissues are still not well known [6, 30, 31]. Enzymatic degradation has been considered to be responsible for anthocyanin breakdown in planta [6, 30], leading to pigment concentration reduction and red color fading. Polyphenol oxidases (PPOs) were presumed to be of anthocyanin degradation activities based on their abilities to degrade the pigments in fruit or fruit juice [32–34]. In plants, PPOs are located in cytosol and plastids [32], implying that PPOs might not be the enzymes that degrade the vacuole-located anthocyanins in vivo*.* In our previous study, an anthocyanin degradation enzyme (ADE) was purified from Litchi pericarp and identified as a laccase (ADE/LAC). The enzyme was demonstrated to be located in vacuoles and degraded anthocyanins coupled with epicatechin oxidation [35]. Recently, a basic peroxidase, BcPrx01, was found to be responsible for the in vivo degradation of anthocyanins in *Brunfelsia calycina*. BcPrx01 has the ability to degrade complex anthocyanins. It co-localizes with these pigments in the vacuoles of petals, and both mRNA and protein levels of BcPrx01 are greatly induced parallel to the degradation of anthocyanins [36]. Some environmental factors, such as high temperature and low density light, have been reported to enhance the degradation of anthocyanins by activating anthocyanin degradation peroxidase activity [15, 31].

Anthocyanin maintenance in leaves may also be influenced by anthocyanin stabilities, which were affected by their molecular structures [37, 38], the intravacuolar pH conditions, and the co-pigmentation [6, 39]. At pH < 3.0, not only the red color of anthocyanins was intensified, but also the stability of the pigments increased [40]. On the contrary, increased pH in the vacuole of senescing tissue may decrease the stability of the anthocyanins and cause degradation [41, 42]. It was found that the interaction between anthocyanin and coexisting colorless flavonoid components in the vacuoles prevented hydration of the anthocyanidin nucleus and brought about the color stabilization [28, 43]. This interaction, also called co-pigmentation, was able to increase the pigment color intensity and stability [44, 45].

In this paper, to investigate the mechanism of abaxial anthocyanin maintenance, we chose *Excoecaria cochinchinensis* as material. *Excoecaria* distributes throughout tropical Asia [46], growing wild and also being cultivated as a medicinal and garden bush, and characterized by the features that its leaves are nearly opposite, deep green above, and purple red beneath [47]. We chose another garden bush, *Osmanthus fragrans* var. semperflorens, with rapid anthocyanin degradation during leaf maturation [48], as a reference plant to investigate the mechanism of anthocyanin maintenance in *Excoecaria*. Anthocyanin components, activities of anthocyanin degradation-related enzymes (ADE), and the factors affecting the stability of the pigments in the two plants were analyzed in the present study. High levels of hydrolysable tannins [mainly galloylglucoses/ellagitannins (GGs/ETs)] were found to be co-localized with the anthocyanins in the leaves of *Excoecaria*. The substances inhibited peroxidases-mediated anthocyanin degradation and increased the pigments color density, which may be one of major factors for permanent maintenance of abaxially red leaves.

## Result

### *Excoecaria* leaves maintain abaxially red during leaf maturation

**Fig. 1:**
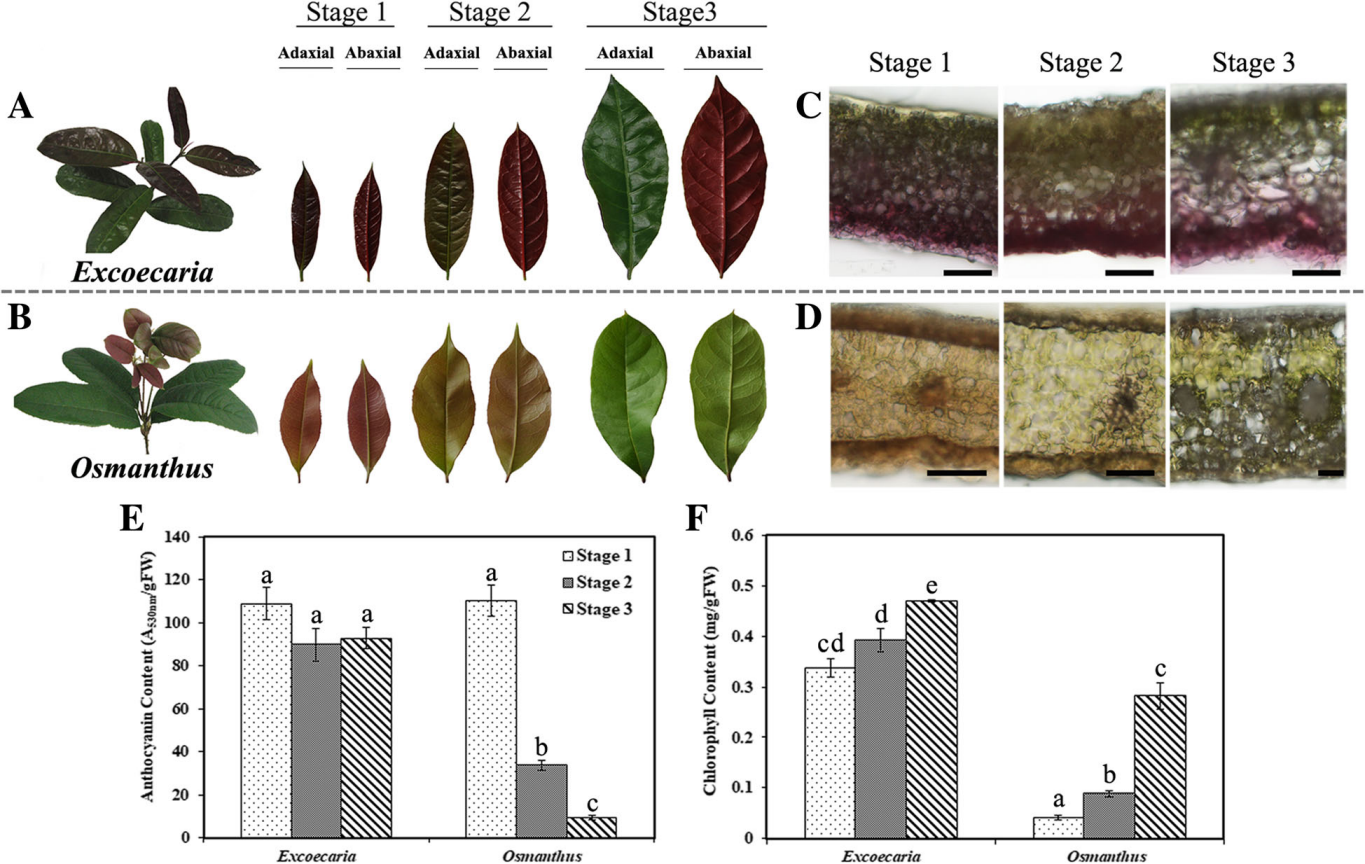
Change in color and anthocyanin levels during leaf maturation of *Excoecaria cochinchinensis* and *Osmanthus fragrans*. **A-B** The images of the leaves of *Excoecaria* (A) and *Osmanthus* (B). Stage 1 to 3 indicates red young, greening, and mature leaves respectively for the two species. **C-D** Cross-sections of the leaf blades of *Excoecaria* (C) and *Osmanthus* (D) were observed for anthocyanins location. Scale bars indicate 50 μm. **E** Change in anthocyanin content during leaf maturation. **F** Change in chlorophyll content during leaf maturation. The values are means of the measurements of three individual extractions. Error bars indicate the standard error of mean (SEM) of the values. Different letters denote significant differences in the values according to unpaired t test (*p* < 0.05) while shared letters denote non-significant differences in the values

The young leaves of *Excoecaria* and *Osmanthus* unfurled in red at both adaxial and abaxial surfaces. Completely greening occurred at the adaxial surface of the *Excoecaria* leaves, while the abaxial surface of the leaves maintained red in the process of leaf maturation (Fig. 1A). *Osmanthus* leaves gradually lost the red color and completely turned green at both sides (Fig. 1B). In the present study, stage 1, 2 and 3 were used to indicate the leaves at the stages of red, greening and completely adaxially green for both species (Fig. 1A and B). Microscopic observation of the cross sections of the leaves showed that large amounts of anthocyanins mainly located at the abaxial surface of *Excoecaria* leaves at the 3 stages (Fig. 1C). In contrast, anthocyanins located at both the adaxial/abaxial surface of leaves at stage 1 and 2 of *Osmanthus*, no anthocyanins were observed at stage 3 (Fig. 1D). To investigate whether color pattern of the leaves of the two species is related to the change in anthocyanins, the pigment contents during leaf maturation was determined. Around 110 A_530nm_/g FW anthocyanins were detected in both plants at stage 1. In contrast to around 90% reduction in anthocyanin content recorded in *Osmanthus* leaves from stage 1 to 3, no reduction in anthocyanin content was detected in *Excoecaria* leaves (Fig. 1E). With the leaf greening, chlorophyll contents increased from 0.34 to 0.47 and 0.05 to 0.3 mg/g FW respectively in *Excoecaria* and *Osmanthus* (Fig. 1F). These results indicated that, during leaf maturation, anthocyanins were maintained in *Excoecaria*, not like the large anthocyanin degradation in *Osmanthus* leaves.

### High levels of galloylated anthocyanins were identified in *Excoecaria* but not in *Osmanthus* leaves

**Fig. 2:**
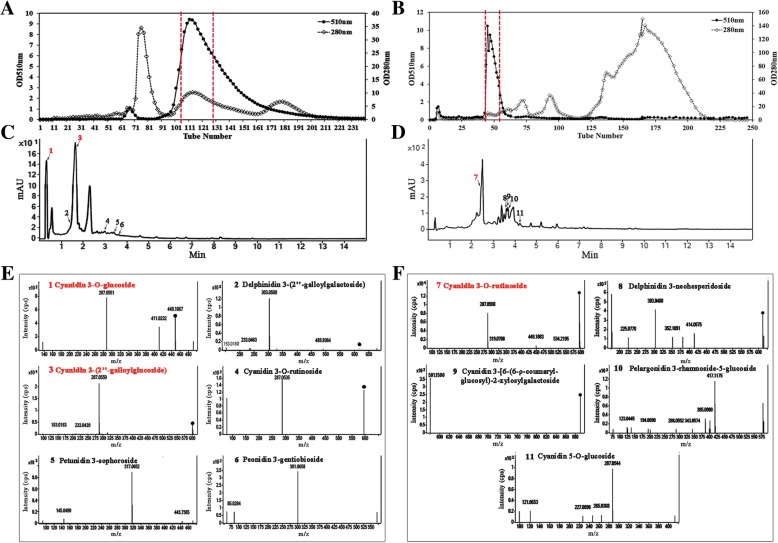
Identification of the main anthocyanins in the leaves of *Excoecaria* and *Osmanthus*, using UPLC-DAD-QTOF-MS/MS. **A-B** Anthocyanin purification profiles by column chromatography. Absorbance at 510 nm and 280 nm was determined in the fractions of Sephadex LH-20 column chromatography for the anthocyanin purification of *Excoecaria* (A) and *Osmanthus* (B) respectively. The major anthocyanin fractions as the indicated peaks between two dotted lines were collected for UPLC-DAD-QTOF-MS/MS analysis. **C-D** Anthocyanin UPLC-DAD profiles represented by absorbance at 280 nm. The anthocyanin fraction from *Excoecaria* (C) and *Osmanthus* (D) leaves as indicated in (A) and (B) were respectively analyzed by UPLC-DAD-QTOF-MS/MS. **E-F** MS/MS spectra of the main anthocyanins detected in *Excoecaria* (E) and *Osmanthus* (F) respectively. The prediction of the anthocyanins in (E-F) were based on the comprehensive MS/MS metabolite database “METLIN” (https://metlin.scripps.edu/)

To identify the anthocyanin molecules in *Excoecaria* and *Osmanthus* leaves, the anthocyanins in *Excoecaria* and *Osmanthus* leaves at stage 1 were purified by a Sephadex LH-20 column chromatography respectively. In the elution profiles, one major A510 nm peak was observed for each species, and the red fractions including No.104–127 tubes for *Excoecaria* sample and No.44–54 tubes for *Osmanthus* sample were analyzed by UPLC-DAD-QTOF-MS/MS respectively (Fig. 2A and B). UPLC-DAD analysis revealed six and five distinct anthocyanins in *Excoecaria* and *Osmanthus* respectively (Fig. 2C and D). According to comparison of the m/z and MS/MS fragment profiles of each anthocyanin positive ion with the information in “METLIN” database, six anthocyanins (No. 1–6) in *Excoecaria* were identified (Table 1), including cyanidin 3-O-glucoside, delphinidin 3-(2″-galloylgalactoside), cyanidin 3-(2″-galloylglucoside), cyanidin 3-O-rutinoside, petunidin 3-sophoroside and peonidin 3-gentiobioside (Table 1, Fig. 2E). Five identified anthocyanins (No.7–11) in *Osmanthus* were cyanidin 3-O-rutinoside, delphinidin 3-neohesperidoside, cyanidin 3-[6-(6-ρ-coumarylglucosyl)-2-xylosylgalactoside, pelargonidin 3-rhamnoside-5-glucoside and cyanidin 5-O-glucoside (Table 1, Fig. 2F). Based on the UPLC-DAD profiles represented by absorbance at 280 nm, cyanidin 3-(2″-galloylglucoside) (No. 3) and cyanidin 3-O-glucoside (No. 1) were regarded as the major anthocyanins in *Excoecaria* (Fig. 2C), while cyanidin 3-O-rutinoside (No. 7) was the one in *Osmanthus* (Fig. 2D). In *Excoecaria*, the most abundant cyanidin 3-(2″-galloylglucoside) and a minor delphinidin 3-(2″-galloylglucoside) were galloylated anthocyanins, while only non-galloylated anthocyanins were found in *Osmanthus*.

**Table 1:** The main anthocyanins detected in the leaves using UPLC-DAD-QTOF-MS/MS

Species	Compound No.^a^	Rt (min)	m/z		Molecular Formula^b^	Putative Compounds^b^
			[M+H]^+^	Fragment Ions		
*Excoecaria*	1	0.364	449.1080	176.9858, 190.9661, 287.0551	C_21_ H_21_ O_11_^+^	Cyanidin 3-O-glucoside
	2	1.392	617.1148	153.0183, 303.0500	C_28_ H_25_ O_16_^+^	Delphinidin 3-(2″-galloylgalactoside)
	3	1.724	601.1224	153.0181, 287.0552	C_28_ H_25_ O_15_^+^	Cyanidin 3-(2″-galloylglucoside)
	4	3.034	595.1666	287.0535	C_27_ H_31_ O_15_^+^	Cyanidin 3-O-rutinoside
	5	3.466	641.1705	145.0499, 317.0652, 443.7365, 479.1180	C_28_ H_33_ O_17_^+^	Petunidin 3-sophoroside
	6	3.698	625.1761	301.0658, 464.1260	C_28_ H_33_ O_16_^+^	Peonidin 3-gentiobioside
*Osmanthus*	7	2.399	595.1691	287.0550, 449.1083	C_27_ H_31_ O_15_^+^	Cyanidin 3-O-rutinoside
	8	3.577	611.1626	303.0488	C_27_ H_31_ O_16_^+^	Delphinidin 3-neohesperidoside
	9	3.626	889.2407	287.0550, 581.1500	C_41_ H_45_ O_22_^+^	Cyanidin 3-[6-(6-ρ-coumarylglucosyl)- 2-xylosylgalactoside
	10	3.676	579.1687	399.1070, 401.1230, 417.1175	C_27_ H_31_ O_14_^+^	Pelargonidin 3-rhamnoside-5-glucoside
	11	4.207	449.1078	121.0653, 137.0589, 287.0553	C_21_ H_21_ O_11_^+^	Cyanidin 5-O-glucoside

^**a**^ The compound numbers represent the main anthocyanin peaks as indicated in Fig. 2C and D

^b^ The molecular formula and the compounds were deduced based on searching the m/z and MS/MS spectra of positive modes in “METLIN” database

Rt—Retention time

[M+H]^+^—Molecular ion

### The galloylated anthocyanins in *Excoecaria* showed slightly higher stability than non-galloylated anthocyanins in vitro

**Fig. 3:**
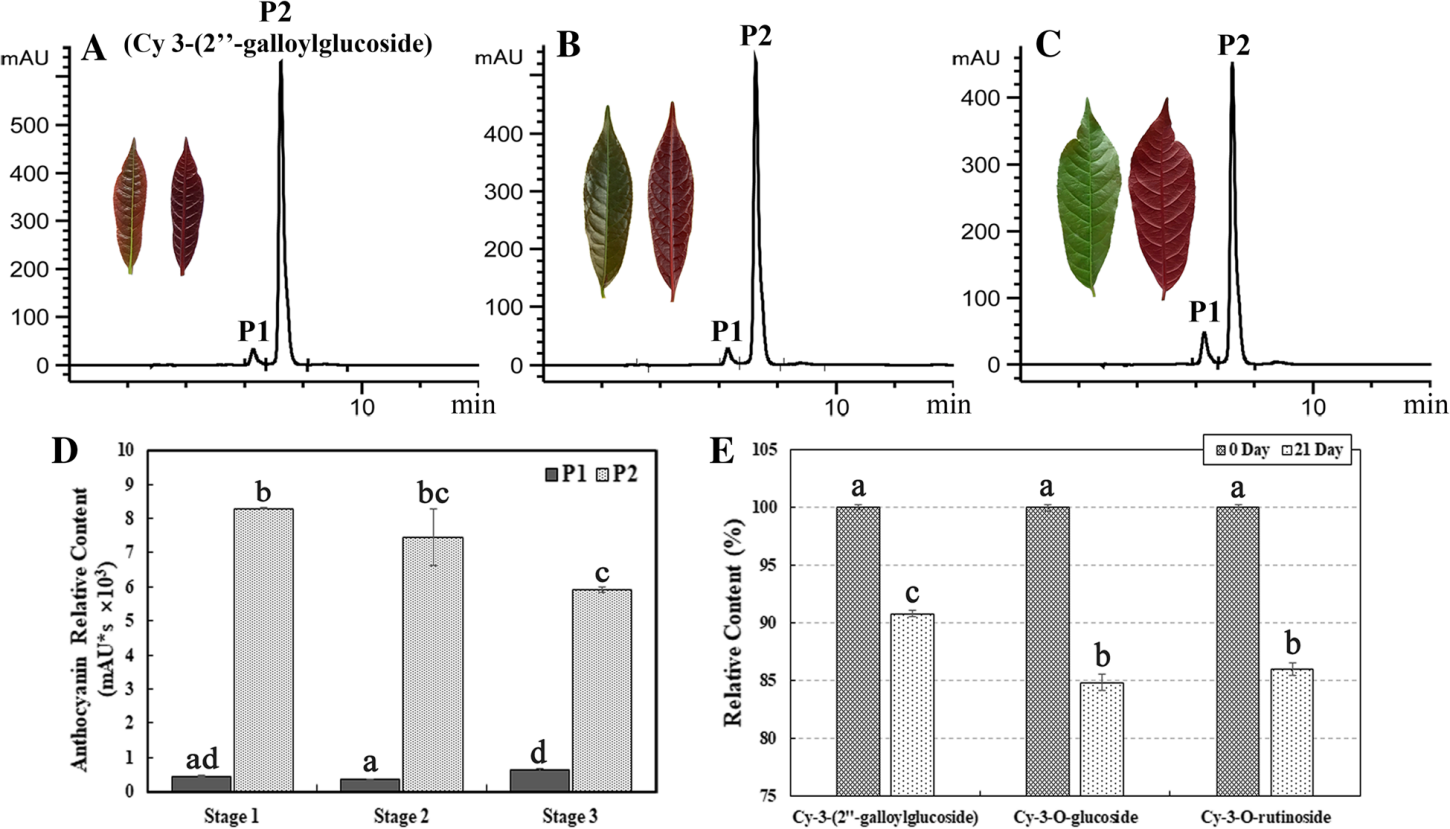
Stability of the major anthocyanins in *Excoecaria* leaves and in vitro. **A-C** Anthocyanin contents in *Excoecaria* leaves during maturation. Anthocyanin contents in *Excoecaria* leaves from stage 1 to 3 (as indicated in Fig. 1A) were analyzed by HPLC (A510nm). Peak 1 (P1) was further identified to be cyanidin-3-O-glucoside by UPLC-MS/MS (Additional file 1: Figure S1D), while Peak 2 (P2) was identified to be cyanidin 3-(2″-galloylglucoside) (Additional file 1: Figure S1E). **D** The relative content of P1 and P2 during leaf maturation in *Excoecaria* was represented by the peak area of the pigments. **E** In vitro stability of the major anthocyanins. The relative content of the three major anthocyanins as indicated in (A) and Fig. 2F was detected after staying at room temperature for 21 days. All the values were means of the measurements of three individual repeats, the statistical details of the values presented in (D) and (E) are as described in Fig. 1

The anthocyanin content in *Excoecaria* leaves were also measured by HPLC. Two major peaks of A510 nm, Peak 1 (P1) and Peak 2 (P2) were detected, with much higher abundance for P2 than P1 (Fig. 3A-C). The two pigment components showed marginal degradation during leaf maturation (Fig. 3D), correlating to the unchanged total anthocyanin contents during maturation in *Excoecaria* leaves (Fig. 1E). The anthocyanins in the two peaks were further identified by UPLC-DAD-QTOF-MS/MS, the major components in P1 and P2 were cyanidin 3-O-glucoside and cyanidin 3-(2″-galloyl- glucoside) respectively (Additional file 1: Figure S1D and E).

The above-mentioned major anthocyanins in *Excoecaria*, cyanidin 3-(2″-galloylglucoside) and cyanidin 3-O-glucoside, cyanidin 3-O-rutinoside in *Osmanthus* (Fig. 2F) were respectively purified by LH-20 column chromatography (Fig. 2A and B) for stability comparison in vitro. The relative content of the three anthocyanins in pH 3.0 buffer dropped to 91, 85 and 86% respectively, in relevance to the pigment levels at day 0, after 21 days at room temperature in vitro. Cyanidin 3-(2″-galloylglucoside) displayed slightly more stable than the other two pigments (Fig. 3E).

### Low H_2_O_2_ dependent anthocyanin degradation enzyme activity in *Excoecaria* leaves may be responsible for the pigment maintenance

**Fig. 4:**
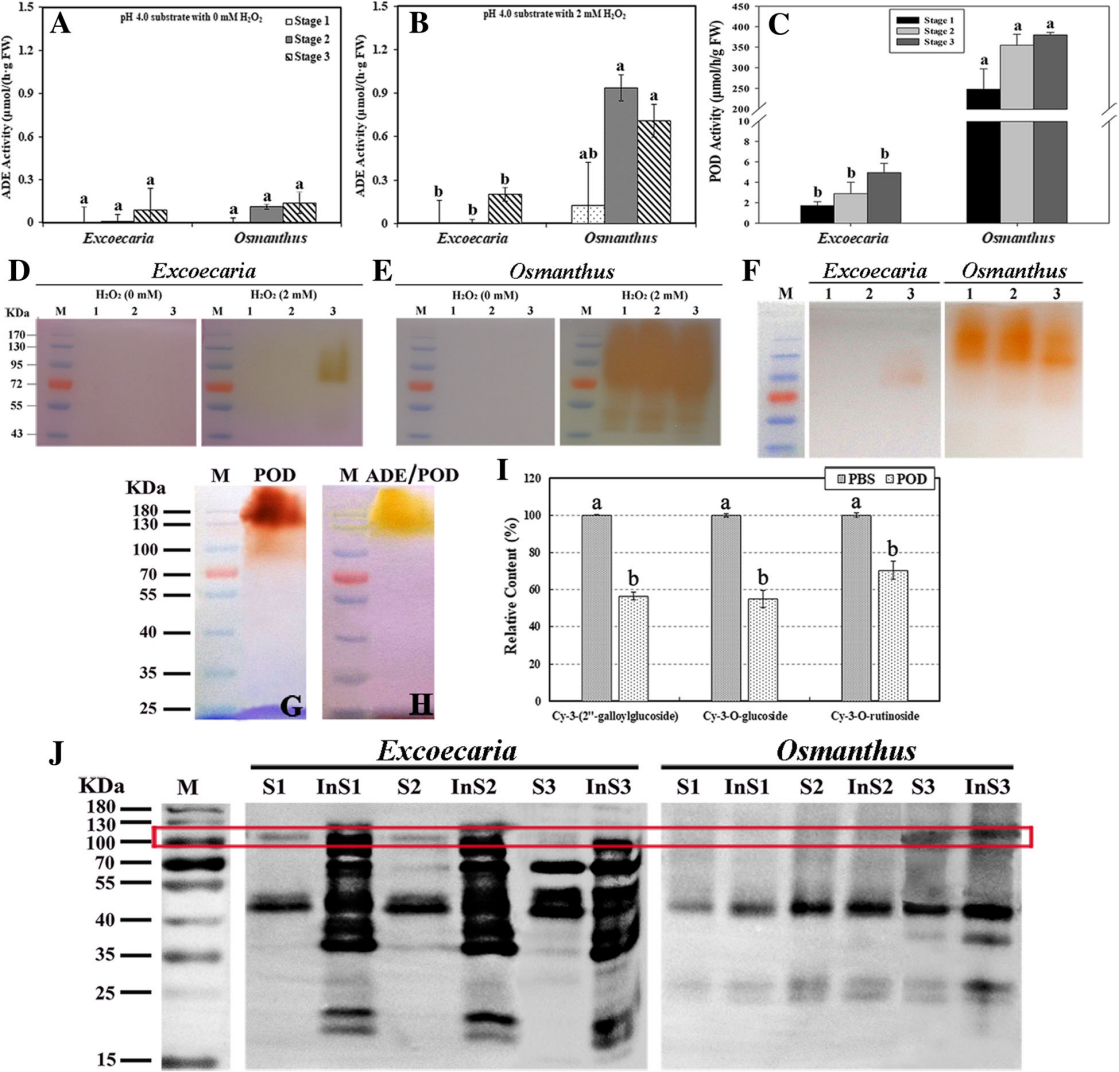
ADE/POD activity and protein levels during leaf maturation of *Excoecaria* and *Osmanthus*. **A-B** Anthocyanin degradation enzyme (ADE) activities. ADE activities of the three leaf developmental stages as indicated in Fig. 1A and B were determined with the anthocyanin substrates partially purified from *Excoecaria* and *Osmanthus* leaves respectively, at pH 4.0, with or without H_2_O_2_. **C** Activities of PODs. POD (peroxidase) activities were measured with guaiacol and H_2_O_2_ as substrates at pH 7.0. **D-E** In gel ADE activity assays. Ten micrograms of total proteins were separated by semi-native PAGE. The ADE activities were visualized by using the anthocyanin substrates as indicated in (A-B). Lane M indicated the protein markers, while Lane 1–3 represented the leaf samples as in (A-B). **F** In-gel POD activity assays. POD activity was visualized by immersing the gel in the substrate as described in (C). Lanes are as described in (D-E). **G-H** In-gel activity assay of the purified POD. POD was extracted and purified from *Osmanthus* leaves. The POD activity and its activity on anthocyanin degradation (ADE/POD) was visualized by using guaiacol (G) and partially purified *Osmanthus* anthocyanins (H) respectively as substrates in the presence of 2 mM H_2_O_2_. Lane M is as described in (D-E). **I** POD mediated degradation of the major anthocyanins. The major anthocyanins of *Excoecaria* and *Osmanthus*, as indicated in Fig. 3(A-C) and Fig. 2F, were purified respectively as described in Fig. 2A and B and added as substrates to the ADE/POD reactions as in (C), with denatured enzyme as control. The remained anthocyanin contents were determined after the reaction for 20 min. **J** Immuno-detection of PODs. POD protein abundance was detected in both soluble and insoluble fraction proteins from the leaf samples as in (A). The red frame indicates the size of the POD band as about 110 kDa. The details of the values presented in (A-C, I) are as described in Fig. 1

Anthocyanins were partially purified from the respective plants and used as substrates to detect the anthocyanin degradation enzyme (ADE) activities. Both H_2_O_2_ dependent and in-dependent ADE activities were monitored during leaf development (Fig. 4). The ADE activities in both plants were hardly detected without addition of H_2_O_2_ (Fig. 4A). When in the presence of H_2_O_2_, markedly higher ADE activities were detected for *Osmanthus* leaves than the activity in the absence of H_2_O_2,_ while the ADE activity detected for *Excoecaria* leaves remained low even in the presence of H_2_O_2_ (Fig. 4B).

The ADE activity patterns were further confirmed by the in-gel activity assays using the same anthocyanin substrates, with or without H_2_O_2_ at pH 4.0 (Fig. 4D and E). Correlated to the patterns, very weak H_2_O_2_ independent ADE activity signals were observed in the two plants while much more intensive activity staining was observed in the presence of H_2_O_2_ for *Osmanthus*. The H_2_O_2_ dependence of the ADE implies that the enzymes may be peroxidase (POD), correlating to the previous finding that PODs play an important role in anthocyanin degradation in planta [36]. The POD activities in the leaves were further determined by spectrophotometry (Fig. 4C) and in-gel methods (Fig. 4F). The activities detected by the two methods showed similar patterns, in which the POD activity was around 100-fold higher in *Osmanthus* leaves than *Excoecaria*. The POD activity was hardly detected using in-gel activity assay in *Excoecaria* at stage 1 and 2, and weak signal at around 100 kDa was observed only at stage 3, while intensive POD activity bands were detected for *Osmanthus* leaves at all the 3 stages (Fig. 4F). The major ADE activity band was of similar size as the POD activity band, both of around 100 kDa (Fig. 4E and F). Together with the H_2_O_2_ dependence of the enzyme, the ADE was actually POD, functioning in the anthocyanin degradation in leaves, which was designated as ADE/POD in the remainder of this article.

To better understand the maintenance of anthocyanins in *Excoecaria* leaves, peroxidase (ADE/POD) mediated degradation of the major *Excoecaria* anthocyanins were compared with the degradation of *Osmanthus* pigments. ADE/POD was partially purified from *Osmanthus* leaves by precipitation with ammonium sulfate at 70–100% saturation. In the presence of 2 mM H_2_O_2_, the partially purified ADE/POD fraction contained high ADE/POD activity that was visualized by in-gel activity assay, presenting as a major activity band of around 110 kDa, either with guaiacol (Fig. 4G) or *Osmanthus* anthocyanins as substrates (Fig. 4H). The POD fraction was therefore used for POD mediated degradation of anthocyanins identified in the two species. All the three major anthocyanins degraded rapidly after the ADE/POD was added (Fig. 4I). After 20 min at 25 °C, the relative content of cyanidin 3-(2″-galloylglucoside), cyanidin 3-O-glucoside and cyanidin 3-O-rutinoside, was recorded of 57, 55 and 70% as the content of the de-natured enzyme control, respectively (Fig. 4I). These results indicate that, even though the galloylated anthocyanin from *Excoecaria* is slightly more stable than the non-galloylated anthocyanins in vitro (Fig. 3E), the galloylated anthocyanin shows similar degradation by POD as the non-galloylated ones.

To understand the dramatical difference in ADE/POD activity between the two species, the protein levels of ADE/POD in the leaves were immune-detected by a polyclonal POD antibody. Protein bands of around 110 kDa were detected by the antibody in both soluble and insoluble fractions of the protein extracts from both plants. The 110 kDa-protein showed similar levels in the soluble fractions of both plants, while higher level in the insoluble fractions were detected in *Excoecaria* than *Osmanthus* at all stages (Fig. 4J). Since 110-kDa ADE/POD bands were both found in *Excoecaria* and *Osmanthus*, the dramatical difference in anthocyanin degradation patterns may be due to the inactivation of ADE/POD by unknown constituents in *Excoecaria* leaves.

### Natural POD inhibitors were isolated from *Excoecaria* leaves and constituted of galloylglucoses/ellagitannins

**Fig. 5:**
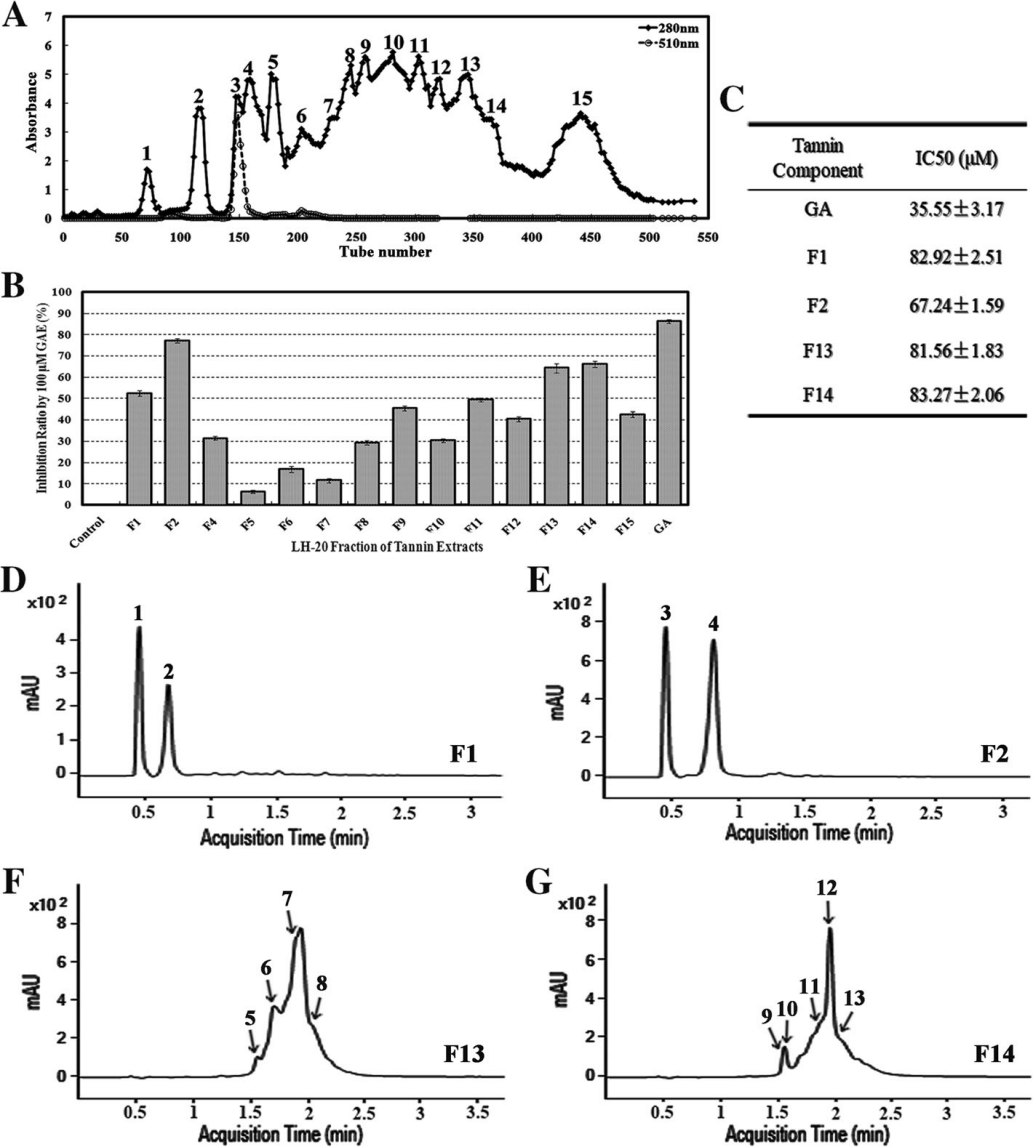
Isolation of natural POD inhibitors from *Excoecaria* leaves. **A** Separation of phenolic compounds from the *Excoecaria* leaf by Sephadex LH 20 column chromatography. Fifteen fractions were obtained based on the absorbance peaks at 280 nm of the chromatography profile. **B** The inhibition of the phenolic fractions on POD activity. The inhibition ratio on the POD activity indicated in Fig. 4G was measured by the addition of 100 μM GAE (gallic acid equivalent) of each fraction (A) to the activity assay reaction as described in Fig. 4C. **C** IC50 values of the fractions on POD. The half maximal inhibitory concentration (IC50) values of the fractions (F1, F2, F13, F14), which showed more than 50% inhibition on the POD activity as indicated in (B) and regarded to be of high inhibitory efficacy, were measured with gallic acid as a reference. **D-G** UPLC-DAD profiles of the high inhibition fractions represented by absorbance at 280 nm. The high inhibition fractions from *Excoecaria* leaves as indicated in (C) were respectively analyzed by UPLC-DAD

Phenolics in *Excoecaria* leaves were extracted and separated by Sephadex LH-20 column chromatography, so as to investigate whether natural POD inhibitors exist in the leaves. Fifteen fractions were obtained based on A280 nm peaks (Fig. 5A) and the phenolic content of each fraction was determined as gallic acid equivalent (GAE). The inhibition effects of 100 μM GAE of each fraction were investigated on the ADE/POD activity (Fig. 4G). Among these fractions and GA, F1, F2, F13, F14 and GA showed more than 50% inhibition rate, with 52.45, 77.22, 64.31, 66.08 and 83.36% inhibition respectively (Fig. 5B). The half maximal inhibitory concentration (IC50) values of GA, F1, F2, F13 and F14 on ADE/POD were estimated as 35.55, 82.92, 67.24, 81.56 and 83.27 μM GAE respectively, indicating the phenolic fractions from *Excoecaria* leaves contained natural ADE/POD inhibitors.

The compounds in the F1, F2, F13 and F14 fraction were further analyzed by UPLC-DAD-QTOF-MS/MS (Table 2 and Fig. 5D-G). Based on the fragment ions in the MS/MS graphs in negative modes (Additional file 2: Figure S2), GA fragments (78.01, 125.02 and 169.01) and HHDP (hexahydroxydiphenoyl) unit (300.99) were detected in the compounds of the 4 fractions, indicating the compounds are GA or HHDP derivatives (Table 2; Additional file 2: Figure S2). The major compounds in F1, both Cpd 1 and 2 were monogalloylglucose with mass of 332 Da. Both Cpd 3 and 4 in F2 were galloyl quinic acid with mass of 344 Da. In F13, Cpd 5 was identified as methyl gallic acid with mass of 184 Da, Cpd 6,7 and 8, were identified as three ellagitannins (HHDP derivatives, with mass of 952, 1104, 1118 Da respectively). Five compounds were identified in F14, including Cpd 9, another methyl gallic acid with mass of 184 Da; another two ellagitannins (Cpd 10 and 11, with mass of 952 and 966 Da respectively); two galloylglucoses (simple gallic acid derivatives), tetragalloyl hexoside (Cpd 12) and pentagalloyl hexoside (Cpd 13) (Table 2). Taken together, even though further identification of the compounds by other methods is required, the existence of galloyl/HHDP-moiety was confirmed in the major compounds of the fractions, indicating high levels of GGs/ETs in the phenolic fractions are responsible for the POD inhibition.

**Table 2:** Identification of the main POD inhibitors from *Excoecaria* leaves by UPLC-DAD-QTOF-MS/MS

Fraction^a^	Compound No.^b^	Rt(min)	Molecular Formula^c^	Molecular Mass	△mass (ppm)	m/z		Putative Name^c^
						[M-H]^−^	Fragment Ions	
F1	1	0.509	C_13_ H_16_ O_10_	332.0754	3.05	331.0682	151.0030; 169.0135; 211.0233; 331.0616	Galloyl hexoside [49] (Monogalloylglucose)
	2	0.726	C_13_ H_16_ O_10_	332.0745	0.59	331.0673	151.0032; 169.0135; 211.0235; 331.0642	Galloyl hexoside [49] (Monogalloylglucose)
F2	3	0.517	C_14_ H_16_ O_10_	344.0748	1.41	343.0676	125.0250; 169.0137; 191.0552; 343.0642	Galloyl quinic acid [49]
	4	0.859	C_14_ H_16_ O_10_	344.0749	1.49	343.0676	127.0398; 169.0136; 191.0549; 343.0642	Galloyl quinic acid [49]
F13	5	1.629	C_8_ H_8_ O_5_	184.0375	1.99	183.0303	78.0112;124.0168; 153.0219; 168.0070	Methyl gallic acid
	6	1.724	C_41_ H_28_ O_27_	952.0814	0.38	951.0738	300.9991; 461.0359; 583.0310; 933.0622	Ellagitannin (HHDP derivative [49])
	7	1.855	C_55_ H_28_ O_26_	1104.0936	0.76	1103.0859	300.9992; 445.0416; 631.0566; 951.0722	Ellagitannin [50] (HHDP derivative)
	8	2.002	C_56_ H_30_ O_26_	1118.1099	6.57	1117.1012	166.9995; 300.9995; 461.0346; 965.0902	Ellagitannin [50] (HHDP derivative)
F14	9	1.608	C_8_ H_8_ O_5_	184.0373	0.5	183.0560	78.0109;124.0164; 153.0175; 168.0058	Methyl gallic acid
	10	1.612	C_41_ H_28_ O_27_	952.0819	0.16	951.0747	169.0143; 300.9993; 461.0390; 933.0627	Ellagitannin (Galloyl HHDP derivative [49])
	11	1.862	C_42_ H_30_ O_27_	966.097	0.51	965.0897	300.9994; 461.0401; 805.9860; 933.0648	Ellagitannin [50] (HHDP derivative)
	12	1.885	C_34_ H_28_ O_22_	788.1076	0.42	787.1003	169.0143; 465.0666; 573.0867; 617.0789	Tetragalloyl hexoside (Tetragalloyl glucose) [49]
	13	2.017	C_41_ H_32_ O_26_	940.1182	3.83	939.1110	125.0247; 169.0146; 465.0682; 617.0791	Pentagalloyl hexoside (Pentagalloyl glucose) [49]

^**a**^ The fractions were of high inhibitory efficacy on POD as indicated in Fig. 5B

^**b**^ The compound numbers represent the main peaks as indicated in Fig. 5D-G

^c^ The molecular formula and the compounds were deduced based on searching the m/z and MS/MS spectra of negative modes in “METLIN” database

HHDP—Hexahydroxydiphenoyl unit

Rt—Retention time

[M-H]^−^—Molecular ion

### More hydrolysable tannins were identified in *Excoecaria* but not in *Osmanthus* leaves

**Fig. 6:**
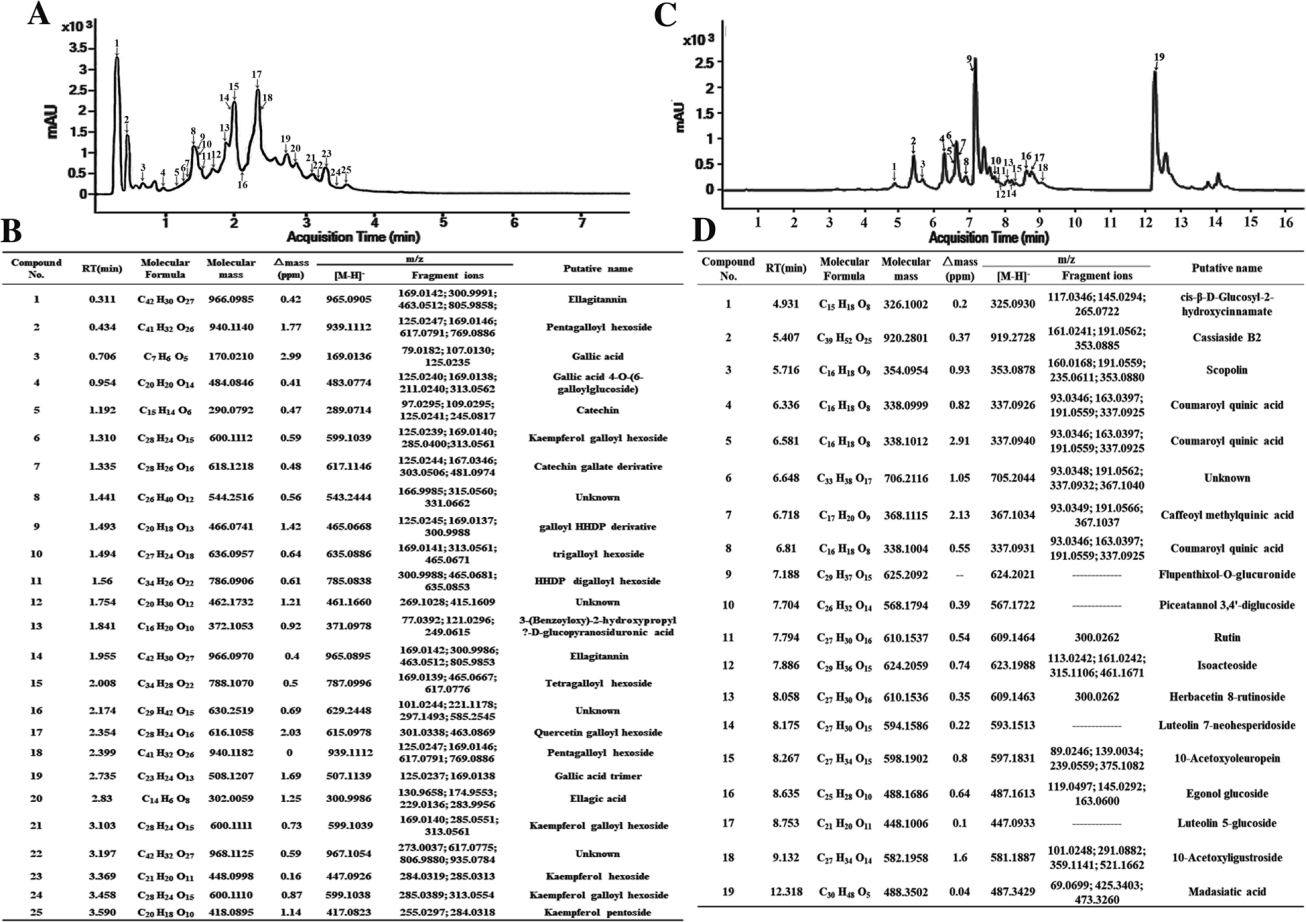
Non-targeted phenolic compound analysis of the leaves of *Excoecaria* and *Osmanthus* by UPLC-DAD-QTOF-MS/MS. **A** UPLC-DAD profiles (A280 nm) of the phenolic extract from *Excoecaria* leaves. Phenolic compounds were extracted from the *Excoecaria* leaves at stage 2 and subjected to UPLC-DAD-QTOF-MS/MS analysis. The peaks including the main phenolic compounds in (B) were numbered from 1 to 25. **B** Identification of the main phenolic compounds in *Excoecaria* leaves. The compounds in the extract were identified based on the MS and MS/MS spectra (Additional file 4: Data file 1), details are as described in Table 2. **C** UPLC-DAD profiles (A280 nm) of the phenolic extract from *Osmanthus* leaves. Total phenolic compounds were extracted from the *Osmanthus* leaves at stage 2 and subjected to UPLC-DAD-QTOF-MS/MS analysis. The peaks including the main phenolic compounds in (D) were numbered from 1 to 19. **D** Identification of the main phenolic compounds in *Osmanthus* leaves. The compounds in the extract were identified based on the MS and MS/MS spectra (Additional file 5: Data file 2), details are as described in Table 2

Prompted by the multiple GGs/ETs found in the phenolic fractions with high inhibitory efficacy on POD, we compared the phenolic compound profiling between *Excoecaria and Osmanthus* leaves by UPLC-DAD-QTOF-MS/MS analysis. The chromatogram profiles showed around 25 and 19 peaks of the absorbance at 280 nm in leaf extracts of *Excoecaria and Osmanthus* respectively (Fig. 6A and C). The detected compounds whose MS/MS spectra were successfully acquired and of high A280nm were subjected for alignment with the information obtained in Metlin databases (https://metlin.scripps.edu/), Chemspider (http://www.chemspider.com/) and Pubchem (https://pubchem.ncbi.nlm.nih.gov/). In *Excoecaria*, gallic acid (Cpd 3) and ellagic acid (Cpd 20) were identified with [M-H]^−^ ion at m/z 169.0136 and 300.9986 respectively, with characteristic daughter ion at m/z 125.0235 and 283.9956 respectively. Among the 25 identified compounds, 17 compounds (Cpd 1, 2, 3, 4, 6, 7, 9, 10, 11, 14, 15, 17, 18, 19, 20, 21, 24) were detected of fragments at m/z 169.0136 or 125.0235 or 300.9991. Together with the MS and MS/MS spectra alignments with the information obtained from the above-mentioned databases (Additional file 4: Data file 1), these compounds were predicted to be gallic acid, ellagic acid or HHDP derivatives, most of them were predicted to be GGs/ETs with galloyl (Cpd 1, 2, 4, 6, 9, 10, 14, 15, 17, 18, 19, 21, 24) or/and HHDP units (Cpd 1, 9, 11, 14), bounded to sugar moiety (Fig. 6B). Among the simple gallic acid derivatives or ellagitannins, high levels of ellagitannins (Cpd 1, 14), pentagalloyl hexoside (Cpd 2, 18) and tetragalloyl hexoside (Cpd 15) were also found in the *Excoecaria* fractions of high inhibitory efficacy on POD (Fig. 6B; Table 2). Compared to *Excoecaria*, hydrolysable tannins were not found in *Osmanthus* (Additional file 5: Data file 2). In contrast, some lignin constitutes, such as cinnamate in compound 1; coumaroyl in compound 4, 5 and 8; and caffeoyl in compound 7 were identified in *Osmanthus* (Fig. 6D).

### High levels of hydrolysable tannins co-localized with anthocyanins in *Excoecaria* inhibited the in vivo POD activity and increased the color intensity by copigmentation

**Fig. 7:**
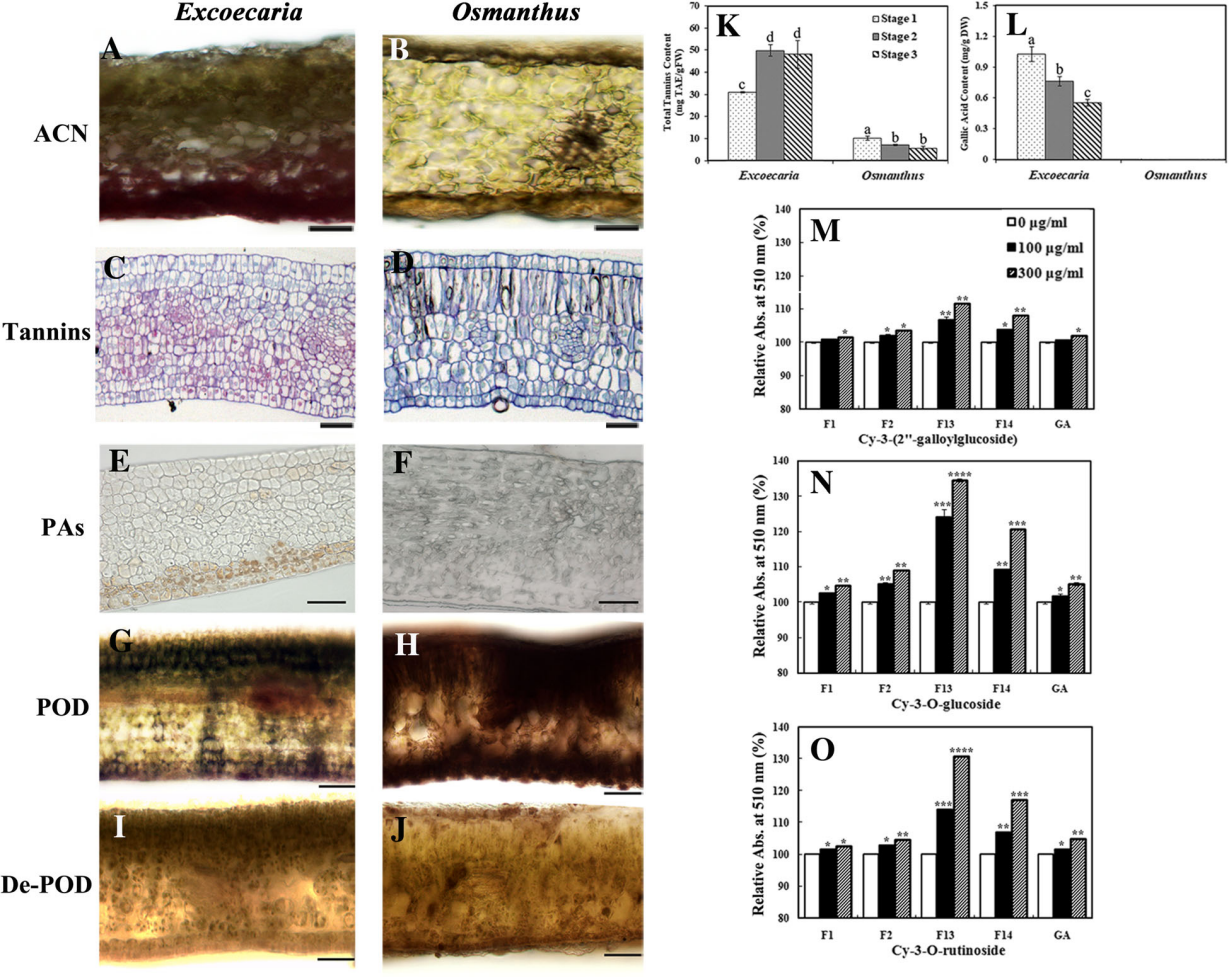
Localization of hydrolysable tannins in leaves and their copigmentation with anthocyanins and inhibition on POD activity. **A-B** Cross-sections of the stage 2 leaf blades of *Excoecaria* (A) and *Osmanthus* (B) for anthocyanin localization. Scale bars indicate 50 μm. **C-D** The combination of ruthenium red and toluidine blue staining of the sections (5-μm-thick) of the stage 2 leaf blades of *Excoecaria* (C) and *Osmanthus* (D). Scale bars indicate 100 μm. **E-F** The vanillin staining of the sections (5-μm-thick) of the stage 2 leaf blades of *Excoecaria* (E) and *Osmanthus* (F). Scale bars indicate 100 μm. **G-H** Histochemical localization of POD activity in the leaves. Cross-sections of *Excoecaria* (G) and *Osmanthus* (H) leaf blades at stage 2 were incubated with guaiacol and H_2_O_2_ at pH 7.0. Scale bars indicate 100 μm. **I-J** Control cross-sections for the POD histochemical localization. Cross-sections of *Excoecaria* (I) and *Osmanthus* (J) leaf blades as in (I-J) was boiled for 15 min for enzyme denature and served as the control. Scale bars indicate 100 μm. **K** Total tannin content as TAE (tannic acid equivalent) in the leaves of *Excoecaria* and *Osmanthus*. **L** Gallic acid content in the leaves determined by HPLC using gallic acid as standard (Additional file 3: Figure S3). The statistical details of the values presented in (K-L) were as described in Fig. 1. **M-O** Copigmentation of galloylglucoses/ellagitannins on the main anthocyanins in *Excoecaria* and *Osmanthus*. The relative absorbance at 510 nm of Cy-3-(2″-galloylglucoside) (M) and Cy-3-O-glucoside (N) from *Excoecaria* leaves, of Cy-3-O-rutinoside from *Osmanthus* leaves (O) after addition of 0, 100, 300 μg/mL GAE of the tannins. An asterisk (* p < 0.05) represents the significance of difference between the addition of 0 and 100 or 300 μg/mL GAE of the tannins. Two asterisks represent *p* < 0.01, three asterisks represent *p* < 0.001, four asterisks represent *p* < 0.0001. All the values were means of the measurements of three individual repeats

Most of the identified phenolics in *Excoecaria*, particularly for those of high POD inhibitory efficacy, were GGs/ETs belonging to hydrolysable tannins (Table 2 and Fig. 6B). Therefore, total tannin contents were determined in *Excoecaria* and *Osmanthus* leaves during leaf development. Increased tannin contents were found in *Excoecaria* along with the leaf maturation, while the content in *Osmanthus* leaves decreased during the process. Five folds higher tannin contents were detected in *Excoecaria* leaves than *Osmanthus* leaves at stage 2, with 49.94 and 7.14 mg TAE/g FW, respectively (Fig. 7K). High content of GA was detected in *Excoecaria* leaves at all stages, ranged between 0.55 to 1.01 mg/g DW. However, GA was not detected in *Osmanthus* at all the stages (Fig. 7L; Additional file 3: Figure S3).

The location of tannins in the leaves were observed by ruthenium red and toluidine blue staining. Strong pink-red staining was observed in 7–8 cell layer of the abaxial surface of *Excoecaria*, but not in *Osmanthus* cells (Fig. 7C and D), indicating high content of tannins accumulated in *Excoecaria* leaves. To better understand the levels of hydrolysable tannins in the leaf tissue, vanillin staining of condensed tannins (PAs) was carried out. Light brown staining was observed only in 2–3 cell layer of the abaxial surface of *Excoecaria*, but not in *Osmanthus* cells (Fig. 7E and F). The above result revealed large amount of hydrolysable tannins accumulated at the abaxial surface of *Excoecaria* leaves while none was in *Osmanthus* leaves. The result of frozen sections showed that anthocyanins were mainly located at the abaxial surface of *Excoecaria* leaves at the three stages (Fig. 1C, Fig. 7A), unlike the even distribution of anthocyanins at both the adaxial/abaxial surface of *Osmanthus* leaves at stage 1 and 2 (Fig. 1D, Fig. 7B). Furthermore, when leaf blade sections were incubated with POD substrates, dark-brown color appeared at the cells of both adaxial and abaxial surface in *Osmanthus* (Fig. 7H); while in *Excoecaria* leaves, browning was less intensive than in *Osmanthus* and only appeared at the adaxial surface (Fig. 7G). The results indicate that high POD activity was found for both sides of the leaves in *Osmanthus*, while the activity was hardly detected at the abaxial surface of *Excoecaria* leaves, correlating with the high level of tannins locating at the abaxial side of the leaves (Fig. 7C-J).

The copigmentation of hydrolysable tannins and anthocyanins were also investigated. After 0–300 μg/mL GAE of GA or the fractions (F1, F2, F13, F14) (Table 2) were added to *Excoecaria* and *Osmanthus* anthocyanin solutions (0.05 mM) at pH 3.0, significant increase in red color intensities of the solutions were observed (Fig. 7M-O), indicating copigmentation occurred between the GGs/ETs and anthocyanins. GGs/ETs produced more profound effect on the red color of cyanidin 3-O-rutinoside and cyanidin 3-O-glucoside than on the color of cyanidin 3-(2″-galloyl- glucoside) (Fig. 7M-O). F13 and F14, which contained high levels of GGs/ETs (Fig. 5F and G, Table 2), produced more enhancement in the color intensity than F1, F2 and GA (Fig. 7M-O). Addition of 300 μg/mL GAE F13 and F14 respectively led to 30.88 and 17.09% higher absorbance at 510 nm (A510 nm) for cyanidin 3-O-rutinoside (Fig. 7O); 34.57 and 20.83% higher A510nm for cyanidin 3-O-glucoside (Fig. 7N), 11.67 and 7.99% higher A510nm for cyanidin 3-(2″-galloylglucoside) (Fig. 7M).

Taken together, high levels of hydrolysable tannins were found to be co-localized with the anthocyanins at the abaxial surface of *Excoecaria* leaves, which significantly enhanced the color of the pigments and reduced the activity of the anthocyanin degradation related POD.

### High levels of hydrolysable tannins led to low pH values at the abaxial surface of *Excoecaria* leaves

**Fig. 8:**
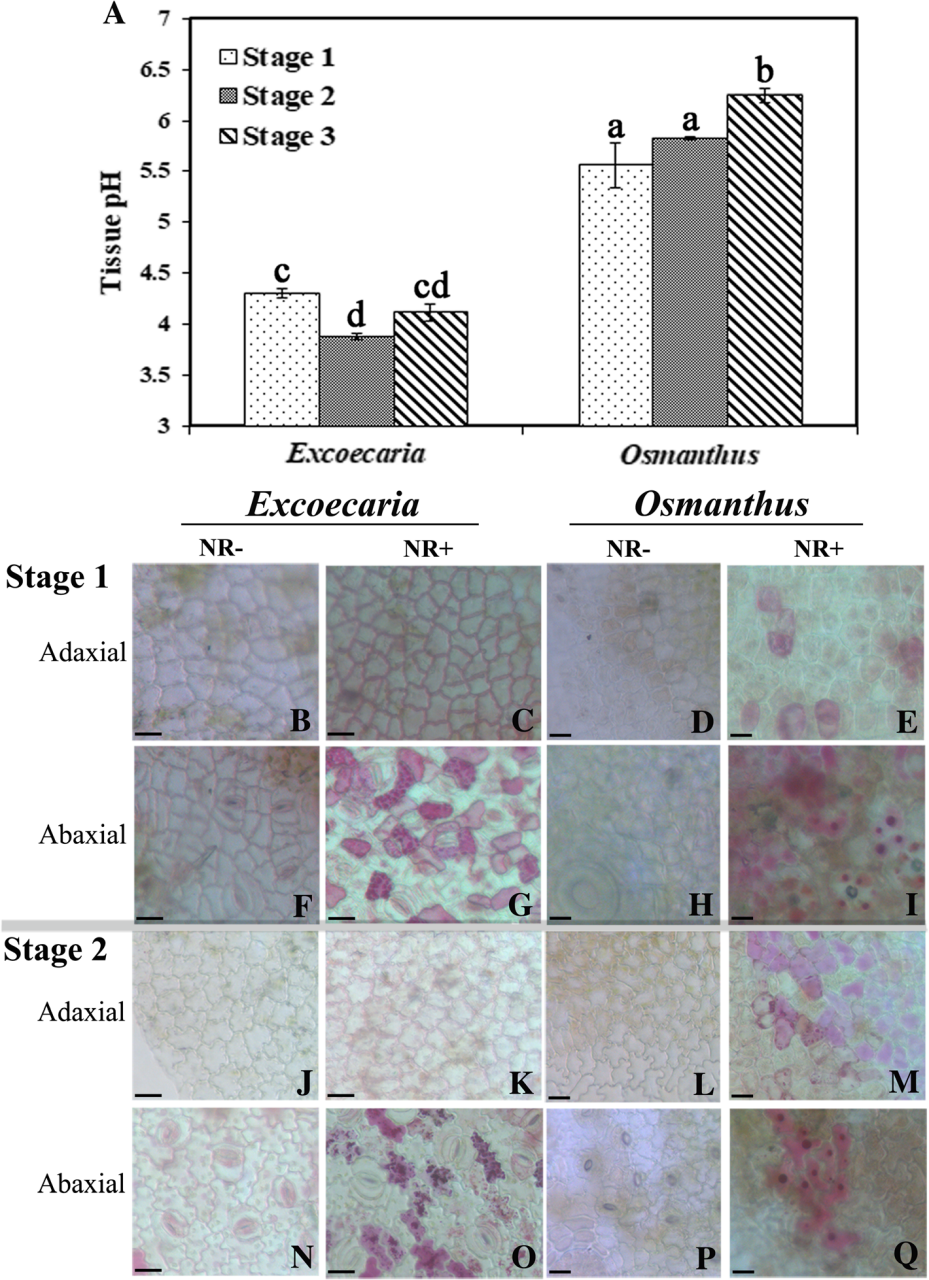
pH values of *Excoecaria* and *Osmanthus* leaves. (A) The pH values of the homogenate of *Excoecaria* and *Osmanthus* leaves in water. The details of the leaf developmental stages are as described in Fig. 1. **B-C, J-K** Neutral Red staining of the adaxial epidermal cells of *Excoecaria* leaves. The adaxial epidermal cells of *Excoecaria* leaves without Neutral Red (−NR) staining at stage 1 (B), stage 2 (J) and with NR (+NR) staining at stage 1 (C), stage 2 (K). **F-G, N-O** NR staining of the abaxial epidermal cells of *Excoecaria* leaves. The abaxial epidermal cells of *Excoecaria* leaves without NR staining at stage 1 (F), stage 2 (N) and with NR staining at stage 1 (G), stage 2 (O). **D-E, L-M** NR staining of the adaxial epidermal cells of *Osmanthus* leaves. The adaxial epidermal cells of *Osmanthus* leaves without NR staining at stage 1 (D), stage 2 (L) and with NR staining at stage 1 (E), stage 2 (M). **H-I, P-Q** NR staining of the abaxial epidermal cells of *Osmanthus* leaves. The abaxial epidermal cells of *Osmanthus* leaves without NR staining at stage 1 (H), stage 2 (P) and with NR staining at stage 1 (I), stage 2 (Q). Scale bars indicate 30 μm

Since the stability of anthocyanins is also dependent on the pH values of cells, the pH values in both plants were measured. Around 4.0 to 4.5 pH values were recorded in *Excoecaria* leaves, which were lower than the 5.5 to 6 pH values detected in *Osmanthus* leaves (Fig. 8A). The in vivo vacuole acidity of the epidermal cells was further measured by Neutral Red (NR) staining (Fig. 8B-Q). After staining, red coloration was only observed in the abaxial epidermal cells of *Excoecaria* leaves (Fig. 8G and O) while red coloration was observed in the epidermal cells at both sides of *Osmanthus* leaves (Fig. 8E, I, M and Q). The stained cells in *Excoecaria* exhibited much stronger accumulation of NR than the cells in *Osmanthus* (Fig. 8G, O, I and Q), indicating these *Excoecaria* cells were in a more acidic environment, which were expected to increase the stability of anthocyanin.

## Discussion

In general, anthocyanins are transiently accumulated in young leaves to protect the tissue from light damage, and the pigments are rapidly degraded after leaf maturation or in shape [1]. However, in some shape understorey plants, such as *Tradescantia* [51], *Begonia* [23], *Cyclamen* [22], and *Excoecaria* [47], anthocyanins are still maintained in the abaxial leaves after maturation, even in low light conditions [21, 52]. Anthocyanin maintenance in the abaxial surface of mature leaves plays important photoprotection roles for these understorey plants, while the mechanism of the anthocyanin maintenance is still unclear. In the present study, to investigate the potential factors in leaves that may prevent the anthocyanins from degradation in abaxially red leaves, we compared anthocyanin enzymatic degradation and the phenolic metabolites between a permanent pigmentation plant (*Excoecaria*) and a transient pigmentation plant (*Osmanthus*).

We first investigated whether the permanent and transient pigmentation in the *Excoecaria* and *Osmanthus* leaves respectively was due to the different type of anthocyanins. Indeed, we found high level of galloylated anthocyanins (anthocyanins acylated with gallic acid) in *Excoecaria* while all the pigments in *Osmanthus* are non-galloylated (Table 1, Fig. 2E and F). Galloylated anthocyanins were also found in red leaves of *Acer platanoides* “Crimson King” [53], red flowers of chenille plant, *Acalypha hispida* Burm. [54]. It has been reported that the anthocyanins carrying aromatic acyl substituents including *p*-coumaric, caffeic, ferulic, sinapic, gallic or *p*-hydroxybenzoic acids, displayed great stability due to intramolecular copigmentation [55–57]. Furthermore, acylated anthocyanins were reported to represent a much more efficient means of photoprotection by providing the plants with ultrafast energy dissipation and significant UV-B absorption capacity [58]. In the present study, cyanidin 3-(2″-galloylglucoside) was found to be more stable than cyanidin 3-O-rutinoside and cyanidin 3-O-glucoside (Fig. 3E). However, both galloylated and non-galloylated anthocyanins isolated from *Excoecaria* and *Osmanthus* respectively degraded rapidly in the presence of peroxidase (POD) (Fig. 4I). Considering enzymatic degradation may play more significant role than spontaneous breakdown in vivo tissue [35, 36], we speculated that other factors, rather than the stability of the pigments, may play more important roles in the prevention of pigment degradation in *Excoecaria* (Fig. 3E).

Studies have shown that PODs and laccases (LACs) are responsible for anthocyanin degradation in planta [35, 36]. In this paper, by using the partially purified anthocyanins from *Excoecaria* and *Osmanthus* young leaves respectively, strong H_2_O_2_ dependent ADE activities were detected for *Osmanthus* leaves but only trace activities for *Excoecaria* leaves (Fig. 4A-B, D-E). Interestingly, the size of ADE activity bands detected by in-gel assay (Fig. 4D and E) was of the similar size of the bands shown in the POD activity assay in gel (Fig. 4F), which confirmed that PODs played a major role in anthocyanin degradation in leaves [36, 59]. A POD antibody was used to prove that the ADE/POD protein levels in the leaves of the two species were not significantly different (Fig. 4J); However, the ADE/POD activities in *Excoecaria* were dramatically lower than the activities in *Osmanthus* (Fig. 4C and F), indicating inhibitors for ADE/PODs might exist in *Excoecaria leaves*.

Therefore, we isolated 15 fractions of phenolic components from *Excoecaria* leaves (Fig. 5A) and found that 4 of the fractions possessed high inhibitory effect on the ADE/POD activity (Fig. 5B and C). The results of UPLC-MS/MS further showed that the major components from the 4 fractions (F1, F2, F13, F14) were derivatives of gallic acid (GA), mostly were GGs/ETs (Table 2). The fractions enriched with GGs/ETs were found to inhibit the activity of ADE/POD with half maximal inhibitory concentration (IC50) estimated from 67.24 to 83.27 μM GAE, which was of slightly less inhibitory effect than GA (IC50 = 35.55 μM) (Fig. 5C). The results indicate that the naturally existed GA and GGs/ETs in *Excoecaria* leaves inhibit the POD mediated anthocyanin degradation. Inhibition of GA and its derivatives on POD was reported in a previous study, where gallic acid and ellagic acid acted as noncompetitive inhibitors with Ki values as 28.29 and 14.74 μM respectively to a bovine lacto-POD [60]. Ellagitannins have been reported to be effective inhibitors for many other enzymes, probably due to their ability to bind proteins [61]. Pomegranate ellagitannins, punicalagin, punicalin, and ellagic acid were identified as α-glucosidase inhibitors (IC50 of 140.2, 191.4, and 380.9 μM, respectively) [62]. Seven ellagitannins were isolated from rose bud extract powder and showed their inhibitory activities on dipeptidyl peptidase-IV (DPP-IV) in the same range as the ellagitannin fractions in the present study [63] (Fig. 5C). An ellagitannin from *Agrimonia pilosa* Ledeb showed significant inhibitory effect with an IC50 value of 17.03 ± 0.09 μM on protein tyrosine phosphatase [64]. In the present study, except for gallic acid and ellagic acid, multiple GGs/ETs were detected in *Excoecaria* leaves while neither gallic acid nor its derivatives were found in *Osmanthus* leaves (Fig. 6). The data are in agreement with the results that, although similar protein levels of POD were found between *Excoecaria* and *Osmanthus* (Fig. 4J), markedly lower activities were detected for the *Excoecaria* leaves (Fig. 4C). Furthermore, histochemical analysis showed extremely low POD activity was detected in abaxial surfaces of *Excoecaria* leaves, where hydrolysable tannins located (Fig. 7G-J), which further confirmed that the gallic acid derivatives and ellagitannins inhibited the activity of peroxidases mediating anthocyanin degradation in *Excoecaria* leaves (Fig. 5).

The stability of anthocyanins also depends on the presence of complexing agents (phenols, metalions) and pH [65]. Many studies have shown that copigmentation was an effective way to improve the color intensity and stability of anthocyanins [66–69]. Gallic acid is a well-known copigmentation agent effecting anthocyanin stability in cranberry juice [70], attributed to the shortest distance of its aromatic ring to the anthocyanin panel [71]. Several galloylglucoses and an ellagitannin were identified in the petals of *Geranium sylvaticum* and efficiently maintained the purple flower color through intermolecular co-pigmentation [72]. In the present study, high levels of simple gallic acid derivatives (galloylglucoses) and ellagitannins were found in *Excoecaria* leaves, but none was found in *Osmanthus* (Fig. 6). Gallic acid and the phenolic fractons containing GGs/ETs significantly increased red color intensity of anthocyanin solutions, while the GGs/ETs showed much more significant copigmentation effects than gallic acid (Fig. 7M-O). Furthermore, correlating to previous studies that the gallic acid derivatives caused a more acidic environment than many other organic acids [73, 74], more acid condition was detected for the *Excoecaria* leaves than *Osmanthus*, either by the method of pH value measurement of leaf homogenate (Fig. 8A), or by vacuole neutral red enrichment (Fig. 8B-Q). The fact that hydrolysable tannins and anthocyanins were co-localized on the abaxial side of *Excoecaria* leaves (Fig. 7A-F); that more acid condition was found for the abaxial surface than the adaxial surface of *Excoecaria* leaves (Fig. 8), suggested that the existence of gallic acid and its derivatives not only interacted with anthocyanins to increase their stability and color intensification, but also resulted in a low pH environment in the abaxial side of *Excoecaria* leaves to maintain red color.

## Conclusion

Taken together, anthocyanins accumulated in abaxial surface of tropical understorey plants provide photoprotection during intermittent exposure to high-intensity sunlight (i.e. sun-patches). Based on the data of the present study, we suggest that a distinct group of phenolics, GGs/ETs, that abundantly accumulated at the abaxially layers of *Excoecaria* leaves inhibit POD mediated anthocyanin degradation and increase stability and color intensity of anthocyanins by copigmentation, leading to the permanent maintenance of the abaxial red leaves.

## Materials and methods

### Plant materials

*Excoecaria cochinchinensis* Lour. and *Osmanthus fragrans* var. semperflorens leaves at three stages (red, greening, green, indicated as stage 1, 2, 3 in Fig. 1A and B) were collected respectively in the campus of South China Agricultural University, Guangzhou City, the South-East of China (23°7′ N, 113° E). The sampled leaves were either subjected to analysis immediately or frozen in liquid N_2_ and stored under − 80 °C until use.

### Anthocyanin and chlorophyll content determination

Quantification of anthocyanin was performed as described [75]. The absorbance of the chlorophyll-removed anthocyanin extracts was measured by a spectrophotometer (Shimadzu UV-2450, Kyoto, Japan) at 530 nm and 645 nm. Anthocyanin content was calculated as (A_530_–0.25 **×** A_645_) g^− 1^ fresh weight (FW).

To further measure the content of each major anthocyanin during maturation in *Excoecaria* leaves, the anthocyanin extracts were filtered through 0.22 μm hydrophobic polyvinylidene difluoride (PVDF) membrane (ANPEL Scientific Instruments, Shanghai, China) before subjected to HPLC analysis. Separation of the anthocyanins was performed in an Agilent 1200 Series HPLC system (Agilent Tech., Santa Clara, CA, USA). The extracts were injected into a C18(2) column (Luna®, 5 μm, 250 × 4.6 mm, Phenomenex, Torrance, CA, USA). The mobile phase consisted of 0.1% formic acid (FA) in acetonitrile (A) and 0.1% FA in water (B). Gradient elution at a flow rate of 0.8 mL/min was used from 10 to 45% A over 35 min at 35 °C. The injection volume was 20 μL. The monitoring wavelength was recorded at 530 nm. The relative content of each major anthocyanin (the major peak fractions respectively collected by the HPLC and further identified by the UPLC-QTOF-MS/MS as described in “*Anthocyanin profiling*”) during maturation in *Excoecaria* leaves was calculated according to the peak area.

For chlorophyll (Chl) content determination, frozen leaves (1 g) were ground in liquid nitrogen and placed into 10 mL cold aqueous acetone (80%, v/v) overnight in the dark at 4 °C. After centrifugation at 10,000 g for 10 min, the residue was re-extracted with the cold aqueous acetone until it became colorless. All the supernatants were combined and brought to 20 mL. Then the absorbance of the Chl supernatant was measured by the spectrophotometer at 663 nm and 645 nm. The Chl concentration per fresh weight of the leaves was calculated as described [76].

### Anthocyanin profiling

Fifty grams of the red leaves of *Excoecaria* and *Osmanthus* were respectively blanched with 350 mL of 0.3 M HCl solution by multiple soaking for anthocyanin extraction. Then the crude anthocyanin extracts were partially purified by Amberlite XAD-7 resin (Sigma-Aldrich, Saint Louis, MO, USA) column (1.5 × 40 cm) as described [77]. The red fractions were collected and concentrated by a rotary evaporator (Heidolph, Schwabach, Germany). The concentrated anthocyanins were dissolved in 4 mL of 10% (v/v) FA and then loaded onto a column filled with Sephadex LH 20 (5.5 × 90 cm, Sigma) for further purification. Elution was performed with 10% FA as eluent at a flow rate of 1 mL/min. Fractions (2 mL/tube) were collected with a fraction collector. Elution was monitored by the spectrophotometer at 510 nm. The fractions of the major peak, based on the absorbance values at 510 nm, were pooled and purified again by Amberlite XAD-7 resin to exchange the solvent from 10% FA to 0.05% FA in methanol and then concentrated. The purified anthocyanins were filtered through the 0.22 μm PVDF membrane and transferred to a vial before analysis.

To identify the purified anthocyanins, high performance liquid chromatography (HPLC) separation coupled with diode array detection (DAD) and electrospray ionization mass spectrometer (ESI/MS) was performed as described [78], in a UPLC1290-6540B Q-TOF (Agilent Tech., Singapore), coupled with a 6540 UHD Q-TOF ESI Mass spectrometer (Agilent Tech., Singapore). The chromatographic separation was achieved on an Agilent eclipse plus 50 × 2.1 mm, 1.8 μm column and the monitoring wavelength was recorded at 280 nm. The mobile phase consisted of acetonitrile (A) and 0.2% FA in water (B). Gradient elution at a flow rate of 0.4 mL/min was used from 10 to 90% A at 35 °C. The MS was recorded with a heat capillary voltage of 4 kV, spectra were recorded in positive ion mode between m/z 100 and 1500 with heated dry nitrogen gas at temperature 300 °C and flow rate 8 L/min was used. Nitrogen was used as the nebulizing gas (40 psi) and the fragmentation voltage was 160 V. The identification of the compounds were based on the searching of m/z values of the compounds in Metlin databases (https://metlin.scripps.edu/) and comparison of the fragment ions of MS/MS in the databases of Chemspider (http://www.chemspider.com/) and Pubchem (https://pubchem.ncbi.nlm.nih.gov/).

### Total tannin content measurement

The extraction and determination of total tannins were carried out as described [39]. Tannin content was expressed as milligrams of tannic acid (gallotannin, Sigma) equivalents per gram fresh tissue (mg TAE/g FW).

### Anthocyanin location and staining of tannins in the leaf tissue

Hand cross sections of leaf blades of *Excoecaria* and *Osmanthus* were respectively immersed in ddH_2_O and observed for pigment distribution by a light microscopy (Leica Microsystems, Germany).

Staining of tannins in the leaf tissue was performed according to the method as described [79]. Firstly, the fixation and sectioning were carried out as described [39]. Briefly, discs (2 mm × 2 mm) of *Excoecaria* and *Osmanthus* leaves were respectively fixed for 12 h in 100 mM phosphate buffer (PBS, pH 7.2) containing 4% (w/v) paraformaldehyde, 2% (w/v) glutaraldehyde. After washed in PBS and dehydrated and infiltrated in gradient ethanol and LRWhite resin (Sigma), respectively, the mixture was finally replaced by pure LRWhite. Samples were then transferred to capsules with fresh LRWhite, and cured under two 15-watt ultraviolet lamps (360 nm) for at least 24 h at − 20 °C, and then continued curing for 48 h at RT [39]. For tannins staining, 5-μm-thick sections obtained on a microtome (Leica Microsystems, Wetzlar, Germany) were incubated in ruthenium red (0.05% aqueous) for 2 min and then washed before incubated again in toluidine blue (0.1% aqueous) for 1 min [79]. Observations and photographs were done on a light microscope (Optiphot, Nikon, Tokyo, Japan). To better understand the levels of hydrolysable tannins out of the above tannins staining in the leaves, vanillin staining of condensed tannins (PAs) in plant tissues was also performed as described [39].

### Non-targeted phenolic compound analysis by UPLC-DAD-QTOF-MS/MS

Phenolics were extracted as described [80], using 70% (v/v) of aqueous acetone containing 1% (w/v) benzothiadiazole (BTH) as extraction solution. The final extracts (100 μL) were dried under the nitrogen stream, re-dissolved in 1 mL 60% (v/v) ethanol, filtered through the 0.22 μm PVDF membrane and transferred to a vial before analysis.

Non-target profiles of the above phenolic extracts from the leaves of *Excoecaria* and *Osmanthus* were further respectively characterized by UPLC-QTOF-MS/MS as described in “*Anthocyanin profiling*”, while the MS was recorded with a heat capillary voltage of 3.5 kV, spectra were recorded in negative ion mode between m/z 100 and 1100. The fragmentation voltage was 150 V.

### Column chromatography of the phenolic compounds with Sephadex LH 20

The above phenolic extracts from the leaves were also purified by Sephadex LH 20 (1.5 × 70 cm) column chromatography. The dried crude extracts (1 g dried sample extraction for described column dimension) were first dissolved in 2 mL aqueous 3% (v/v) FA and the insoluble residue was then dissolved in 1 mL methanol. The 3 mL sample was loaded onto the column that had been equilibrated with aqueous 3% (v/v) FA. Elution was performed with gradient aqueous methanol, from 20 to 100%, at a flow rate of 20 mL/h. Fractions (3 mL/tube) were collected with a fraction collector.

Based on the peaks of absorbance values at 280 nm (Fig. 5A), the fractions of the phenolic extracts were combined and grouped to 15 big fractions namely F1-F15. Total phenolic content of the 15 fractions was determined using Folin-Ciocalteu assay as described [39]. The content was calculated as gallic acid equivalents (μg/mL GAE) by using gallic acid calibration curve.

### Gallic acid content determined by HPLC

Phenolics in the three stages of *Excoecaria* and *Osmanthus* leaves were respectively extracted and prepared as described in “*Non-targeted phenolic compound analysis by UPLC-DAD-QTOF-MS/MS*” [80]. The filtered extracts of each sample were separated by HPLC as described [39]. The temperature of the column was 25 °C. The peak of the gallic acid (GA) in each sample was identified by comparison with the retention time of standard gallic acid (Sigma-Aldrich), and was further confirmed by a standard addition method. Briefly, 2, 4 μg/mL standard gallic acid was added respectively in the sample extracts and the increase in the peak area of gallic acid was monitored. The gallic acid content [mg/g DW (dry weight)] in the crude extracts of each sample was calculated according to the gallic acid standard curve.

### Enzyme extraction, SDS-PAGE and POD immunodetection

Crude enzyme was extracted by homogenizing the leaves (2 g) with 8 mL of 0.1 M pH 7.0 potassium phosphate buffer (KPB), containing 30% (w/w) Polyvinylpolypyrrolidone (PVPP) and 80 μL of protease inhibitor solution [1 tablet of protease inhibitor (cOmplete™, Mini, Ethylenediaminetetraacetic acid (EDTA)-free Protease Inhibitor Cocktail, Roche, Mannheim, Germany) was dissolved in 10 ml KPB]. After centrifugation (20 min, 12,000 g, 4 °C), the supernatants were collected as crude enzyme extract or soluble fraction of the protein extract [81]. The residue from the above extraction was resuspended and homogenized in the above KPB containing 8 M urea. After centrifugation, the supernatants were collected as insoluble fraction of the protein extract [39]. Protein concentration was determined using Coomassie Brilliant Blue G-250.

Crude enzyme extract was denatured in Laemmli’s sample buffer by 10 min of boiling prior to separation in 10% (w/v) sodium dodecyl sulfate-polyacrylamide gel electrophoresis (SDS-PAGE) according to standard conditions [39].

Immunodetection for POD level in the leaves was performed as described [39], using the polyclonal anti-Horseradish POD antibody (Agrisera Antibody, Vännäs, Sweden).

### Anthocyanin degradation enzyme/peroxidase (ADE/POD) activity assay

Anthocyanins in *Excoecaria* and *Osmanthus* leaves were respectively extracted and partially purified as described in “*Anthocyanin profiling*”. The concentration of anthocyanins was determined using the pH-differential method as described [82]. Two anthocyanin substrates for ADE/POD activity assay were prepared respectively diluting the concentrated anthocyanins using 0.2 M sodium acetate buffer (pH 4.0) to final concentration 0.05 M.

For ADE/POD activity using the anthocyanin substrates, crude enzyme extracts (0.1 mL) were added to 2.0 mL of the above anthocyanin substrates with or without 2 mM H_2_O_2_. The mixture was incubated for 20 min at 40 °C. The reaction was terminated by adding 2 mL of 0.1 M HCl in methanol. The decrease in absorbance due to degradation of anthocyanins in the reaction mixture was recorded at 530 nm, compared to the reaction set up in parallel with denatured enzyme. As described [40], one unit of ADE activity was defined as the amount of ADEs needed for degradation of 1 μmol of cyanidin-3-glucoside (ε = 29,600 M^− 1^ cm^− 1^) per hour at 40 °C. ADE activity was expressed as μmol h^− 1^ g^− 1^ FW.

For POD activity using the guaiacol substrate, the activity was measured using guaiacol as a substrate in a reaction mixture (3 mL), containing 0.05 mL crude enzyme extract, 2.75 mL 50 mM phosphate buffer (pH 7.0), 100 μL of 150 mM H_2_O_2_ and 200 μL of 200 mM guaiacol. As described [83], the increase in absorbance due to oxidation of guaiacol in the reaction mixture was monitored at 470 nm (ε = 6740 M^− 1^ cm^− 1^) for 2 min by the spectrophotometer. POD activity was expressed as μmol/h^− 1^ g^− 1^ FW (1 μmol of substrate conversion/h^− 1^ g^− 1^ FW).

ADE/POD activity gel staining using the two substrates with or without H_2_O_2_ was carried out after PAGE as described above but without protein sample boiling namely semi-native PAGE. The gel was then rinsed twice with the buffer for 5 min to remove SDS and immersed in the same buffer containing the substrates with 2 mM H_2_O_2_ or without H_2_O_2_ until the bands were visualized.

### Partial purification of peroxidase from *Osmanthus* leaves

Eighty grams of frozen *Osmanthus* leaves were ground and immediately homogenized in 300 mL of 0.1 M KPB (pH 7.0), containing 8.6 mM Dithiothreitol (DTT), 5 mM EDTA, 1 mM phenylmethylsulfonyl fluoride (PMSF) and 5% (w/v) PVPP, followed by centrifugation at 12,000 g for 20 min [39]. The extract was fractionated further by precipitation with ammonium sulfate at 70 to 100% saturation and centrifugation at 12,000 g for 20 min. The precipitate was re-suspended again and dialyzed against 0.01 M KPB (pH 7.0) containing 2.2 mM DTT.

### The stabilities of anthocyanins in vitro

The three major anthocyanins purified and identified in “*Anthocyanin profiling*” and shown in Fig. 2 were freeze-dried and re-dissolved in 0.2 M sodium acetate buffer (pH 4.0). ADE/POD activity assay was carried out as described below, the purified *Osmanthus* POD (5 μL) were added to 125 μL of the above prepared substrates with 1 μL of 1 mM H_2_O_2_. The mixture was incubated for 20 min at 25 °C. The reaction was terminated by adding 125 μL chloroform. After centrifugation (13,000 g, 5 min), relative content of the remained anthocyanins in the supernatant was determined by HPLC at 510 nm as described in “*Anthocyanin and chlorophyll content determination*”, compared to the reaction set up in parallel with denatured enzyme.

The anthocyanins (0.05 mM) were prepared respectively in 0.4 M citric acid-disodium hydrogen phosphate buffer at pH 3.0 and placed at room temperature for 21 days. The absorbance at 510 nm of each anthocyanin solution was recorded at 0 day and 21 days respectively to analyze the stabilities of the different types of anthocyanins**.**

### Determination of IC50 values and copigmentation effect for the tannin constituents

The half maximal inhibitory concentration (IC50) of the tannin constituents on the purified *Osmanthus* POD (0.6 μg) was tested by 0, 10, 20, 40, 60, 80, 100 μM GAE of the F1, F2, F13, F14 sub-fractions and GA. The POD activities were measured as described above and the inhibition ratio was obtained by calculating the percentage of the decrease in the activity due to the addition of the tannin constituents, to the activity without addition.

The F1, F1, F2, F13 and F14 four fractions of 0, 100, 300 μg/mL GAE were added to the anthocyanin solutions respectively, with same level of gallic acid as a reference. The absorbance at 510 nm of each mixture was recorded 2 h after addition to analyze the effect of tannin constituents on color intensities of anthocyanins**.**

### Histochemical staining and localization of ADE/POD in the leaf tissue

Leaf blade cross sections were hand cut using a razor blade, rinsed in cold PBS (phosphate buffer) (50 mM, pH 7.0), and incubated for 20 min at 25 °C with catalase (100 mg/mL; Sigma-Aldrich) to eliminate endogenous hydrogen peroxide. Samples were then rinsed several times to remove the catalase and incubated at 25 °C in the PBS containing 13.3 mM guaiacol and 5 mM H_2_O_2_. The controls were cross sections boiled for 15 min to destroy enzyme activity. The treated cross sections were observed by the light microscopy [35].

### Tissue cell sap pH and vacuolar acidity detection by neutral red staining

The leaf tissue cell sap pH was determined as described [84]. The pH of the homogenate was measured by a pH meter (Satorious, Göttingen, Germany). Neutral red (NR, Sigma) was used to detect the subvacuolar acidity of the leaf cells [85]. Fresh leaf tissue slices of *Excoecaria* and *Osmanthus* were respectively immersed in NR solution (20 mg/L) and incubated for 20 min at room temperature. The adaxial/abaxial epidermal cells of NR-stained and non-stained tissue slices were observed by the light microscopy.

### Statistical analysis

Data were collected from three biological replicate samples. Values were presented as means ± standard error of mean (SEM). Means were compared by unpaired t test (*P* < 0.05) using the GraphPad QuickCalcs online software (Prism 7, GraphPad Software, San Diego, CA, USA).

## Additional files

Additional file 1: **Figure S1**. HPLC profiles of the anthocyanins in *Excoecaria* leaves and identification by UPLC-DAD-QTOF-MS/MS. (A-C) Anthocyanin contents in *Excoecaria* leaves during maturation. Anthocyanin contents in *Excoecaria* leaves from stage 1 to 3 (as indicated in Fig. 1A) were analyzed by HPLC (A510nm). (D) Peak 1 (P1) identified by UPLC-DAD-QTOF-MS/MS. P1 as indicated in (A-C) was further identified to be Cy-3-O glucoside. (E) Peak 2 (P2) identified by UPLC-DAD-QTOF-MS/MS. P2 as indicated in (A-C) was further identified to be cyanidin 3-(2″-galloylglucoside). (TIF 10184 kb)

Additional file 2: **Figure S2**. Identification of the Major POD inhibitors from *Excoecaria* leaves by UPLC-DAD-QTOF-MS/MS. (A-D) Characterization of the compounds in the high inhibition fractions by UPLC-DAD-QTOF-MS/MS. The MS and MS/MS spectra of the major compounds with high absorbance at 280 nm indicate various hydrolysable tannins in the high inhibition fractions of F1 (A), F2 (B), F13(C) and F14 (D) as described in Fig. 5A and B. (TIF 10206 kb)

Additional file 3: **Figure S3**. Gallic acid content determination by HPLC using internal standard method. (A, E) HPLC analysis of standard gallic acid. (B, F) HPLC analysis of gallic acid in the phenolic extracts of the leaves at stage 2 of *Excoecaria* (B) and *Osmanthus* (F). (C-D) HPLC analysis of gallic acid in the phenolic extracts of the leaves at stage 2 of *Excoecaria*, with 2 μg/mL (C) and 4 μg/mL (D) standard gallic acid addition in the extracts. (G-H) HPLC analysis of gallic acid in the tannin extracts of the leaves at stage 2 of *Osmanthus*, with 2 μg/mL (G) and 4 μg/mL (H) standard gallic acid addition in the extracts. (TIF 9908 kb)

Additional file 4: **Data file 1**. MSMS compound report of *Excoecaria* phenolics used in this study. (PDF 21926 kb)

Additional file 5: **Data file 2**. MSMS compound report of *Osmanthus* phenolics used in this study. (PDF 6486 kb)

## Data Availability

All data generated or analyzed in this study are included in this published article and its additional files.

## Abbreviations

[M+H]^+^: Molecular ion
[M-H]^−^: Molecular ion
ADE: Anthocyanin degradation enzyme
BTH: Benzothiadiazole
Cpd: Compound
DAD: Diode array detection
DPP: Dipeptidyl peptidase
DTT: Dithiothreitol
DW: Dry weight
EDTA: Ethylenediaminetetraacetic acid
ESI/MS: Electrospray ionization mass spectrometer
FA: Formic acid
GA: Gallic acid
GAE: Gallic acid equivalents
GGs/ETs: Galloylglucoses/ellagitannins
HHDP: Hexahydroxydiphenoyl
HPLC: High performance liquid chromatography
IC50: The half maximal inhibitory concentration
KPB: Potassium phosphate buffer
LAC: Laccase
NPQ: Non-photochemical quenching
NR: Neutral red
P: Peak
PAs: Proanthocyanidins/Condensed tannins
PBS: Phosphate buffer
PMSF: Phenylmethylsulfonyl fluoride
POD: Peroxidase
PPOs: Polyphenol oxidases
PVDF: polyvinylidene difluoride
PVPP: Polyvinylpolypyrrolidone
Rt: Retention time
SDS-PAGE: Sodium dodecyl sulfate-polyacrylamide gel electrophoresis
SEM: Standard error of mean
TAE: Tannic acid equivalents
UPLC-QTOF-MS/MS: Ultra-performance liquid chromatography quadrupole time of flight mass spectrometry
VAZ/Chl: Total xanthophyll to chlorophyll
