# Supplementary figures and images for: In planta high levels of hydrolysable tannins inhibit peroxidase mediated anthocyanin degradation and maintain abaxially red leaves of Excoecaria Cochinchinensis
Source: BMC Plant Biol. 2019 Jul 15;19:315. doi: 10.1186/s12870-019-1903-y (PMC6632198; doi:10.1186/s12870-019-1903-y)

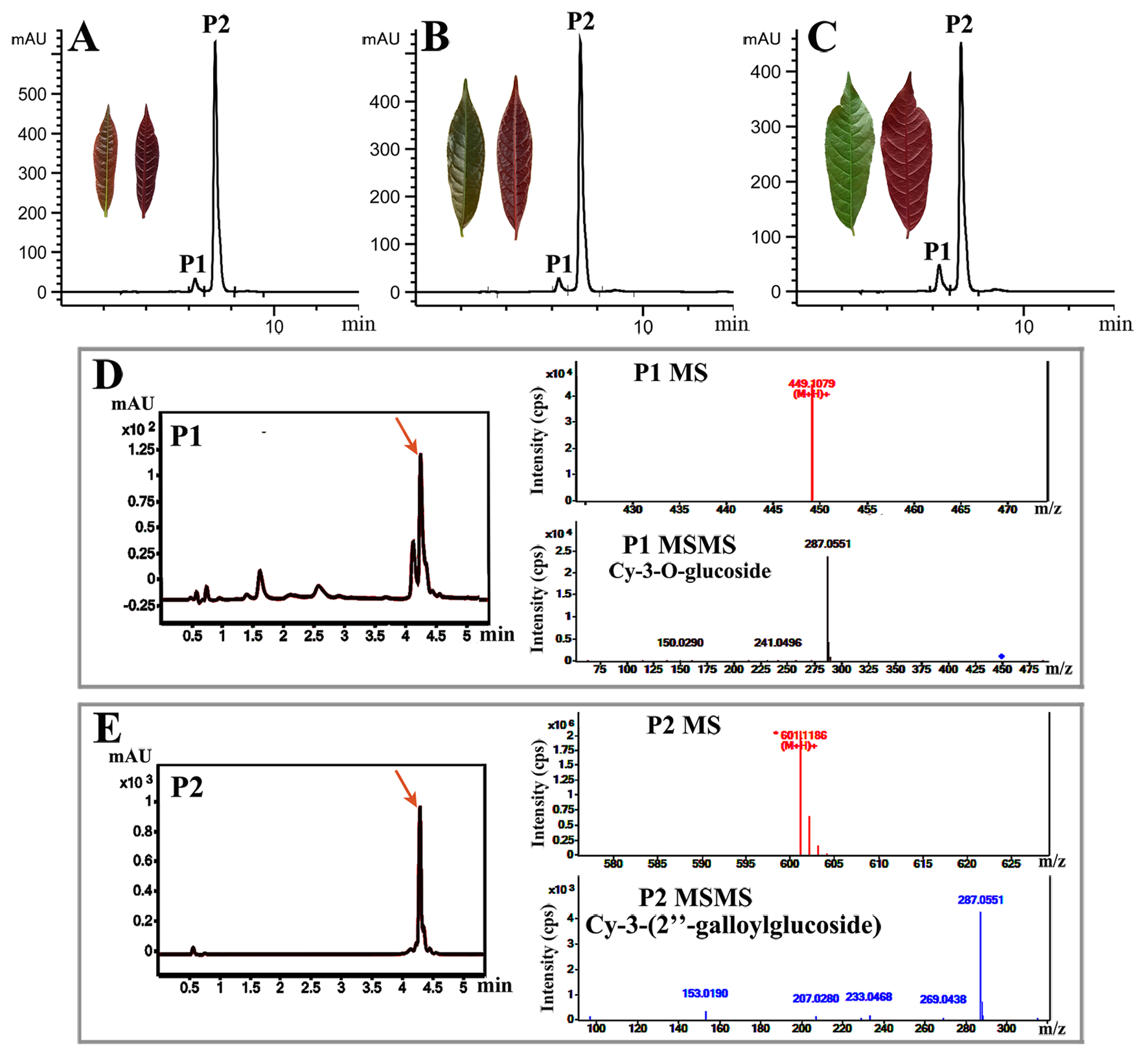

Supplement: Supplementary file 1 — Figure S1. HPLC profiles of the anthocyanins in Excoecaria leaves and identification by UPLC-DAD-QTOF-MS/MS. (A-C) Anthocyanin contents in Excoecaria leaves during maturation. Anthocyanin contents in Excoecaria leaves from stage 1 to 3 (as indicated in Fig. 1A) were analyzed by HPLC (A510nm). (D) Peak 1 (P1) identified by UPLC-DAD-QTOF-MS/MS. P1 as indicated in (A-C) was further identified to be Cy-3-O glucoside. (E) Peak 2 (P2) identified by UPLC-DAD-QTOF-MS/MS. P2 as indicated in (A-C) was further identified to be cyanidin 3-(2″-galloylglucoside). (TIF 10184 kb) [file 12870_2019_1903_MOESM1_ESM.tif]

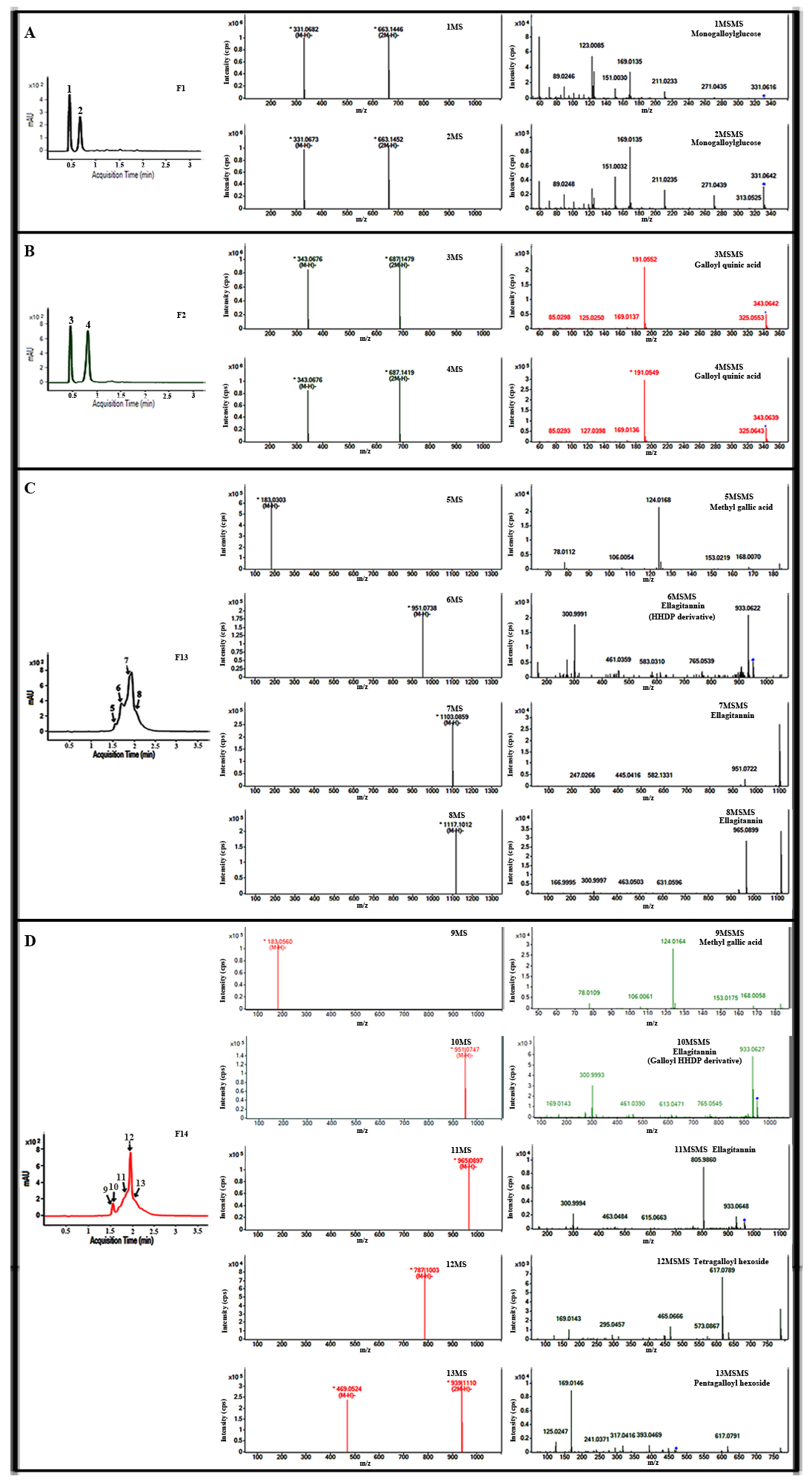

Supplement: Supplementary file 2 — Figure S2. Identification of the Major POD inhibitors from Excoecaria leaves by UPLC-DAD-QTOF-MS/MS. (A-D) Characterization of the compounds in the high inhibition fractions by UPLC-DAD-QTOF-MS/MS. The MS and MS/MS spectra of the major compounds with high absorbance at 280 nm indicate various hydrolysable tannins in the high inhibition fractions of F1 (A), F2 (B), F13(C) and F14 (D) as described in Fig. 5A and B. (TIF 10206 kb) [file 12870_2019_1903_MOESM2_ESM.tif]
