# Supplementary material for: In planta high levels of hydrolysable tannins inhibit peroxidase mediated anthocyanin degradation and maintain abaxially red leaves of Excoecaria Cochinchinensis
Source: BMC Plant Biol. 2019 Jul 15;19:315. doi: 10.1186/s12870-019-1903-y (PMC6632198; doi:10.1186/s12870-019-1903-y)
Supplement: Supplementary file 4 — Data file 1. MSMS compound report of Excoecaria phenolics used in this study. (PDF 21926 kb) [file 12870_2019_1903_MOESM4_ESM.pdf]

Qualitative Compound Report

**Data File**	C ms2.d	**Sample Name**	C
**Sample Type**	Sample	**Position**	P1-A3
**Instrument Name**	Instrument 1	**User Name**	
**Acq Method**	tannic acid automs2.m	**Acquired Time**	4/1/2017 5:57:33 PM
**IRM Calibration Status**	Success	**DA Method**	scau default.m
**Comment**			

**Sample Group**		**Info.**	
**Stream Name**	LC 1	**Acquisition SW Version**	6200 series TOF/6500 series Q-TOF B.06.01 (B6157)

Fragmentor Voltage 175 **Collision Energy** 0 **Ionization Mode** ESI

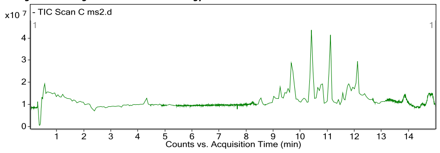

Compound Table

Compound Label	Data File	RT	Mass	Abund	Name	Formula	Tgt Mass	Diff (ppm)	MFG Formula	MFG Diff (ppm)	DB Formula	DB Diff (ppm)	Hits (DB)	Purity Value	Purity Result	Purity Comments	SignificantIonMz
Cpd 1: 0.311		0.311		28603					<none>								965.0905
Cpd 2: C48 H28 O21		0.344	940.11398						C48 H28 O21	-1.77							939.1062
Cpd 3: C20 H20 O14		0.563	484.08511						C20 H20 O14	0.41							483.0778
Cpd 4: C7 H6 O5		0.636	170.02102						C7 H6 O5	2.99							169.0136
Cpd 5: 0.904		0.904							<none>								567.0296
Cpd 6: C7 H6 O5		0.959	170.02094						C7 H6 O5	3.41							169.0138
Cpd 7: C15 H14 O6		1.084	290.0787						C15 H14 O6	1.17							289.0714
Cpd 8: C20 H20 O14		1.093	484.08472						C20 H20 O14	1.21							483.0775
Cpd 9: C28 H24 O15		1.37	600.11148						C28 H24 O15	0.06							599.1042
Cpd 10: C28 H26 O16		1.487	618.12204						C28 H26 O16	0.07							617.1149
Cpd 11: C26 H40 O12		1.504	544.25167						C26 H40 O12	0.56							543.2446
Cpd 12: C20 H18 O13		1.562	466.07408						C20 H18 O13	1.42							465.0671
Cpd 13: C27 H24 O18		1.592	636.09586						C27 H24 O18	0.64							635.0886
Cpd 14: C34 H26 O22		1.621	786.0911						C34 H26 O22	0.61							785.0838
Cpd 15: C20 H30 O12		1.789	462.17346						C20 H30 O12	0.58							461.1662
Cpd 16: C16 H20 O10		1.877	372.10511						C16 H20 O10	1.45							371.0978
Cpd 17: C42 H30 O27		1.881	966.09849						C42 H30 O27	-1.08							965.0898
Cpd 18: C34 H28 O22		2.053	788.10683						C34 H28 O22	0.5							787.0996
Cpd 19: 2.208		2.208		31757					<none>								965.089
Cpd 20: C29 H42 O15		2.234	630.25215						C29 H42 O15	0.35							629.2446
Cpd 21: C28 H42 O13		2.25	586.26198						C28 H42 O13	0.96							585.2548
Cpd 22: C21 H32 O12		2.346	476.1883						C21 H32 O12	2.26							475.1811
Cpd 23: C28 H24 O16		2.464	616.10519						C28 H24 O16	2.03							615.0978
Cpd 24: C41 H32 O26		2.472	940.11788						C41 H32 O26	0.32							939.1108
Cpd 25: C22 H24 O13		2.514	496.12104						C22 H24 O13	1.31							495.1138
Cpd 26: C24 H50 O10		2.661	498.34118						C24 H50 O10	-1.56							497.334
Cpd 27: C14 H6 O8		2.762	302.00588						C14 H6 O8	1.28							300.9986
Cpd 28: C23 H24 O13		2.778	508.12083	23842					C23 H24 O13	1.69							507.1139
Cpd 29: C28 H24 O16		2.954	616.10583						C28 H24 O16	0.98							615.0981
Cpd 30: C28 H24 O15		2.971	600.11084						C28 H24 O15	1.13							599.1036
Cpd 31: C42 H32 O27		3.24	968.11308	48382					C42 H32 O27	0.01							967.1055
Cpd 32: C21 H20 O11		3.353	448.10049						C21 H20 O11	0.16							447.0933
Cpd 33: C28 H24 O15		3.52	600.11063						C28 H24 O15	1.48							599.1032
Cpd 34: C20 H18 O10		3.609	418.08959						C20 H18 O10	0.97							417.0824
Cpd 35: C28 H32 O17		3.77	640.16355						C28 H32 O17	0.63							639.1564
Cpd 36: C24 H26 O12		3.817	506.14181						C24 H26 O12	1.22							505.1346
Cpd 37: C27 H22 O14		3.968	570.10093	21345					C27 H22 O14	0.05							569.0939
Cpd 38: C22 H22 O11		4.014	462.11627						C22 H22 O11	-0.13							461.109
Cpd 39: C41 H72 O10		4.088	724.51005						C41 H72 O10	3.46							723.5027
Cpd 40: C44 H70 O16		4.14	854.46788						C44 H70 O16	-1.74							853.4608
Cpd 41: C40 H70 O8		4.228	678.5045						C40 H70 O8	3.78							677.4974
Cpd 42: 4.358		4.358							<none>								395.1387
Cpd 43: C41 H72 O10		4.374	724.51007						C41 H72 O10	3.42							723.5027
Cpd 44: 4.417		4.417							<none>								431.1337
Cpd 45: C27 H30 O16		4.433	610.15299						C27 H30 O16	0.65							609.1458
Cpd 46: C22 H22 O12		4.597	478.11082						C22 H22 O12	0.63							477.1035
Cpd 47: C23 H26 O9		4.967	446.15716						C23 H26 O9	1.17							445.1499
Cpd 48: C65 H90 O5		5.206	950.67797						C65 H90 O5	0.9							949.6708
Cpd 49: 8.524		8.524							<none>								383.1899
Cpd 50: 8.532		8.532							<none>								525.0637
Cpd 51: 8.551		8.551							<none>								265.1478
Cpd 52: 8.896		8.896							<none>								851.3253
Cpd 53: 8.918		8.918							<none>								365.1636
Cpd 54: C34 H58 O16		8.978	722.37303						C34 H58 O16	-0.75							721.3658
Cpd 55: C33 H56 O14		9.014	676.36643						C33 H56 O14	0.85							675.3594
Cpd 56: 9.069		9.069							<none>								309.1738
Cpd 57: C23 H46 O16		9.19	578.27621						C23 H46 O16	4.1							577.2693
Cpd 58: 9.304		9.304							<none>								353.2001
Cpd 59: C34 H58 O16		9.323	722.37318						C34 H58 O16	-0.96							721.3658
Cpd 60: 9.387		9.387		62304					<none>								566.3554
Cpd 61: 9.496		9.496							<none>								397.2262
Cpd 62: C28 H48 O11		9.542	560.32024						C28 H48 O11	-1.03							559.313
Cpd 63: 9.601		9.601							<none>								441.2529
Cpd 64: 9.672		9.672							<none>								485.2791
Cpd 65: 9.760		9.76		624582					<none>								293.179
Cpd 66: 9.777		9.777							<none>								529.3054
Cpd 67: C14 H30 O8		9.781	326.19142						C14 H30 O8	8.13							325.1843
Cpd 68: C15 H24 O		9.79	220.18253						C15 H24 O	0.85							219.1752
Cpd 69: 9.836		9.836							<none>								297.2433
Cpd 70: 9.928		9.928							<none>								404.3013
Cpd 71: 9.953		9.953							<none>								130.9436
Cpd 72: 9.966		9.966							<none>								358.2961
Cpd 73: C27 H41 O7		10.062	477.28506						C27 H41 O7	0.35							476.2779
Cpd 74: C32 H60 O16		10.07	700.38784						C32 H60 O16	0.42							699.381
Cpd 75: C26 H49 O10		10.125	521.33536						C26 H49 O10	-5.35							520.3281
Cpd 76: 10.217		10.217							<none>								652.2801
Cpd 77: C29 H49 O9		10.23	541.33786						C29 H49 O9	-0.38							540.3307
Cpd 78: C17 H34 O6		10.368	334.2355						C17 H34 O6	0.1							333.2288
Cpd 79: C19 H32 O5		10.427	340.22502	1137828					C19 H32 O5	-0.14							339.2177
Cpd 80: C11 H16 O		10.456	164.11994						C11 H16 O	1.05							163.1127
Cpd 81: C29 H50 O16		10.486	654.30914						C29 H50 O16	1.15							653.302
Cpd 82: C18 H30 O2		10.515	278.22449						C18 H30 O2	0.33							277.2171
Cpd 83: C36 H38 O		10.578	486.28992						C36 H38 O	4.82							485.2825
Cpd 84: C16 H30 O2		10.662	254.22522						C16 H30 O2	-2.53							253.2174
Cpd 85: C19 H32 O3		10.666	308.23573						C19 H32 O3	-1.9							307.2285
Cpd 86: C41 H70 O14		10.699	786.47682						C41 H70 O14	-0.33							785.4698
Cpd 87: C32 H48 O9		10.725	576.33076						C32 H48 O9	-1.6							575.3233
Cpd 88: C18 H36 O3		10.742	300.26709						C18 H36 O3	-2.15							299.2599
Cpd 89: C34 H48 O5		10.787	536.35063						C34 H48 O5	-0.85							535.3434
Cpd 90: C37 H50 O11		10.859	670.33639						C37 H50 O11	-1.61							669.3288
Cpd 91: C18 H32 O2		10.859	280.24045						C18 H32 O2	-0.78							279.2332
Cpd 92: C54 H80 O11		10.876	904.5703						C54 H80 O11	-0.26							903.5639
Cpd 93: C35 H52 O8		10.939	600.36629						C35 H52 O8	-0.12							599.3591
Cpd 94: C37 H54 O10		11.027	658.37243						C37 H54 O10	-1.11							657.3653
Cpd 95: C36 H52 O8		11.052	612.36656						C36 H52 O8	-0.56							611.3594
Cpd 96: C16 H32 O2		11.107	256.24069						C16 H32 O2	-1.81							255.2336
Cpd 97: C50 H82 O7		11.152	794.60708	210737					C50 H82 O7	-1.28							793.5997
Cpd 98: C34 H50 O5		11.169	538.3665						C34 H50 O5	-1.25							537.3592
Cpd 99: C31 H40 O3		11.224	460.2977						C31 H40 O3	0.11							459.2904
Cpd 100: C35 H48 O3		11.307	516.36044						C35 H48 O3	-0.19							515.3531
Cpd 101: C18 H34 O2		11.417	282.25641						C18 H34 O2	-1.89							281.2489
Cpd 102: C22 H44 O6		11.459	404.31403						C22 H44 O6	-0.6							403.3067
Cpd 103: C29 H44 O2		11.61	424.33398						C29 H44 O2	0.37							423.3265
Cpd 104: C37 H50 O10		11.635	654.34138						C37 H50 O10	-1.5							653.3339
Cpd 105: C45 H68 O7		11.922	720.49444						C45 H68 O7	2.86							719.487
Cpd 106: C34 H46 O3		11.94	502.34491						C34 H46 O3	-0.44							501.3375
Cpd 107: C37 H52 O10		12.019	656.35639						C37 H52 O10	-0.53							655.349
Cpd 108: 12.119		12.119							<none>								551.3078

Cpd 109: C18 H36 O2		12.162	284.2714						C18 H36 O2	0.46							283.264
Cpd 110: C38 H68 O8		12.17	652.4941						C38 H68 O8	-4.11							651.4871
Cpd 111: 12.170		12.17							<none>								413.2213
Cpd 112: C45 H68 O7		12.38	720.49453						C45 H68 O7	2.75							719.4873
Cpd 113: 12.433		12.433							<none>								551.3079
Cpd 114: C49 H77 O10		12.473	825.55411						C49 H77 O10	-2.95							824.5455
Cpd 115: C23 H32 O3		12.59	356.23492						C23 H32 O3	0.64							355.2275
Cpd 116: 12.674		12.674							<none>								892.5337
Cpd 117: 12.964		12.964							<none>								463.3101
Cpd 118: 12.990		12.99							<none>								913.5835
Cpd 119: C56 H84 O10		13.125	916.60611						C56 H84 O10	0.37							915.5989

Compound Label	Name	*m/z*	RT	Algorithm	Mass
Cpd 1: 0.311		965.0905	0.311	Auto MS/MS	

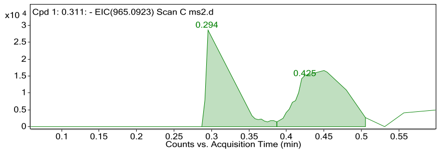

MS Spectrum

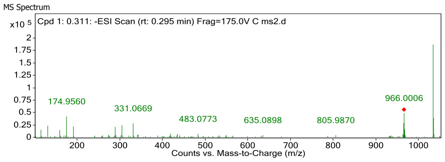

MS Zoomed Spectrum

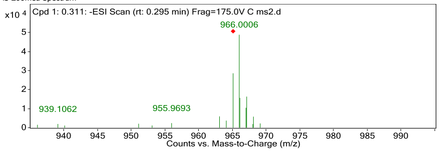

MSMS Spectrum

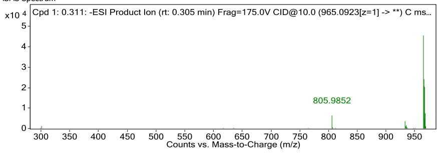

MSMS Spectrum

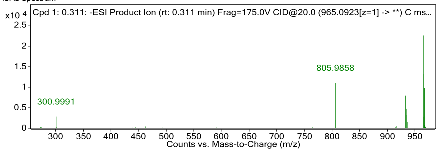

MSMS Spectrum

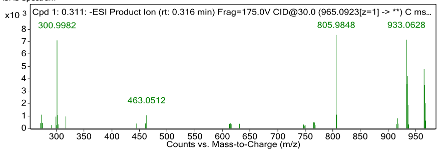

Compound Label	Name	*m/z*	RT	Algorithm	Mass
Cpd 2: C48 H28 O21		939.1062	0.344	Auto MS/MS	940.11398

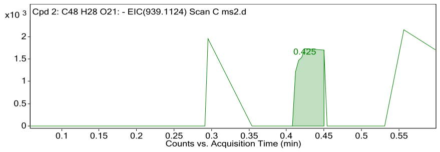

MS Spectrum

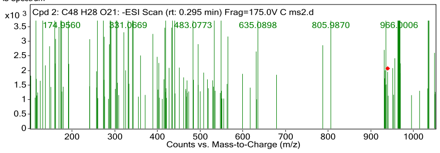

MS Zoomed Spectrum

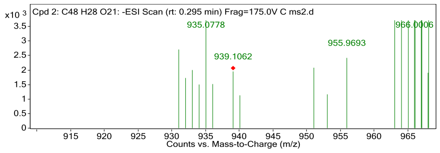

MSMS Spectrum

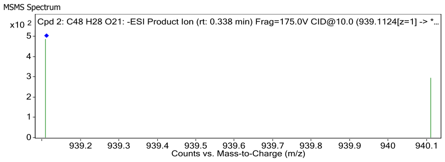

MSMS Spectrum

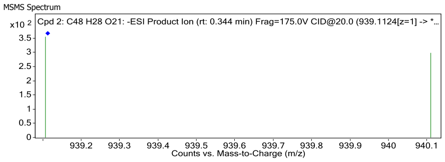

MSMS Spectrum

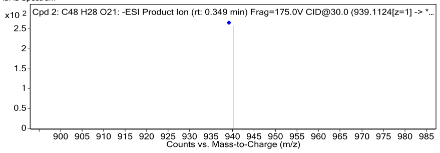

Compound Label	Name	*m/z*	RT	Algorithm	Mass
Cpd 3: C20 H20 O14		483.0778	0.563	Auto MS/MS	484.08511

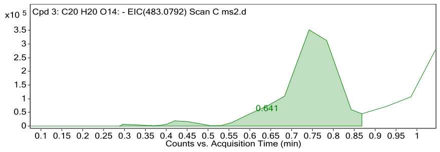

MS Spectrum

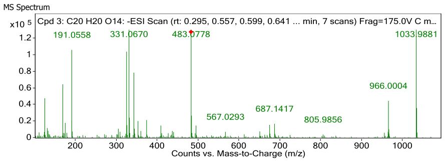

MS Zoomed Spectrum

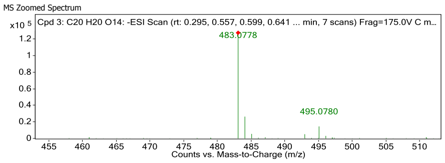

MSMS Spectrum

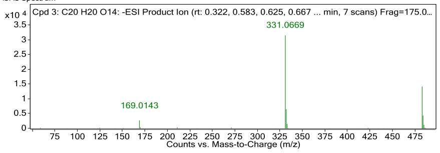

MSMS Spectrum

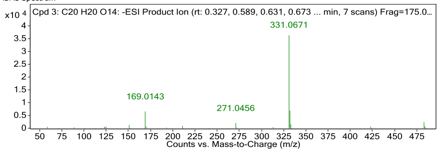

MSMS Spectrum

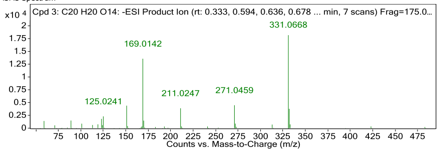

Compound Label	Name	*m/z*	RT	Algorithm	Mass
Cpd 4: C7 H6 O5		169.0136	0.636	Auto MS/MS	170.02102

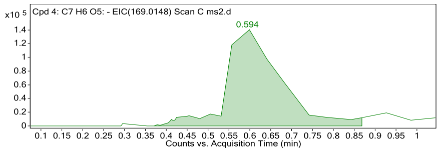

MS Spectrum

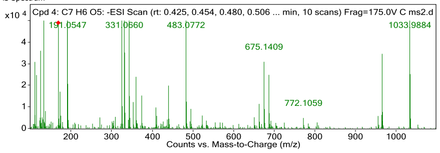

MS Zoomed Spectrum

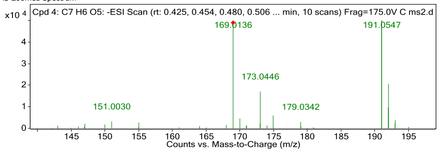

MSMS Spectrum

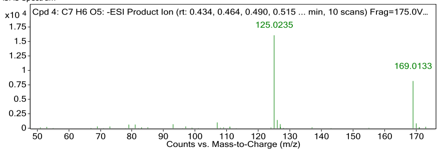

MSMS Spectrum

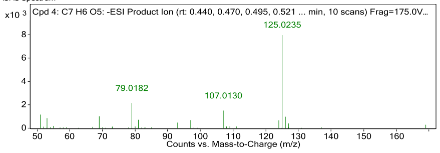

MSMS Spectrum

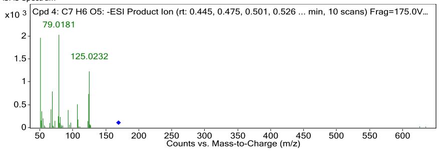

Compound Label	Name	*m/z*	RT	Algorithm	Mass
Cpd 5: 0.904		567.0296	0.904	Auto MS/MS	

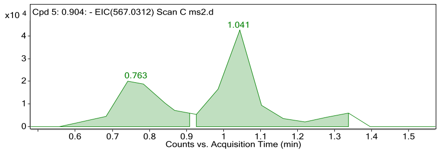

MS Spectrum

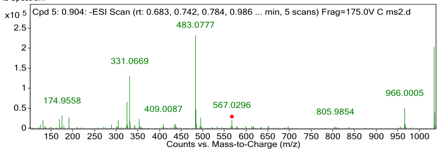

MS Zoomed Spectrum

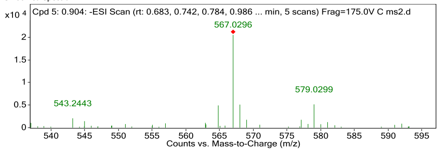

MSMS Spectrum

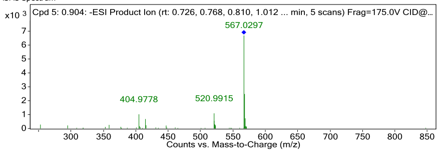

MSMS Spectrum

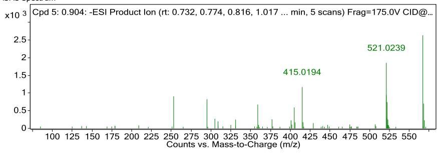

MSMS Spectrum

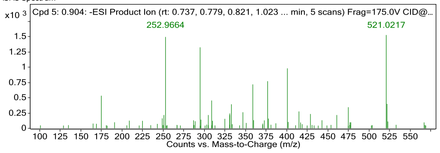

Compound Label	Name	*m/z*	RT	Algorithm	Mass
Cpd 6: C7 H6 O5		169.0138	0.959	Auto MS/MS	170.02094

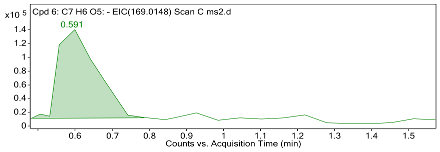

MS Spectrum

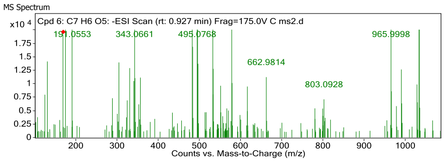

MS Zoomed Spectrum

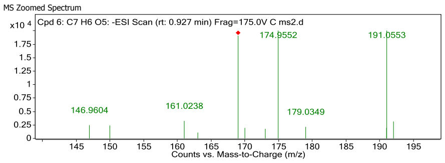

MSMS Spectrum

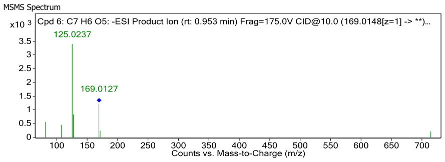

MSMS Spectrum

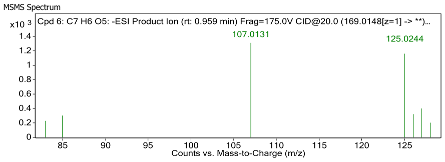

MSMS Spectrum

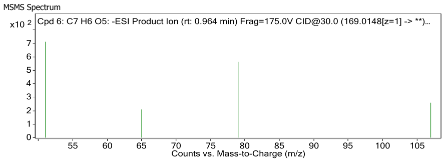

Compound Label	Name	*m/z*	RT	Algorithm	Mass
Cpd 7: C15 H14 O6		289.0714	1.084	Auto MS/MS	290.0787

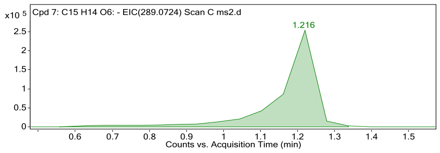

MS Spectrum

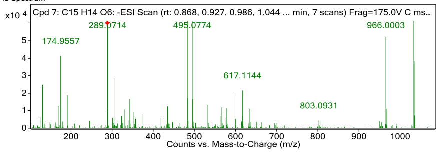

MS Zoomed Spectrum

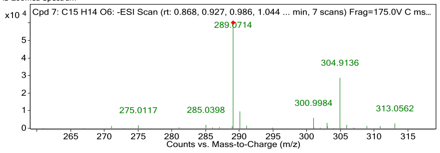

MSMS Spectrum

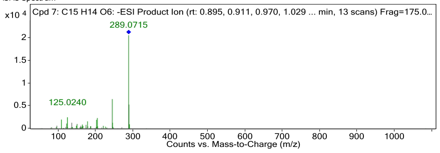

MSMS Spectrum

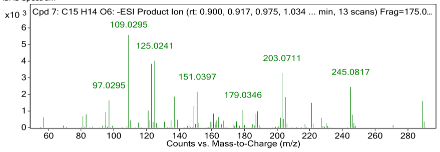

MSMS Spectrum

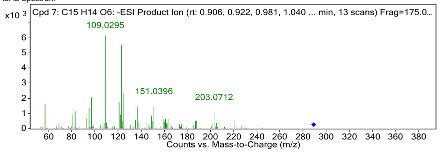

Compound Label	Name	*m/z*	RT	Algorithm	Mass
Cpd 8: C20 H20 O14		483.0775	1.093	Auto MS/MS	484.08472

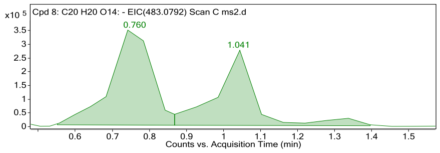

MS Spectrum

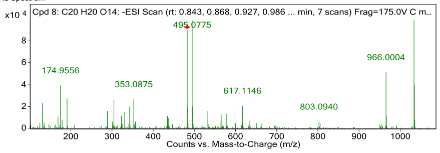

MS Zoomed Spectrum

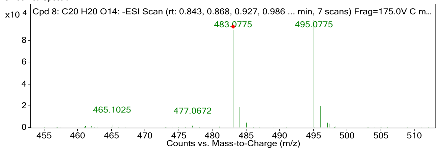

MSMS Spectrum

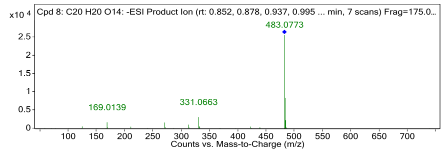

MSMS Spectrum

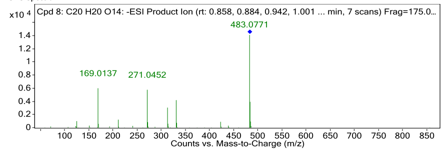

MSMS Spectrum

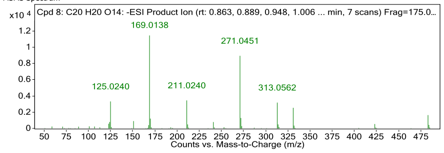

Compound Label	Name	*m/z*	RT	Algorithm	Mass
Cpd 9: C28 H24 O15		599.1042	1.37	Auto MS/MS	600.11148

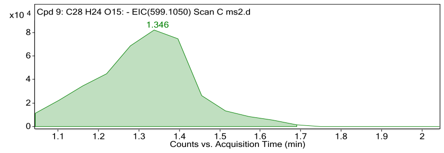

MS Spectrum

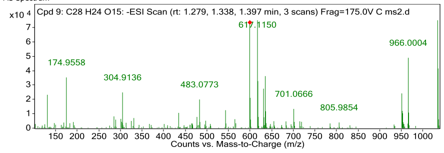

MS Zoomed Spectrum

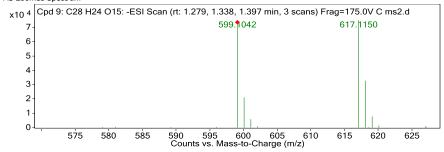

MSMS Spectrum

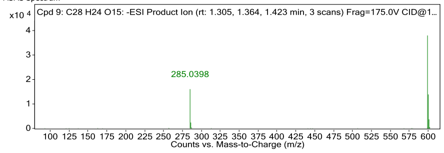

MSMS Spectrum

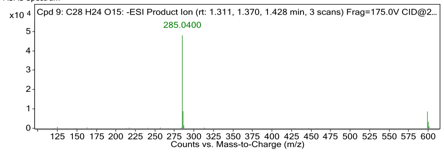

MSMS Spectrum

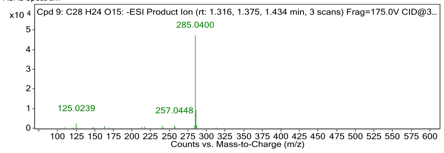

Compound Label	Name	*m/z*	RT	Algorithm	Mass
Cpd 10: C28 H26 O16		617.1149	1.487	Auto MS/MS	618.12204

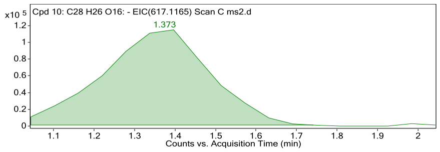

MS Spectrum

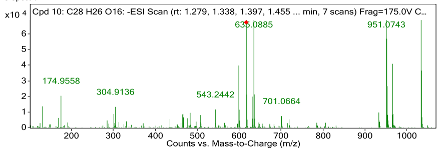

MS Zoomed Spectrum

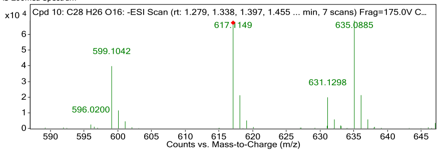

MSMS Spectrum

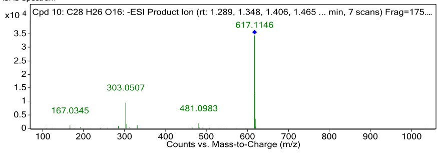

MSMS Spectrum

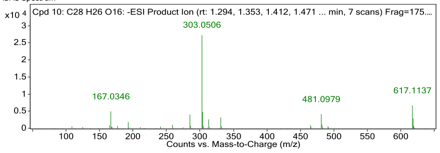

MSMS Spectrum

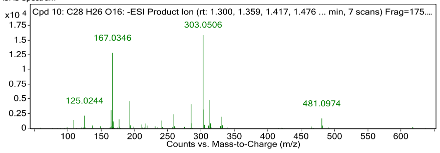

Compound Label	Name	*m/z*	RT	Algorithm	Mass
Cpd 11: C26 H40 O12		543.2446	1.504	Auto MS/MS	544.25167

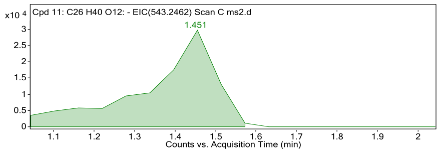

MS Spectrum

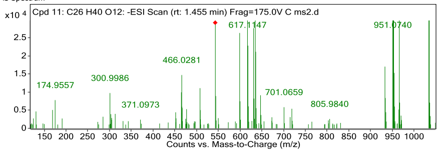

MS Zoomed Spectrum

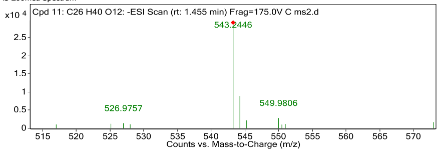

MSMS Spectrum

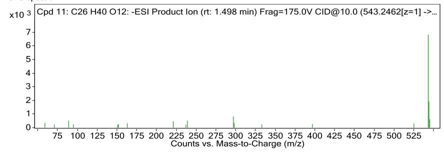

MSMS Spectrum

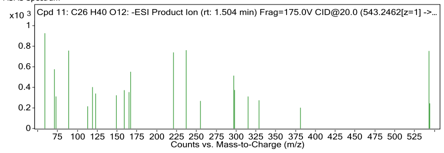

MSMS Spectrum

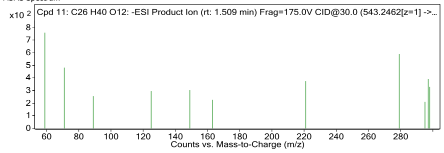

Compound Label	Name	*m/z*	RT	Algorithm	Mass
Cpd 12: C20 H18 O13		465.0671	1.562	Auto MS/MS	466.07408

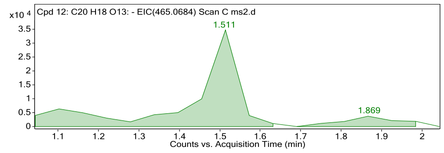

MS Spectrum

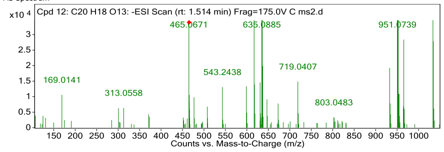

MS Zoomed Spectrum

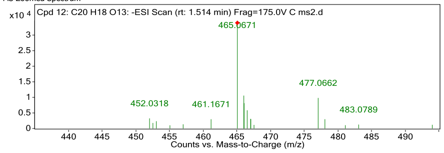

MSMS Spectrum

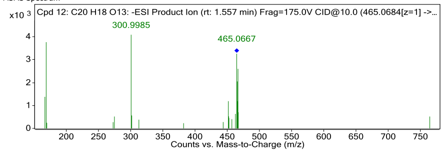

MSMS Spectrum

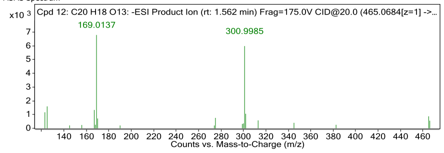

MSMS Spectrum

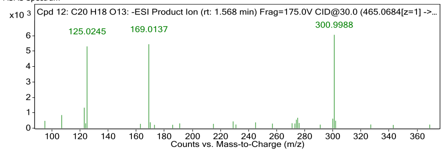

Compound Label	Name	*m/z*	RT	Algorithm	Mass
Cpd 13: C27 H24 O18		635.0886	1.592	Auto MS/MS	636.09586

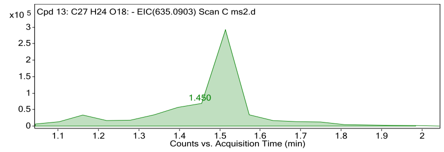

MS Spectrum

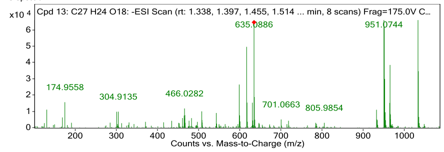

MS Zoomed Spectrum

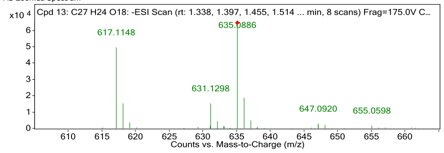

MSMS Spectrum

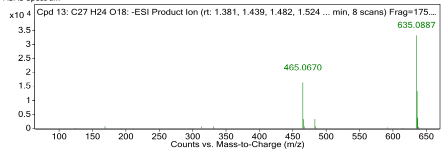

MSMS Spectrum

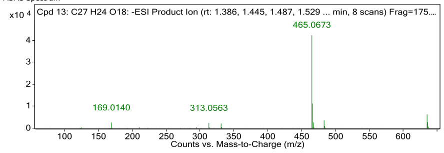

MSMS Spectrum

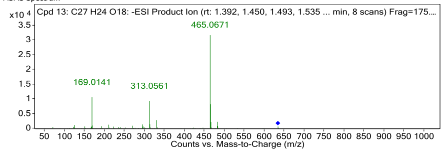

Compound Label	Name	*m/z*	RT	Algorithm	Mass
Cpd 14: C34 H26 O22		785.0838	1.621	Auto MS/MS	786.0911

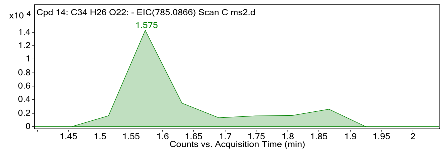

MS Spectrum

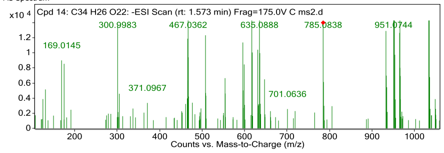

MS Zoomed Spectrum

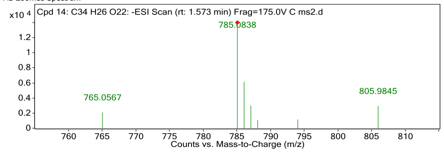

MSMS Spectrum

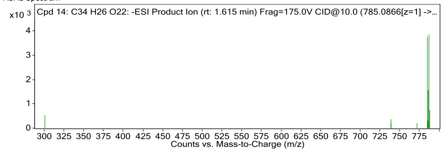

MSMS Spectrum

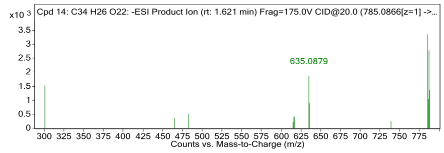

MSMS Spectrum

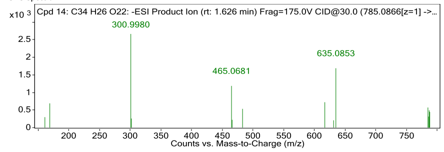

Compound Label	Name	*m/z*	RT	Algorithm	Mass
Cpd 15: C20 H30 O12		461.1662	1.789	Auto MS/MS	462.17346

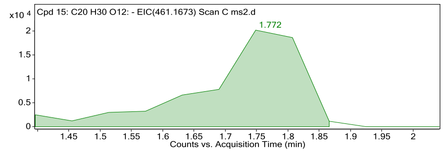

MS Spectrum

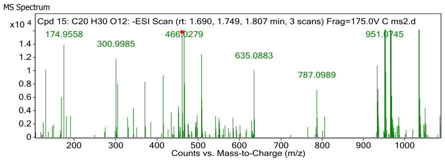

MS Zoomed Spectrum

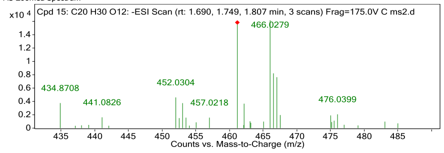

MSMS Spectrum

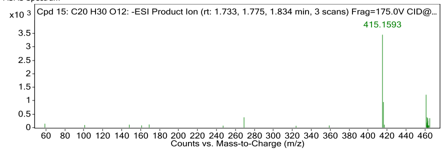

MSMS Spectrum

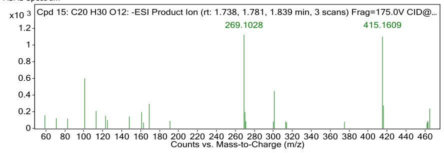

MSMS Spectrum

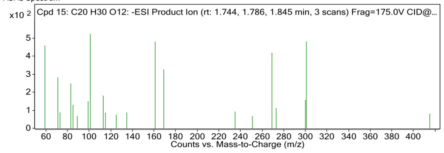

Compound Label	Name	*m/z*	RT	Algorithm	Mass
Cpd 16: C16 H20 O10		371.0978	1.877	Auto MS/MS	372.10511

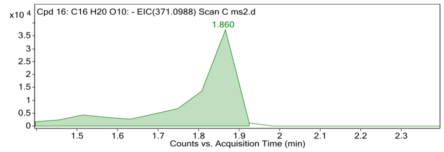

MS Spectrum

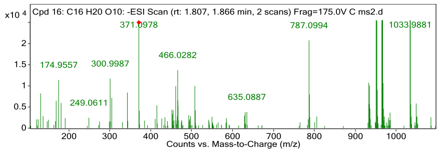

MS Zoomed Spectrum

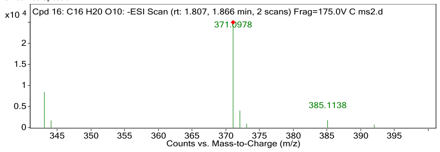

MSMS Spectrum

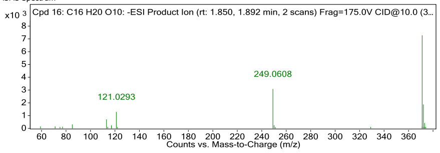

MSMS Spectrum

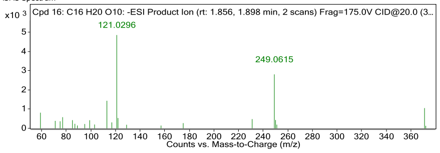

MSMS Spectrum

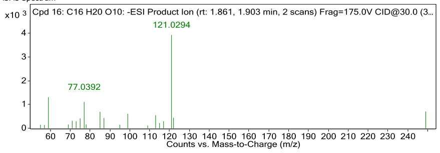

Compound Label	Name	*m/z*	RT	Algorithm	Mass
Cpd 17: C42 H30 O27		965.0898	1.881	Auto MS/MS	966.09849

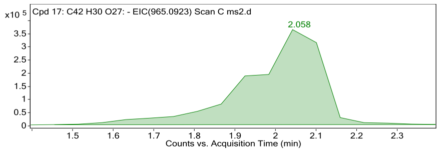

MS Spectrum

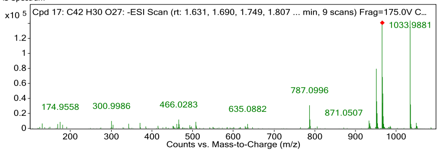

MS Zoomed Spectrum

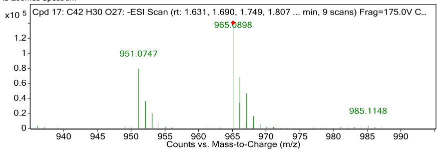

MSMS Spectrum

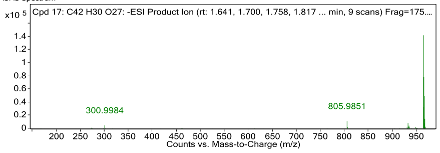

MSMS Spectrum

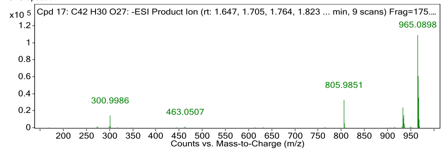

MSMS Spectrum

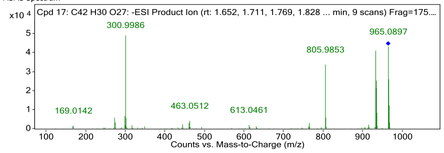

Compound Label	Name	*m/z*	RT	Algorithm	Mass
Cpd 18: C34 H28 O22		787.0996	2.053	Auto MS/MS	788.10683

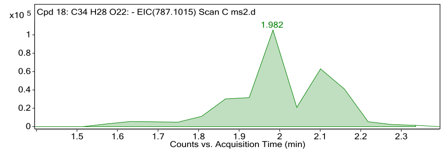

MS Spectrum

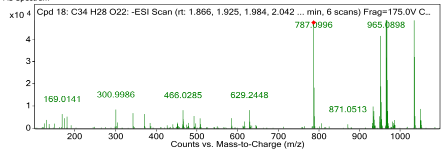

MS Zoomed Spectrum

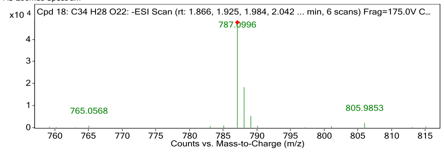

MSMS Spectrum

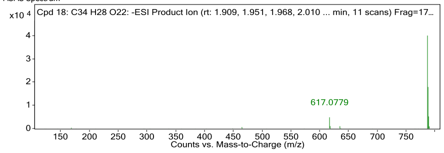

MSMS Spectrum

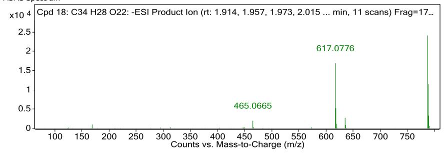

MSMS Spectrum

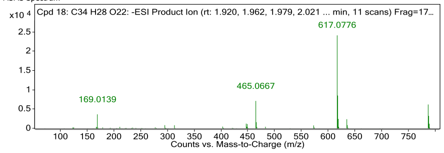

Compound Label	Name	*m/z*	RT	Algorithm	Mass
Cpd 19: 2.208		965.089	2.208	Auto MS/MS	

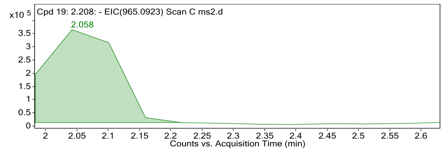

MS Spectrum

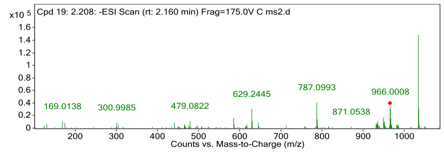

MS Zoomed Spectrum

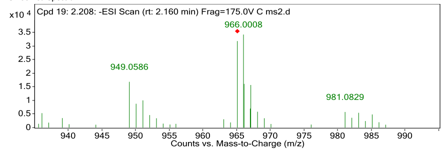

MSMS Spectrum

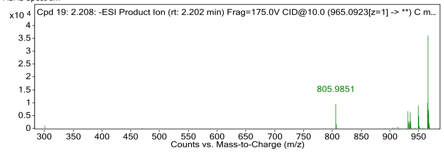

MSMS Spectrum

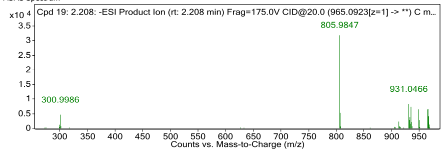

MSMS Spectrum

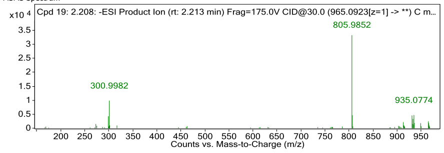

Compound Label	Name	*m/z*	RT	Algorithm	Mass
Cpd 20: C29 H42 O15		629.2446	2.234	Auto MS/MS	630.25215

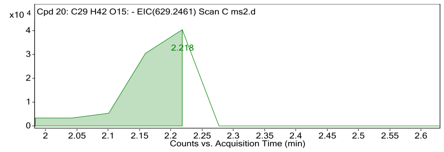

MS Spectrum

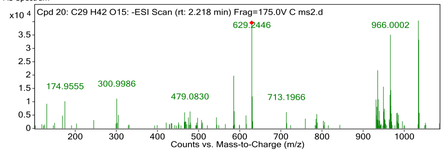

MS Zoomed Spectrum

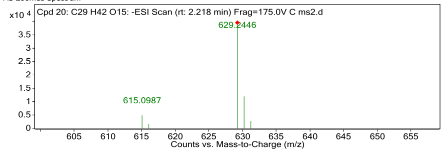

MSMS Spectrum

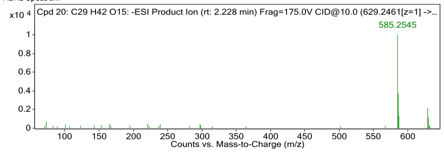

MSMS Spectrum

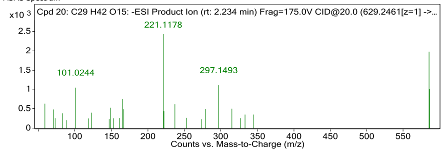

MSMS Spectrum

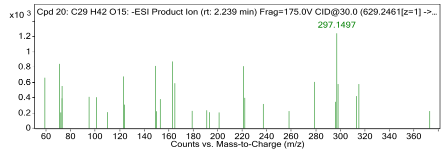

Compound Label	Name	*m/z*	RT	Algorithm	Mass
Cpd 21: C28 H42 O13		585.2548	2.25	Auto MS/MS	586.26198

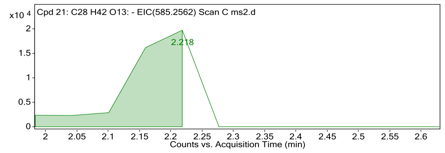

MS Spectrum

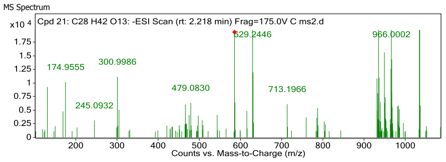

MS Zoomed Spectrum

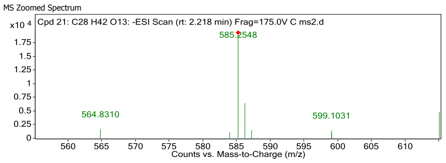

MSMS Spectrum

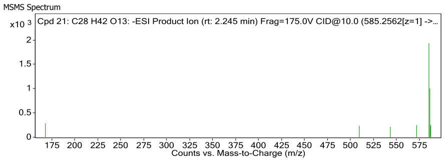

MSMS Spectrum

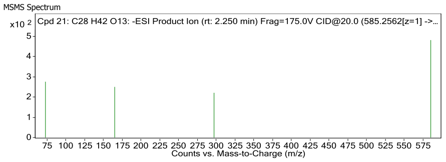

MSMS Spectrum

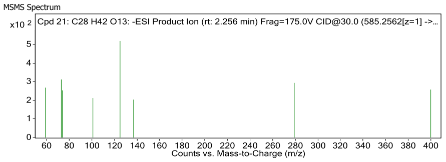

Compound Label	Name	*m/z*	RT	Algorithm	Mass
Cpd 22: C21 H32 O12		475.1811	2.346	Auto MS/MS	476.1883

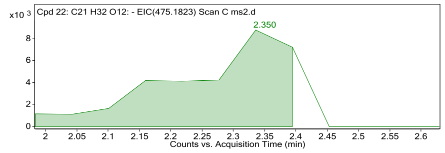

MS Spectrum

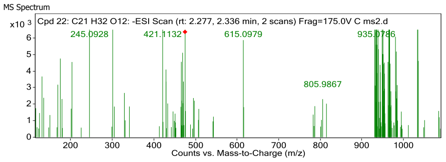

MS Zoomed Spectrum

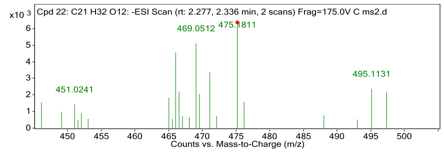

MSMS Spectrum

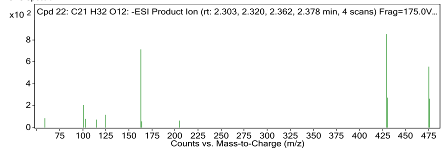

MSMS Spectrum

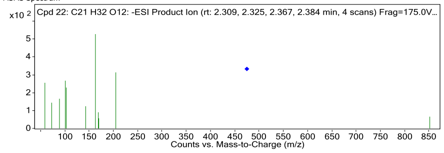

MSMS Spectrum

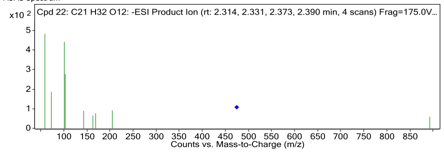

Compound Label	Name	*m/z*	RT	Algorithm	Mass
Cpd 23: C28 H24 O16		615.0978	2.464	Auto MS/MS	616.10519

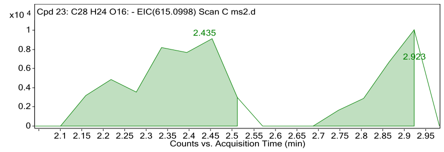

MS Spectrum

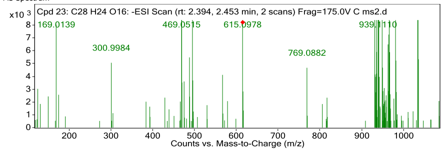

MS Zoomed Spectrum

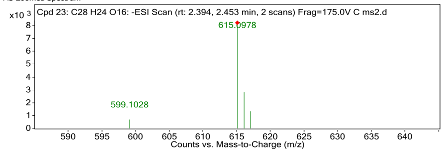

MSMS Spectrum

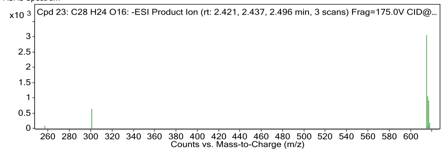

MSMS Spectrum

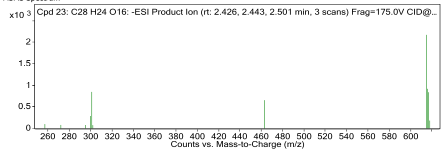

MSMS Spectrum

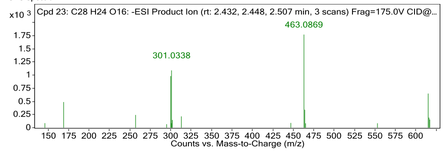

Compound Label	Name	*m/z*	RT	Algorithm	Mass
Cpd 24: C41 H32 O26		939.1108	2.472	Auto MS/MS	940.11788

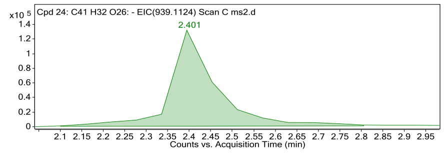

MS Spectrum

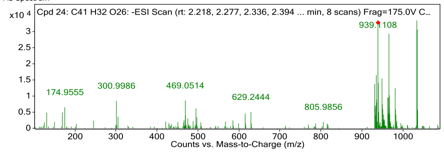

MS Zoomed Spectrum

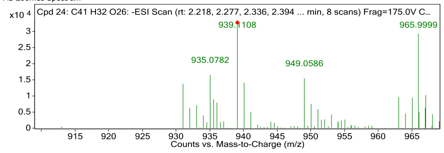

MSMS Spectrum

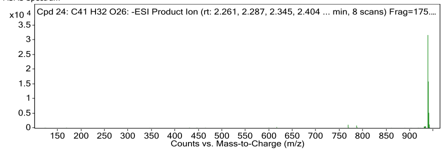

MSMS Spectrum

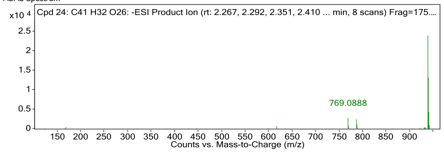

MSMS Spectrum

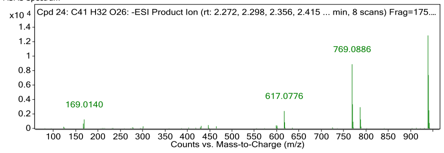

Compound Label	Name	*m/z*	RT	Algorithm	Mass
Cpd 25: C22 H24 O13		495.1138	2.514	Auto MS/MS	496.12104

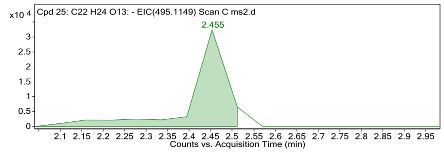

MS Spectrum

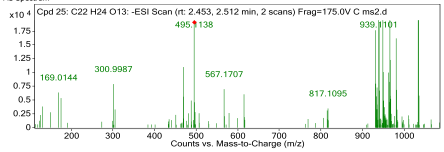

MS Zoomed Spectrum

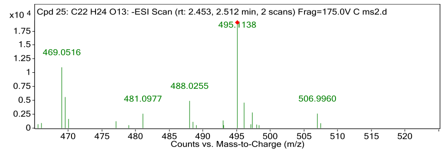

MSMS Spectrum

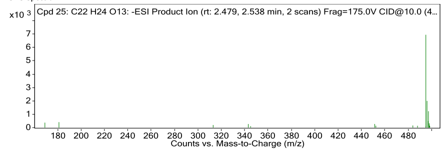

MSMS Spectrum

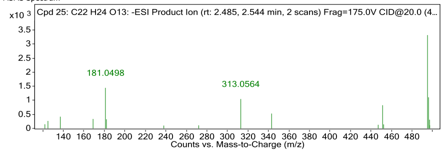

MSMS Spectrum

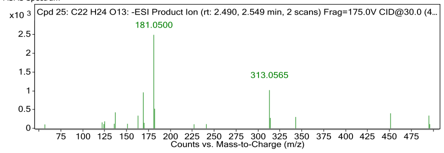

Compound Label	Name	*m/z*	RT	Algorithm	Mass
Cpd 26: C24 H50 O10		497.334	2.661	Auto MS/MS	498.34118

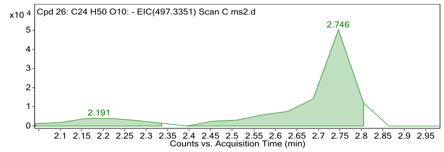

MS Spectrum

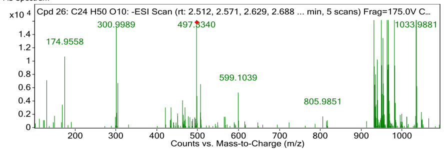

MS Zoomed Spectrum

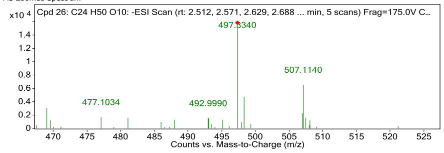

MSMS Spectrum

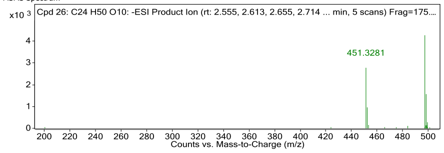

MSMS Spectrum

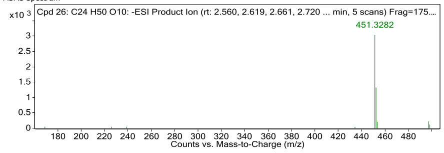

MSMS Spectrum

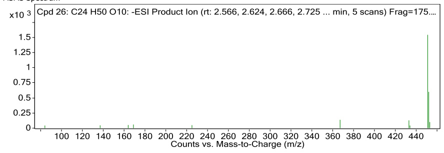

Compound Label	Name	*m/z*	RT	Algorithm	Mass
Cpd 27: C14 H6 O8		300.9986	2.762	Auto MS/MS	302.00588

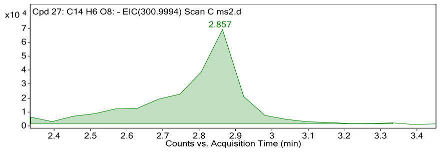

MS Spectrum

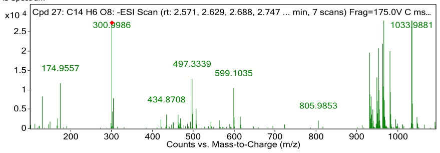

MS Zoomed Spectrum

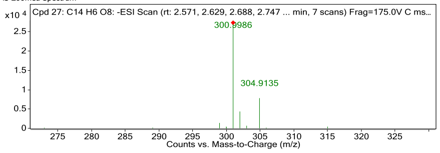

MSMS Spectrum

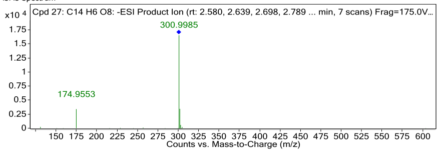

MSMS Spectrum

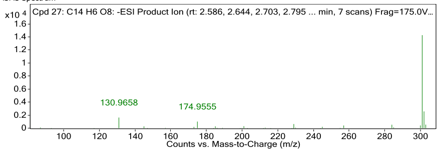

MSMS Spectrum

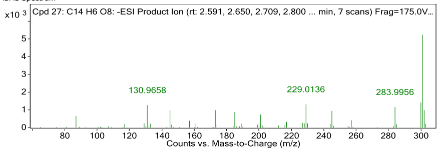

Compound Label	Name	*m/z*	RT	Algorithm	Mass
Cpd 28: C23 H24 O13		507.1139	2.778	Auto MS/MS	508.12083

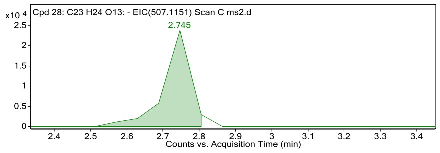

MS Spectrum

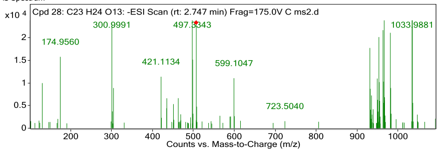

MS Zoomed Spectrum

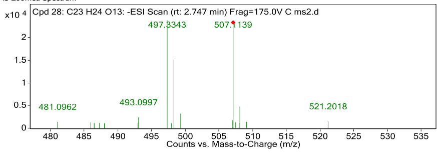

MSMS Spectrum

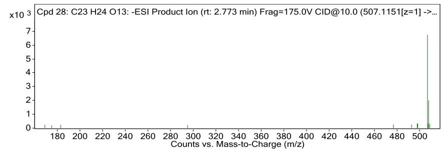

MSMS Spectrum

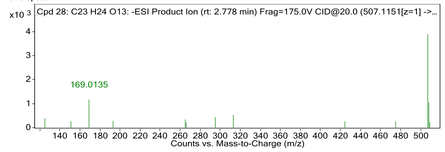

MSMS Spectrum

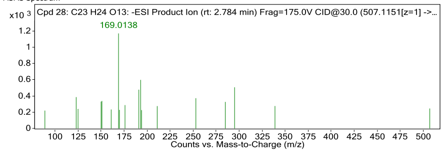

Compound Label	Name	*m/z*	RT	Algorithm	Mass
Cpd 29: C28 H24 O16		615.0981	2.954	Auto MS/MS	616.10583

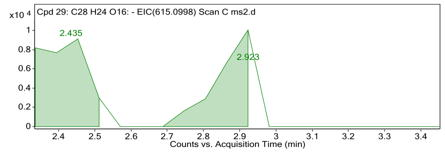

MS Spectrum

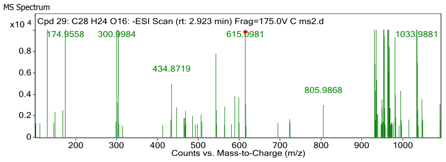

MS Zoomed Spectrum

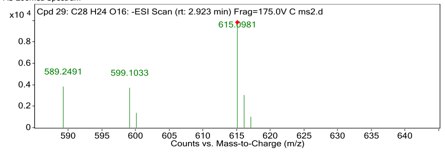

MSMS Spectrum

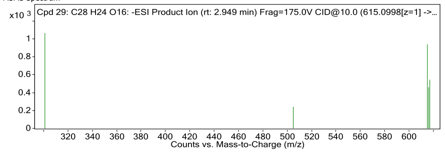

MSMS Spectrum

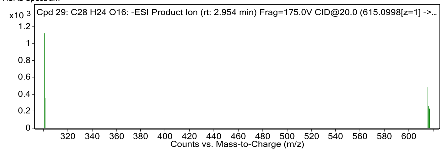

MSMS Spectrum

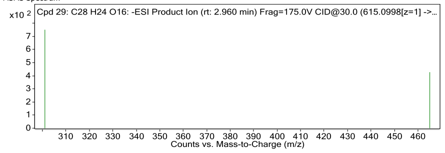

Compound Label	Name	*m/z*	RT	Algorithm	Mass
Cpd 30: C28 H24 O15		599.1036	2.971	Auto MS/MS	600.11084

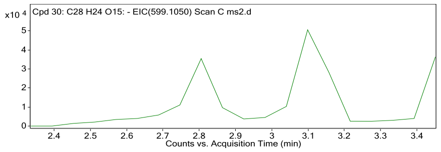

MS Spectrum

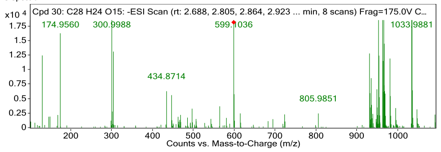

MS Zoomed Spectrum

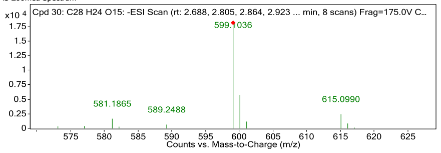

MSMS Spectrum

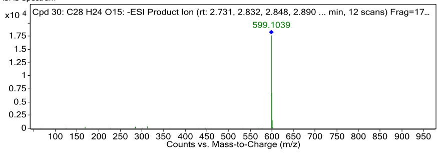

MSMS Spectrum

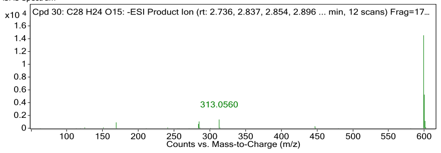

MSMS Spectrum

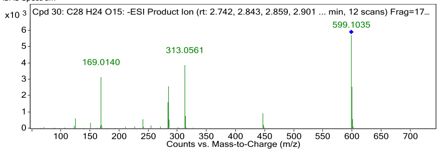

Compound Label	Name	*m/z*	RT	Algorithm	Mass
Cpd 31: C42 H32 O27		967.1055	3.24	Auto MS/MS	968.11308

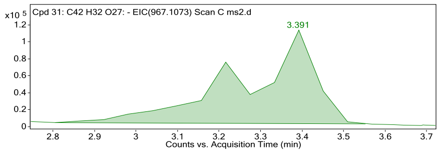

MS Spectrum

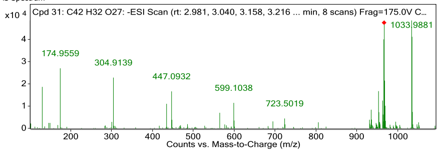

MS Zoomed Spectrum

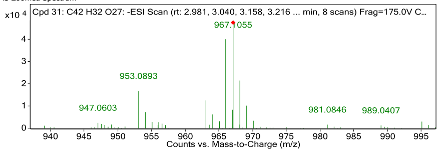

MSMS Spectrum

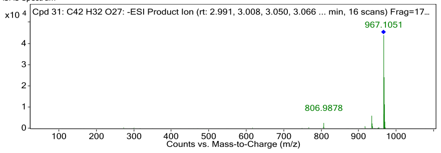

MSMS Spectrum

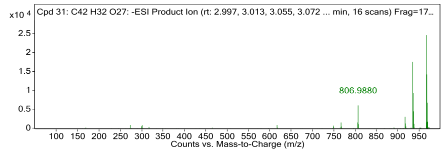

MSMS Spectrum

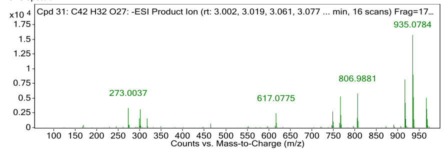

Compound Label	Name	*m/z*	RT	Algorithm	Mass
Cpd 32: C21 H20 O11		447.0933	3.353	Auto MS/MS	448.10049

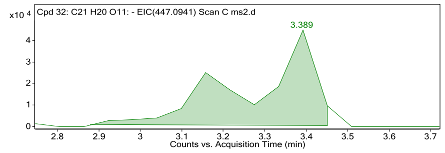

MS Spectrum

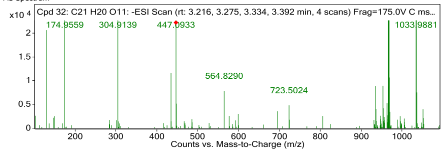

MS Zoomed Spectrum

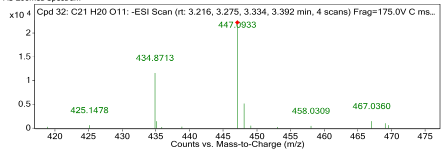

MSMS Spectrum

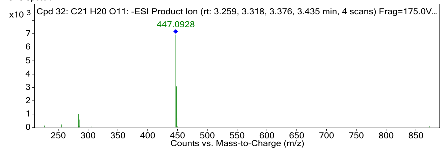

MSMS Spectrum

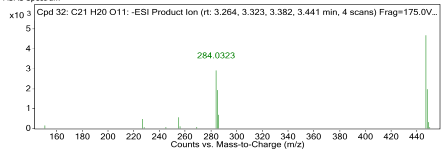

MSMS Spectrum

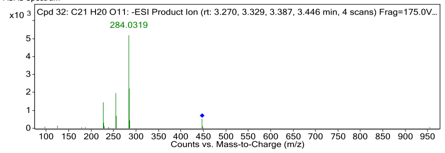

Compound Label	Name	*m/z*	RT	Algorithm	Mass
Cpd 33: C28 H24 O15		599.1032	3.52	Auto MS/MS	600.11063

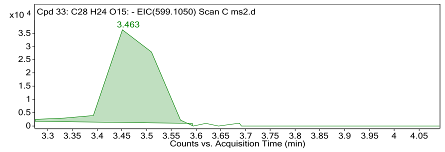

MS Spectrum

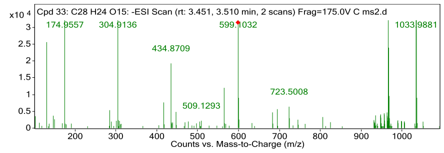

MS Zoomed Spectrum

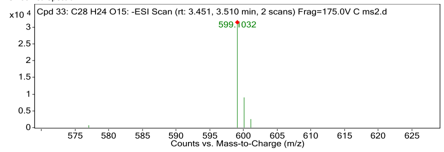

MSMS Spectrum

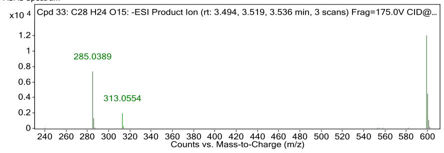

MSMS Spectrum

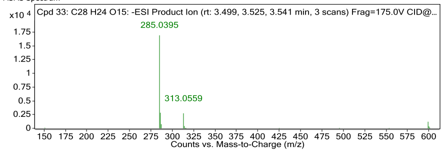

MSMS Spectrum

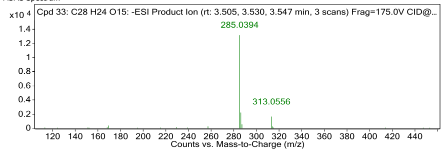

Compound Label	Name	*m/z*	RT	Algorithm	Mass
Cpd 34: C20 H18 O10		417.0824	3.609	Auto MS/MS	418.08959

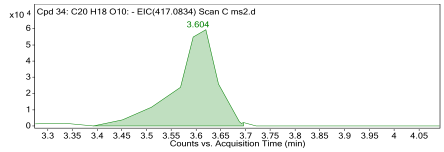

MS Spectrum

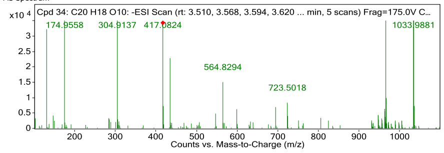

MS Zoomed Spectrum

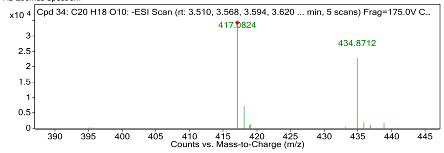

MSMS Spectrum

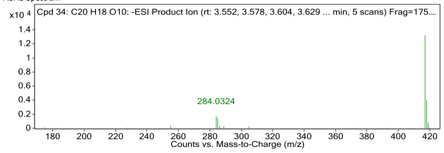

MSMS Spectrum

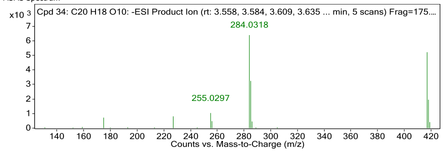

MSMS Spectrum

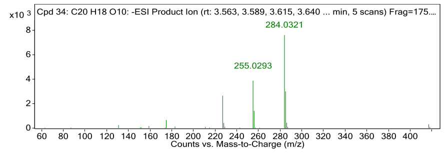

Compound Label	Name	*m/z*	RT	Algorithm	Mass
Cpd 35: C28 H32 O17		639.1564	3.77	Auto MS/MS	640.16355

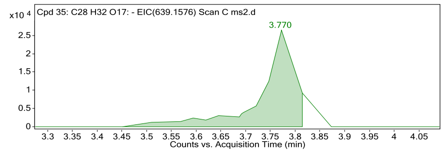

MS Spectrum

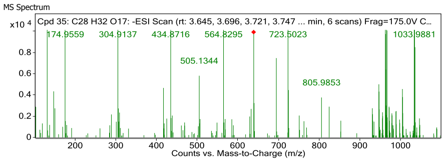

MS Zoomed Spectrum

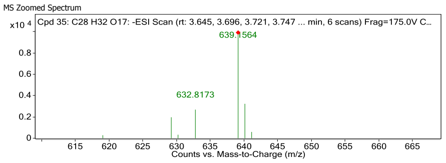

MSMS Spectrum

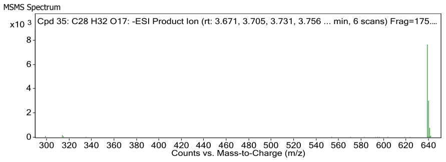

MSMS Spectrum

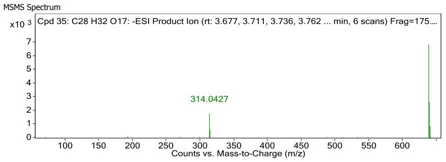

MSMS Spectrum

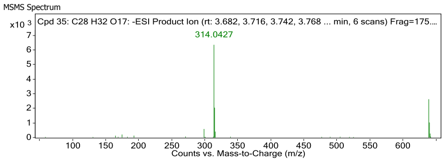

Compound Label	Name	*m/z*	RT	Algorithm	Mass
Cpd 36: C24 H26 O12		505.1346	3.817	Auto MS/MS	506.14181

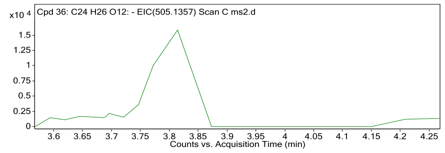

MS Spectrum

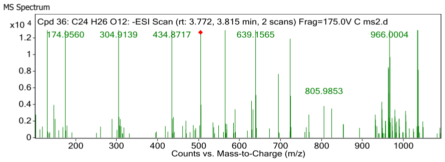

MS Zoomed Spectrum

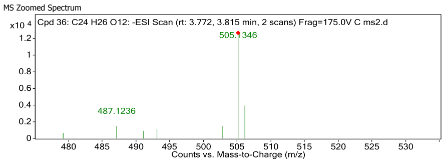

MSMS Spectrum

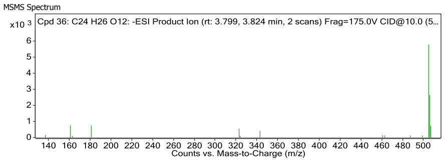

MSMS Spectrum

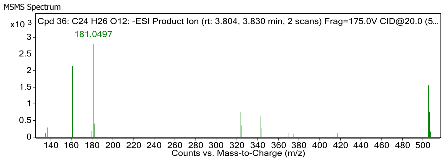

MSMS Spectrum

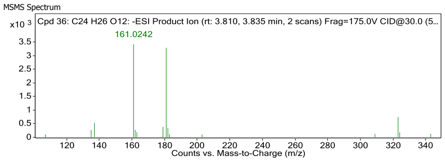

Compound Label	Name	*m/z*	RT	Algorithm	Mass
Cpd 37: C27 H22 O14		569.0939	3.968	Auto MS/MS	570.10093

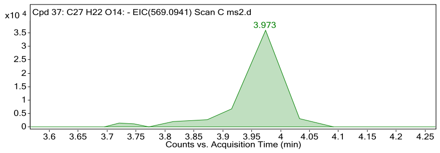

MS Spectrum

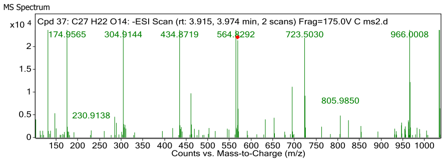

MS Zoomed Spectrum

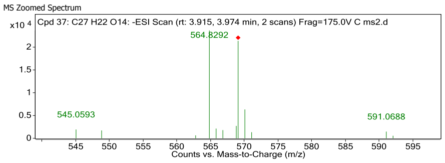

MSMS Spectrum

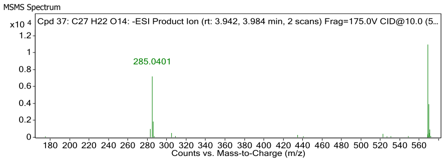

MSMS Spectrum

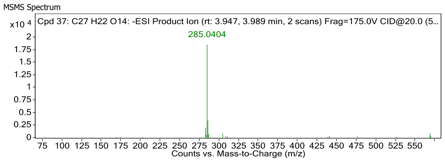

MSMS Spectrum

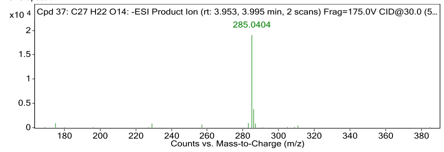

Compound Label	Name	*m/z*	RT	Algorithm	Mass
Cpd 38: C22 H22 O11		461.109	4.014	Auto MS/MS	462.11627

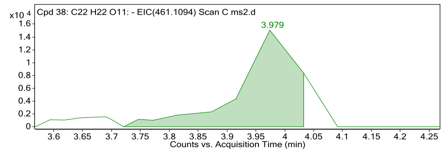

MS Spectrum

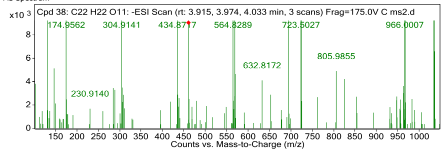

MS Zoomed Spectrum

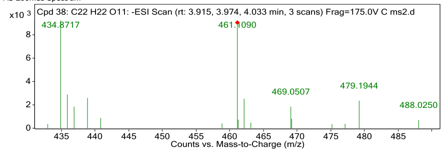

MSMS Spectrum

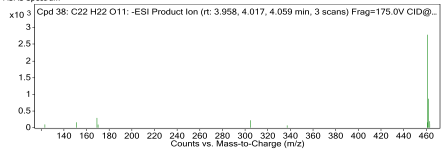

MSMS Spectrum

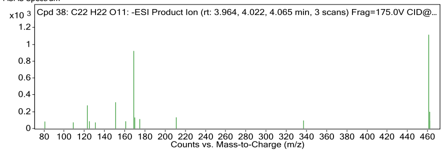

MSMS Spectrum

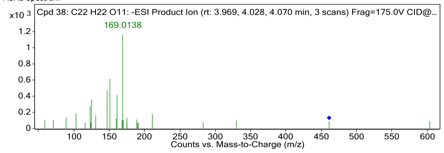

Compound Label	Name	*m/z*	RT	Algorithm	Mass
Cpd 39: C41 H72 O10		723.5027	4.088	Auto MS/MS	724.51005

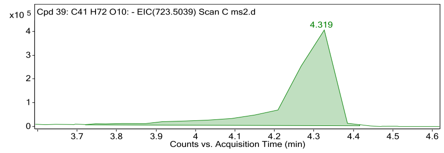

MS Spectrum

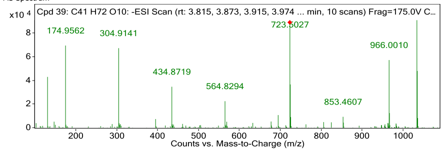

MS Zoomed Spectrum

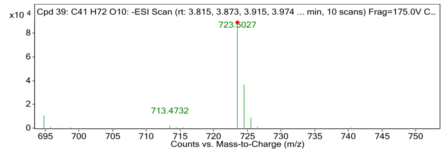

MSMS Spectrum

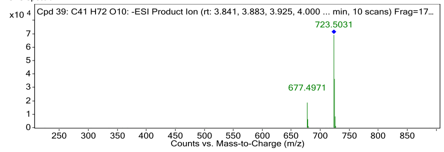

MSMS Spectrum

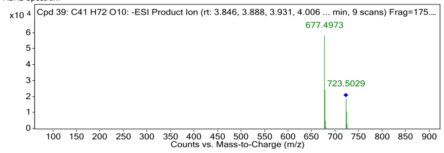

MSMS Spectrum

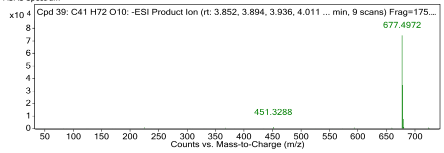

Compound Label	Name	*m/z*	RT	Algorithm	Mass
Cpd 40: C44 H70 O16		853.4608	4.14	Auto MS/MS	854.46788

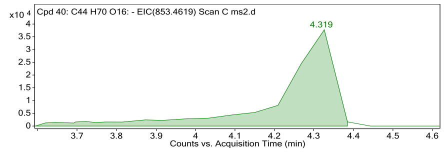

MS Spectrum

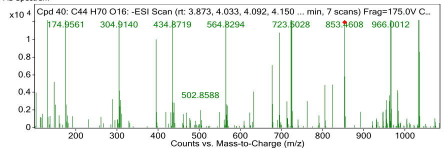

MS Zoomed Spectrum

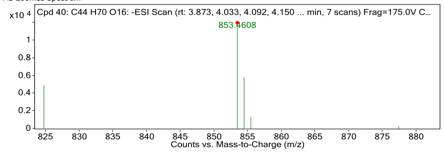

MSMS Spectrum

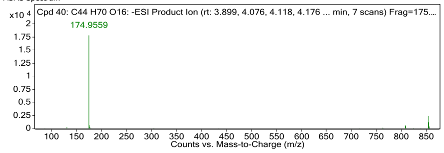

MSMS Spectrum

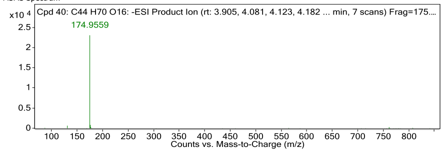

MSMS Spectrum

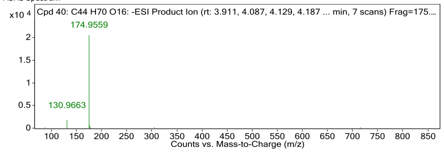

Compound Label	Name	m/z	RT	Algorithm	Mass
Cpd 41: C40 H70 O8		677.4974	4.228	Auto MS/MS	678.5045

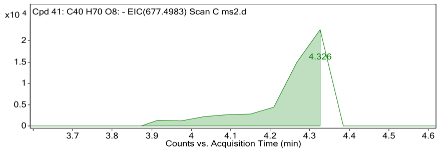

MS Spectrum

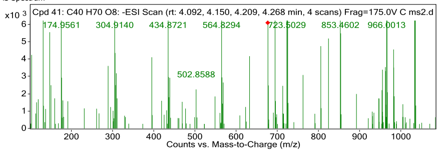

MS Zoomed Spectrum

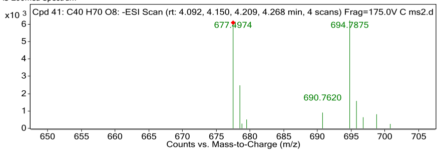

MSMS Spectrum

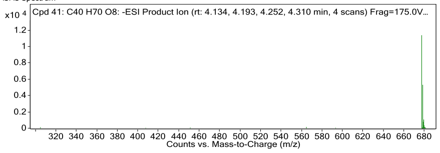

MSMS Spectrum

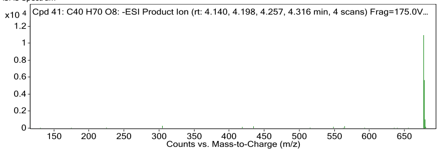

MSMS Spectrum

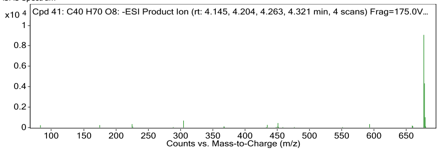

Compound Label	Name	m/z	RT	Algorithm	Mass
Cpd 42: 4.358		395.1387	4.358	Auto MS/MS	

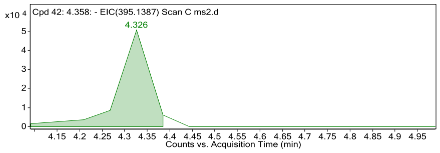

MS Spectrum

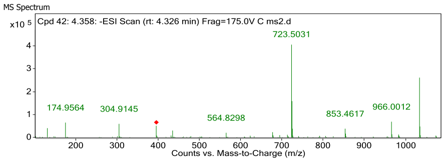

MS Zoomed Spectrum

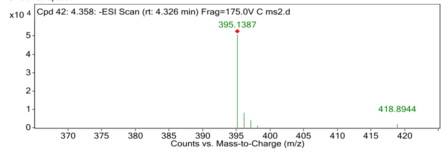

MSMS Spectrum

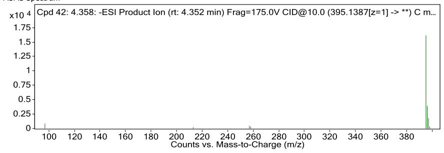

MSMS Spectrum

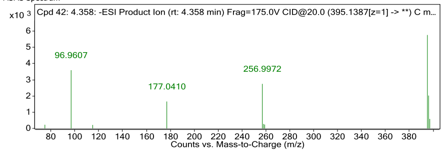

MSMS Spectrum

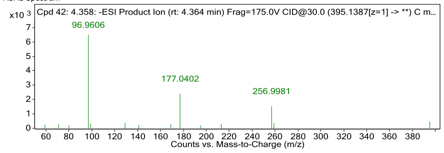

Compound Label	Name	m/z	RT	Algorithm	Mass
Cpd 43: C41 H72 O10		723.5027	4.374	Auto MS/MS	724.51007

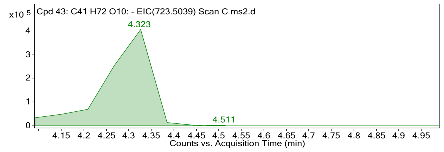

MS Spectrum

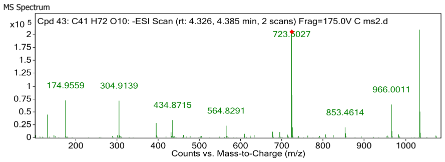

MS Zoomed Spectrum

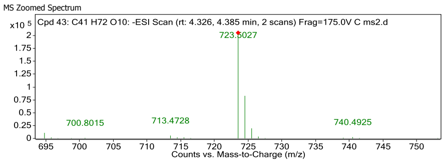

MSMS Spectrum

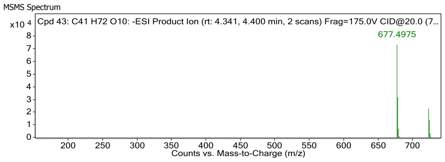

MSMS Spectrum

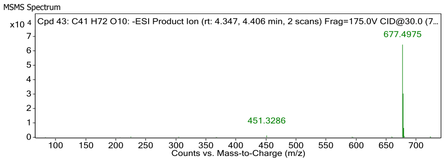

MSMS Spectrum

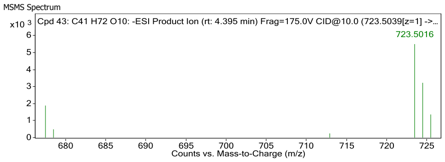

Compound Label	Name	*m/z*	RT	Algorithm	Mass
Cpd 44: 4.417		431.1337	4.417	Auto MS/MS	

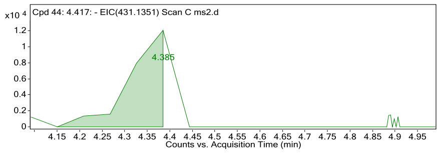

MS Spectrum

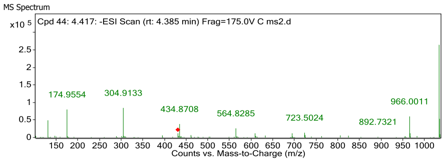

MS Zoomed Spectrum

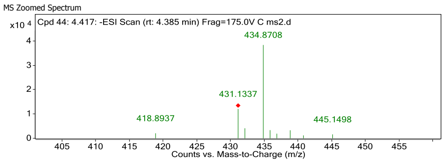

MSMS Spectrum

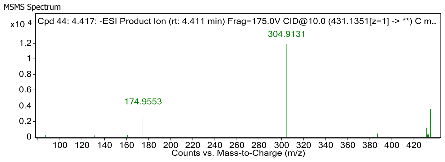

MSMS Spectrum

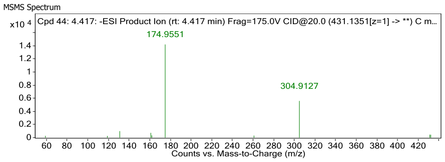

MSMS Spectrum

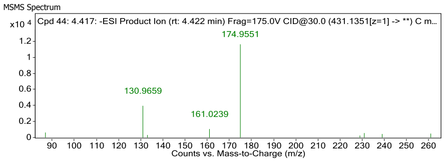

Compound Label	Name	*m/z*	RT	Algorithm	Mass
Cpd 45: C27 H30 O16		609.1458	4.433	Auto MS/MS	610.15299

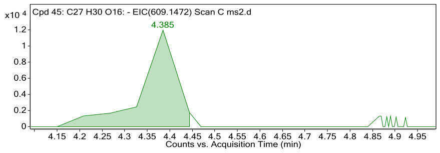

MS Spectrum

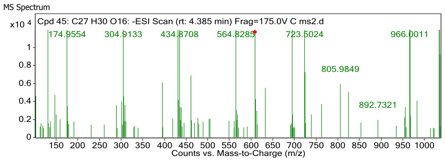

MS Zoomed Spectrum

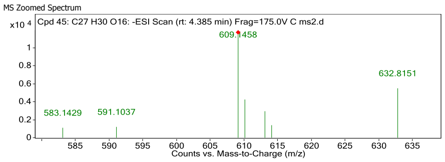

MSMS Spectrum

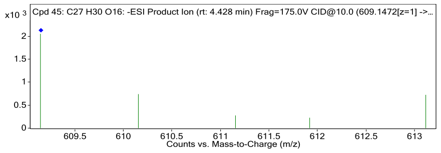

MSMS Spectrum

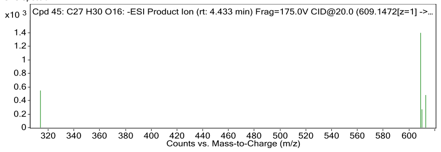

MSMS Spectrum

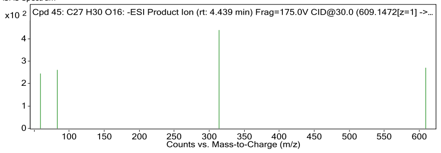

Compound Label	Name	*m/z*	RT	Algorithm	Mass
Cpd 46: C22 H22 O12		477.1035	4.597	Auto MS/MS	478.11082

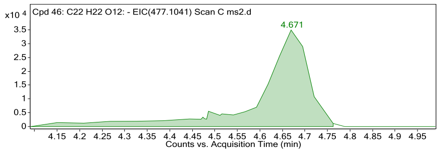

MS Spectrum

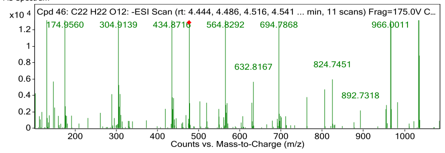

MS Zoomed Spectrum

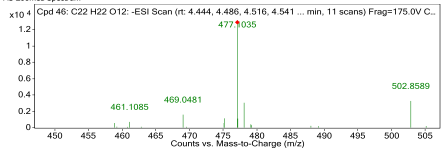

MSMS Spectrum

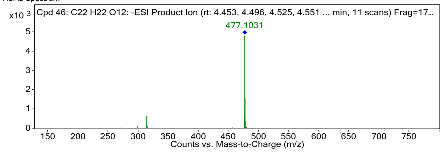

MSMS Spectrum

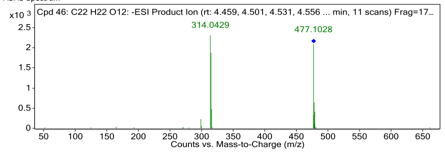

MSMS Spectrum

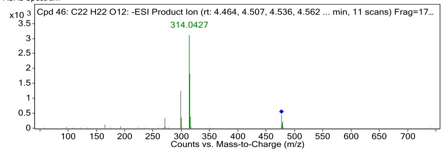

Compound Label	Name	*m/z*	RT	Algorithm	Mass
Cpd 47: C23 H26 O9		445.1499	4.967	Auto MS/MS	446.15716

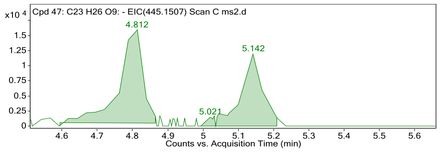

MS Spectrum

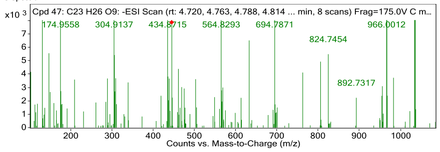

MS Zoomed Spectrum

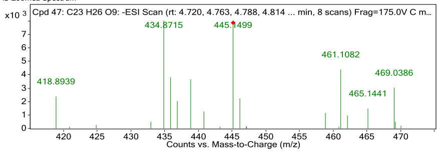

MSMS Spectrum

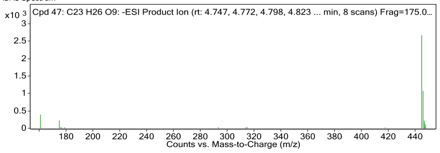

MSMS Spectrum

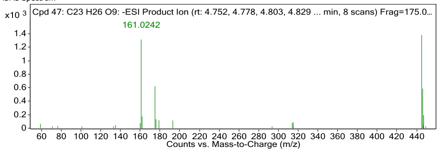

MSMS Spectrum

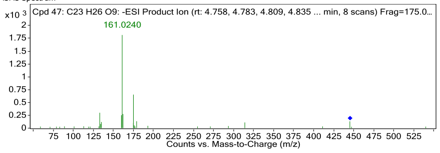

Compound Label	Name	*m/z*	RT	Algorithm	Mass
Cpd 48: C65 H90 O5		949.6708	5.206	Auto MS/MS	950.67797

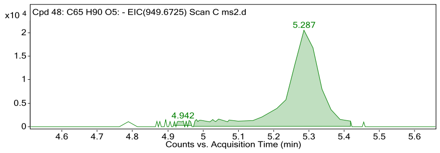

MS Spectrum

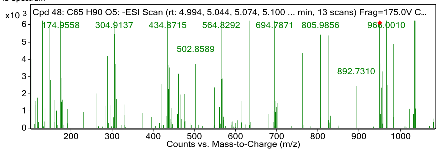

MS Zoomed Spectrum

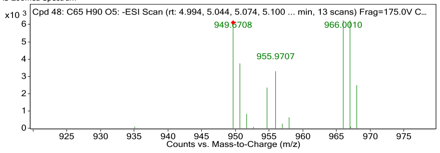

MSMS Spectrum

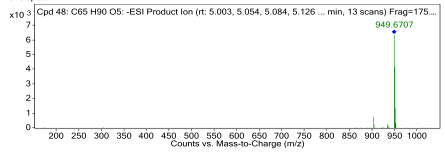

MSMS Spectrum

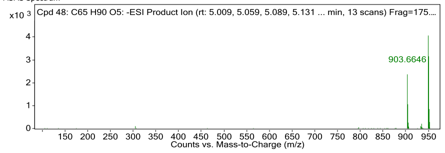

MSMS Spectrum

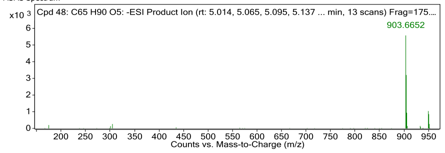

Compound Label	Name	*m/z*	RT	Algorithm	Mass
Cpd 49: 8.524		383.1899	8.524	Auto MS/MS	

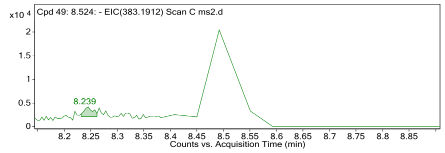

MS Spectrum

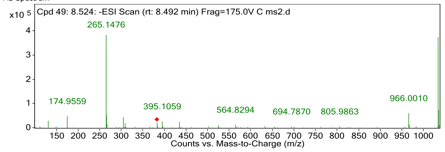

MS Zoomed Spectrum

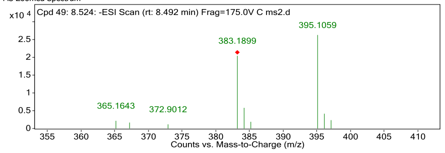

MSMS Spectrum

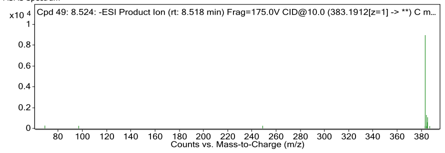

MSMS Spectrum

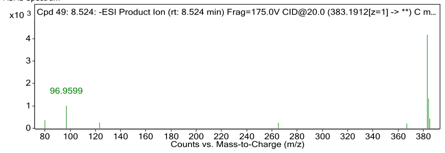

MSMS Spectrum

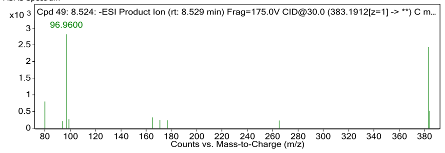

Compound Label	Name	*m/z*	RT	Algorithm	Mass
Cpd 50: 8.532		525.0637	8.532	Auto MS/MS	

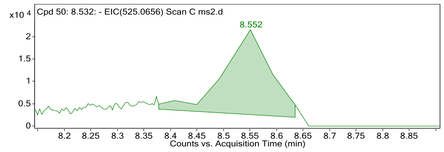

MS Spectrum

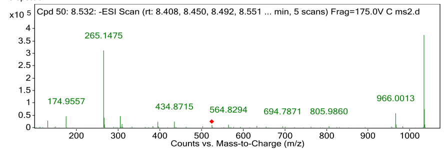

MS Zoomed Spectrum

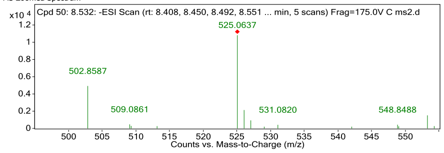

MSMS Spectrum

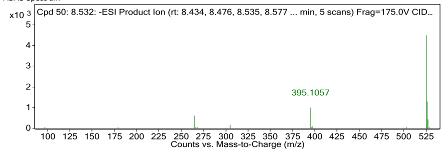

MSMS Spectrum

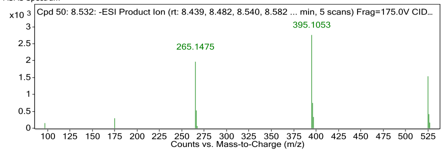

MSMS Spectrum

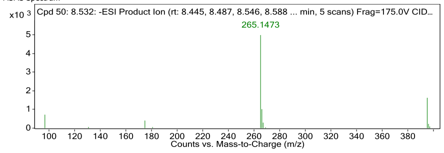

Compound Label	Name	*m/z*	RT	Algorithm	Mass
Cpd 51: 8.551		265.1478	8.551	Auto MS/MS	

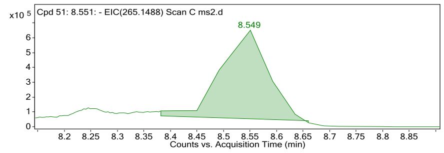

MS Spectrum

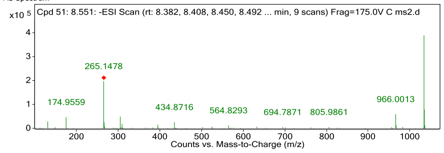

MS Zoomed Spectrum

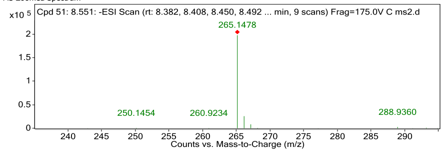

MSMS Spectrum

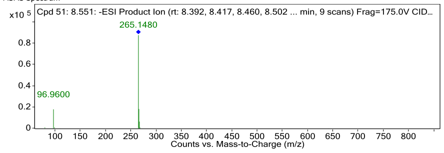

MSMS Spectrum

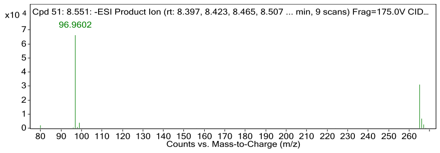

MSMS Spectrum

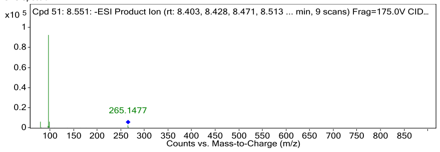

Compound Label	Name	*m/z*	RT	Algorithm	Mass
Cpd 52: 8.896		851.3253	8.896	Auto MS/MS	

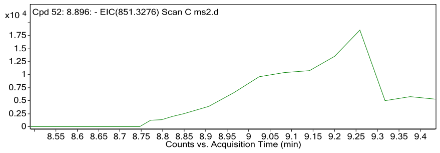

MS Spectrum

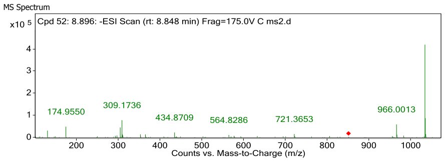

MS Zoomed Spectrum

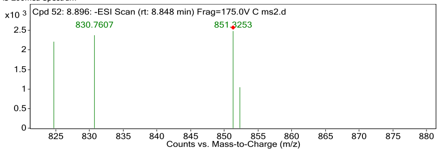

MSMS Spectrum

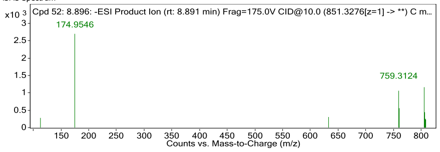

MSMS Spectrum

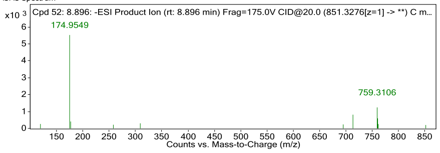

MSMS Spectrum

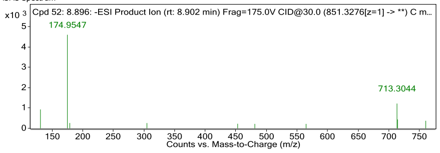

Compound Label	Name	*m/z*	RT	Algorithm	Mass
Cpd 53: 8.918		365.1636	8.918	Auto MS/MS	

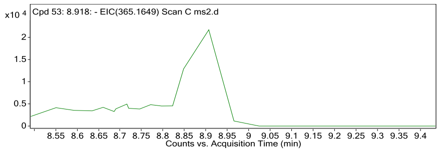

MS Spectrum

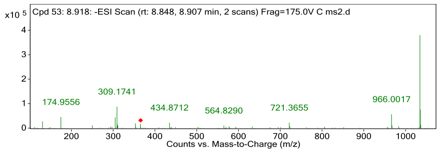

MS Zoomed Spectrum

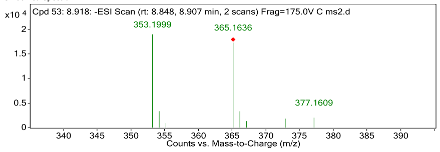

MSMS Spectrum

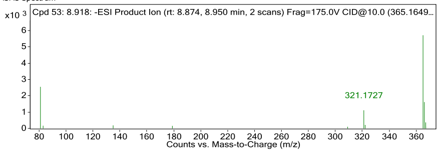

MSMS Spectrum

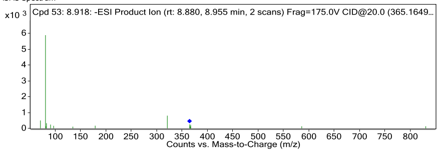

MSMS Spectrum

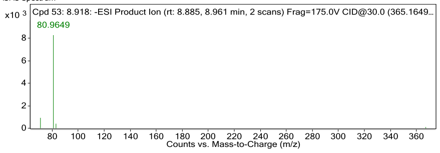

Compound Label	Name	*m/z*	RT	Algorithm	Mass
Cpd 54: C34 H58 O16		721.3658	8.978	Auto MS/MS	722.37303

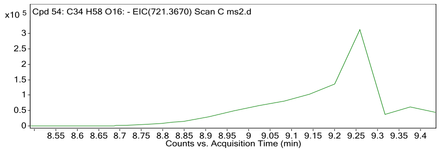

MS Spectrum

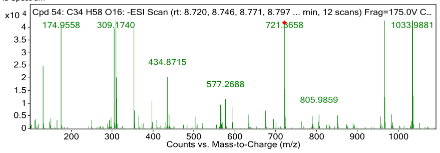

MS Zoomed Spectrum

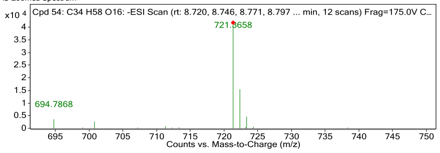

MSMS Spectrum

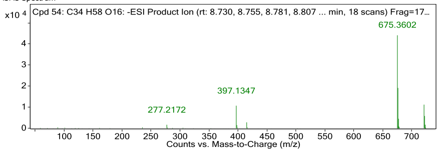

MSMS Spectrum

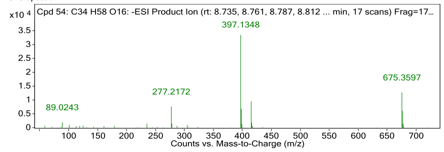

MSMS Spectrum

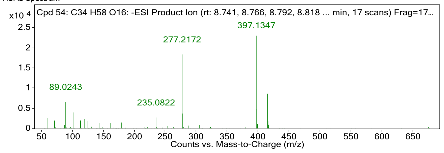

Compound Label	Name	*m/z*	RT	Algorithm	Mass
Cpd 55: C33 H56 O14		675.3594	9.014	Auto MS/MS	676.36643

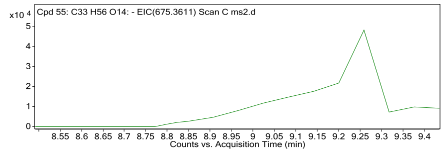

MS Spectrum

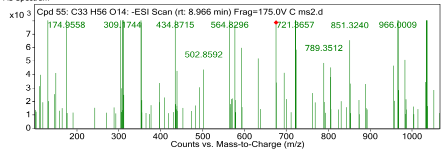

MS Zoomed Spectrum

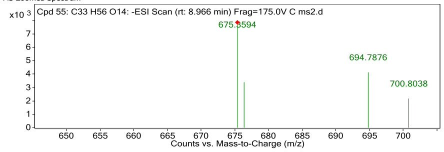

MSMS Spectrum

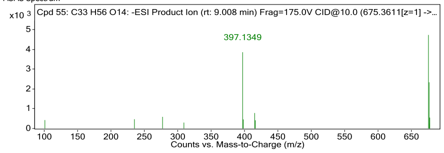

MSMS Spectrum

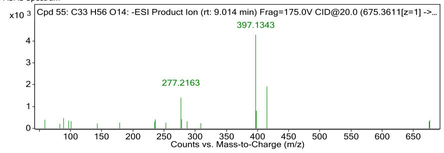

MSMS Spectrum

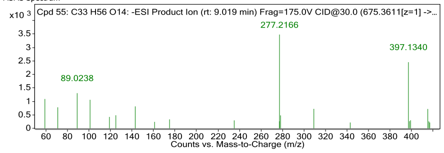

Compound Label	Name	*m/z*	RT	Algorithm	Mass
Cpd 56: 9.069		309.1738	9.069	Auto MS/MS	

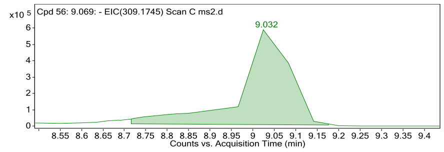

MS Spectrum

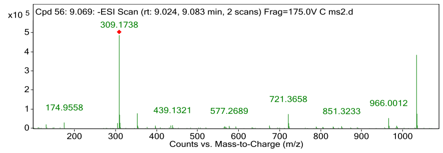

MS Zoomed Spectrum

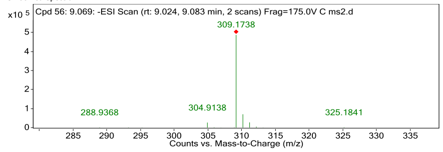

MSMS Spectrum

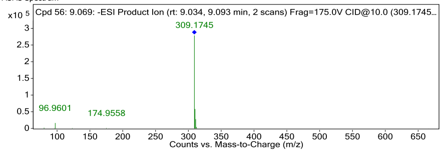

MSMS Spectrum

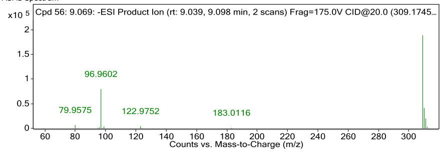

MSMS Spectrum

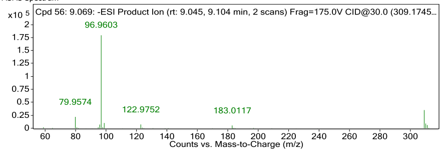

Compound Label	Name	*m/z*	RT	Algorithm	Mass
Cpd 57: C23 H46 O16		577.2693	9.19	Auto MS/MS	578.27621

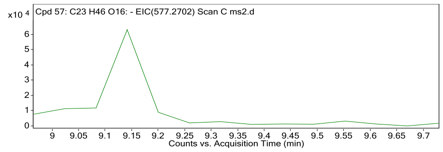

MS Spectrum

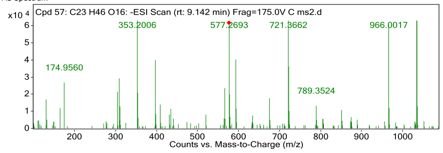

MS Zoomed Spectrum

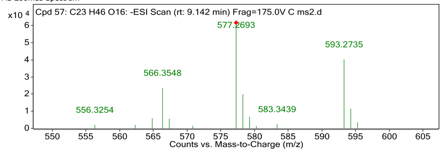

MSMS Spectrum

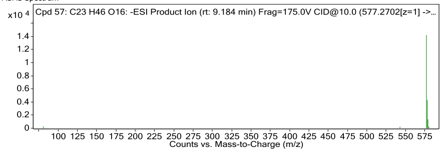

MSMS Spectrum

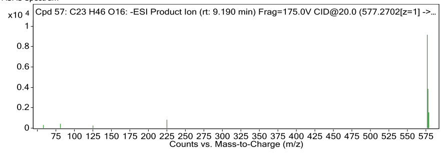

MSMS Spectrum

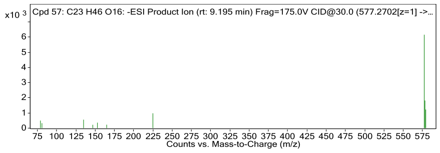

Compound Label	Name	*m/z*	RT	Algorithm	Mass
Cpd 58: 9.304		353.2001	9.304	Auto MS/MS	

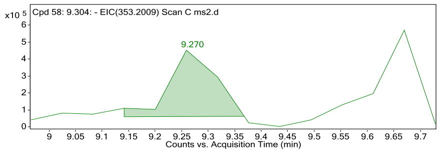

MS Spectrum

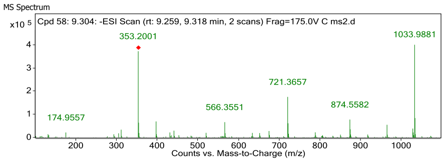

MS Zoomed Spectrum

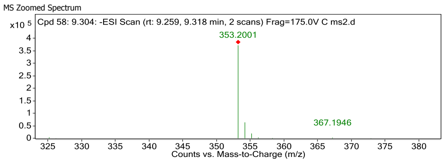

MSMS Spectrum

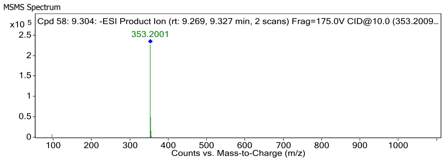

MSMS Spectrum

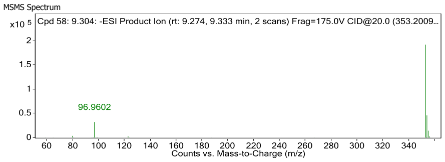

MSMS Spectrum

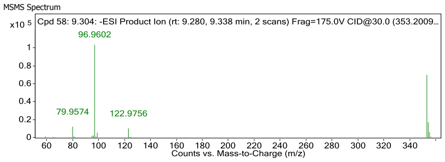

Compound Label	Name	*m/z*	RT	Algorithm	Mass
Cpd 59: C34 H58 O16		721.3658	9.323	Auto MS/MS	722.37318

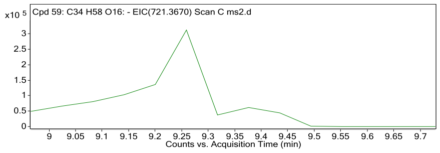

MS Spectrum

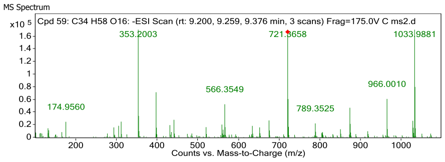

MS Zoomed Spectrum

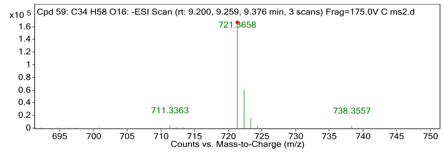

MSMS Spectrum

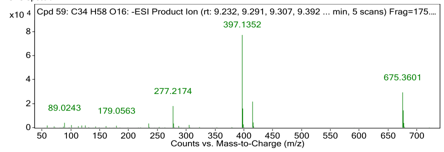

MSMS Spectrum

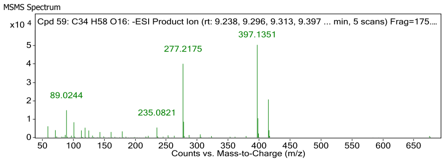

MSMS Spectrum

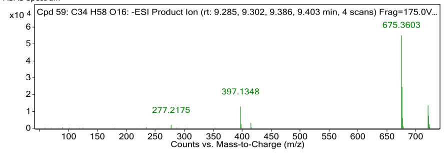

Compound Label	Name	*m/z*	RT	Algorithm	Mass
Cpd 60: 9.387		566.3554	9.387	Auto MS/MS	

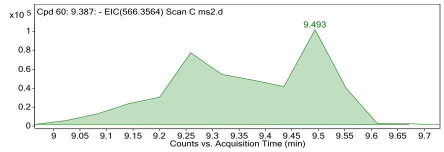

MS Spectrum

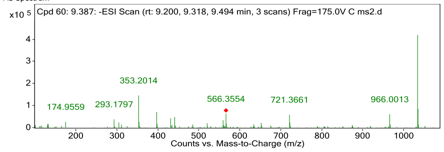

MS Zoomed Spectrum

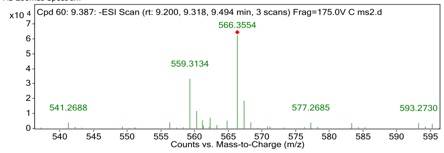

MSMS Spectrum

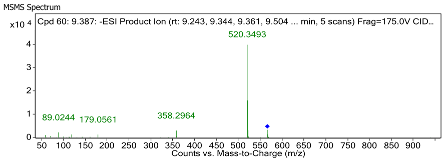

MSMS Spectrum

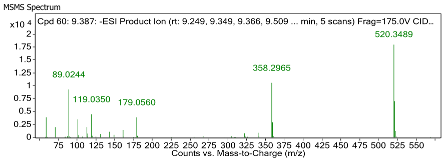

MSMS Spectrum

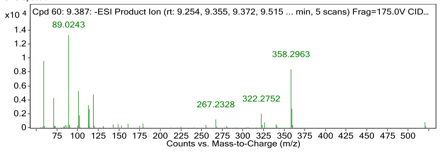

Compound Label	Name	*m/z*	RT	Algorithm	Mass
Cpd 61: 9.496		397.2262	9.496	Auto MS/MS	

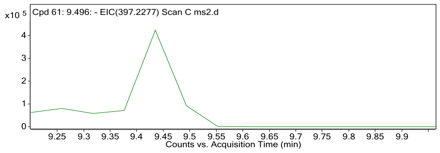

MS Spectrum

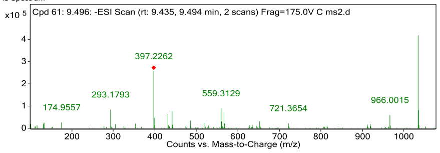

MS Zoomed Spectrum

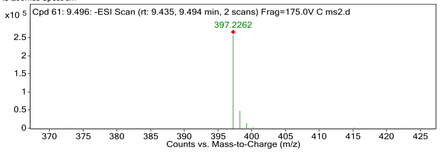

MSMS Spectrum

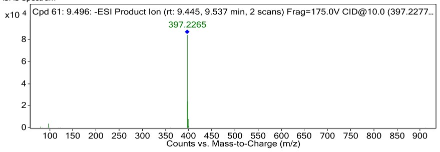

MSMS Spectrum

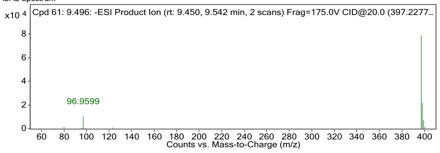

MSMS Spectrum

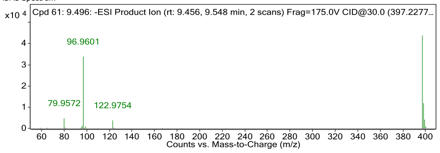

Compound Label	Name	*m/z*	RT	Algorithm	Mass
Cpd 62: C28 H48 O11		559.313	9.542	Auto MS/MS	560.32024

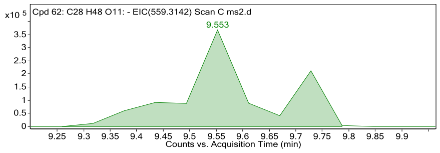

MS Spectrum

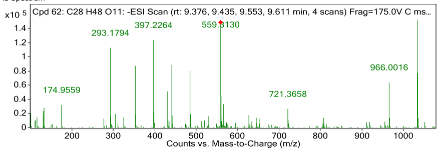

MS Zoomed Spectrum

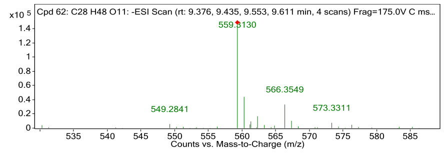

MSMS Spectrum

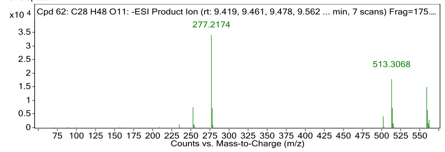

MSMS Spectrum

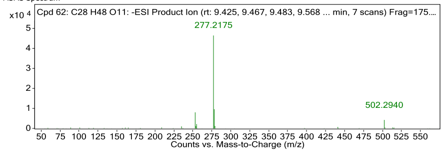

MSMS Spectrum

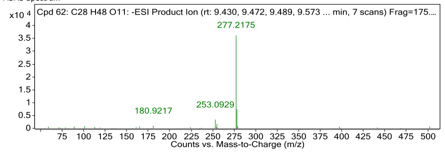

Compound Label	Name	*m/z*	RT	Algorithm	Mass
Cpd 63: 9.601		441.2529	9.601	Auto MS/MS	

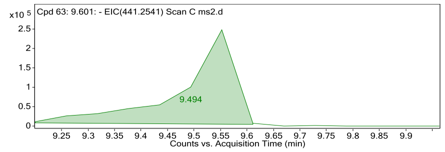

MS Spectrum

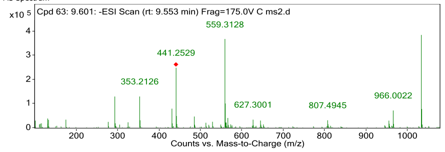

MS Zoomed Spectrum

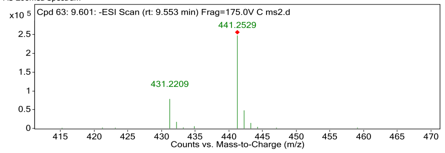

MSMS Spectrum

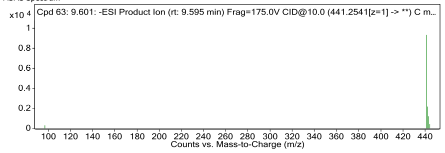

MSMS Spectrum

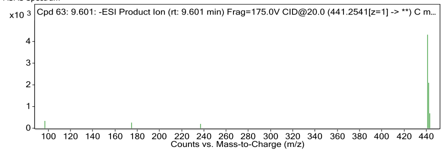

MSMS Spectrum

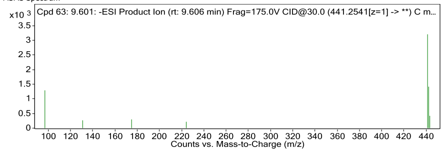

Compound Label	Name	*m/z*	RT	Algorithm	Mass
Cpd 64: 9.672		485.2791	9.672	Auto MS/MS	

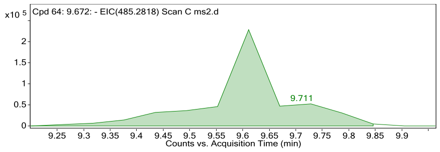

MS Spectrum

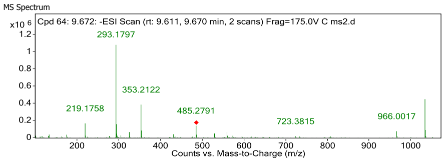

MS Zoomed Spectrum

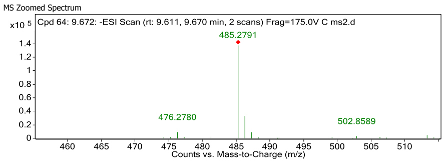

MSMS Spectrum

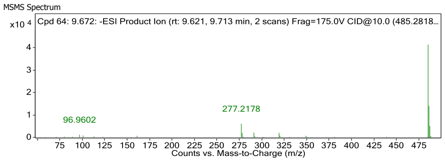

MSMS Spectrum

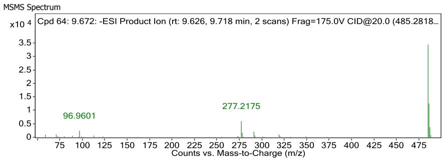

MSMS Spectrum

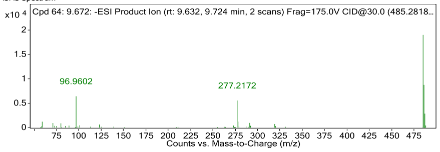

Compound Label	Name	*m/z*	RT	Algorithm	Mass
Cpd 65: 9.760		293.179	9.76	Auto MS/MS	

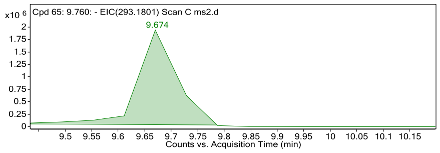

MS Spectrum

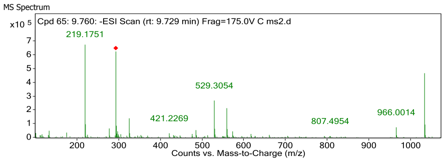

MS Zoomed Spectrum

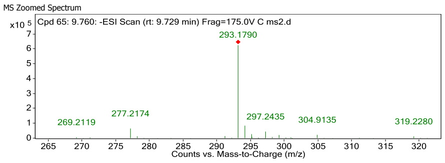

MSMS Spectrum

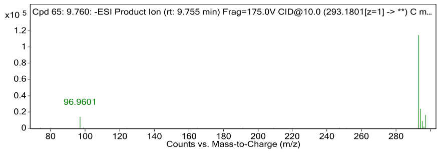

MSMS Spectrum

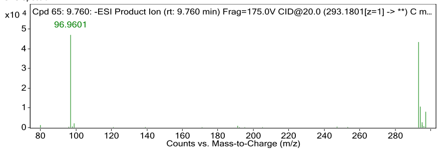

MSMS Spectrum

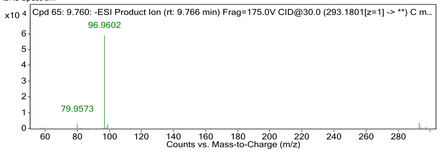

Compound Label	Name	*m/z*	RT	Algorithm	Mass
Cpd 66: 9.777		529.3054	9.777	Auto MS/MS	

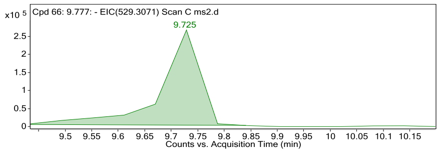

MS Spectrum

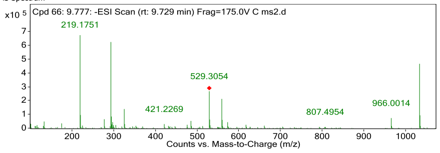

MS Zoomed Spectrum

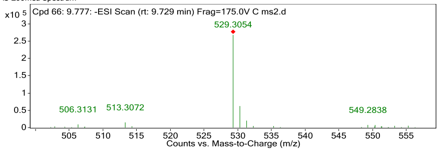

MSMS Spectrum

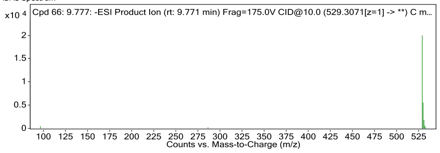

MSMS Spectrum

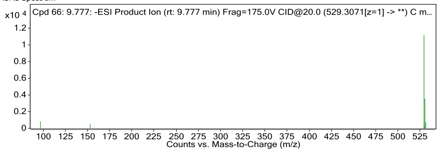

MSMS Spectrum

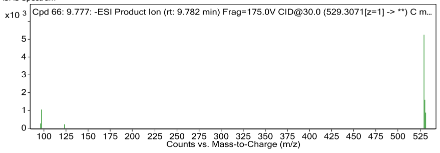

Compound Label	Name	*m/z*	RT	Algorithm	Mass
Cpd 67: C14 H30 O8		325.1843	9.781	Auto MS/MS	326.19142

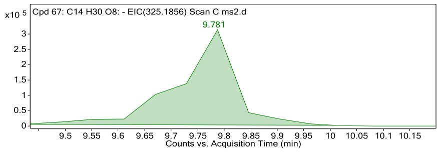

MS Spectrum

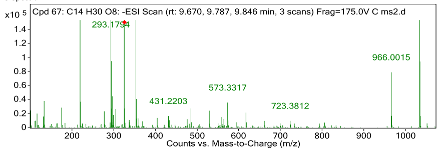

MS Zoomed Spectrum

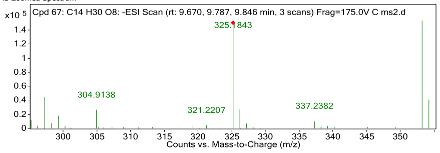

MSMS Spectrum

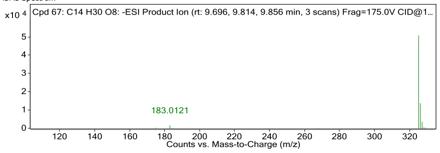

MSMS Spectrum

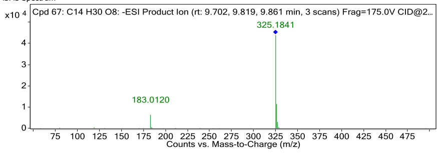

MSMS Spectrum

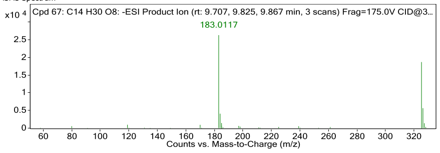

Compound Label	Name	m/z	RT	Algorithm	Mass
Cpd 68: C15 H24 O		219.1752	9.79	Auto MS/MS	220.18253

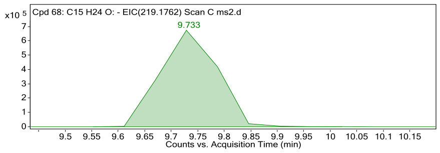

MS Spectrum

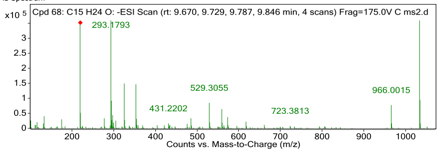

MS Zoomed Spectrum

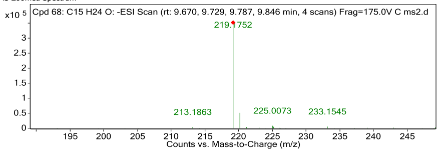

MSMS Spectrum

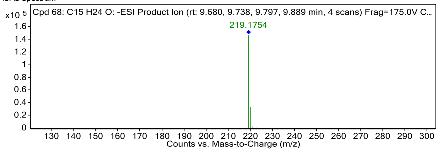

MSMS Spectrum

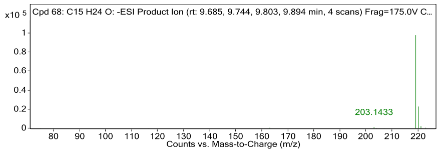

MSMS Spectrum

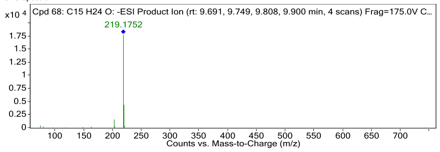

Compound Label	Name	*m/z*	RT	Algorithm	Mass
Cpd 69: 9.836		297.2433	9.836	Auto MS/MS	

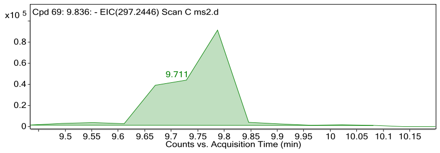

MS Spectrum

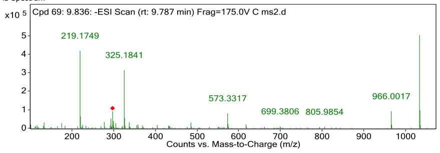

MS Zoomed Spectrum

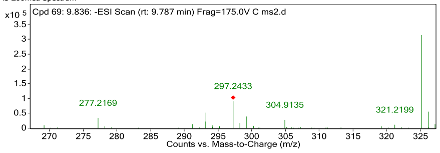

MSMS Spectrum

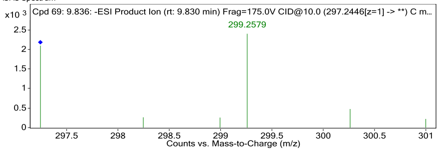

MSMS Spectrum

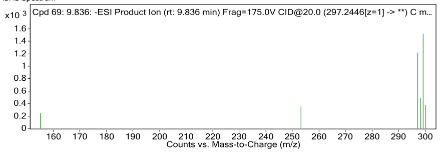

MSMS Spectrum

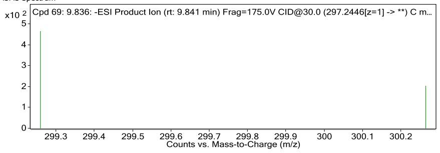

Compound Label	Name	*m/z*	RT	Algorithm	Mass
Cpd 70: 9.928		404.3013	9.928	Auto MS/MS	

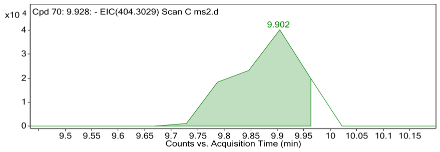

MS Spectrum

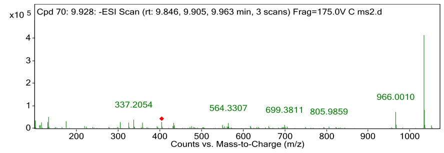

MS Zoomed Spectrum

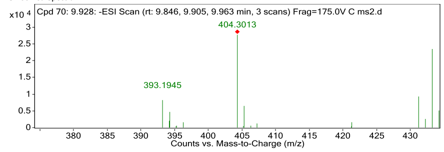

MSMS Spectrum

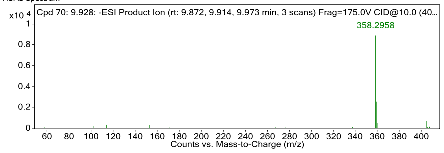

MSMS Spectrum

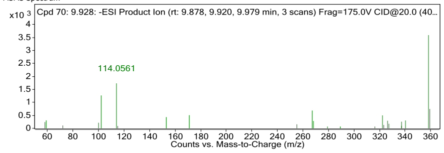

MSMS Spectrum

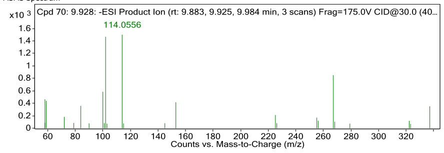

Compound Label	Name	*m/z*	RT	Algorithm	Mass
Cpd 71: 9.953		130.9436	9.953	Auto MS/MS	

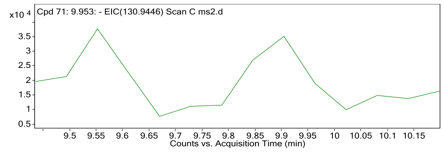

MS Spectrum

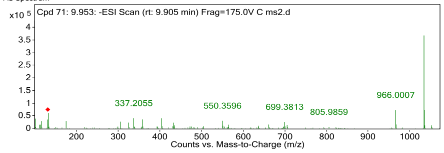

MS Zoomed Spectrum

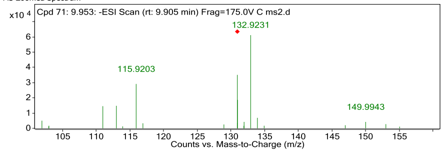

MSMS Spectrum

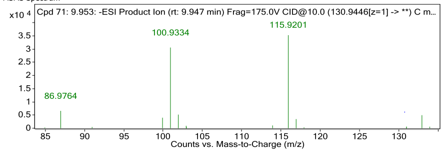

MSMS Spectrum

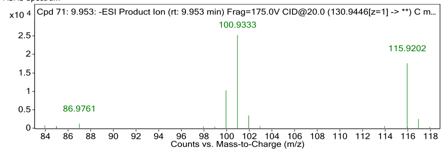

MSMS Spectrum

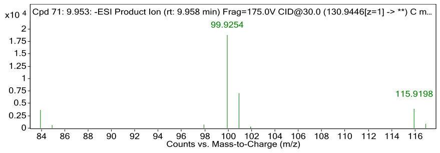

Compound Label	Name	*m/z*	RT	Algorithm	Mass
Cpd 72: 9.966		358.2961	9.966	Auto MS/MS	

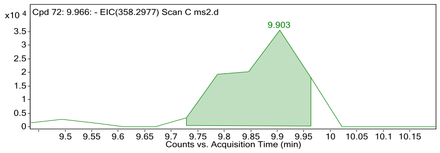

MS Spectrum

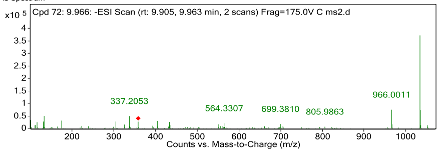

MS Zoomed Spectrum

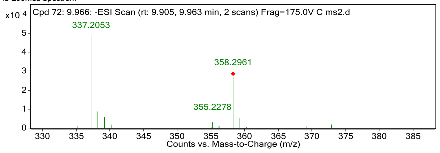

MSMS Spectrum

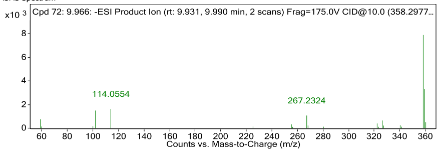

MSMS Spectrum

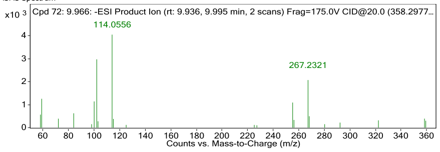

MSMS Spectrum

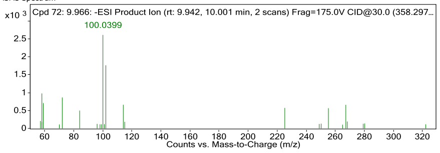

Compound Label	Name	*m/z*	RT	Algorithm	Mass
Cpd 73: C27 H41 O7		476.2779	10.062	Auto MS/MS	477.28506

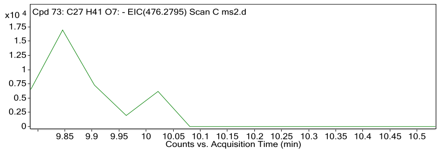

MS Spectrum

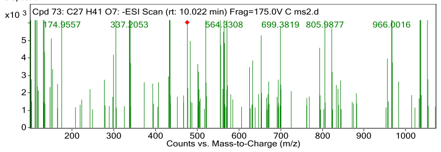

MS Zoomed Spectrum

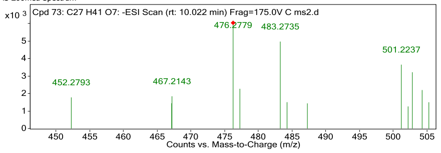

MSMS Spectrum

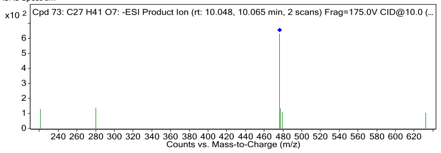

MSMS Spectrum

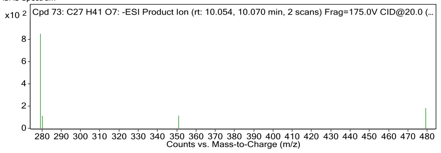

MSMS Spectrum

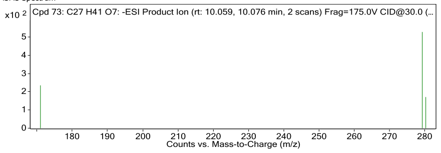

Compound Label	Name	*m/z*	RT	Algorithm	Mass
Cpd 74: C32 H60 O16		699.381	10.07	Auto MS/MS	700.38784

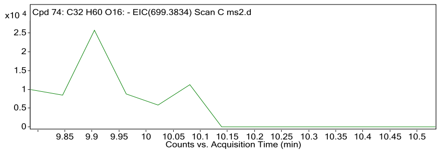

MS Spectrum

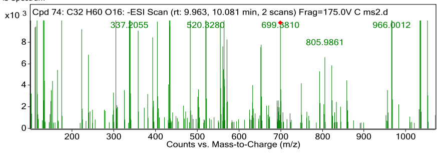

MS Zoomed Spectrum

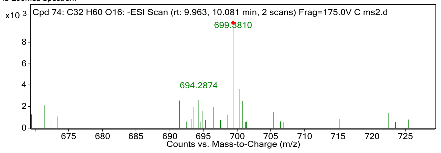

MSMS Spectrum

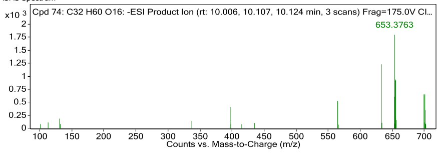

MSMS Spectrum

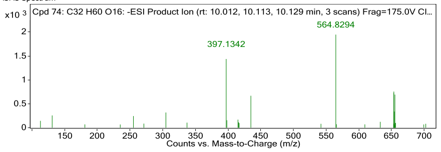

MSMS Spectrum

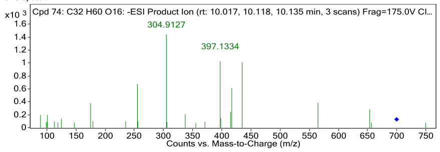

Compound Label	Name	*m/z*	RT	Algorithm	Mass
Cpd 75: C26 H49 O10		520.3281	10.125	Auto MS/MS	521.33536

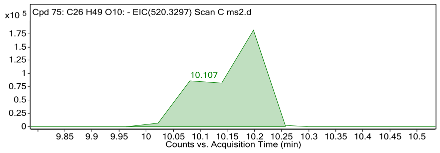

MS Spectrum

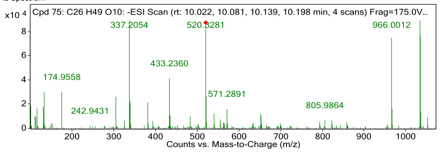

MS Zoomed Spectrum

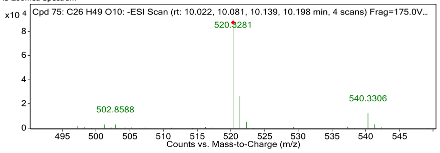

MSMS Spectrum

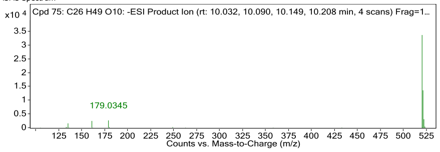

MSMS Spectrum

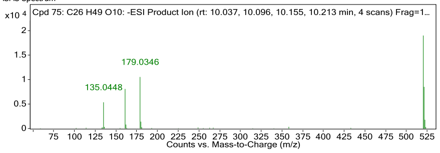

MSMS Spectrum

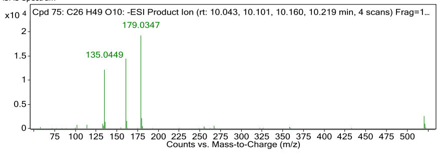

Compound Label	Name	*m/z*	RT	Algorithm	Mass
Cpd 76: 10.217		652.2801	10.217	Auto MS/MS	

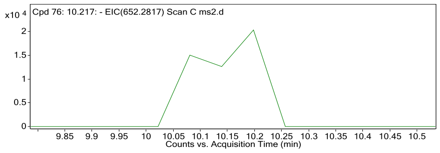

MS Spectrum

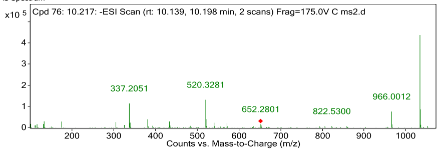

MS Zoomed Spectrum

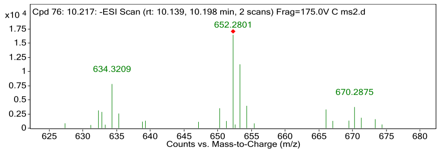

MSMS Spectrum

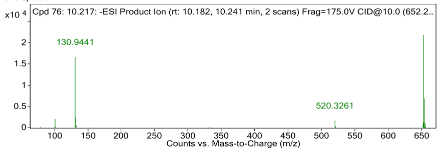

MSMS Spectrum

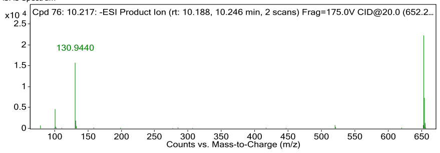

MSMS Spectrum

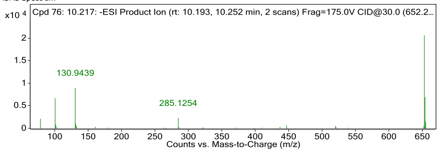

Compound Label	Name	*m/z*	RT	Algorithm	Mass
Cpd 77: C29 H49 O9		540.3307	10.23	Auto MS/MS	541.33786

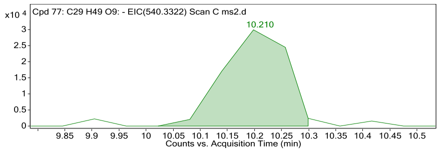

MS Spectrum

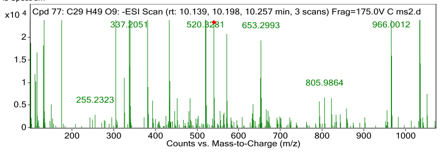

MS Zoomed Spectrum

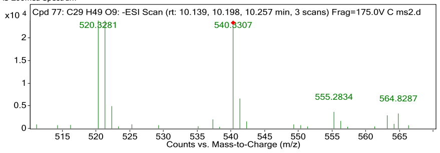

MSMS Spectrum

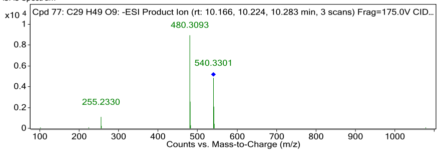

MSMS Spectrum

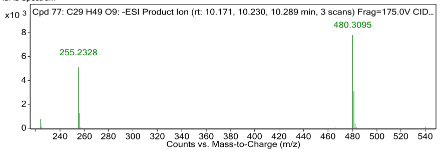

MSMS Spectrum

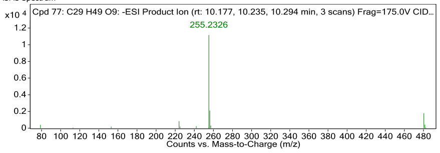

Compound Label	Name	*m/z*	RT	Algorithm	Mass
Cpd 78: C17 H34 O6		333.2288	10.368	Auto MS/MS	334.2355

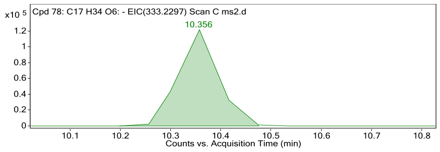

MS Spectrum

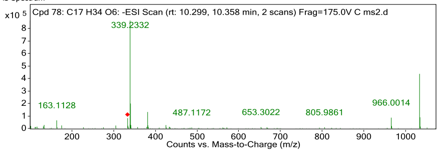

MS Zoomed Spectrum

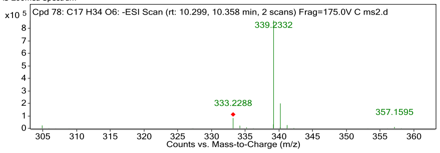

MSMS Spectrum

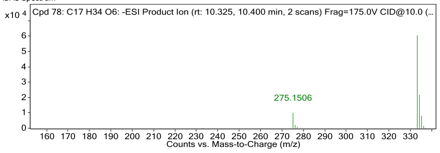

MSMS Spectrum

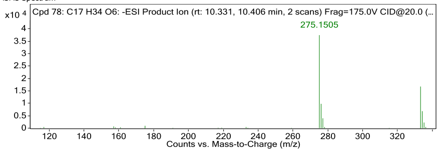

MSMS Spectrum

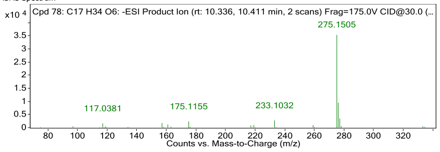

Compound Label	Name	*m/z*	RT	Algorithm	Mass
Cpd 79: C19 H32 O5		339.2177	10.427	Auto MS/MS	340.22502

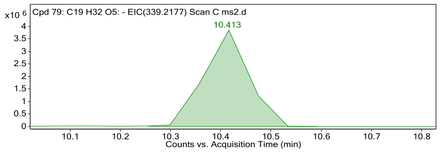

MS Spectrum

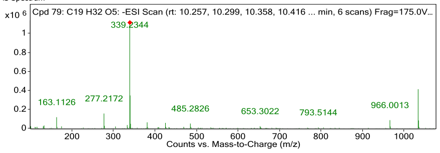

MS Zoomed Spectrum

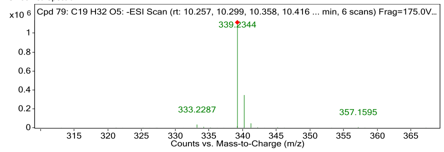

MSMS Spectrum

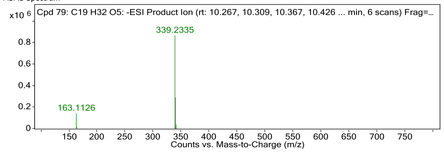

MSMS Spectrum

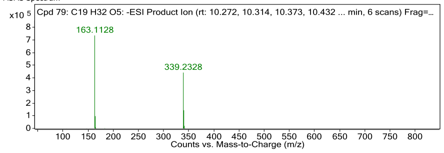

MSMS Spectrum

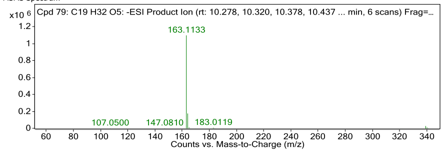

Compound Label	Name	*m/z*	RT	Algorithm	Mass
Cpd 80: C11 H16 O		163.1127	10.456	Auto MS/MS	164.11994

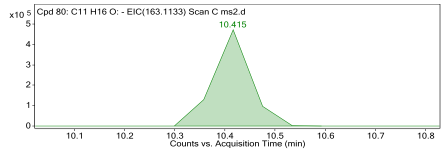

MS Spectrum

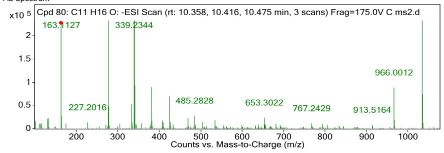

MS Zoomed Spectrum

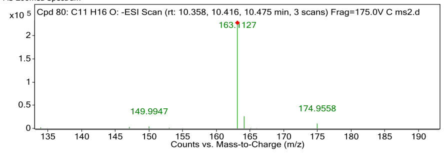

MSMS Spectrum

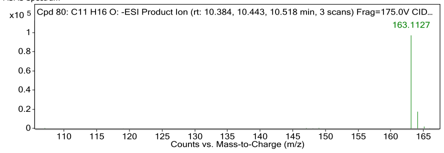

MSMS Spectrum

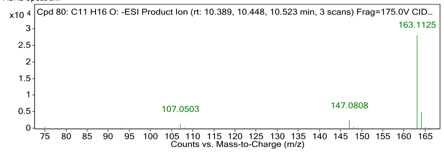

MSMS Spectrum

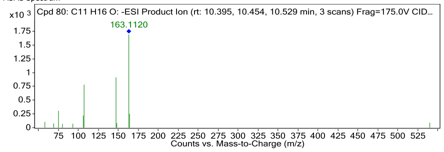

Compound Label	Name	*m/z*	RT	Algorithm	Mass
Cpd 81: C29 H50 O16		653.302	10.486	Auto MS/MS	654.30914

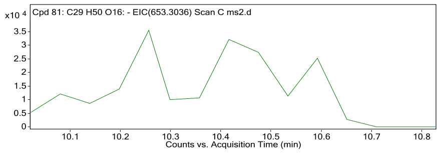

MS Spectrum

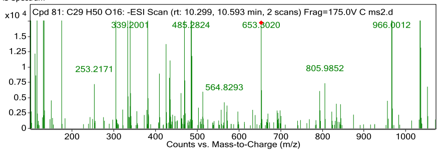

MS Zoomed Spectrum

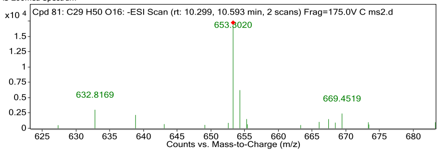

MSMS Spectrum

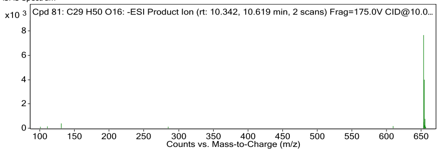

MSMS Spectrum

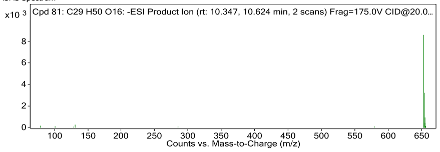

MSMS Spectrum

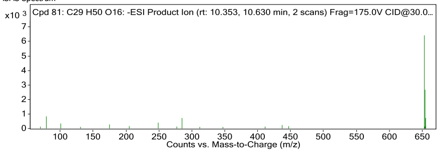

Compound Label	Name	*m/z*	RT	Algorithm	Mass
Cpd 82: C18 H30 O2		277.2171	10.515	Auto MS/MS	278.22449

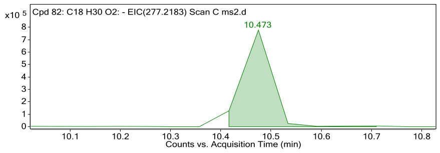

MS Spectrum

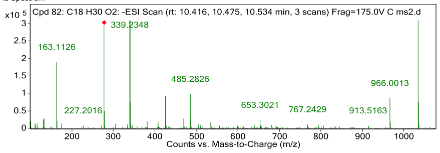

MS Zoomed Spectrum

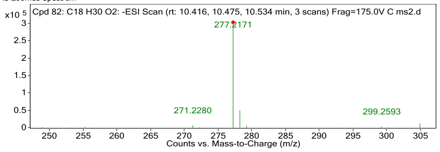

MSMS Spectrum

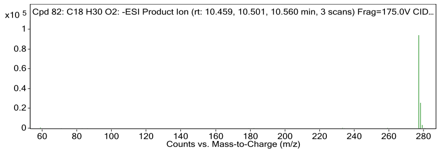

MSMS Spectrum

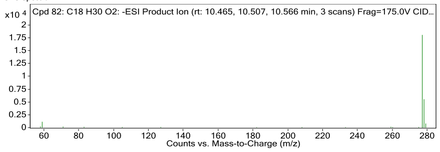

MSMS Spectrum

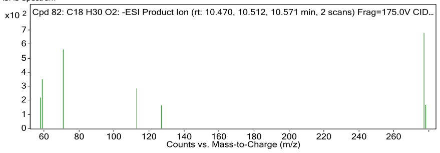

Compound Label	Name	*m/z*	RT	Algorithm	Mass
Cpd 83: C36 H38 O		485.2825	10.578	Auto MS/MS	486.28992

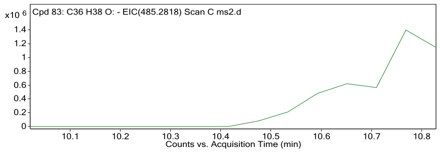

MS Spectrum

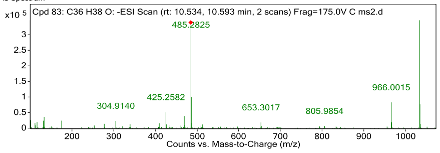

MS Zoomed Spectrum

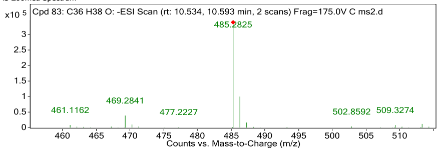

MSMS Spectrum

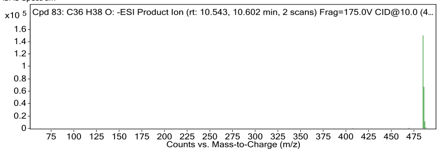

MSMS Spectrum

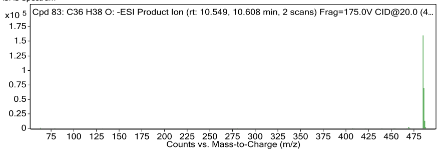

MSMS Spectrum

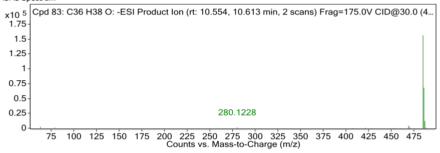

Compound Label	Name	*m/z*	RT	Algorithm	Mass
Cpd 84: C16 H30 O2		253.2174	10.662	Auto MS/MS	254.22522

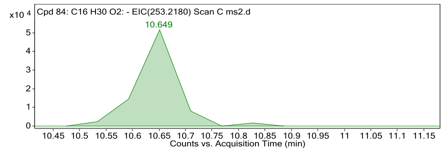

MS Spectrum

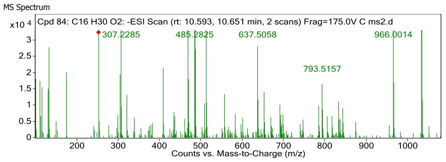

MS Zoomed Spectrum

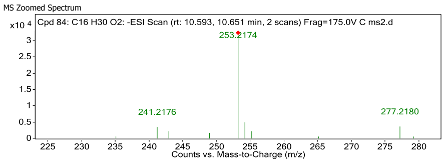

MSMS Spectrum

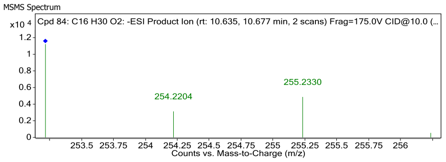

MSMS Spectrum

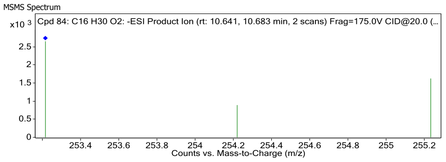

MSMS Spectrum

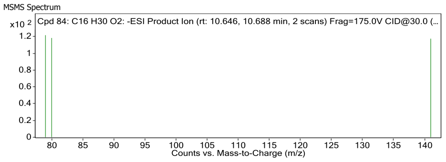

Compound Label	Name	*m/z*	RT	Algorithm	Mass
Cpd 85: C19 H32 O3		307.2285	10.666	Auto MS/MS	308.23573

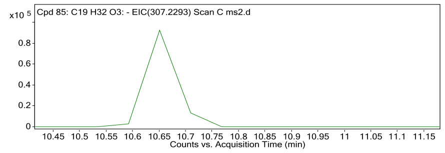

MS Spectrum

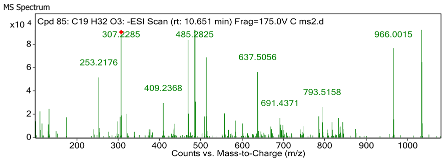

MS Zoomed Spectrum

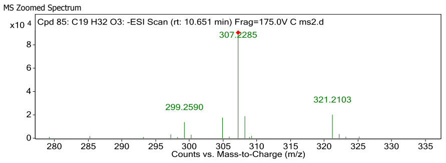

MSMS Spectrum

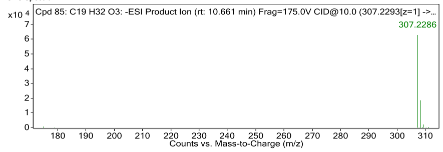

MSMS Spectrum

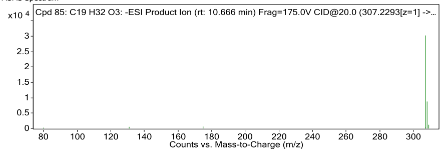

MSMS Spectrum

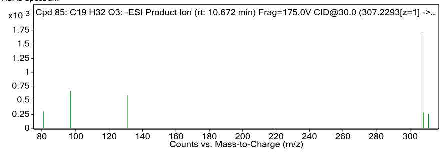

Compound Label	Name	*m/z*	RT	Algorithm	Mass
Cpd 86: C41 H70 O14		785.4698	10.699	Auto MS/MS	786.47682

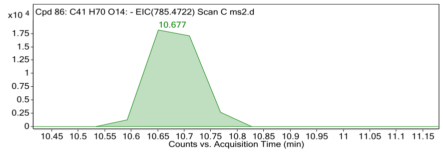

MS Spectrum

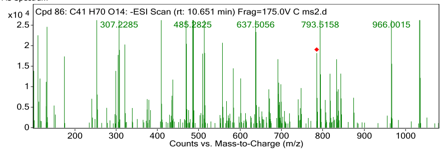

MS Zoomed Spectrum

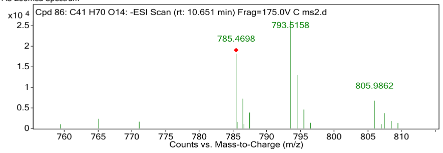

MSMS Spectrum

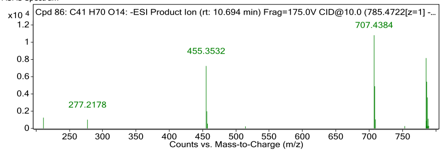

MSMS Spectrum

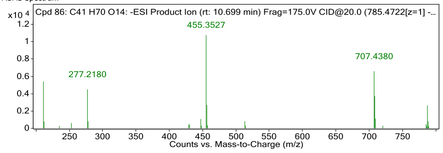

MSMS Spectrum

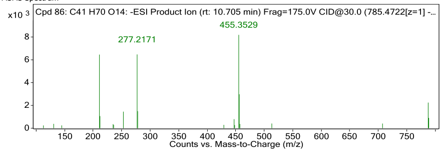

Compound Label	Name	*m/z*	RT	Algorithm	Mass
Cpd 87: C32 H48 O9		575.3233	10.725	Auto MS/MS	576.33076

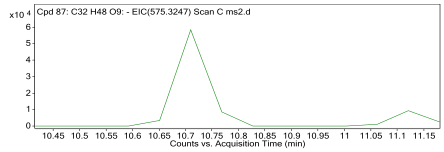

MS Spectrum

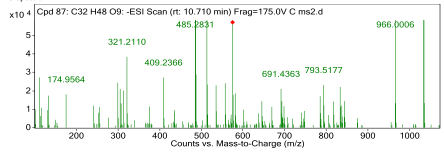

MS Zoomed Spectrum

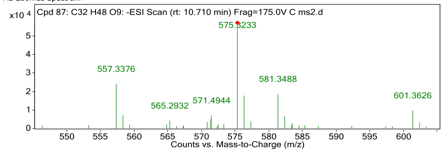

MSMS Spectrum

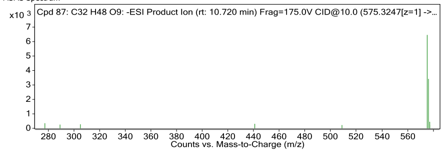

MSMS Spectrum

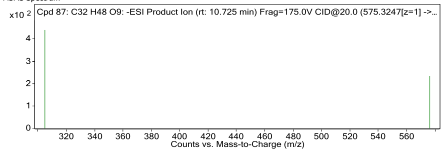

MSMS Spectrum

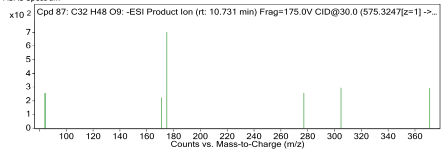

Compound Label	Name	*m/z*	RT	Algorithm	Mass
Cpd 88: C18 H36 O3		299.2599	10.742	Auto MS/MS	300.26709

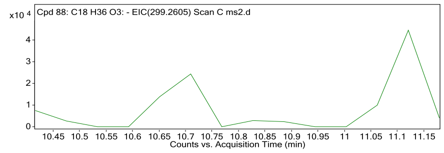

MS Spectrum

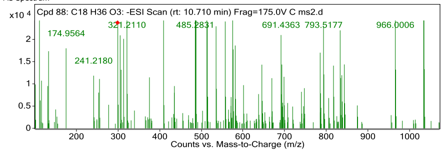

MS Zoomed Spectrum

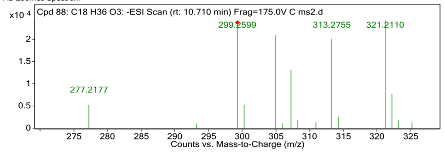

MSMS Spectrum

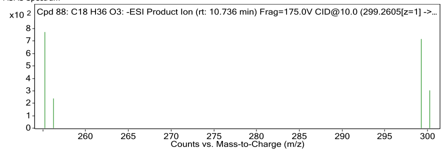

MSMS Spectrum

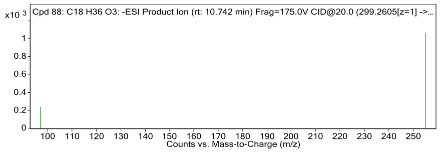

Compound Label	Name	*m/z*	RT	Algorithm	Mass
Cpd 89: C34 H48 O5		535.3434	10.787	Auto MS/MS	536.35063

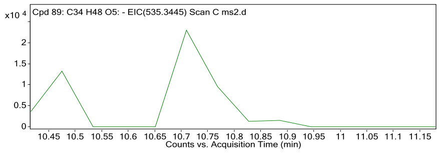

MS Spectrum

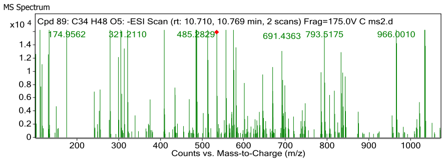

MS Zoomed Spectrum

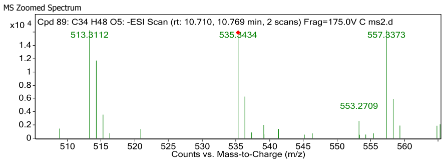

MSMS Spectrum

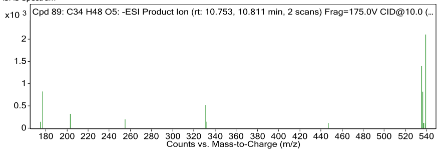

MSMS Spectrum

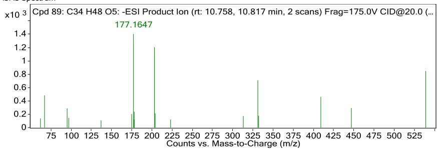

MSMS Spectrum

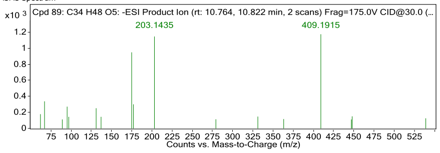

Compound Label	Name	*m/z*	RT	Algorithm	Mass
Cpd 90: C37 H50 O11		669.3288	10.859	Auto MS/MS	670.33639

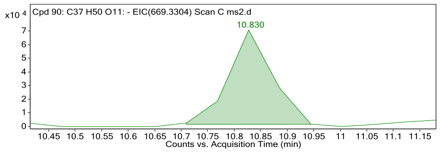

MS Spectrum

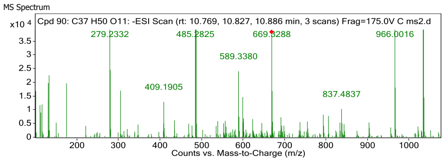

MS Zoomed Spectrum

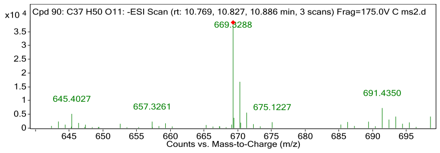

MSMS Spectrum

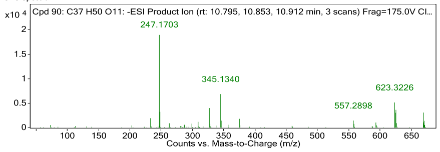

MSMS Spectrum

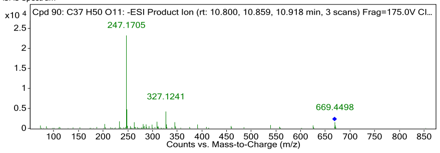

MSMS Spectrum

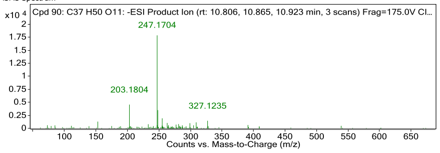

Compound Label	Name	*m/z*	RT	Algorithm	Mass
Cpd 91: C18 H32 O2		279.2332	10.859	Auto MS/MS	280.24045

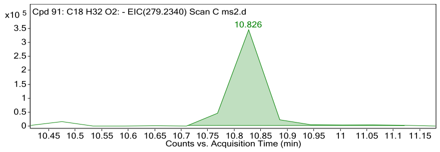

MS Spectrum

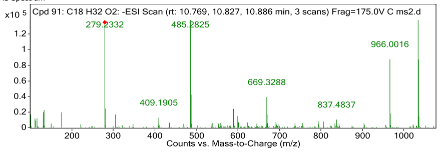

MS Zoomed Spectrum

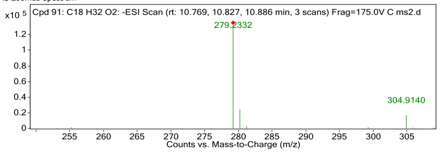

MSMS Spectrum

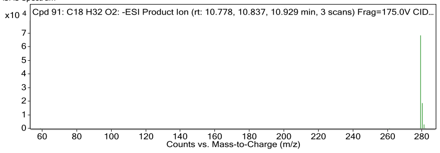

MSMS Spectrum

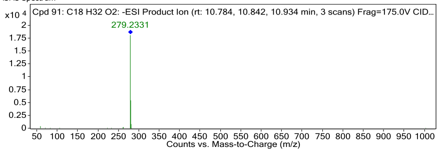

MSMS Spectrum

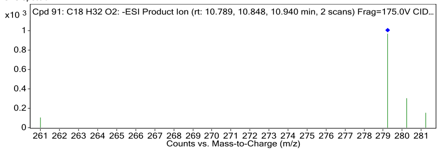

Compound Label	Name	*m/z*	RT	Algorithm	Mass
Cpd 92: C54 H80 O11		903.5639	10.876	Auto MS/MS	904.5703

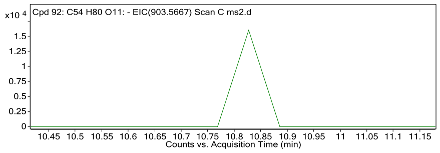

MS Spectrum

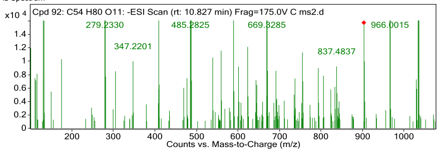

MS Zoomed Spectrum

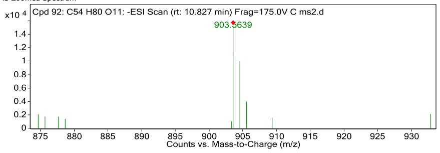

MSMS Spectrum

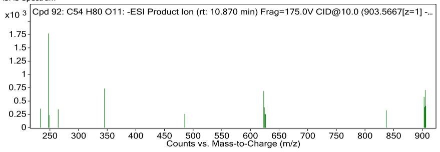

MSMS Spectrum

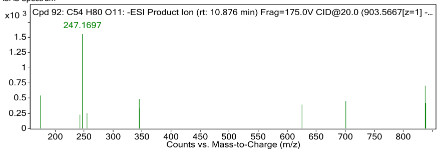

MSMS Spectrum

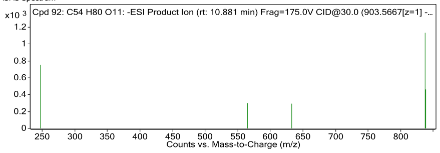

Compound Label	Name	*m/z*	RT	Algorithm	Mass
Cpd 93: C35 H52 O8		599.3591	10.939	Auto MS/MS	600.36629

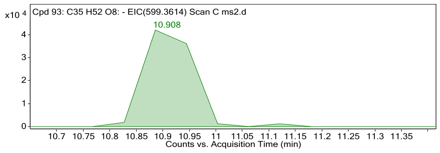

MS Spectrum

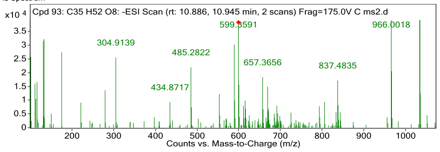

MS Zoomed Spectrum

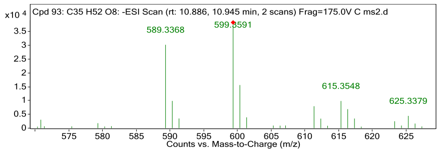

MSMS Spectrum

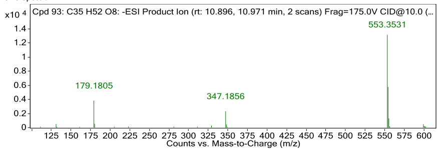

MSMS Spectrum

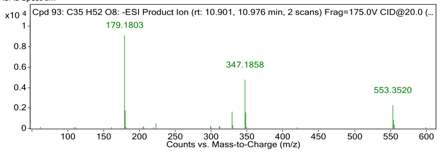

MSMS Spectrum

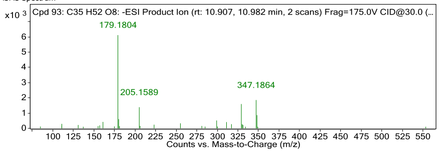

Compound Label	Name	*m/z*	RT	Algorithm	Mass
Cpd 94: C37 H54 O10		657.3653	11.027	Auto MS/MS	658.37243

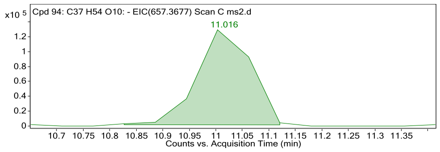

MS Spectrum

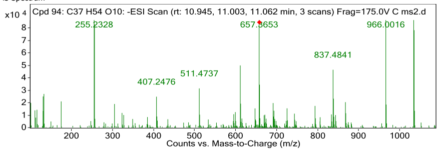

MS Zoomed Spectrum

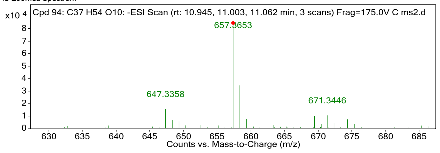

MSMS Spectrum

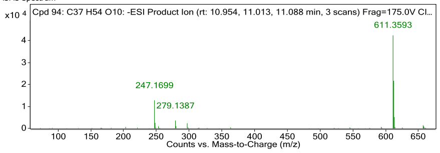

MSMS Spectrum

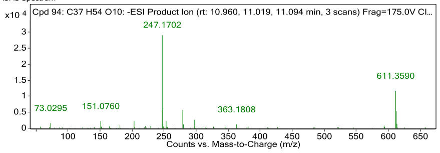

MSMS Spectrum

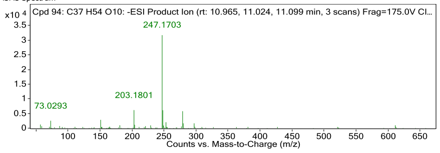

Compound Label	Name	*m/z*	RT	Algorithm	Mass
Cpd 95: C36 H52 O8		611.3594	11.052	Auto MS/MS	612.36656

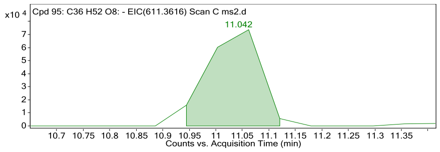

MS Spectrum

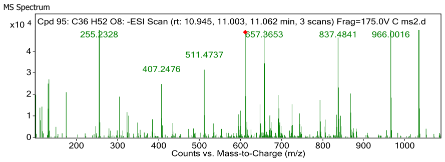

MS Zoomed Spectrum

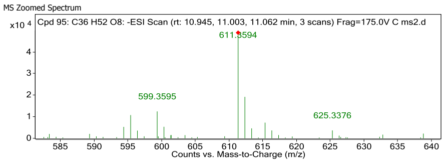

MSMS Spectrum

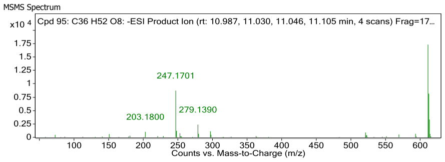

MSMS Spectrum

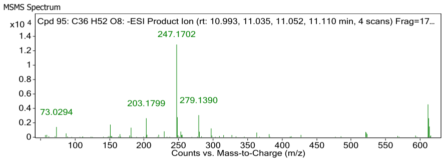

MSMS Spectrum

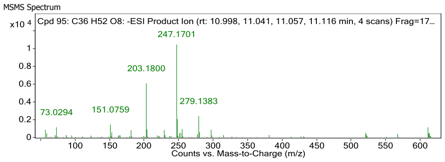

Compound Label	Name	*m/z*	RT	Algorithm	Mass
Cpd 96: C16 H32 O2		255.2336	11.107	Auto MS/MS	256.24069

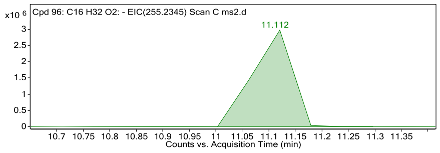

MS Spectrum

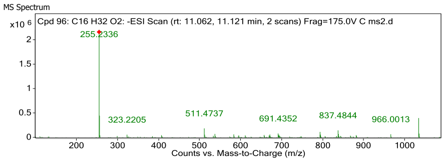

MS Zoomed Spectrum

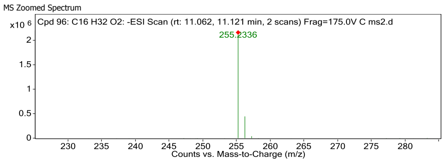

MSMS Spectrum

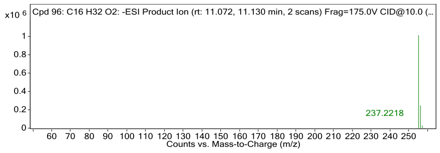

MSMS Spectrum

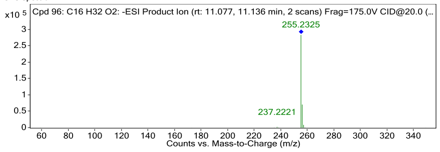

MSMS Spectrum

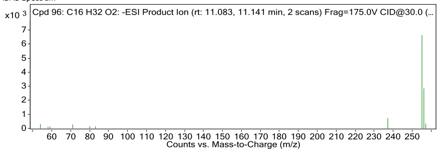

Compound Label	Name	*m/z*	RT	Algorithm	Mass
Cpd 97: C50 H82 O7		793.5997	11.152	Auto MS/MS	794.60708

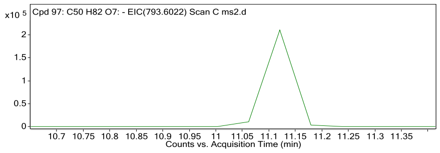

MS Spectrum

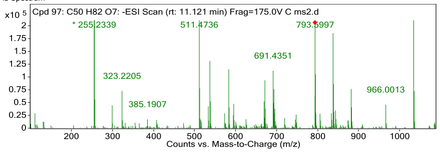

MS Zoomed Spectrum

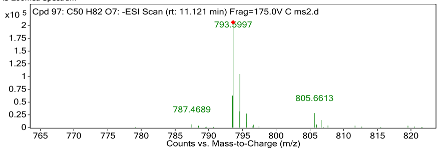

MSMS Spectrum

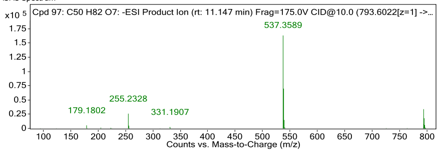

MSMS Spectrum

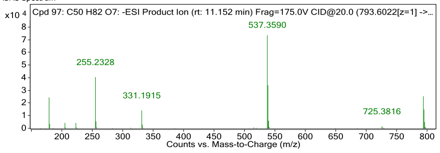

MSMS Spectrum

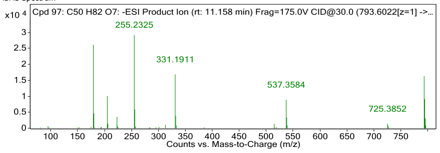

Compound Label	Name	*m/z*	RT	Algorithm	Mass
Cpd 98: C34 H50 O5		537.3592	11.169	Auto MS/MS	538.3665

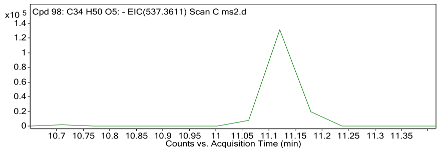

MS Spectrum

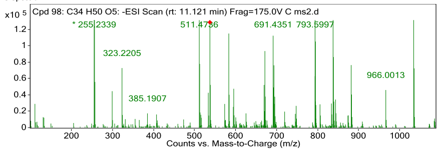

MS Zoomed Spectrum

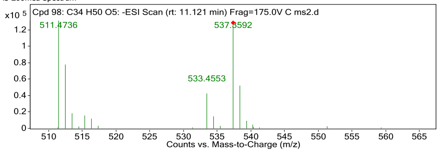

MSMS Spectrum

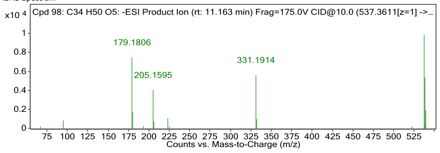

MSMS Spectrum

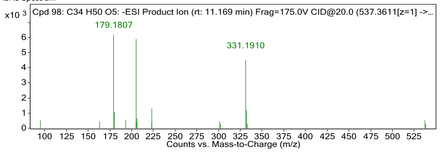

MSMS Spectrum

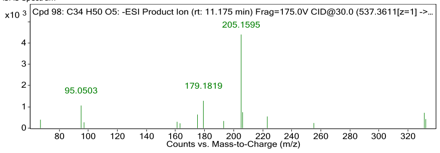

Compound Label	Name	*m/z*	RT	Algorithm	Mass
Cpd 99: C31 H40 O3		459.2904	11.224	Auto MS/MS	460.2977

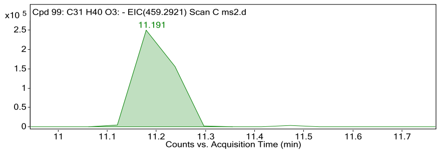

MS Spectrum

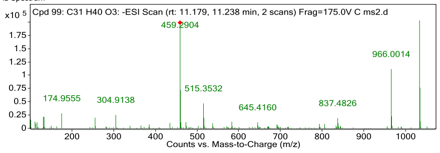

MS Zoomed Spectrum

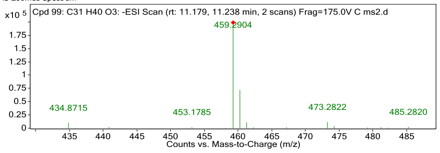

MSMS Spectrum

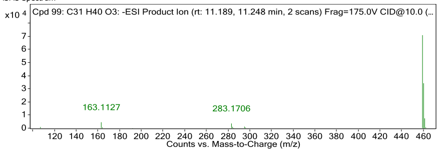

MSMS Spectrum

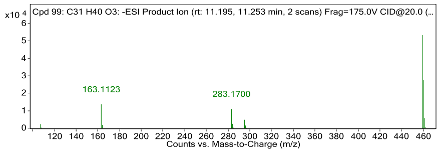

MSMS Spectrum

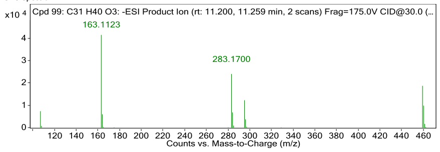

Compound Label	Name	*m/z*	RT	Algorithm	Mass
Cpd 100: C35 H48 O3		515.3531	11.307	Auto MS/MS	516.36044

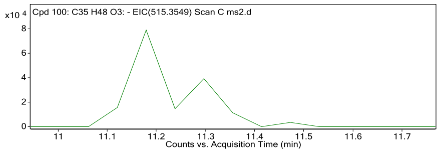

MS Spectrum

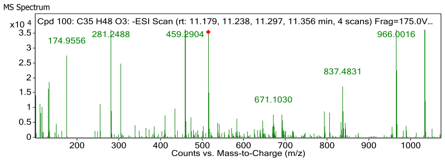

MS Zoomed Spectrum

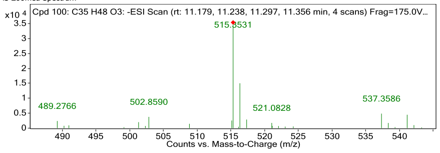

MSMS Spectrum

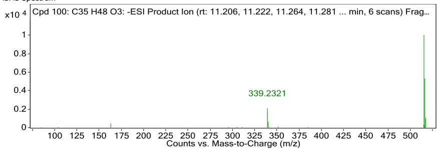

MSMS Spectrum

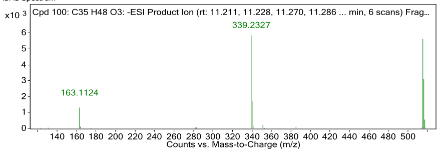

MSMS Spectrum

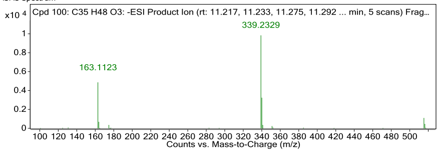

Compound Label	Name	*m/z*	RT	Algorithm	Mass
Cpd 101: C18 H34 O2		281.2489	11.417	Auto MS/MS	282.25641

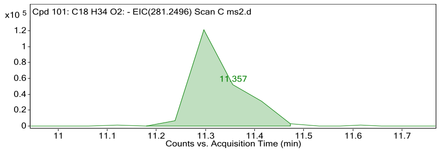

MS Spectrum

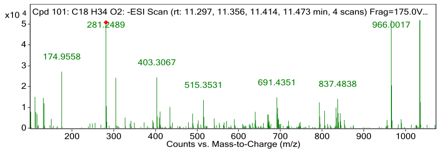

MS Zoomed Spectrum

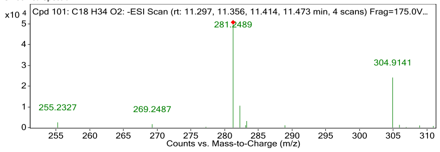

MSMS Spectrum

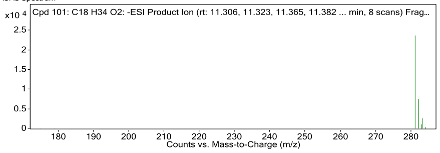

MSMS Spectrum

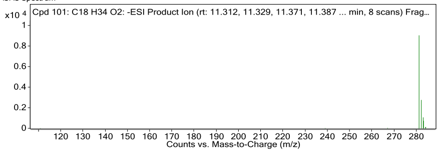

MSMS Spectrum

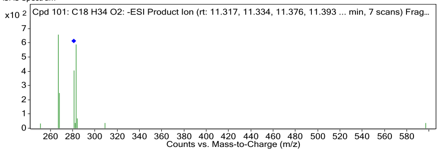

Compound Label	Name	*m/z*	RT	Algorithm	Mass
Cpd 102: C22 H44 O6		403.3067	11.459	Auto MS/MS	404.31403

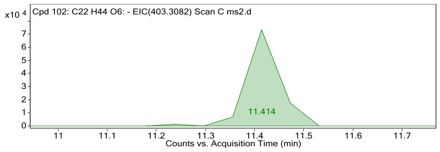

MS Spectrum

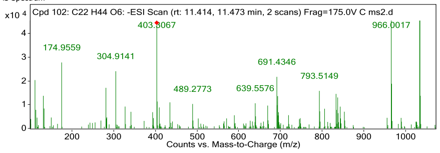

MS Zoomed Spectrum

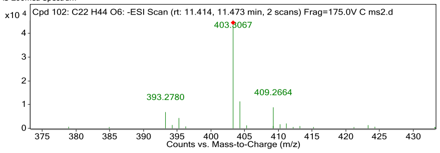

MSMS Spectrum

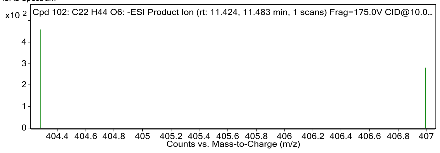

MSMS Spectrum

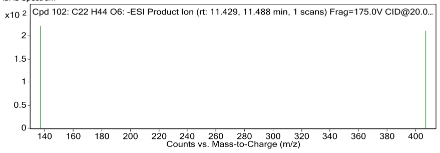

MSMS Spectrum

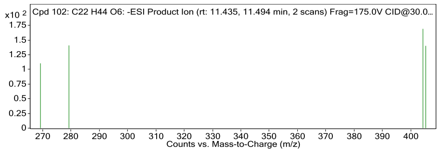

Compound Label	Name	*m/z*	RT	Algorithm	Mass
Cpd 103: C29 H44 O2		423.3265	11.61	Auto MS/MS	424.33398

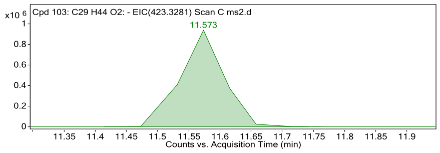

MS Spectrum

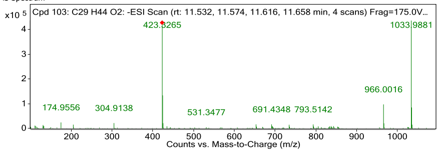

MS Zoomed Spectrum

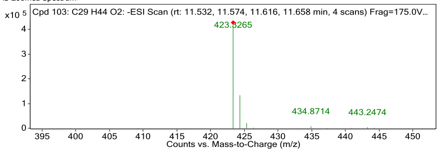

MSMS Spectrum

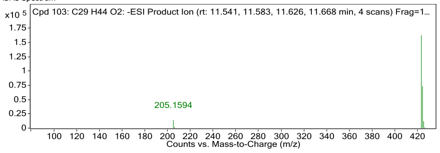

MSMS Spectrum

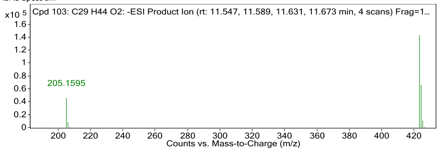

MSMS Spectrum

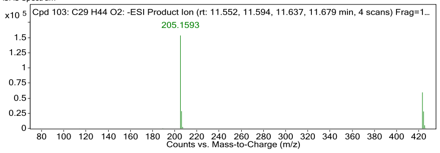

Compound Label	Name	*m/z*	RT	Algorithm	Mass
Cpd 104: C37 H50 O10		653.3339	11.635	Auto MS/MS	654.34138

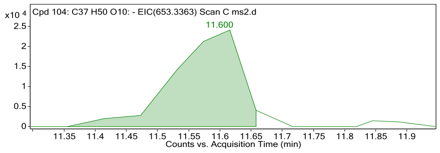

MS Spectrum

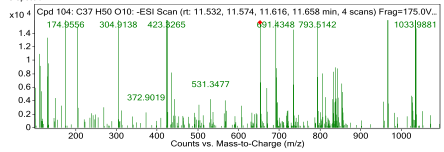

MS Zoomed Spectrum

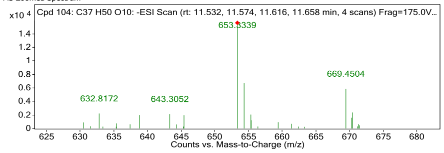

MSMS Spectrum

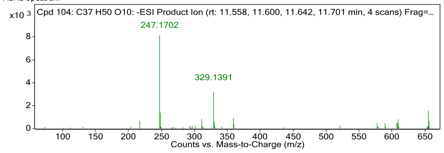

MSMS Spectrum

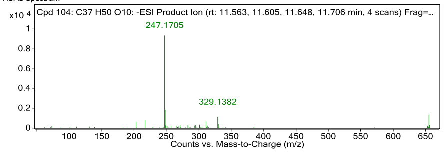

MSMS Spectrum

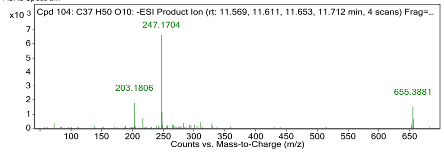

Compound Label	Name	*m/z*	RT	Algorithm	Mass
Cpd 105: C45 H68 O7		719.487	11.922	Auto MS/MS	720.49444

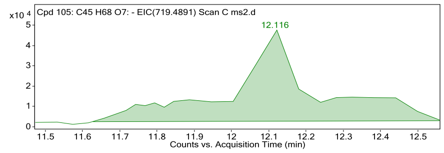

MS Spectrum

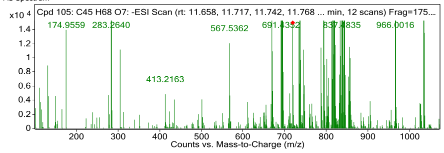

MS Zoomed Spectrum

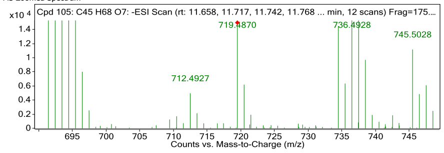

MSMS Spectrum

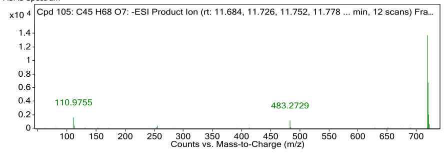

MSMS Spectrum

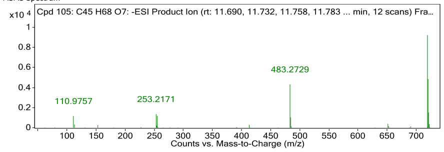

MSMS Spectrum

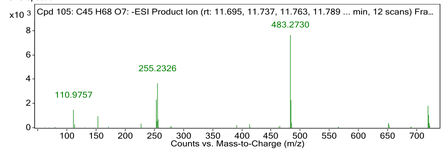

Compound Label	Name	*m/z*	RT	Algorithm	Mass
Cpd 106: C34 H46 O3		501.3375	11.94	Auto MS/MS	502.34491

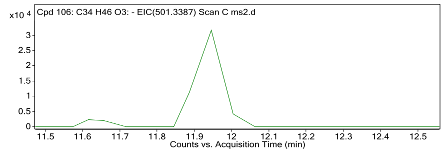

MS Spectrum

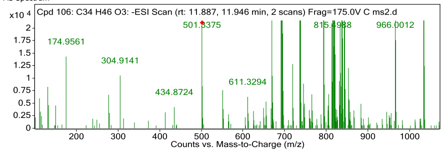

MS Zoomed Spectrum

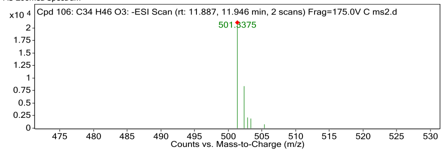

MSMS Spectrum

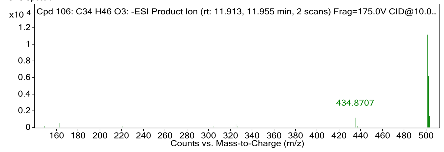

MSMS Spectrum

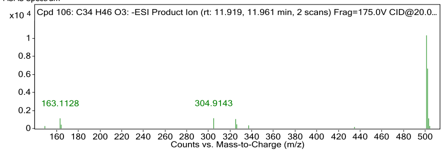

MSMS Spectrum

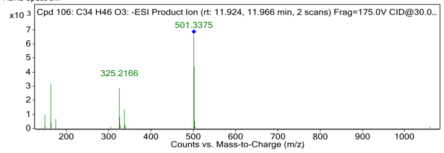

Compound Label	Name	*m/z*	RT	Algorithm	Mass
Cpd 107: C37 H52 O10		655.349	12.019	Auto MS/MS	656.35639

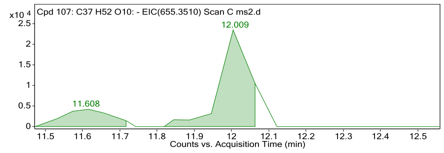

MS Spectrum

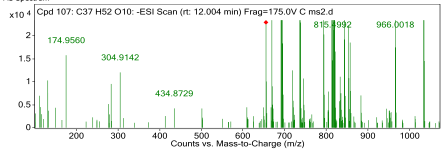

MS Zoomed Spectrum

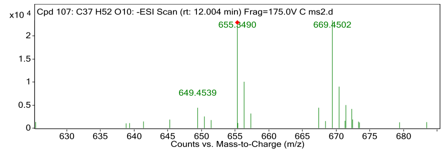

MSMS Spectrum

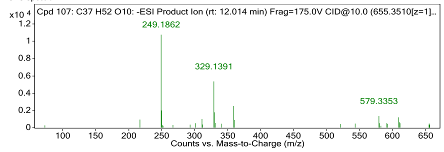

MSMS Spectrum

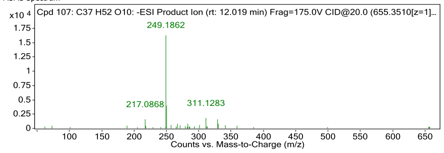

MSMS Spectrum

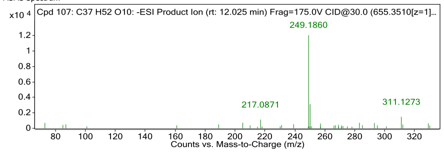

Compound Label	Name	*m/z*	RT	Algorithm	Mass
Cpd 108: 12.119		551.3078	12.119	Auto MS/MS	

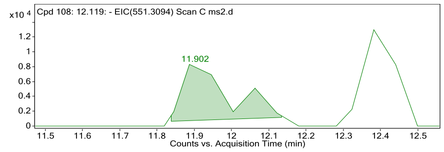

MS Spectrum

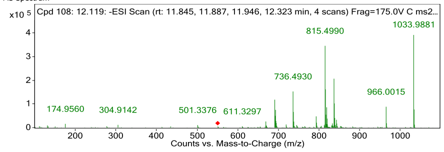

MS Zoomed Spectrum

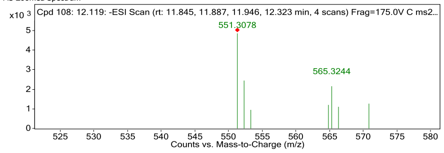

MSMS Spectrum

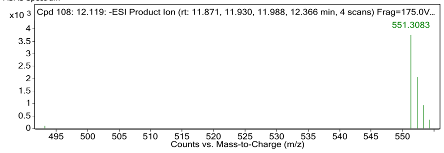

MSMS Spectrum

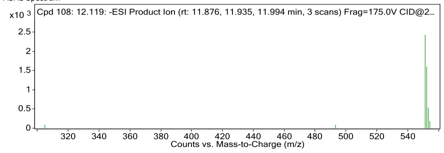

MSMS Spectrum

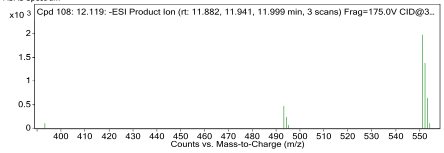

Compound Label	Name	*m/z*	RT	Algorithm	Mass
Cpd 109: C18 H36 O2		283.264	12.162	Auto MS/MS	284.2714

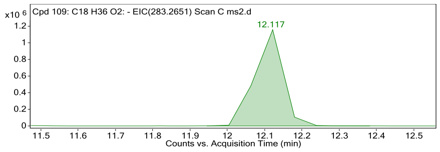

MS Spectrum

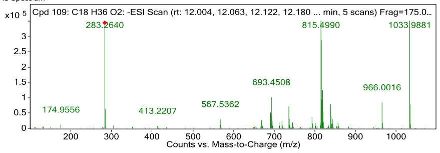

MS Zoomed Spectrum

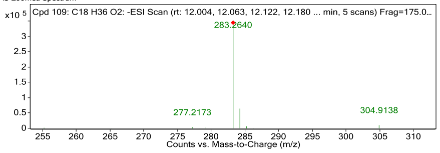

MSMS Spectrum

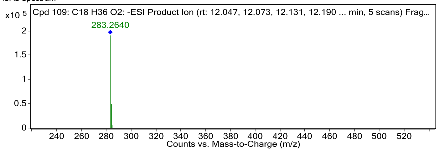

MSMS Spectrum

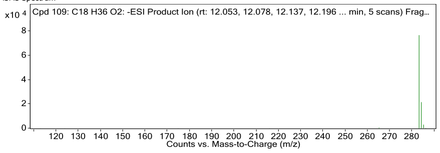

MSMS Spectrum

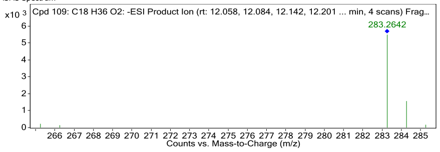

Compound Label	Name	*m/z*	RT	Algorithm	Mass
Cpd 110: C38 H68 O8		651.4871	12.17	Auto MS/MS	652.4941

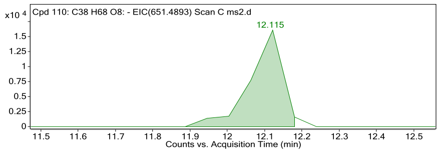

MS Spectrum

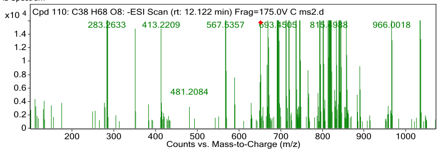

MS Zoomed Spectrum

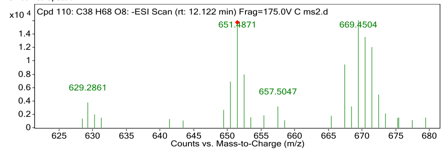

MSMS Spectrum

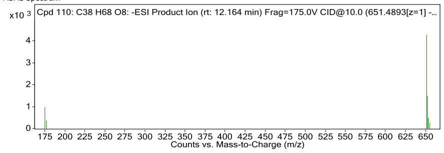

MSMS Spectrum

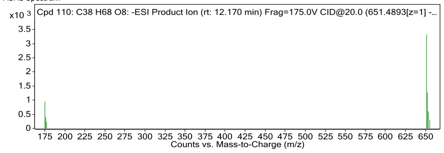

MSMS Spectrum

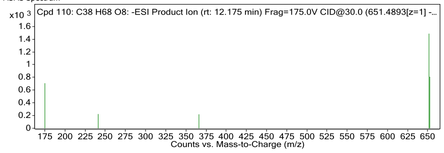

Compound Label	Name	m/z	RT	Algorithm	Mass
Cpd 111: 12.170		413.2213	12.17	Auto MS/MS	

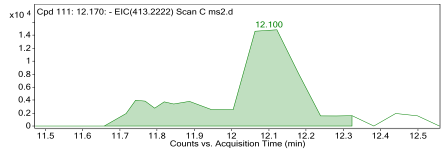

MS Spectrum

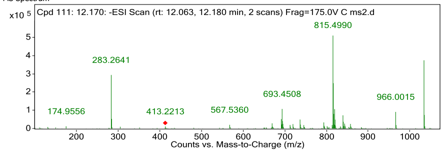

MS Zoomed Spectrum

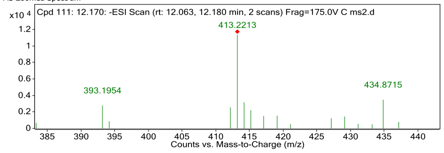

MSMS Spectrum

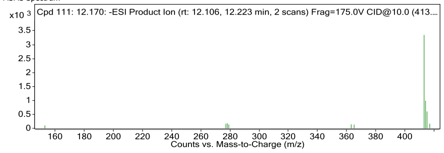

MSMS Spectrum

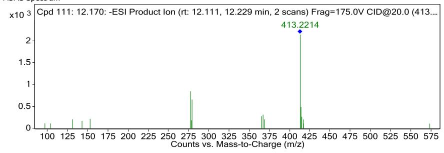

MSMS Spectrum

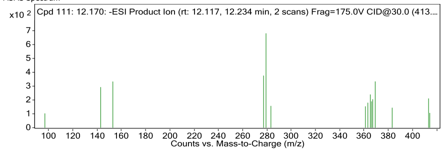

Compound Label	Name	*m/z*	RT	Algorithm	Mass
Cpd 112: C45 H68 O7		719.4873	12.38	Auto MS/MS	720.49453

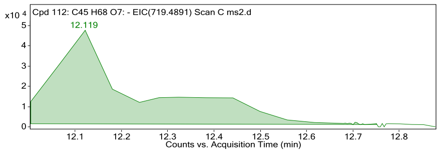

MS Spectrum

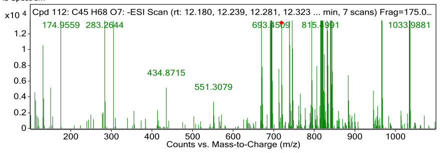

MS Zoomed Spectrum

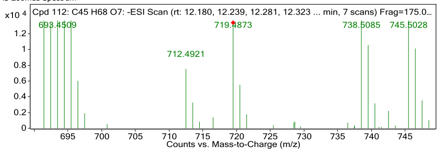

MSMS Spectrum

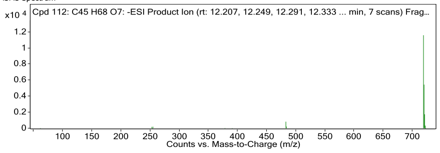

MSMS Spectrum

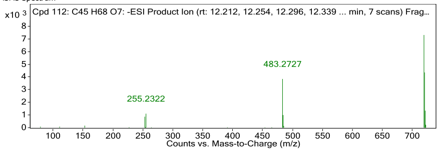

MSMS Spectrum

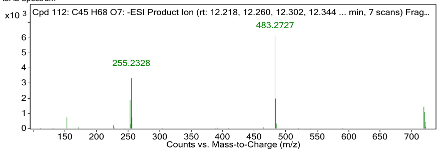

Compound Label	Name	*m/z*	RT	Algorithm	Mass
Cpd 113: 12.433		551.3079	12.433	Auto MS/MS	

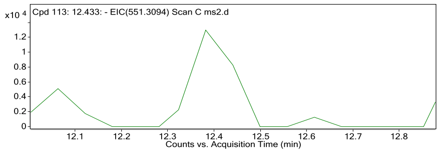

MS Spectrum

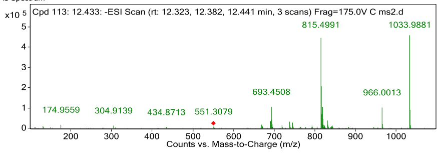

MS Zoomed Spectrum

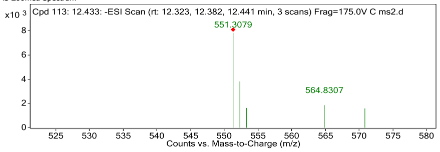

MSMS Spectrum

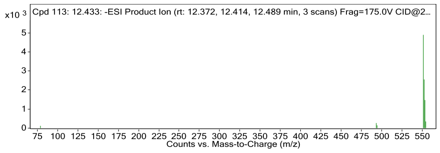

MSMS Spectrum

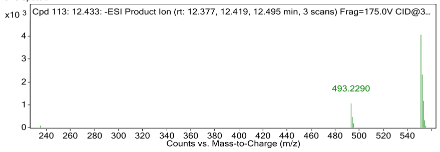

MSMS Spectrum

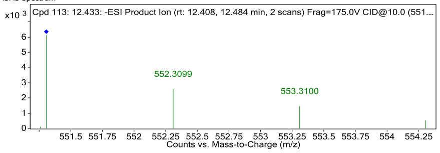

Compound Label	Name	*m/z*	RT	Algorithm	Mass
Cpd 114: C49 H77 O10		824.5455	12.473	Auto MS/MS	825.55411

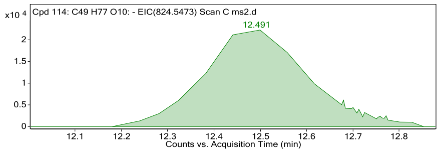

MS Spectrum

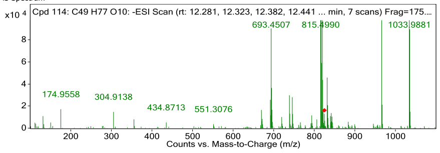

MS Zoomed Spectrum

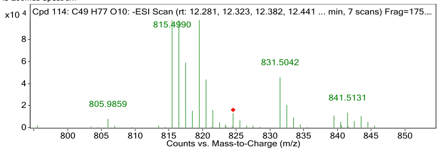

MSMS Spectrum

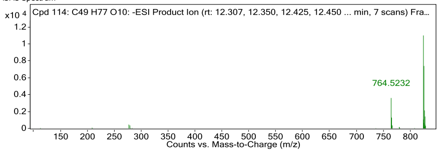

MSMS Spectrum

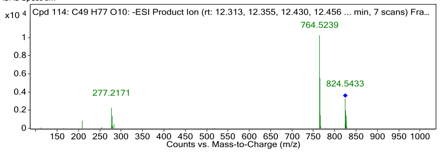

MSMS Spectrum

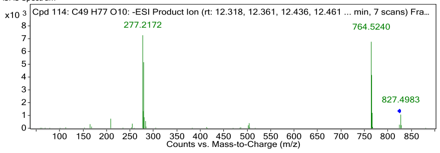

Compound Label	Name	*m/z*	RT	Algorithm	Mass
Cpd 115: C23 H32 O3		355.2275	12.59	Auto MS/MS	356.23492

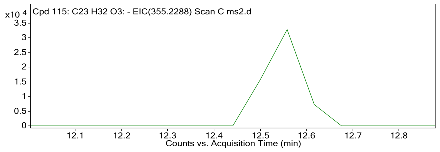

MS Spectrum

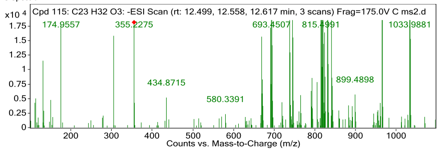

MS Zoomed Spectrum

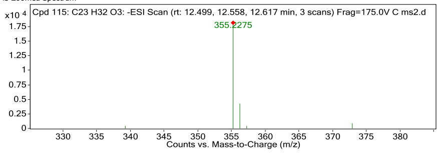

MSMS Spectrum

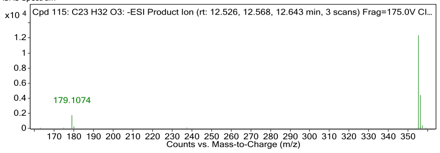

MSMS Spectrum

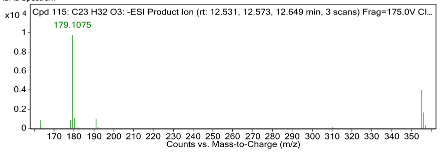

MSMS Spectrum

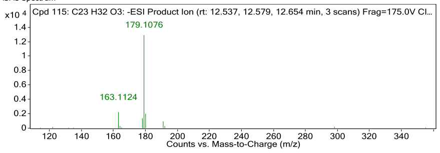

Compound Label	Name	*m/z*	RT	Algorithm	Mass
Cpd 116: 12.674		892.5337	12.674	Auto MS/MS	

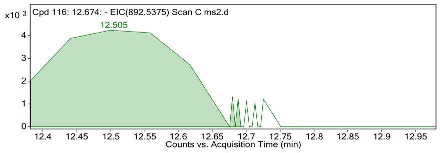

MS Spectrum

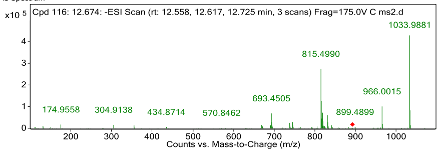

MS Zoomed Spectrum

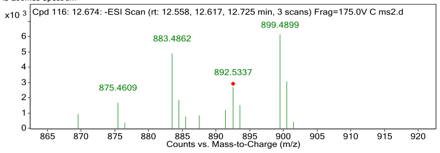

MSMS Spectrum

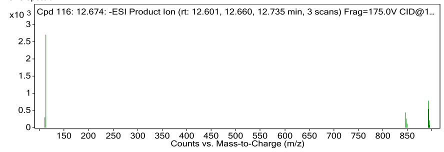

MSMS Spectrum

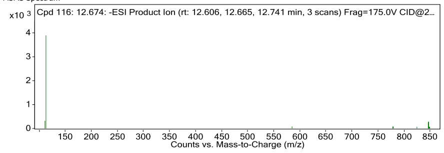

MSMS Spectrum

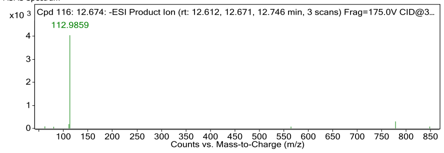

Compound Label	Name	*m/z*	RT	Algorithm	Mass
Cpd 117: 12.964		463.3101	12.964	Auto MS/MS	

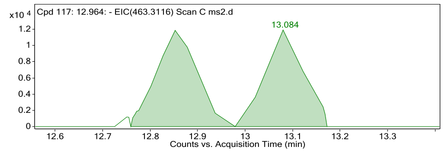

MS Spectrum

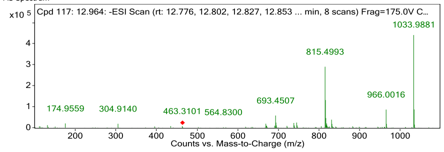

MS Zoomed Spectrum

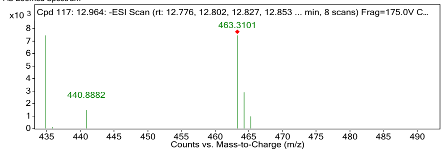

MSMS Spectrum

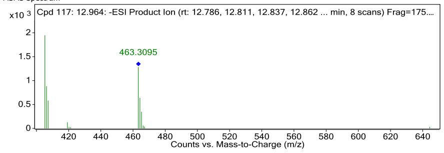

MSMS Spectrum

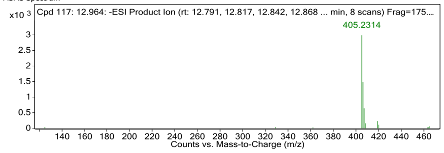

MSMS Spectrum

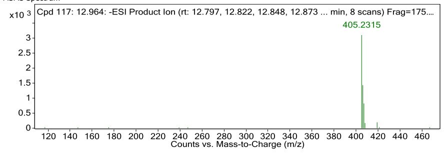

Compound Label	Name	*m/z*	RT	Algorithm	Mass
Cpd 118: 12.990		913.5835	12.99	Auto MS/MS	

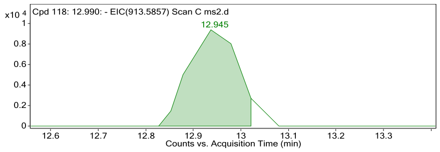

MS Spectrum

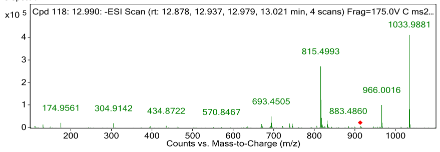

MS Zoomed Spectrum

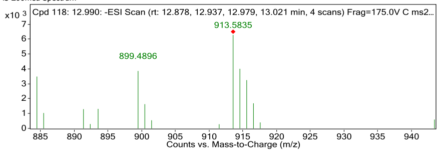

MSMS Spectrum

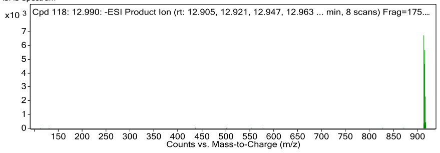

MSMS Spectrum

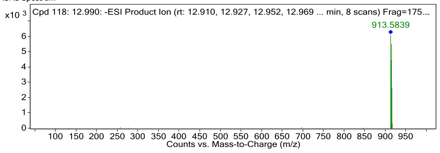

MSMS Spectrum

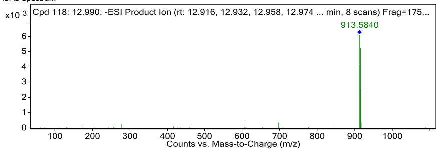

Compound Label	Name	*m/z*	RT	Algorithm	Mass
Cpd 119: C56 H84 O10		915.5989	13.125	Auto MS/MS	916.60611

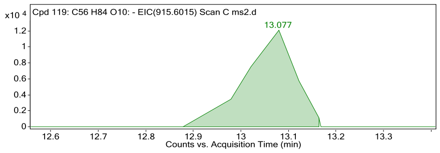

MS Spectrum

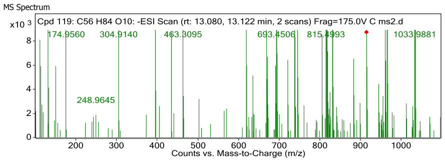

MS Zoomed Spectrum

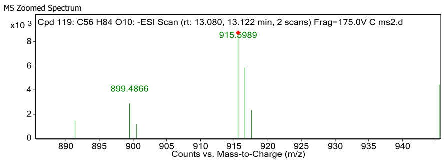

MSMS Spectrum

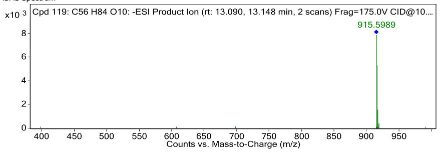

MSMS Spectrum

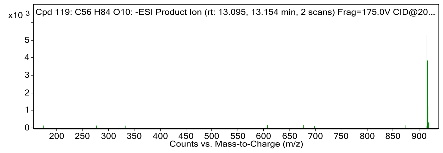

MSMS Spectrum

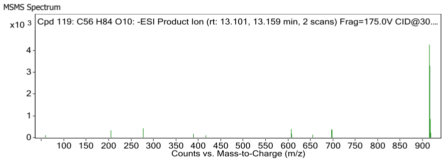

--- End Of Report ---
