# Supplementary material for: In planta high levels of hydrolysable tannins inhibit peroxidase mediated anthocyanin degradation and maintain abaxially red leaves of Excoecaria Cochinchinensis
Source: BMC Plant Biol. 2019 Jul 15;19:315. doi: 10.1186/s12870-019-1903-y (PMC6632198; doi:10.1186/s12870-019-1903-y)
Supplement: Supplementary file 5 — Data file 2. MSMS compound report of Osmanthus phenolics used in this study. (PDF 6486 kb) [file 12870_2019_1903_MOESM5_ESM.pdf]

Qualitative Compound Report

**Data File**	neg ms2 sam03.d	**Sample Name**	Sample3
**Sample Type**	Sample	**Position**	P1-A3
**Instrument Name**	Instrument 1	**User Name**	
**Acq Method**	124sam3 neg ms2.m	**Acquired Time**	3/15/2018 12:20:11 PM
**IRM Calibration Status**	Success	**DA Method**	scau default.m
**Comment**			

**Sample Group**		**Info.**	
**Stream Name**	LC 1	**Acquisition SW Version**	6200 series TOF/6500 series Q-TOF B.06.01 (B6157)

Fragmentor Voltage 130 **Collision Energy** 0 **Ionization Mode** ESI

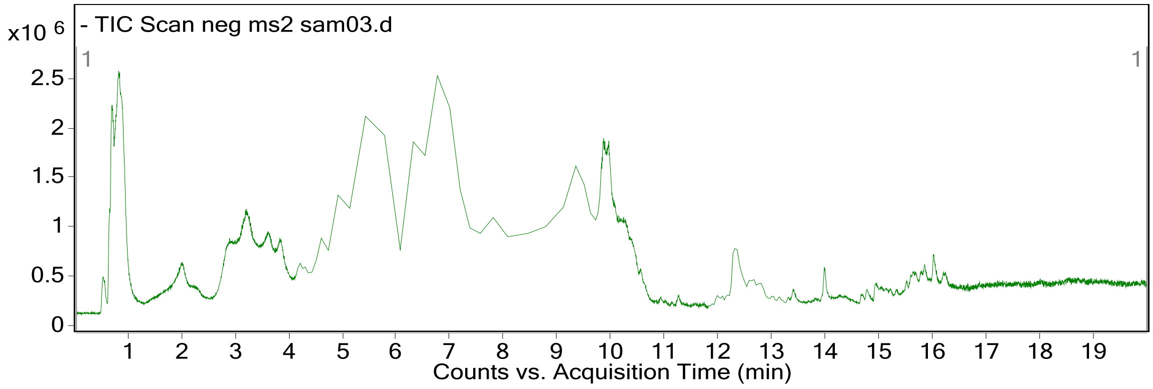

Fragmentor Voltage **Collision Energy** **Ionization Mode**

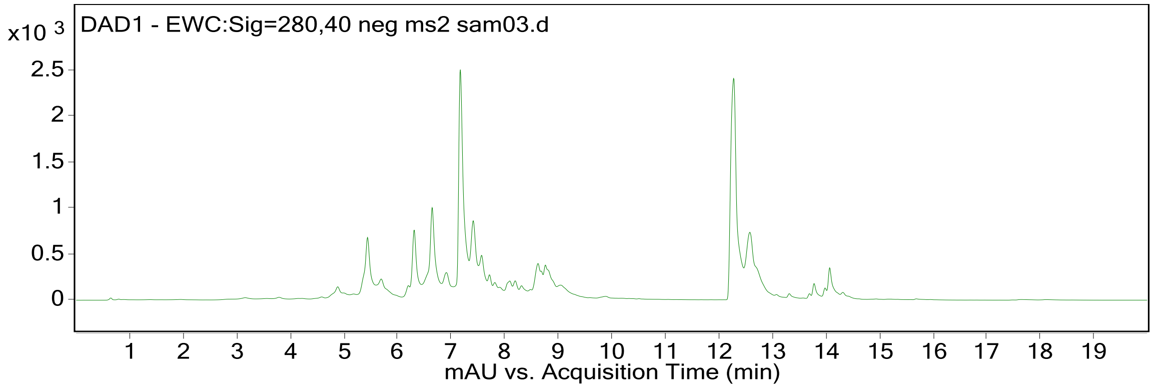

Compound Table

Compound Label	RT	SignificantIonMz
Cpd 1: 4.654	4.654	389.1089
Cpd 2: 4.773	4.773	715.2093
Cpd 3: 4.979	4.979	325.093
Cpd 4: 5.115	5.115	611.1766
Cpd 5: 5.197	5.197	955.294
Cpd 6: 5.235	5.235	403.1245
Cpd 7: 5.408	5.408	377.1454

Cpd 8: 5.540	5.54	755.2984
Cpd 9: 5.626	5.626	919.273
Cpd 10: 5.639	5.639	375.1298
Cpd 11: 5.676	5.676	191.0559
Cpd 12: 5.713	5.713	729.2254
Cpd 13: 5.726	5.726	305.07
Cpd 14: 5.775	5.775	461.1665
Cpd 15: 5.820	5.82	353.0877
Cpd 16: 5.994	5.994	891.2933
Cpd 17: 6.187	6.187	583.2034
Cpd 18: 6.418	6.418	191.0559
Cpd 19: 6.455	6.455	337.0933
Cpd 20: 6.698	6.698	705.204
Cpd 21: 6.723	6.723	703.1886
Cpd 22: 6.735	6.735	367.1034
Cpd 23: 7.159	7.159	735.2652
Cpd 24: 7.678	7.678	609.1464
Cpd 25: 7.909	7.909	607.2034
Cpd 26: 7.921	7.921	623.1988
Cpd 27: 7.995	7.995	377.1242
Cpd 28: 8.412	8.412	597.1832
Cpd 29: 8.449	8.449	539.1773
Cpd 30: 8.544	8.544	693.2038
Cpd 31: 8.606	8.606	591.2084
Cpd 32: 8.618	8.618	435.1296
Cpd 33: 8.668	8.668	377.1242
Cpd 34: 8.886	8.886	1009.3201
Cpd 35: 8.923	8.923	531.1509
Cpd 36: 8.936	8.936	487.1613
Cpd 37: 8.960	8.96	677.209
Cpd 38: 9.084	9.084	601.2141
Cpd 39: 9.154	9.154	579.2087
Cpd 40: 9.253	9.253	471.1874
Cpd 41: 9.303	9.303	523.1825
Cpd 42: 9.340	9.34	581.1884
Cpd 43: 9.397	9.397	361.1293
Cpd 44: 9.697	9.697	377.1242
Cpd 45: 12.010	12.01	219.1754
Cpd 46: 12.236	12.236	219.1754
Cpd 47: 12.321	12.321	487.3429
Cpd 48: 12.837	12.837	633.3801
Cpd 49: 13.147	13.147	633.3801

**Compound Label**	***m/z***	**RT**	**Algorithm**
Cpd 1: 4.654	389.1089	4.654	Targeted MS/MS

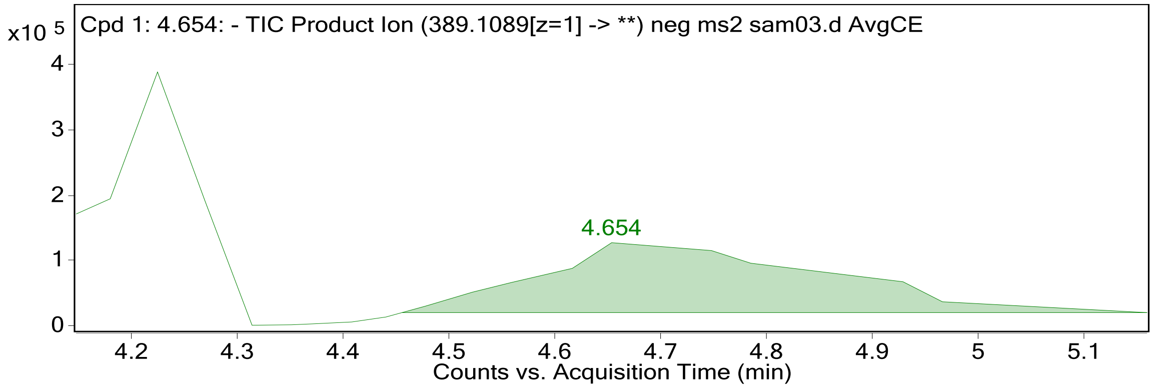

MSMS Spectrum

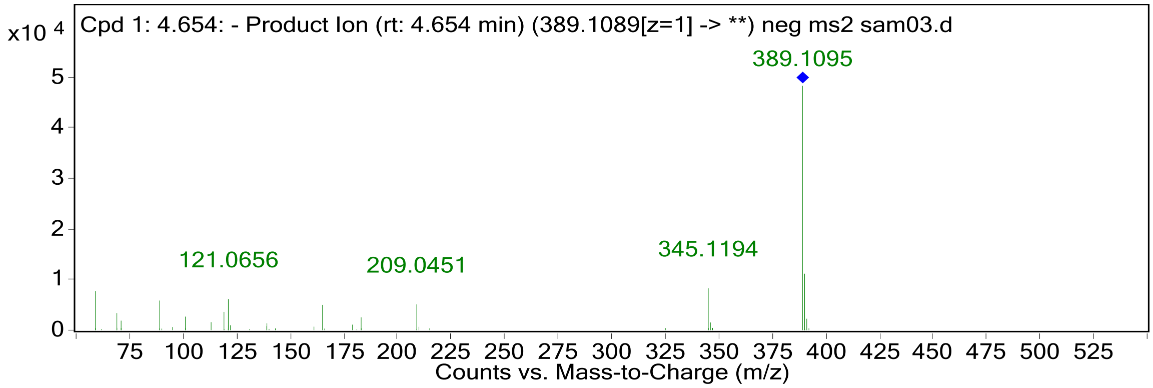

MSMS Spectrum

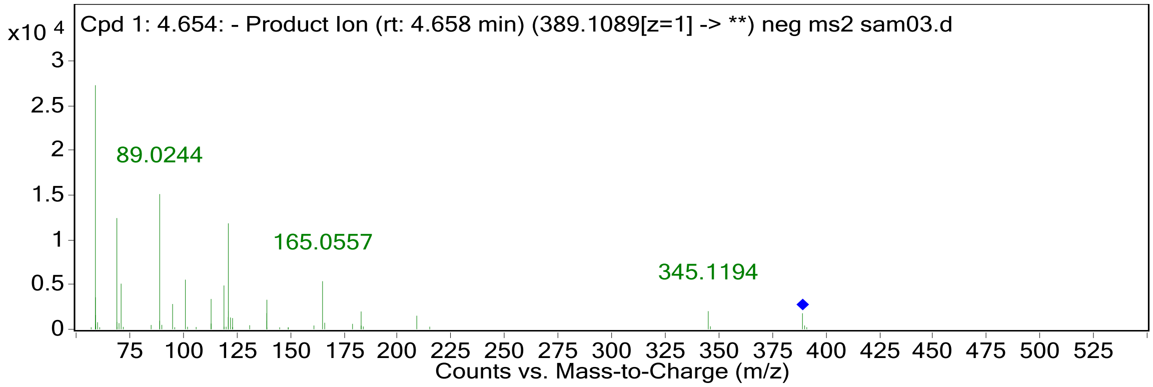

MSMS Spectrum

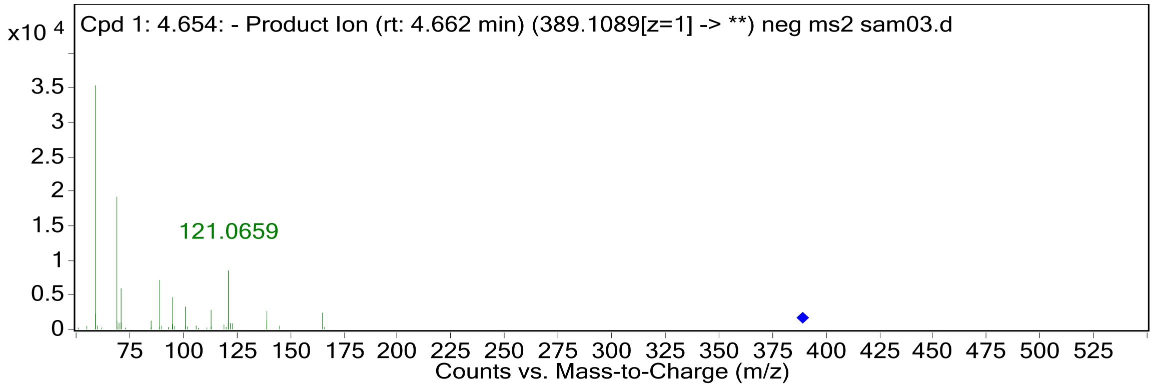

Qualitative Compound Report

Compound Label	*m/z*	RT	Algorithm
Cpd 2: 4.773	715.2093	4.773	Targeted MS/MS

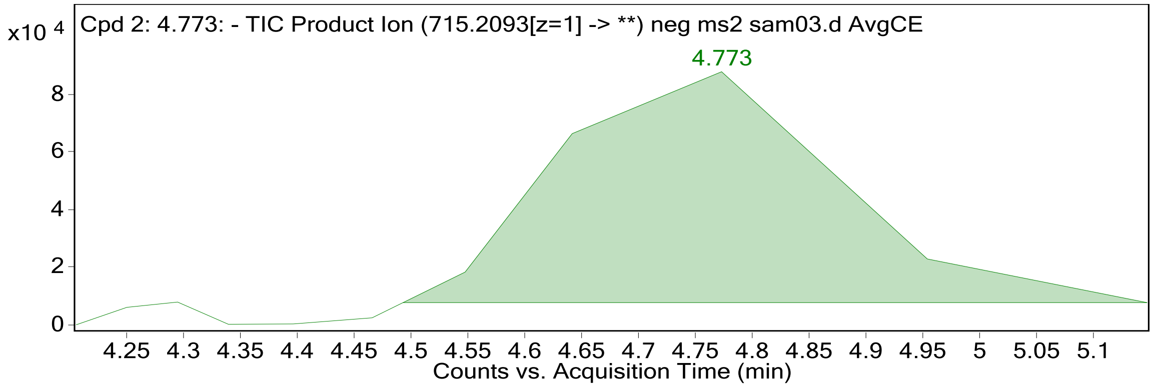

MSMS Spectrum

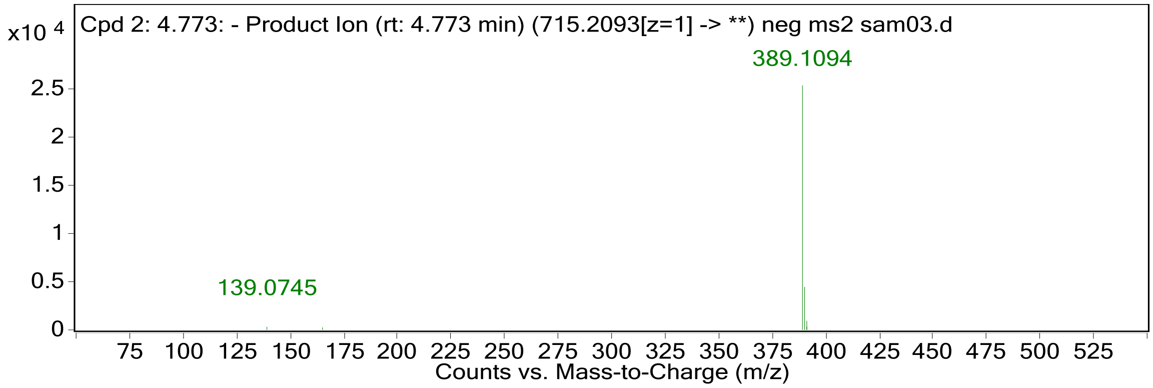

MSMS Spectrum

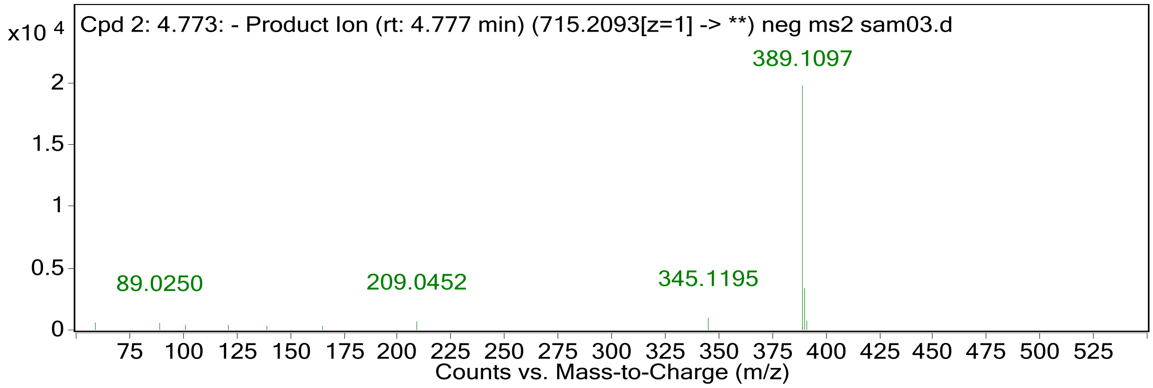

MSMS Spectrum

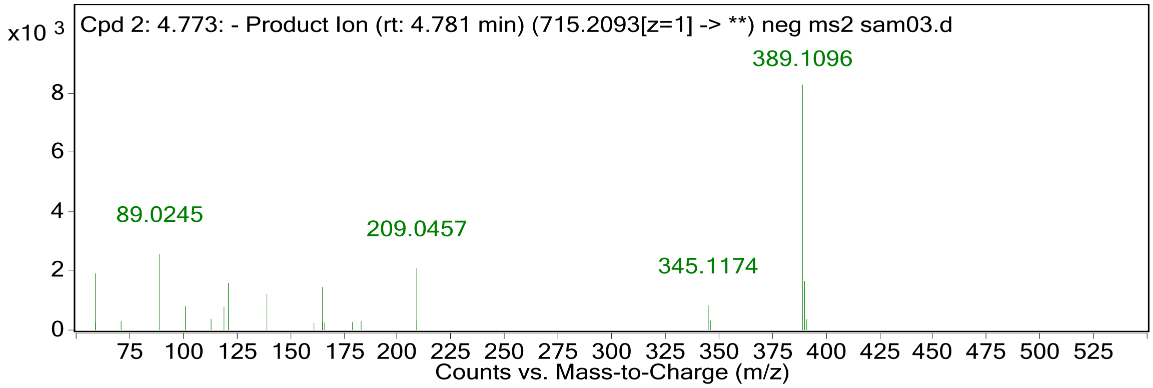

Compound Label	*m/z*	RT	Algorithm
Cpd 3: 4.979	325.093	4.979	Targeted MS/MS

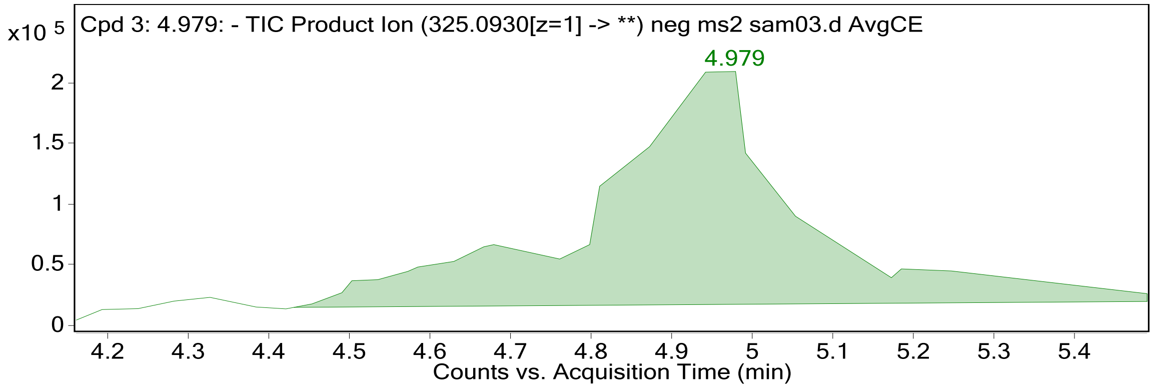

MSMS Spectrum

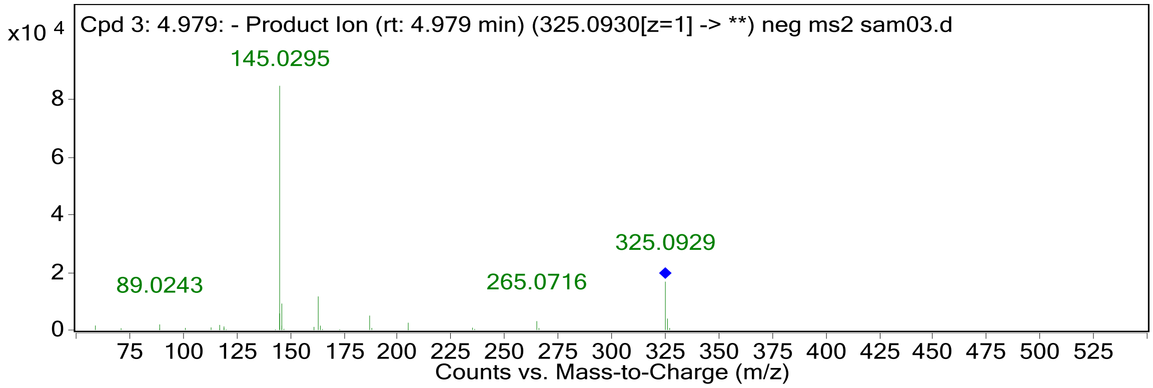

MSMS Spectrum

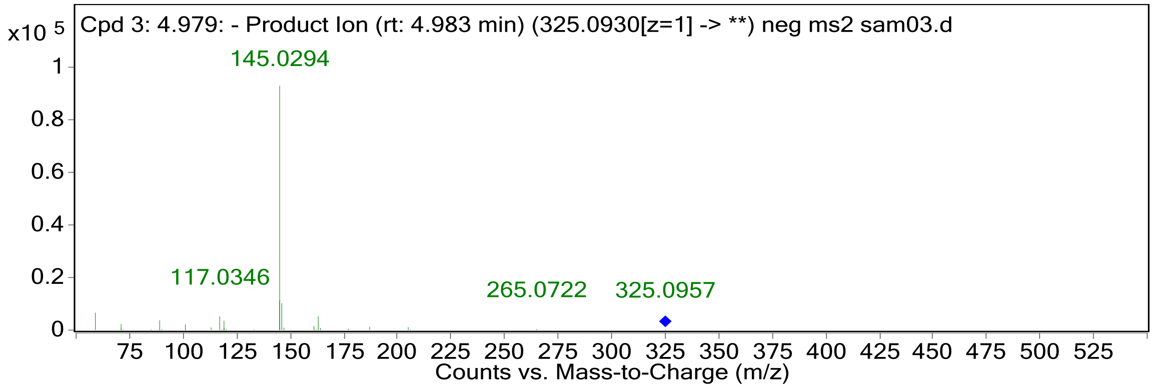

MSMS Spectrum

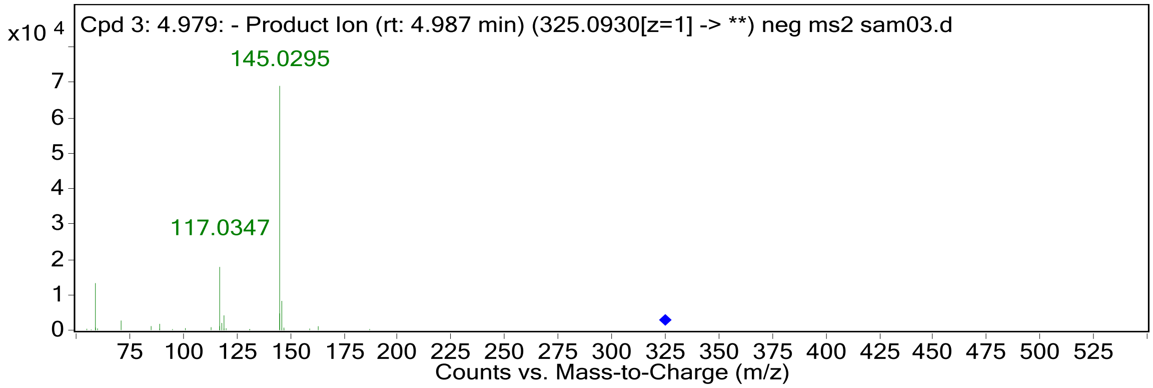

Compound Label	*m/z*	RT	Algorithm
Cpd 4: 5.115	611.1766	5.115	Targeted MS/MS

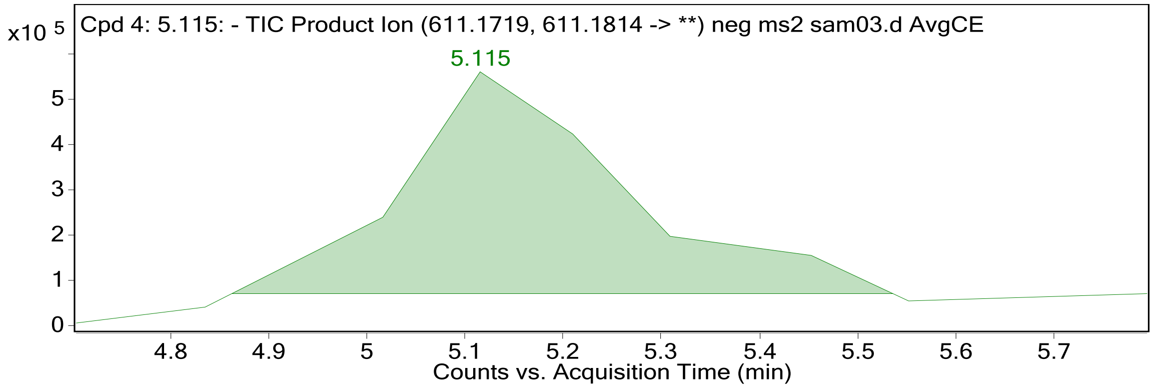

MSMS Spectrum

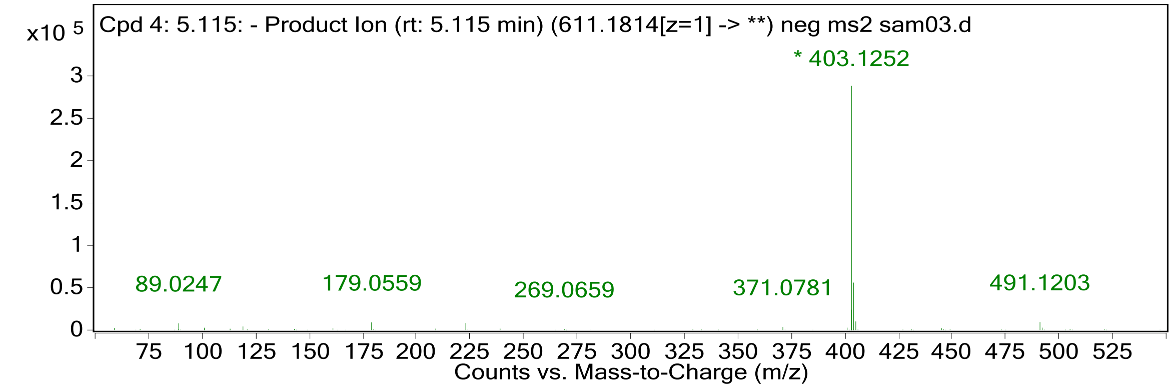

MSMS Spectrum

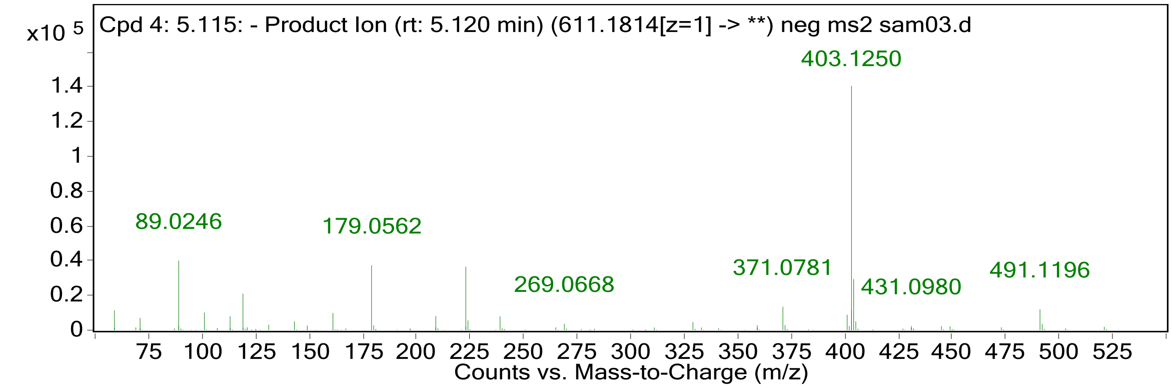

MSMS Spectrum

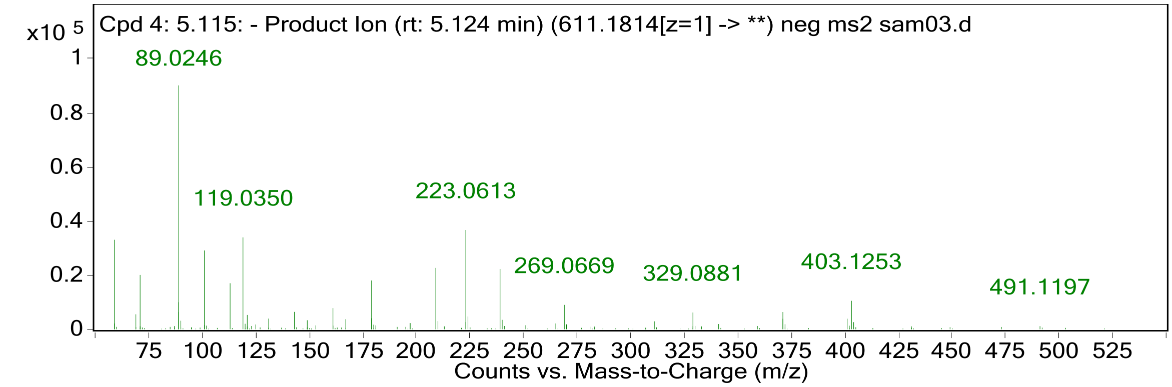

Compound Label	*m/z*	RT	Algorithm
Cpd 5: 5.197	955.294	5.197	Targeted MS/MS

Qualitative Compound Report

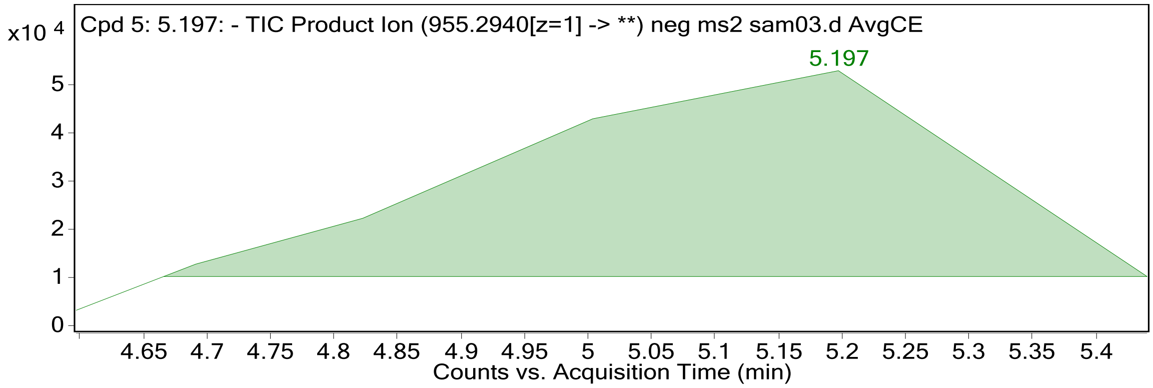

MSMS Spectrum

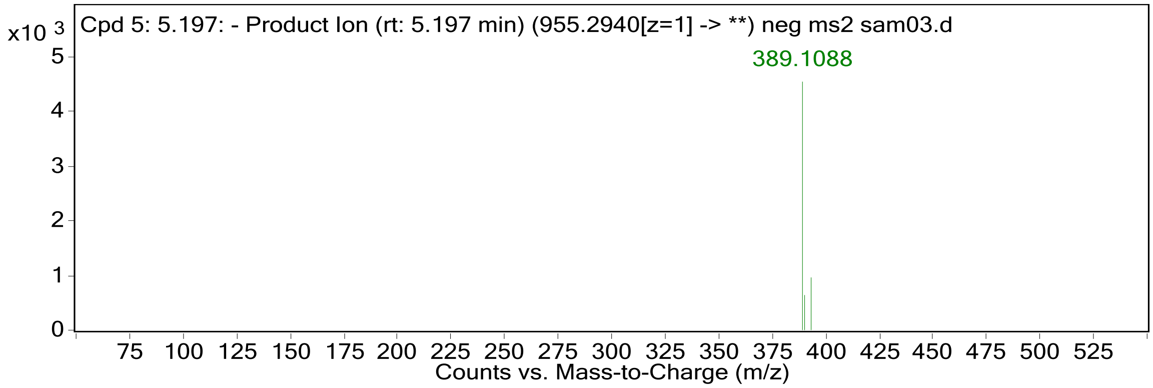

MSMS Spectrum

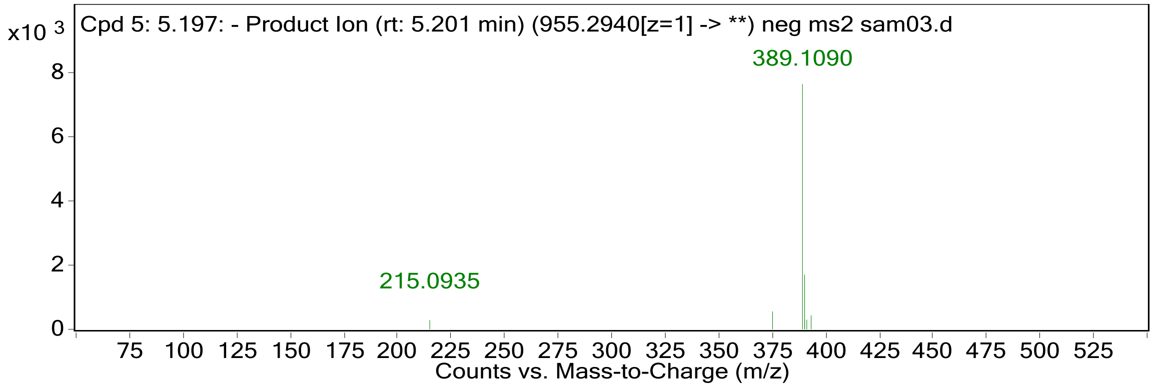

MSMS Spectrum

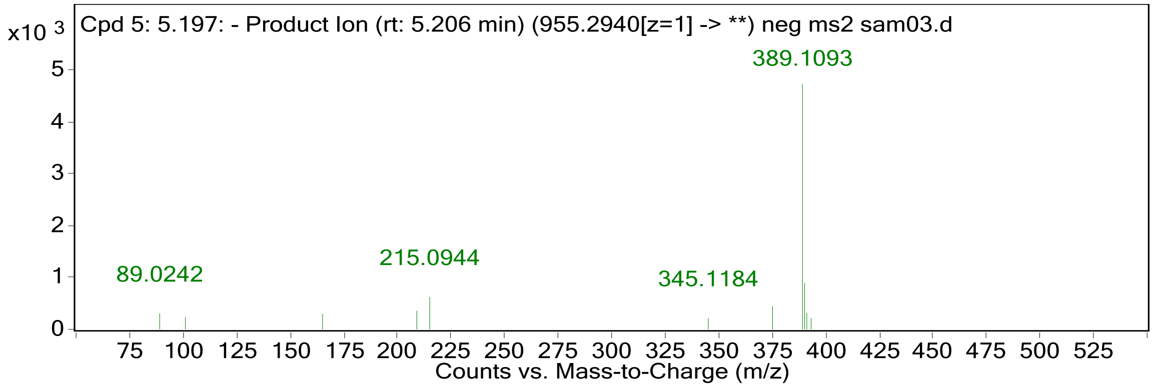

Compound Label	*m/z*	RT	Algorithm
Cpd 6: 5.235	403.1245	5.235	Targeted MS/MS

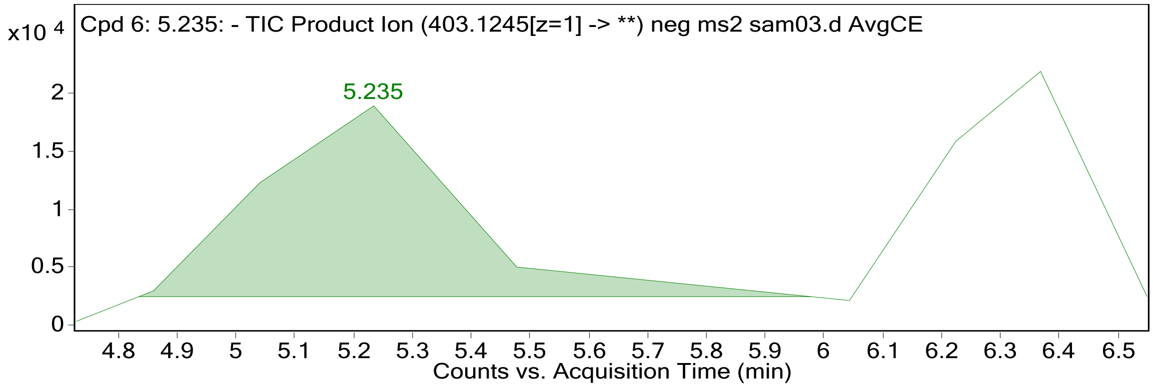

MSMS Spectrum

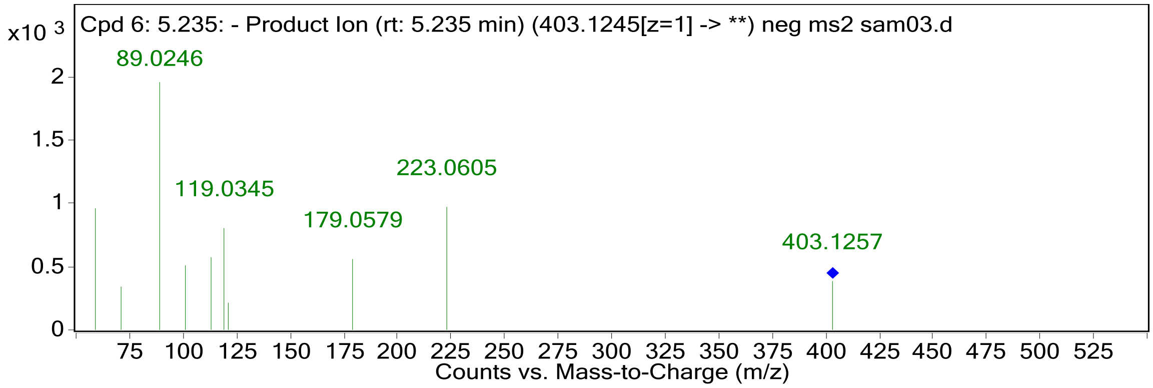

MSMS Spectrum

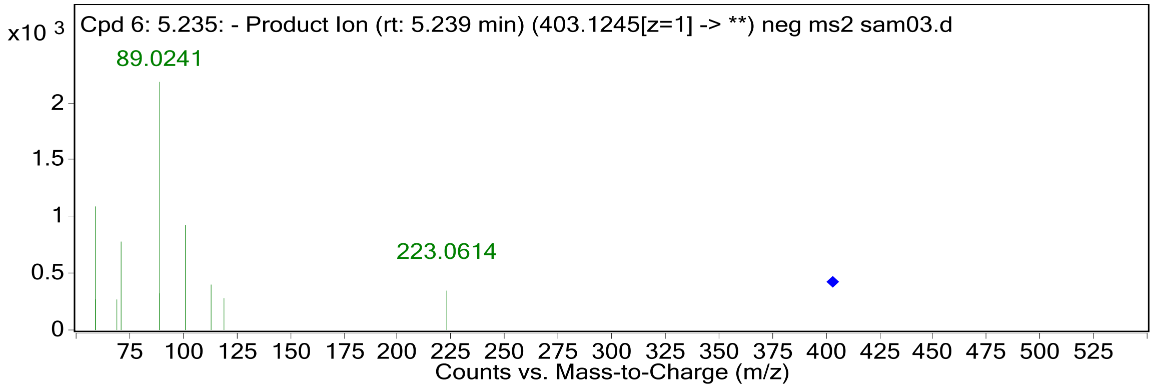

MSMS Spectrum

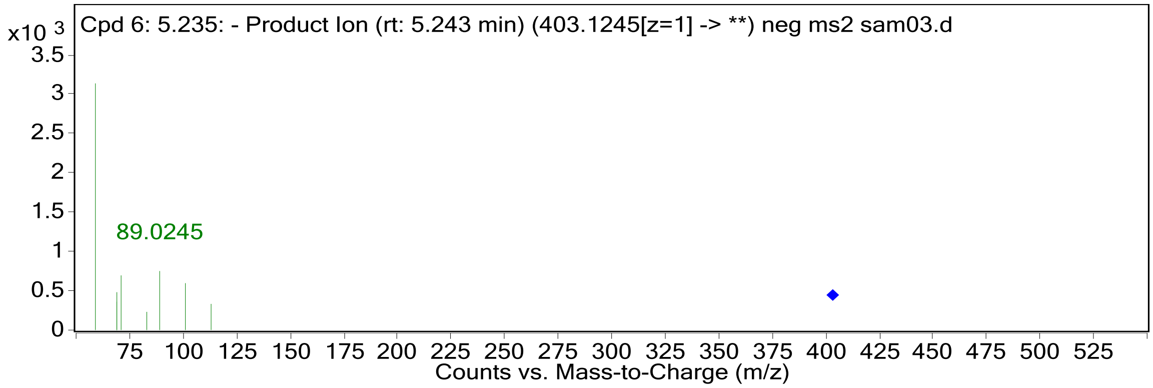

Compound Label	*m/z*	RT	Algorithm
Cpd 7: 5.408	377.1454	5.408	Targeted MS/MS

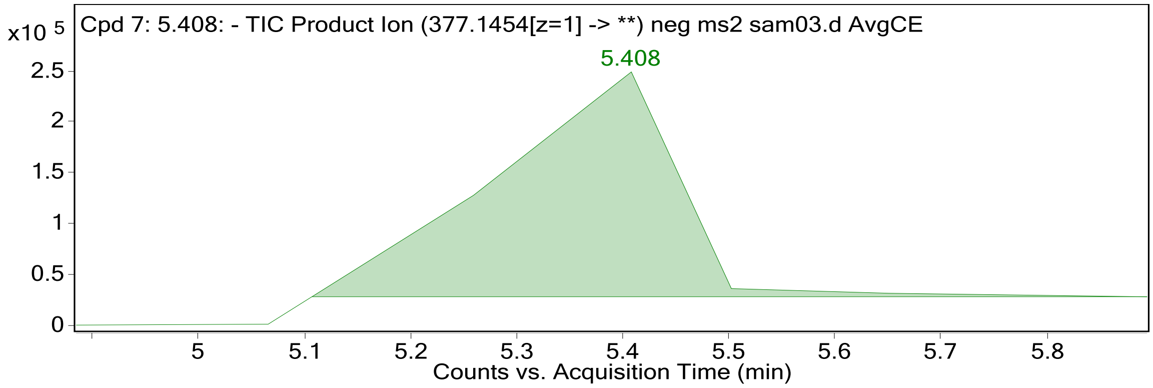

MSMS Spectrum

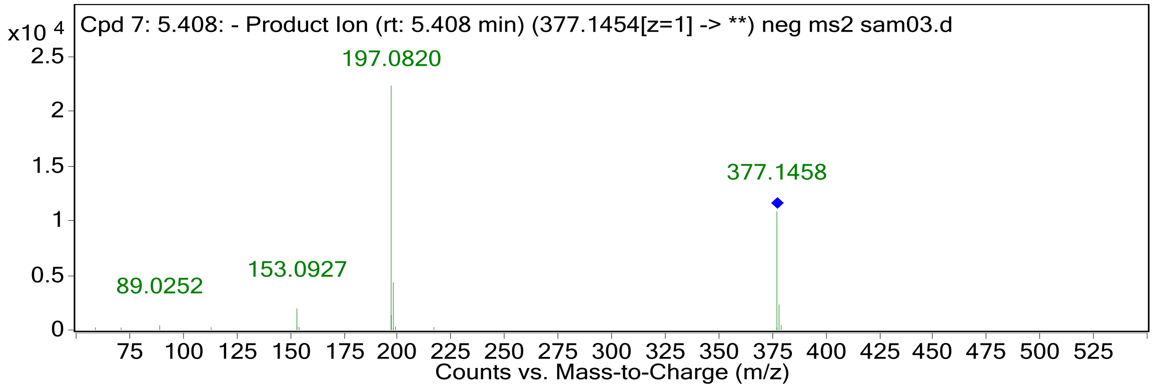

MSMS Spectrum

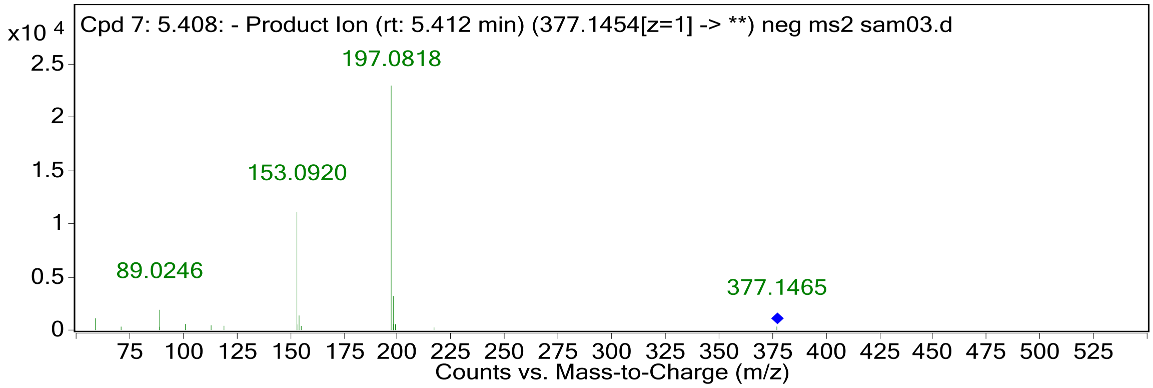

MSMS Spectrum

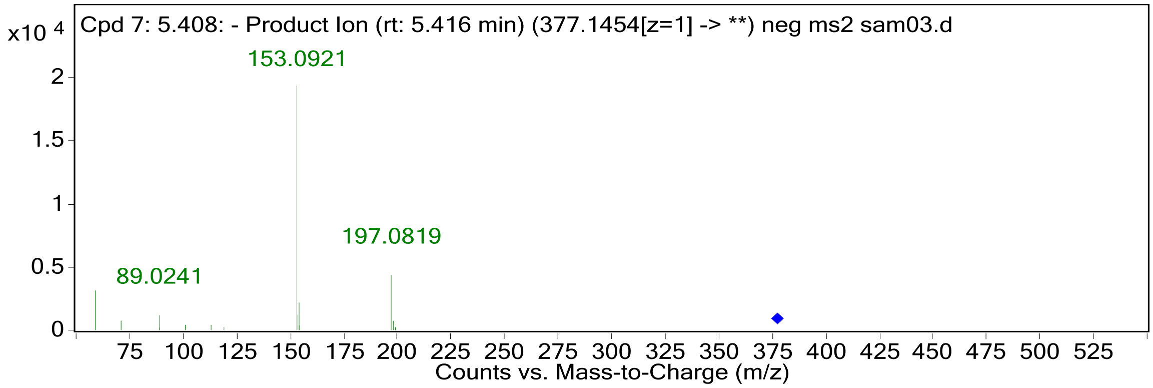

Compound Label	*m/z*	RT	Algorithm
Cpd 8: 5.540	755.2984	5.54	Targeted MS/MS

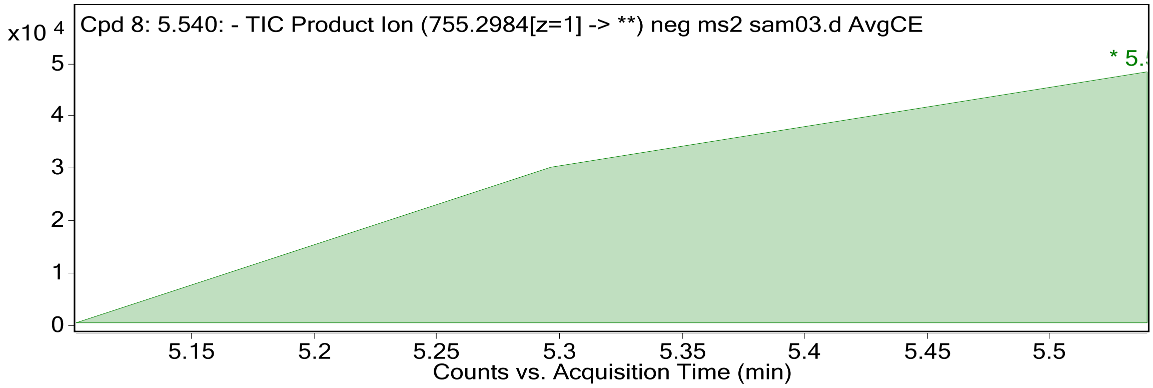

MSMS Spectrum

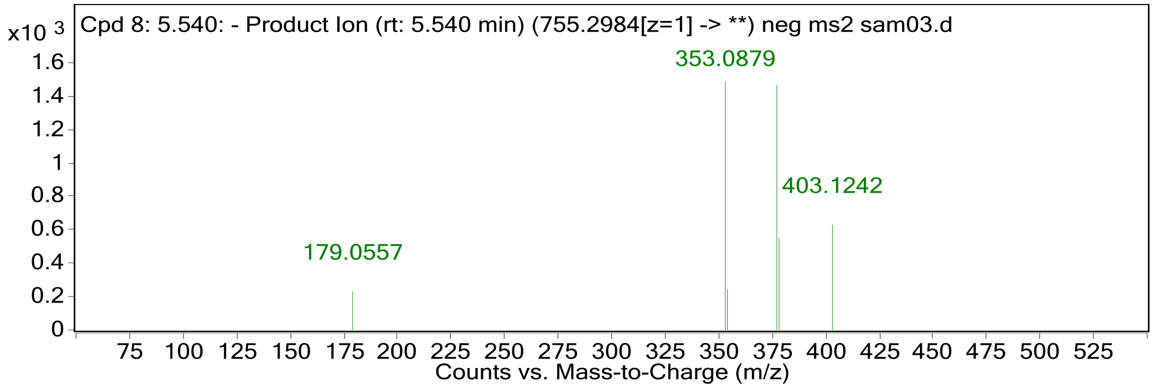

MSMS Spectrum

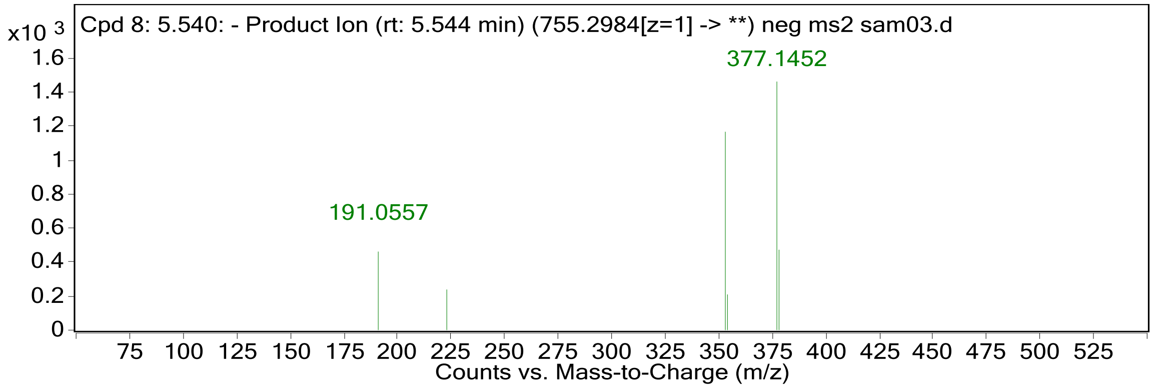

MSMS Spectrum

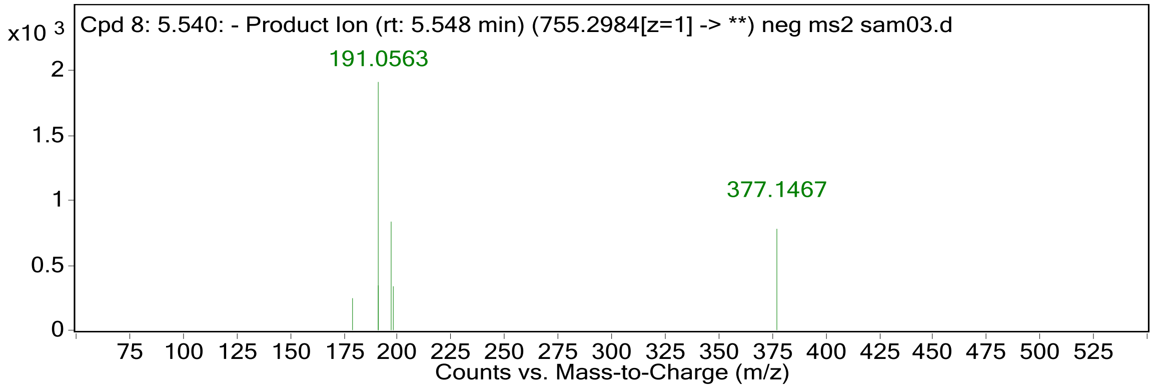

Compound Label	*m/z*	RT	Algorithm
Cpd 9: 5.626	919.273	5.626	Targeted MS/MS

Qualitative Compound Report

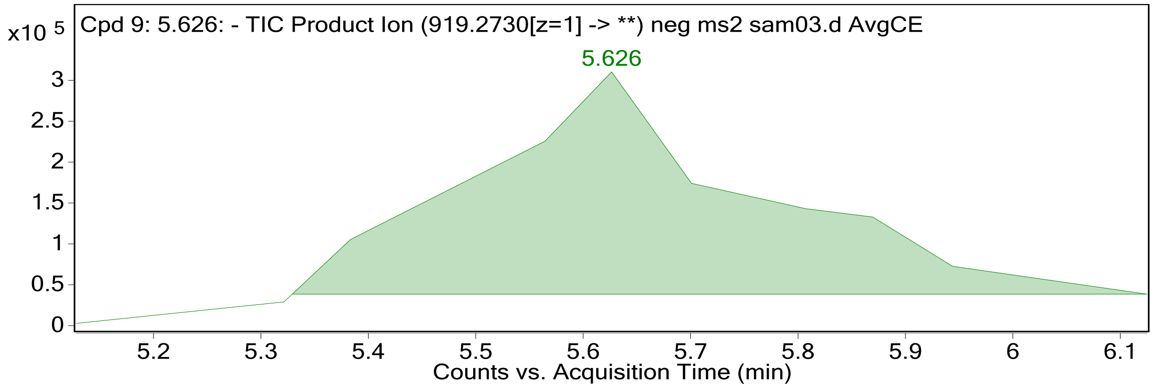

MSMS Spectrum

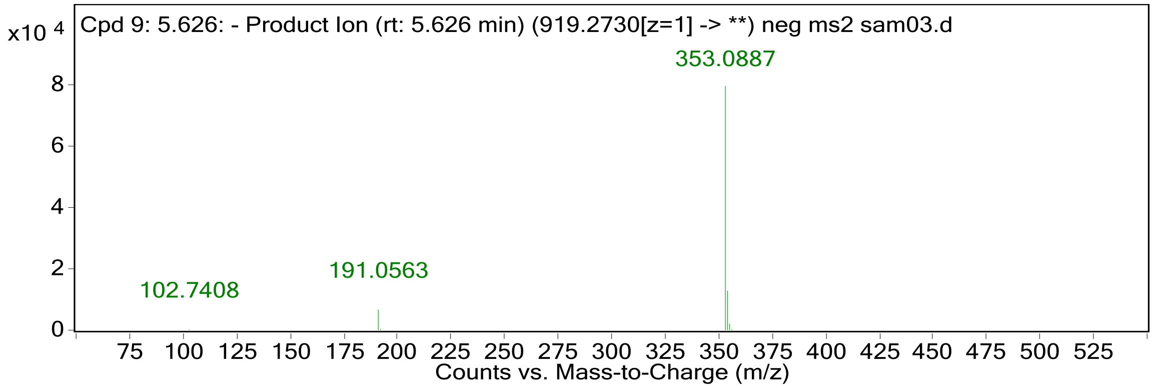

MSMS Spectrum

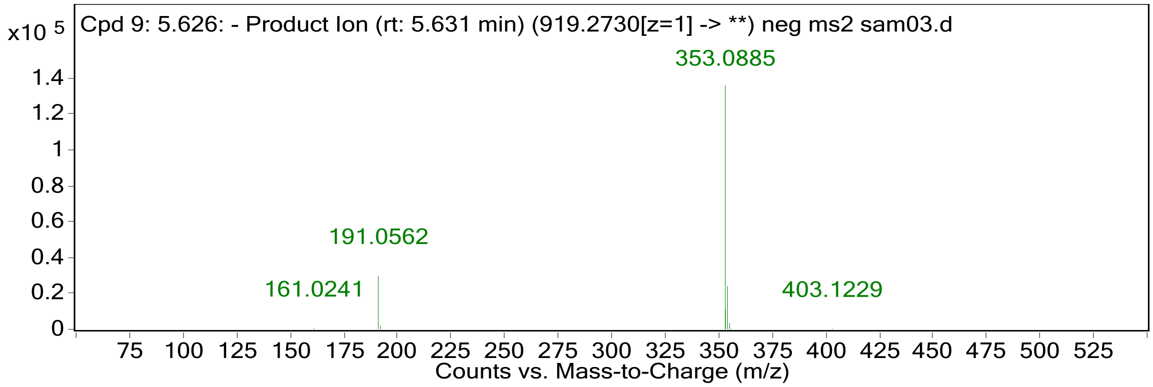

MSMS Spectrum

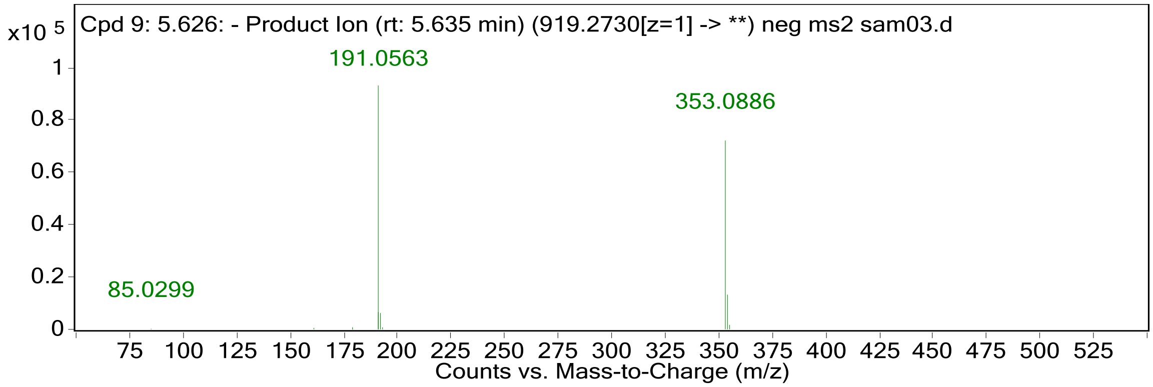

Qualitative Compound Report

Compound Label	*m/z*	RT	Algorithm
Cpd 10: 5.639	375.1298	5.639	Targeted MS/MS

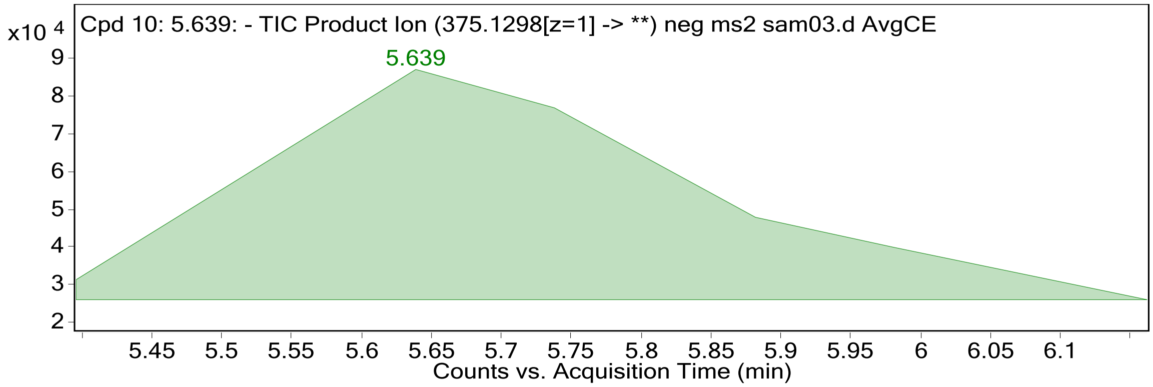

MSMS Spectrum

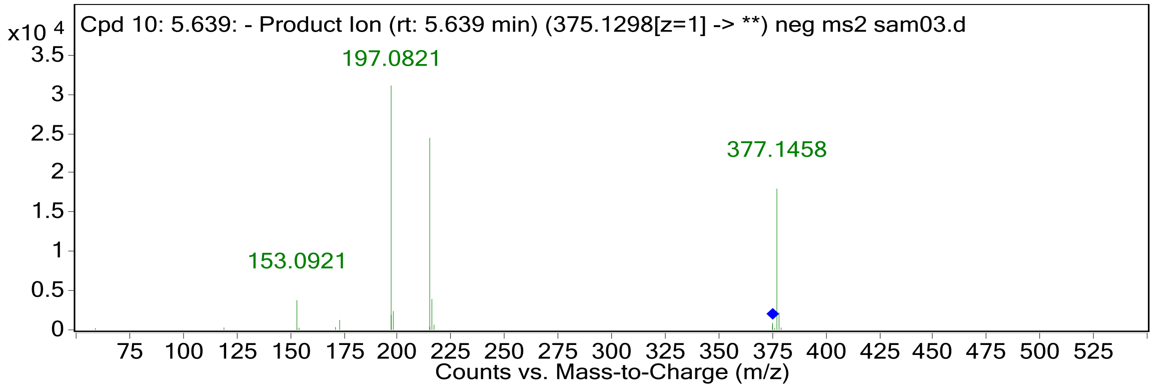

MSMS Spectrum

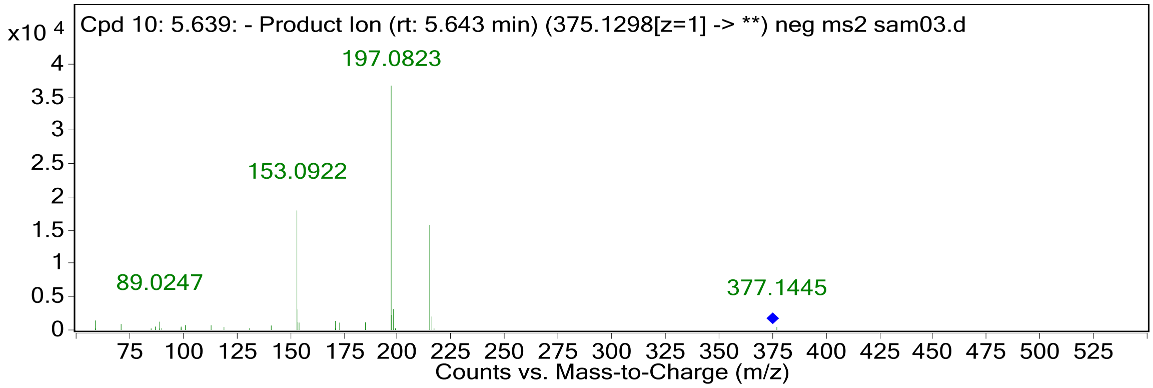

MSMS Spectrum

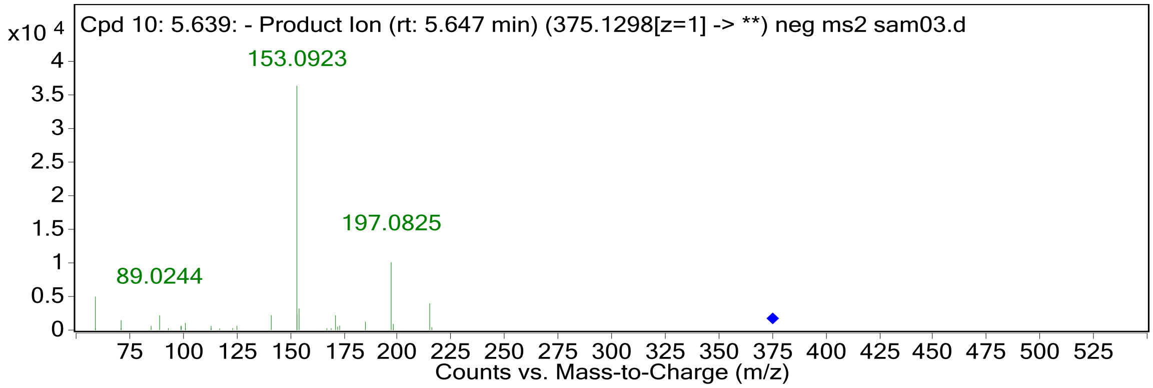

Compound Label	*m/z*	RT	Algorithm
Cpd 11: 5.676	191.0559	5.676	Targeted MS/MS

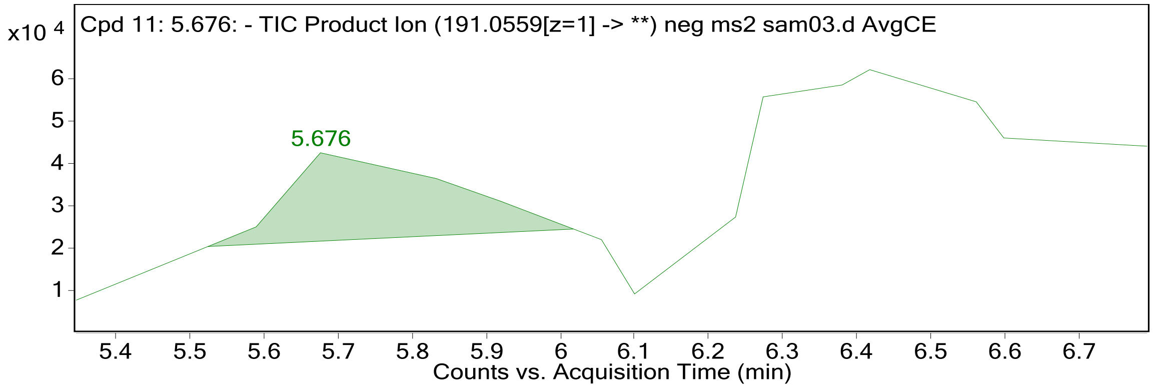

MSMS Spectrum

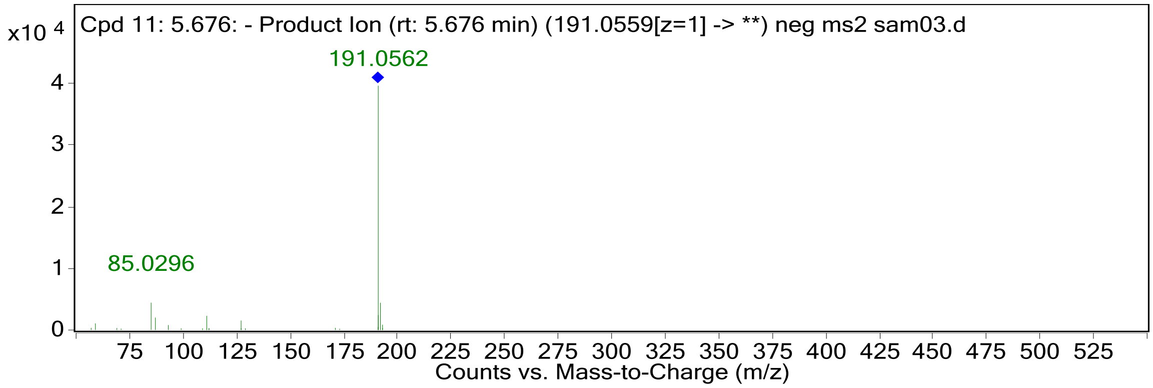

MSMS Spectrum

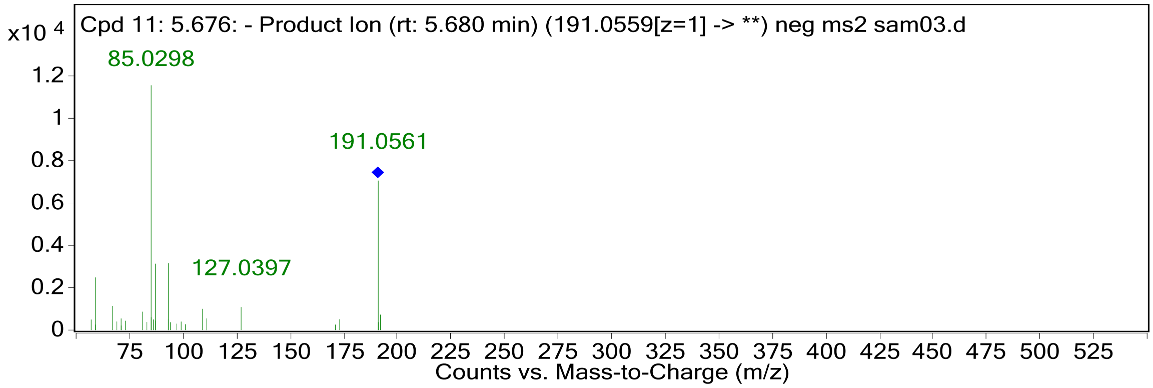

MSMS Spectrum

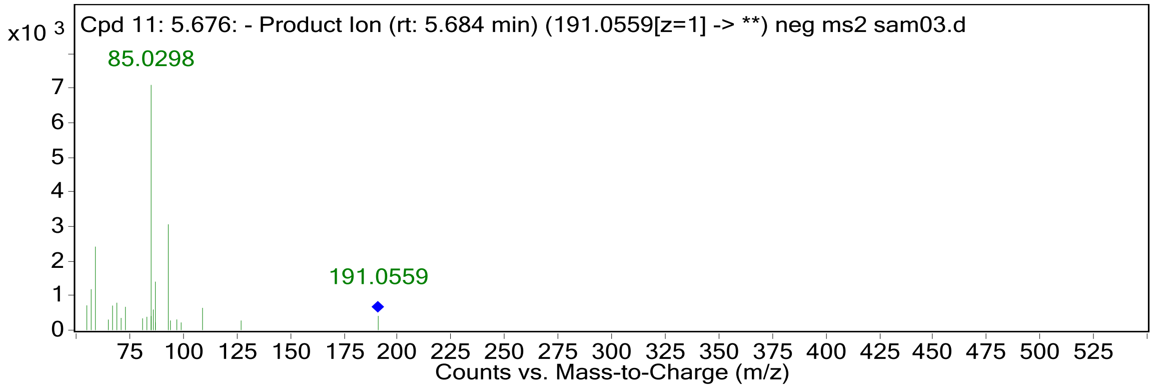

Compound Label	*m/z*	RT	Algorithm
Cpd 12: 5.713	729.2254	5.713	Targeted MS/MS

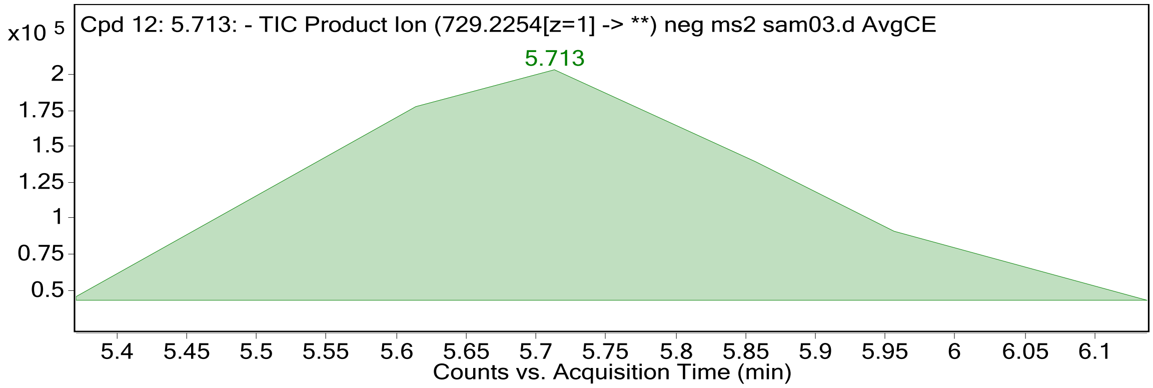

MSMS Spectrum

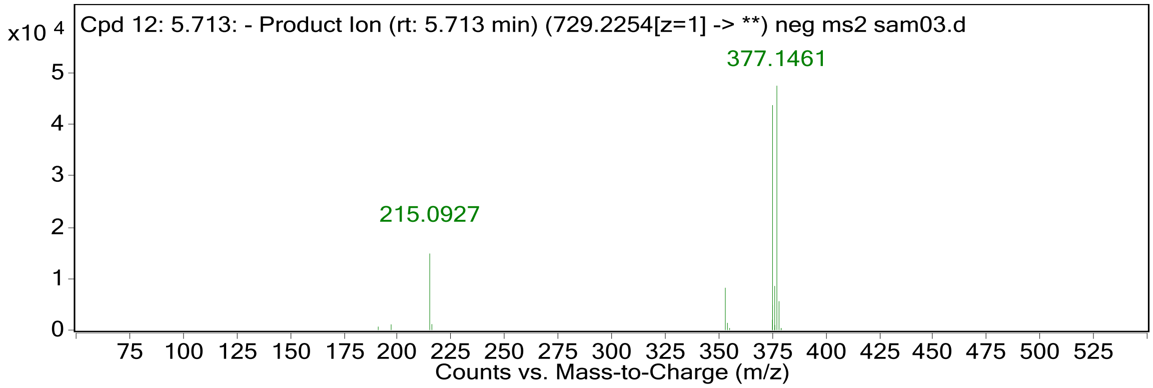

MSMS Spectrum

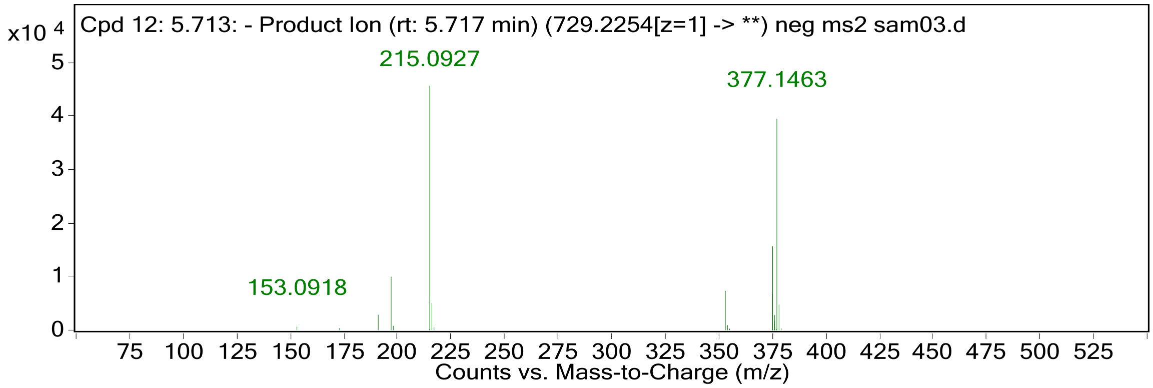

MSMS Spectrum

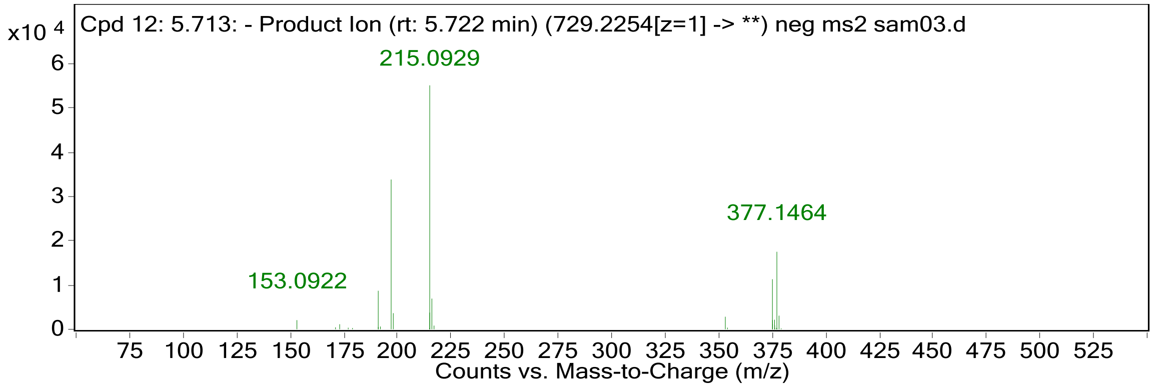

Compound Label	*m/z*	RT	Algorithm
Cpd 13: 5.726	305.07	5.726	Targeted MS/MS

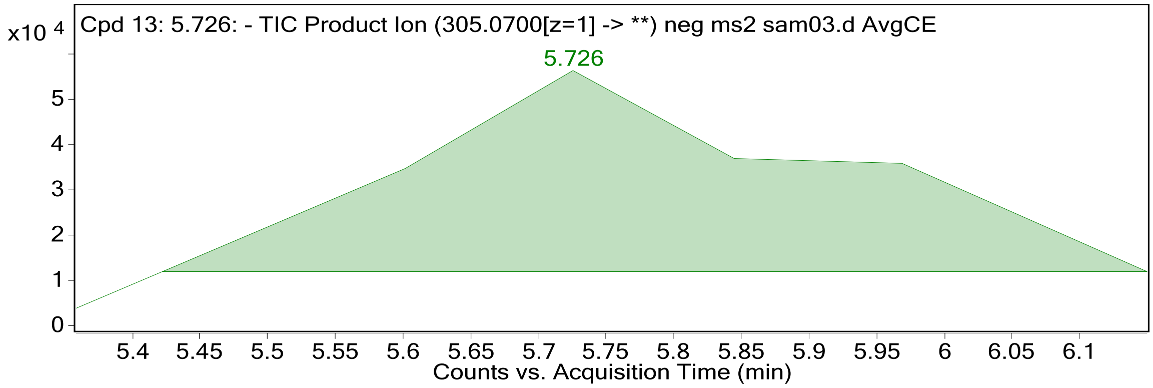

MSMS Spectrum

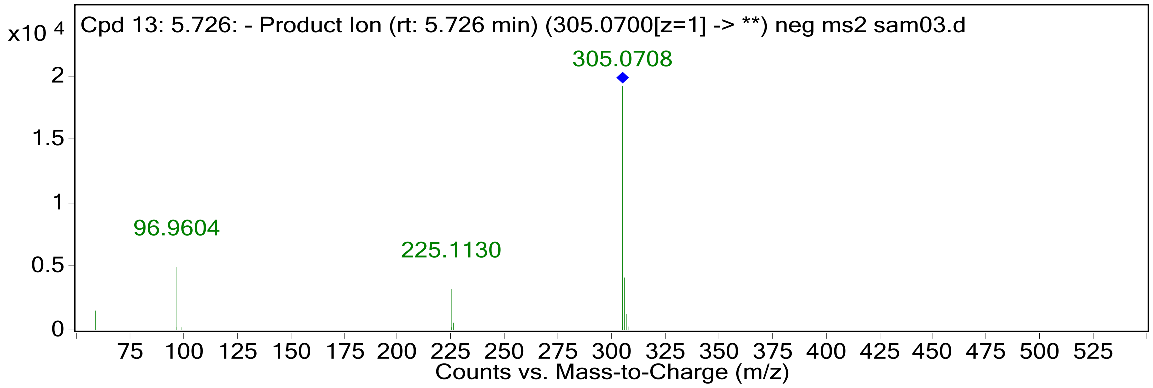

MSMS Spectrum

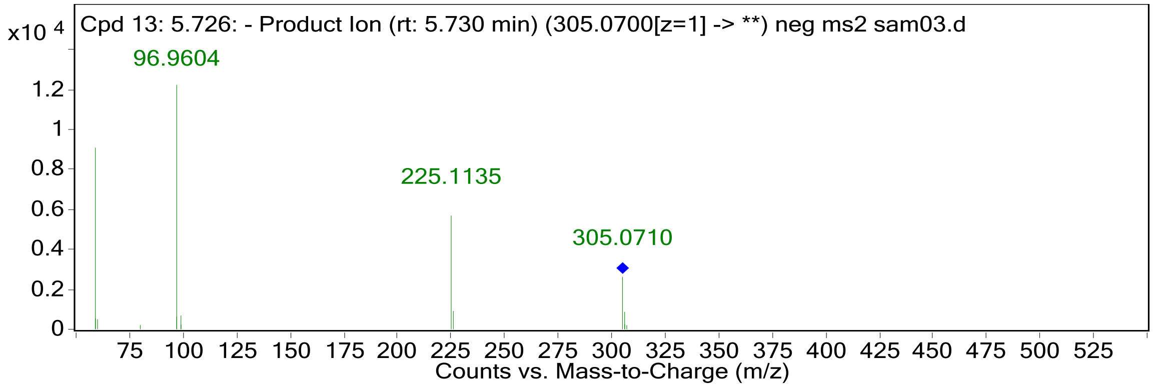

MSMS Spectrum

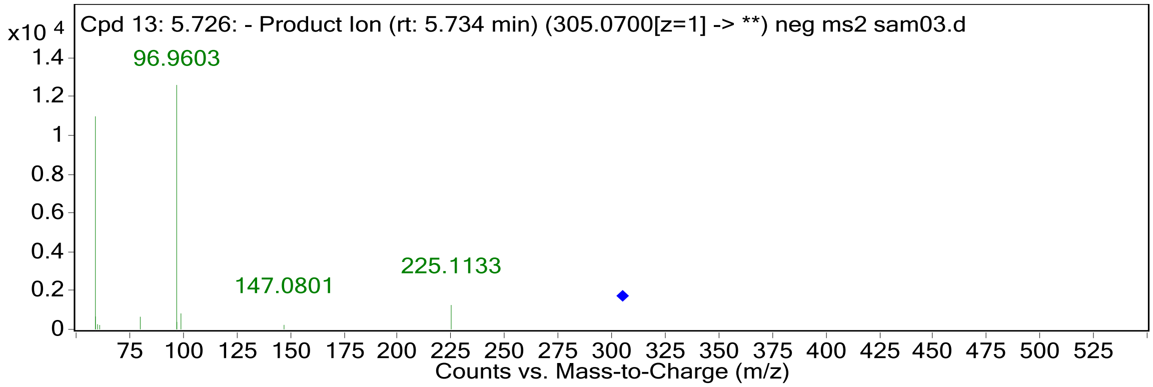

Compound Label	*m/z*	RT	Algorithm
Cpd 14: 5.775	461.1665	5.775	Targeted MS/MS

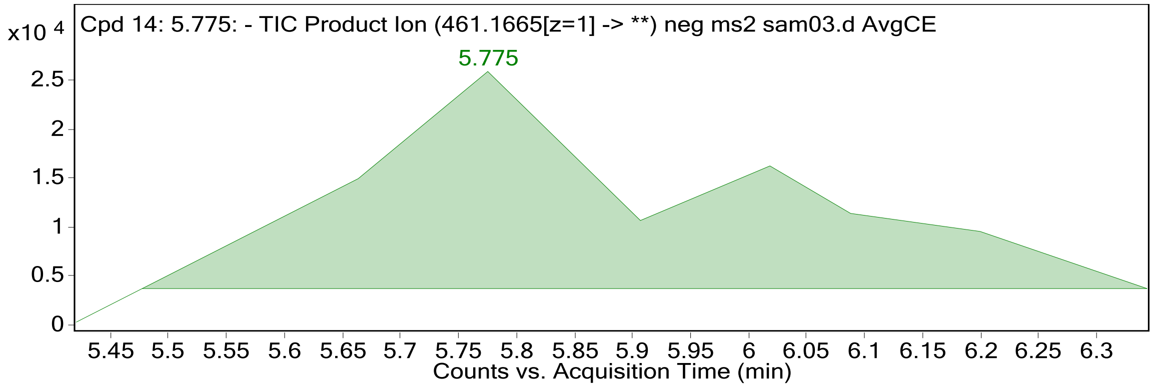

MSMS Spectrum

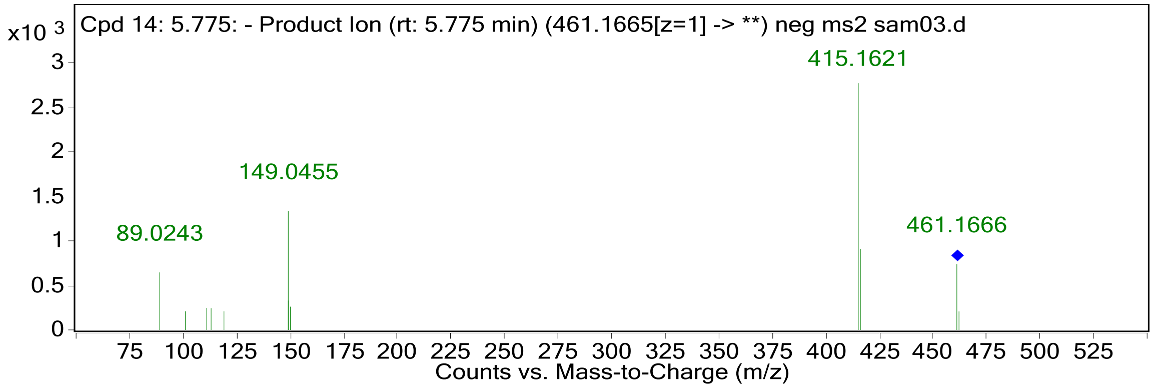

MSMS Spectrum

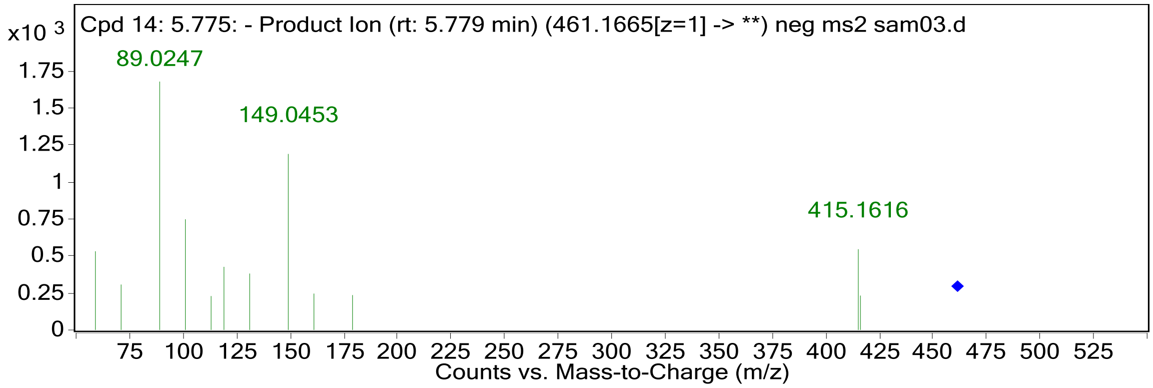

MSMS Spectrum

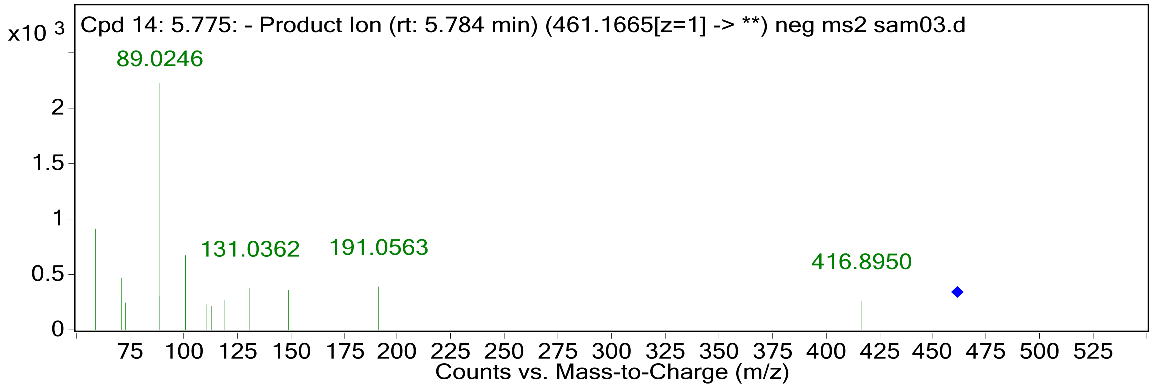

Compound Label	*m/z*	RT	Algorithm
Cpd 15: 5.820	353.0877	5.82	Targeted MS/MS

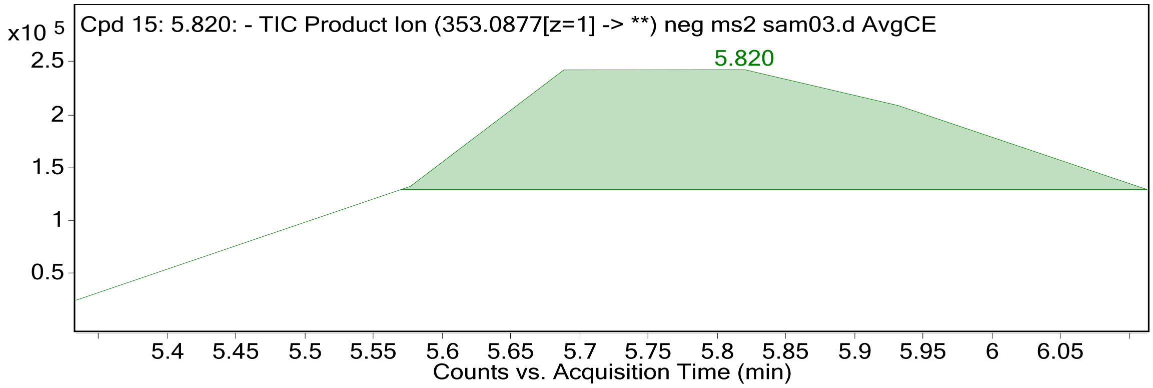

MSMS Spectrum

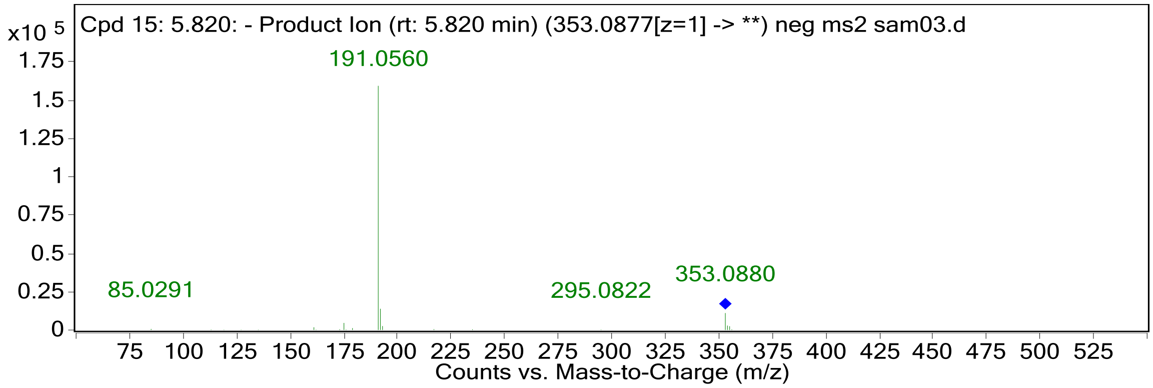

MSMS Spectrum

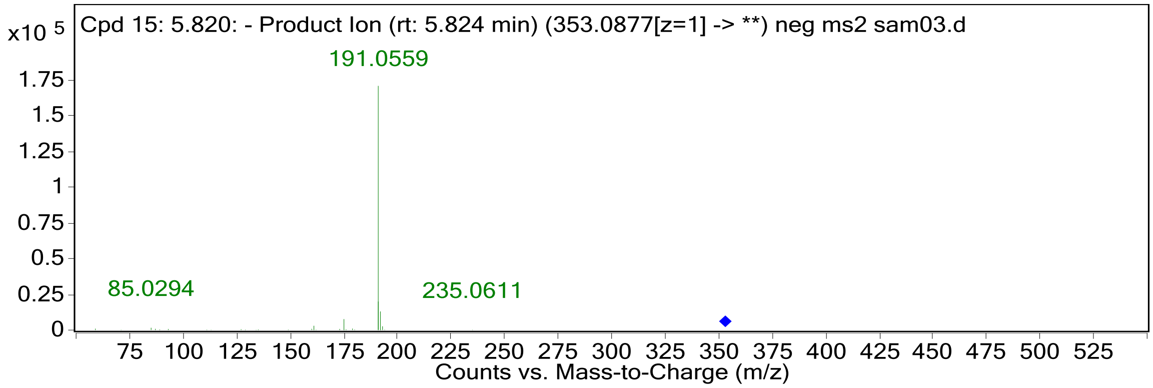

MSMS Spectrum

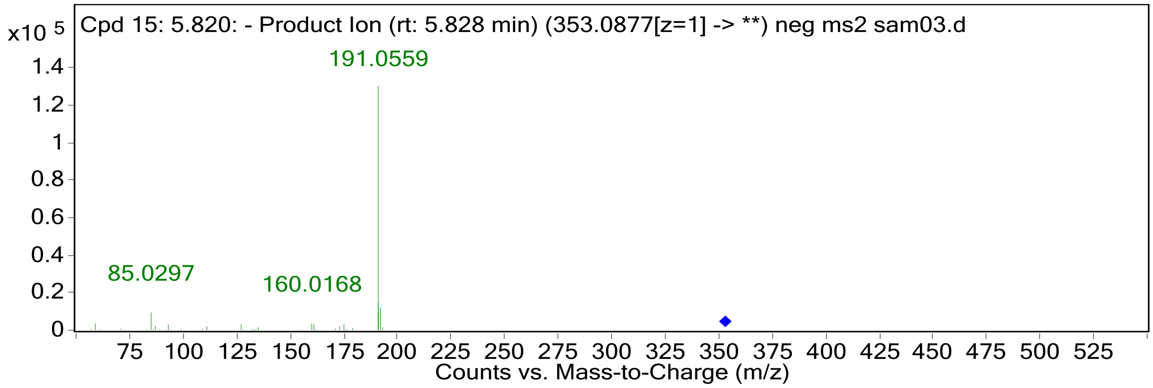

Compound Label	*m/z*	RT	Algorithm
Cpd 16: 5.994	891.2933	5.994	Targeted MS/MS

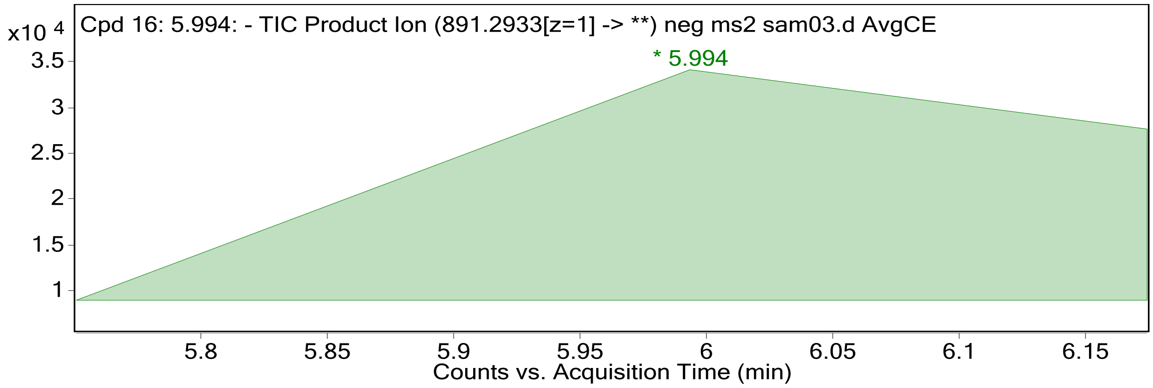

MSMS Spectrum

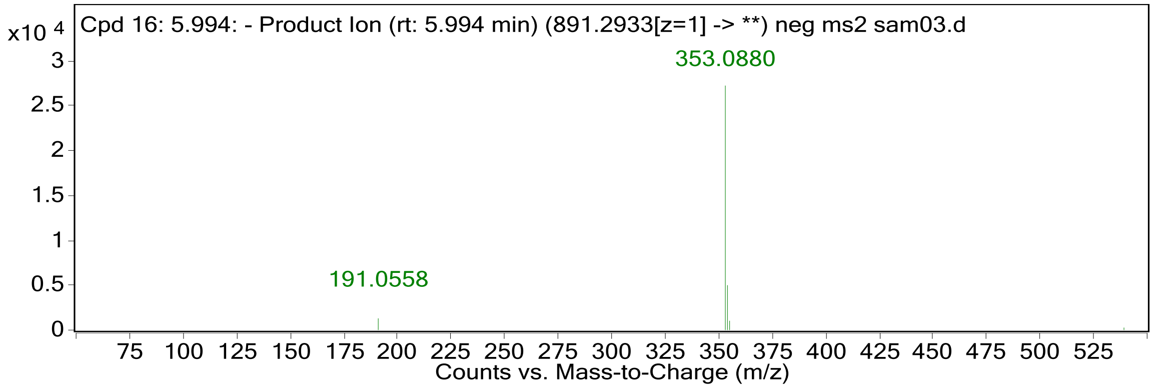

MSMS Spectrum

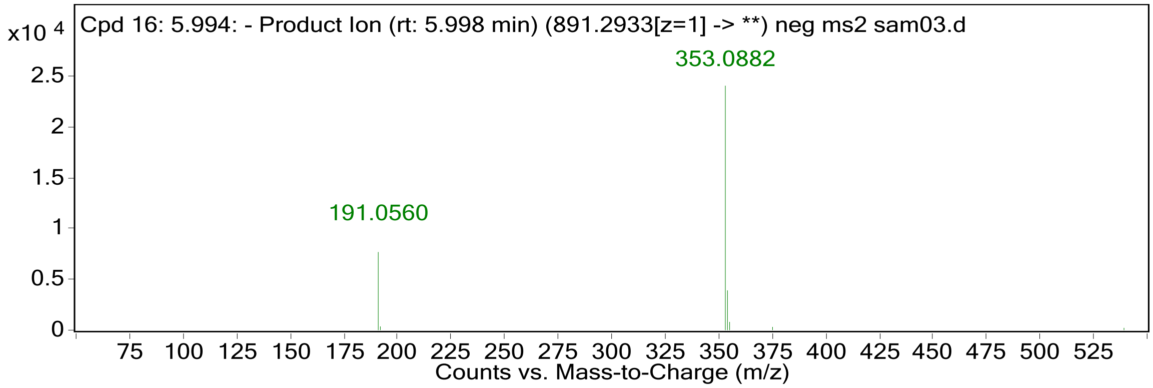

MSMS Spectrum

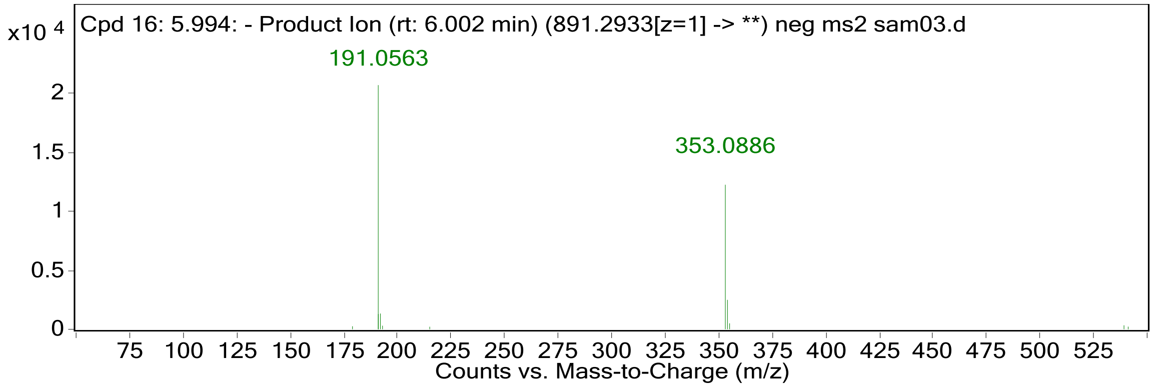

Compound Label	*m/z*	RT	Algorithm
Cpd 17: 6.187	583.2034	6.187	Targeted MS/MS

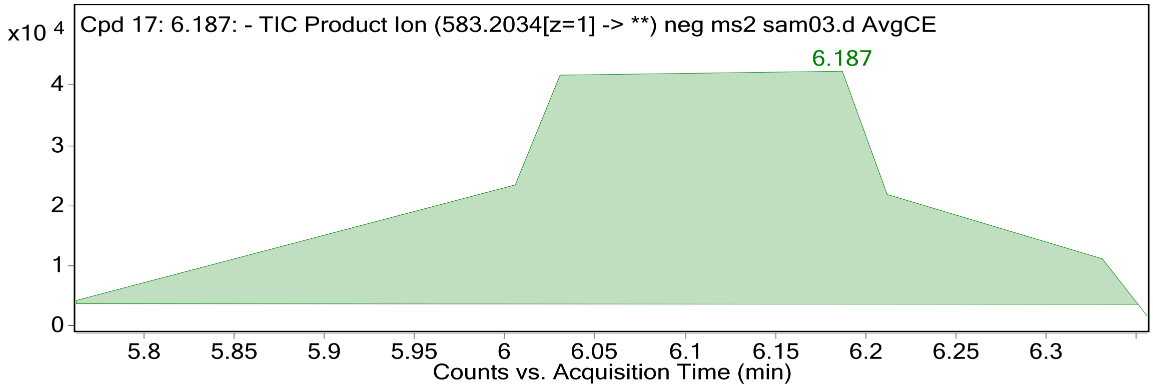
Cpd 17: 6.187: - TIC Product Ion (583.2034[z=1] -> **) neg ms2 sam03.d AvgCE
x10 4
6.187
4
3
2
1
0
5.8
5.85
5.9
5.95
6
6.05
6.1
6.15
6.2
6.25
6.3
Counts vs. Acquisition Time (min)

Qualitative Compound Report

MSMS Spectrum

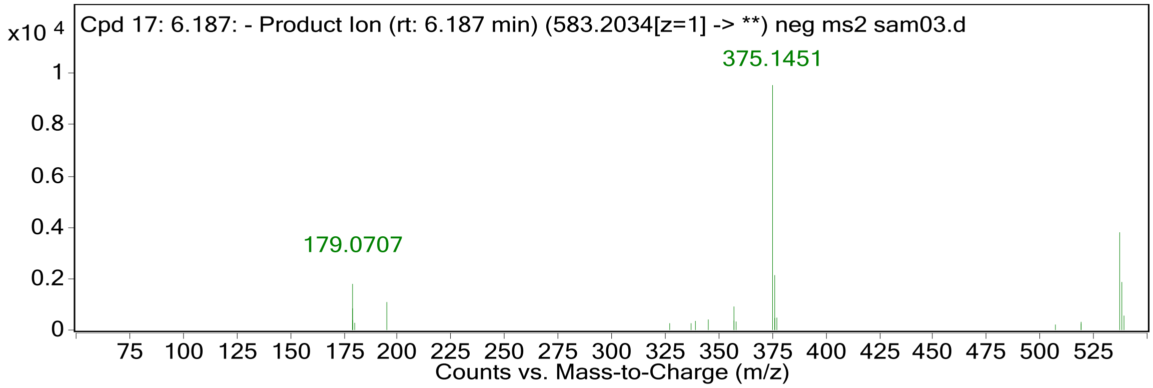

MSMS Spectrum

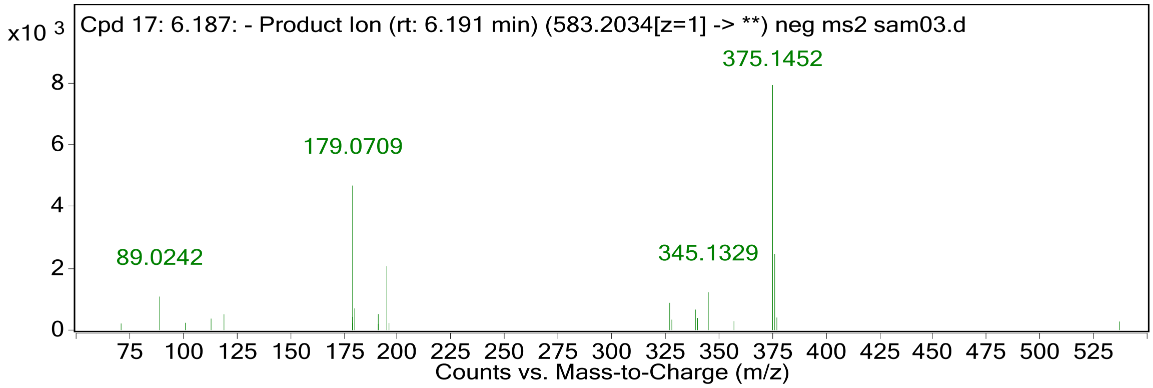

MSMS Spectrum

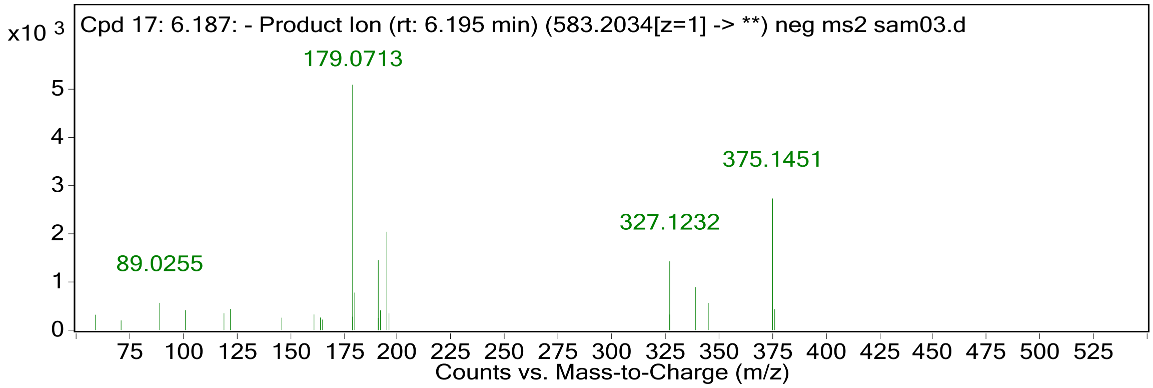

**Compound Label**	***m/z***	**RT**	**Algorithm**
Cpd 18: 6.418	191.0559	6.418	Targeted MS/MS

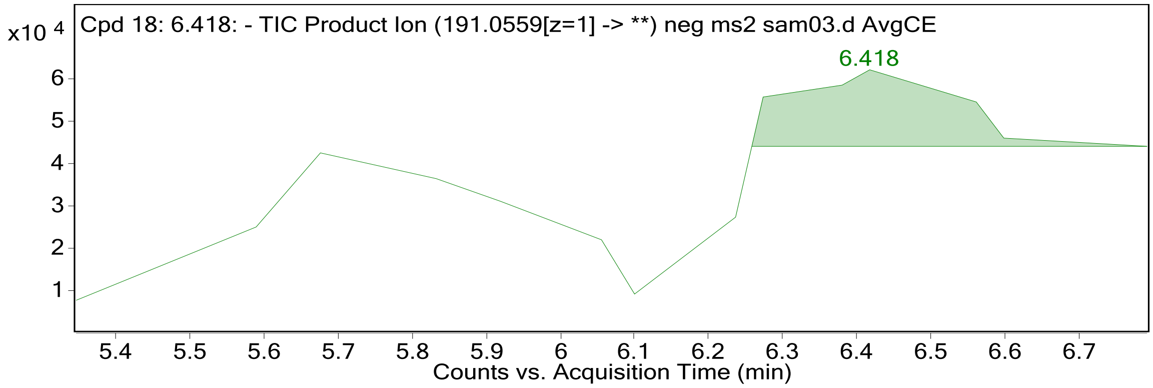

MSMS Spectrum

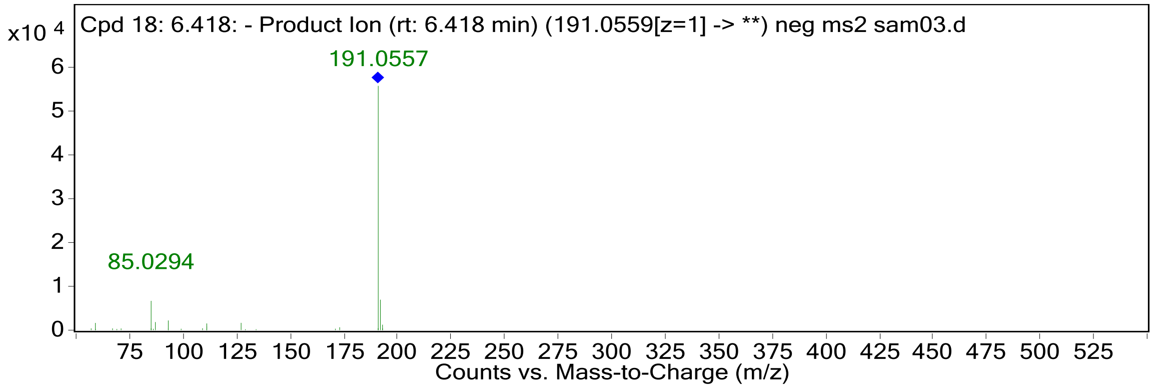

MSMS Spectrum

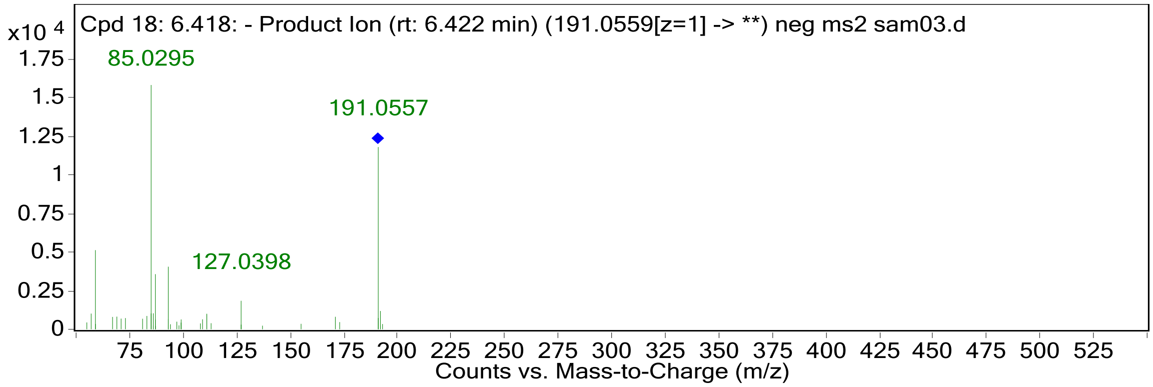

MSMS Spectrum

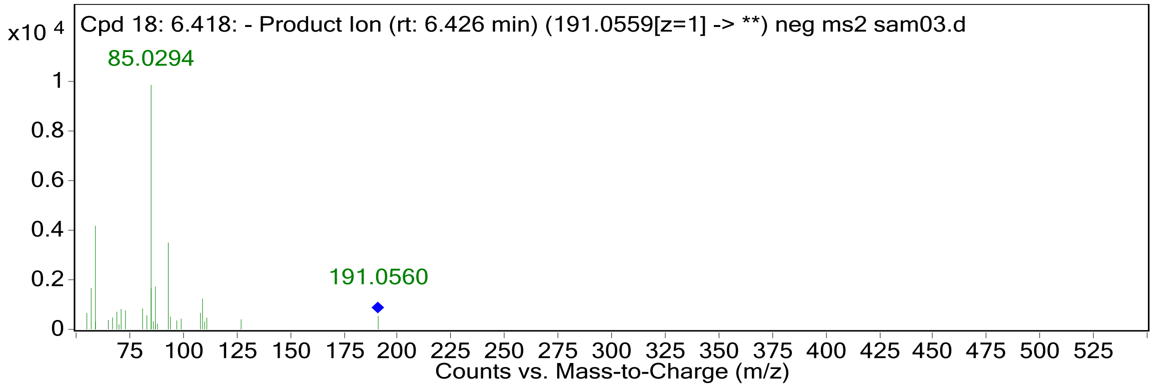

Compound Label	*m/z*	RT	Algorithm
Cpd 19: 6.455	337.0933	6.455	Targeted MS/MS

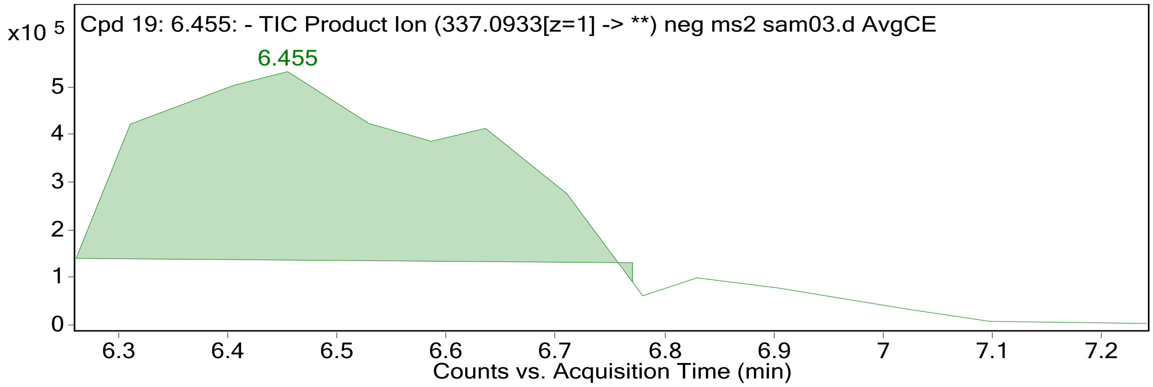

MSMS Spectrum

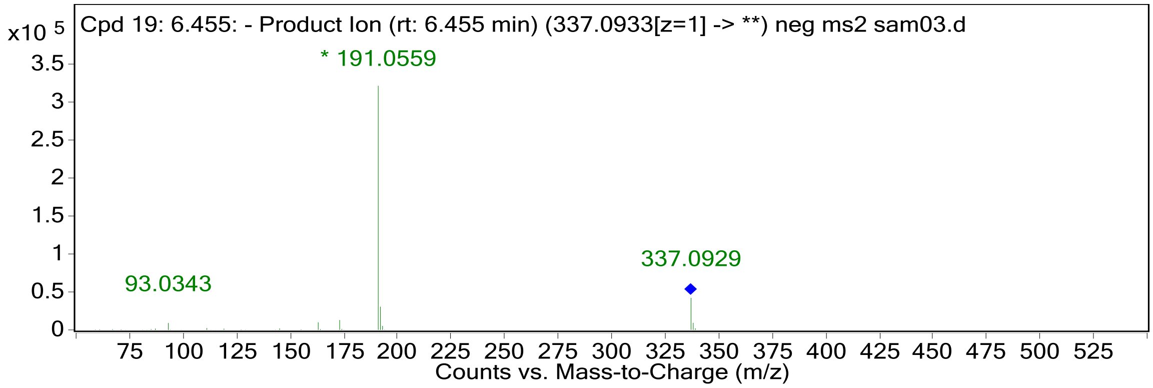

MSMS Spectrum

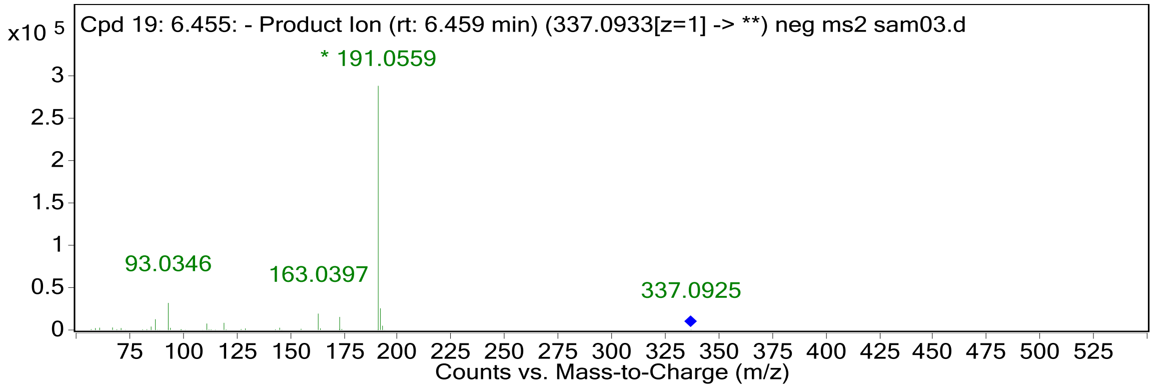

MSMS Spectrum

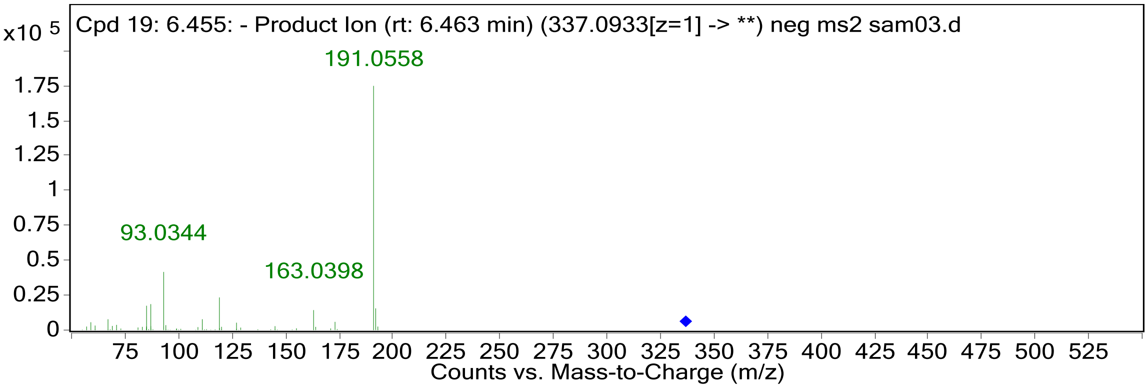

**Compound Label**	***m/z***	**RT**	**Algorithm**
Cpd 20: 6.698	705.204	6.698	Targeted MS/MS

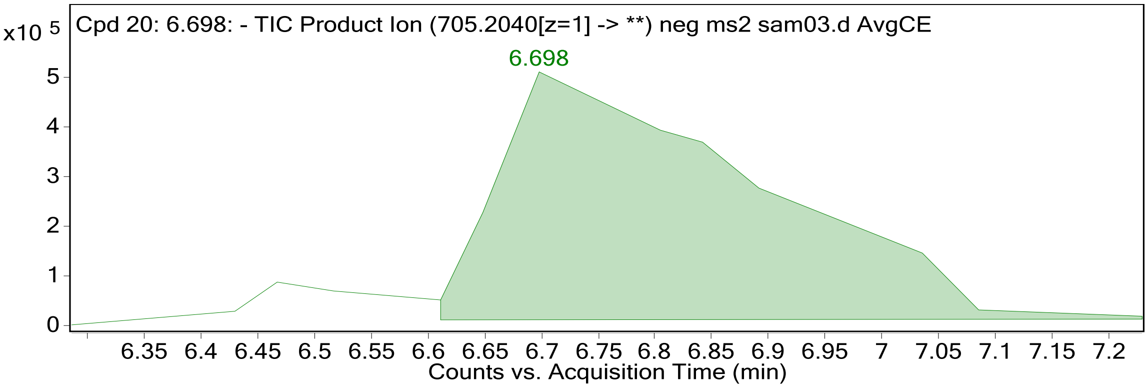

MSMS Spectrum

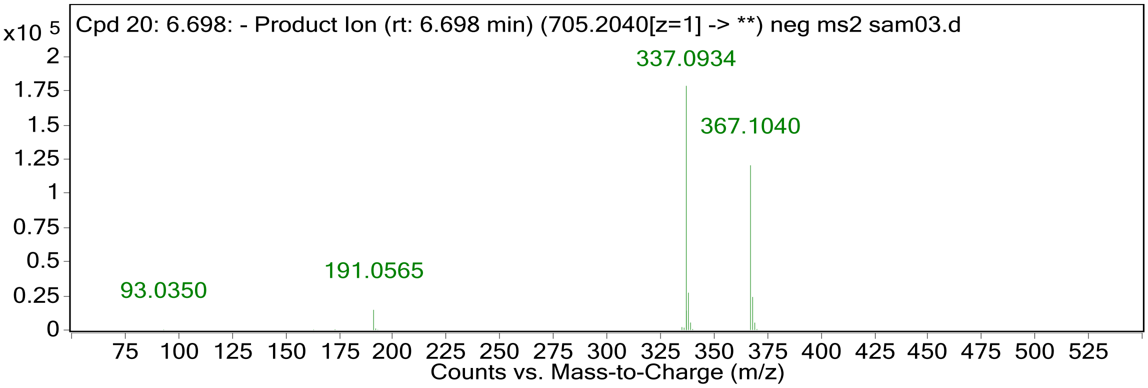

MSMS Spectrum

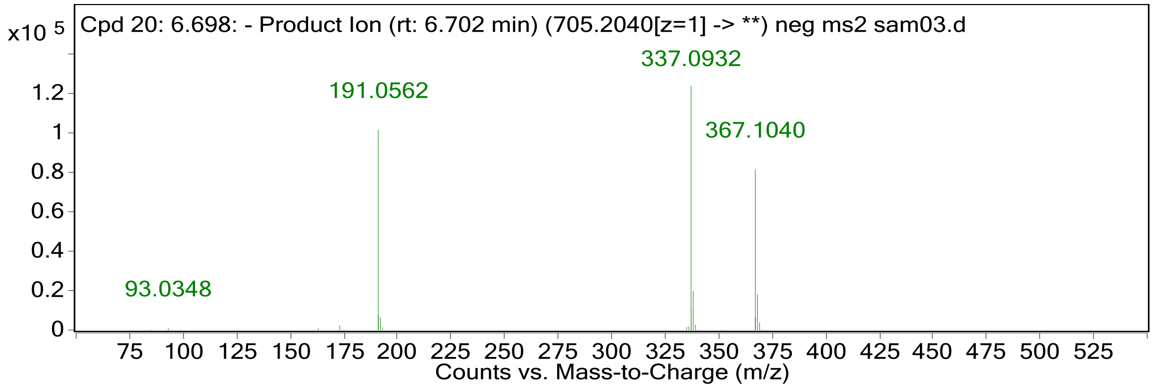

MSMS Spectrum

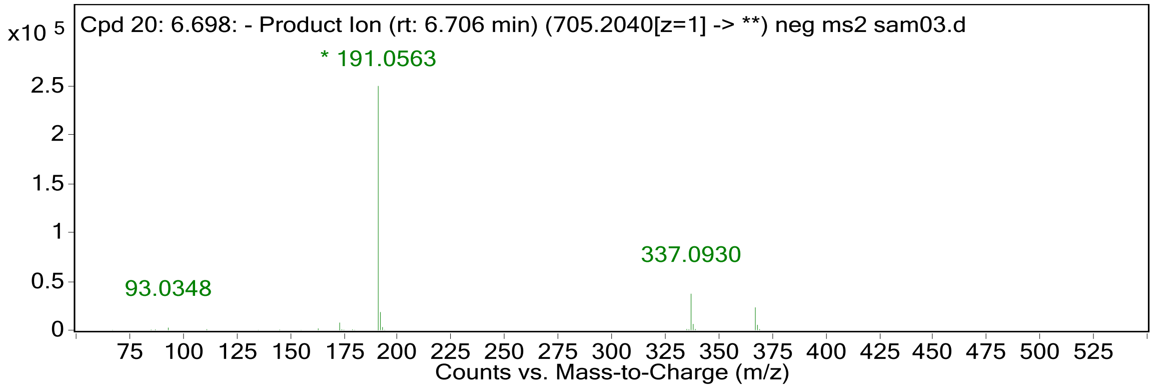

Compound Label	*m/z*	RT	Algorithm
Cpd 21: 6.723	703.1886	6.723	Targeted MS/MS

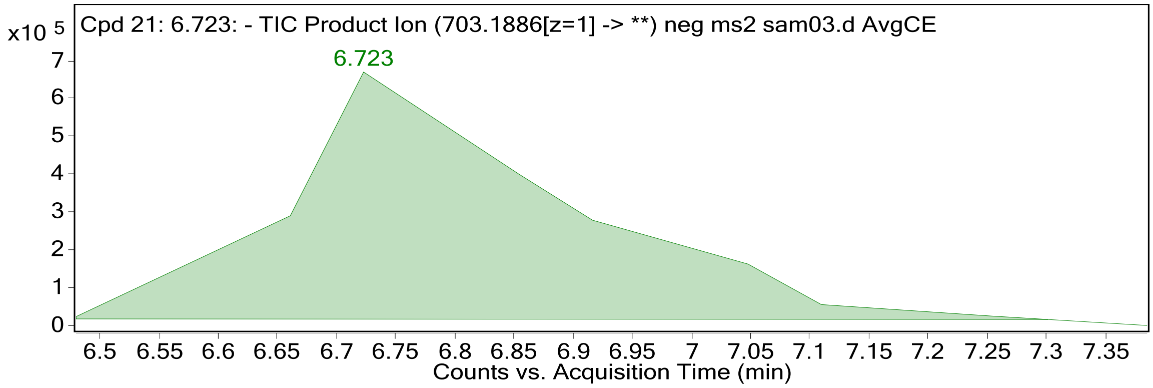

MSMS Spectrum

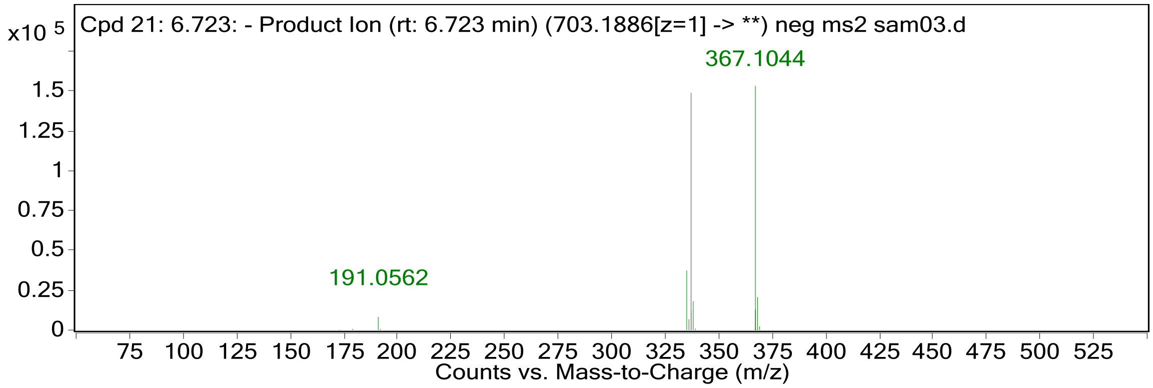

MSMS Spectrum

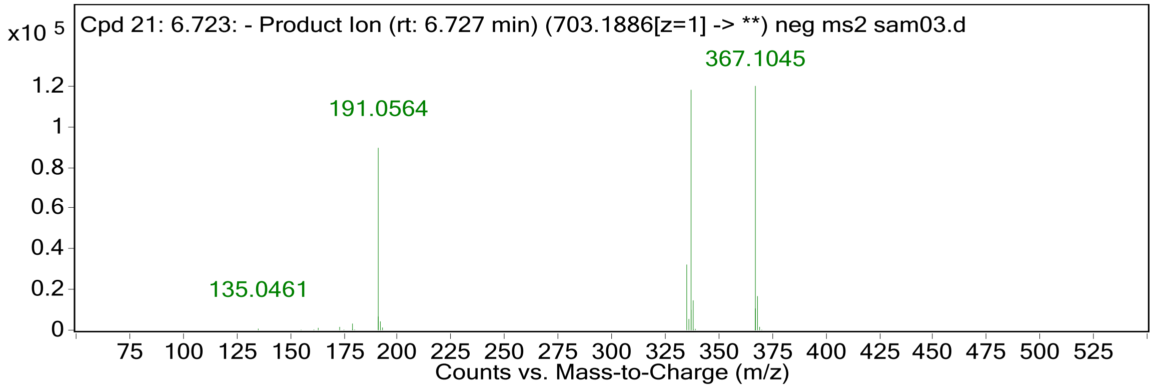

MSMS Spectrum

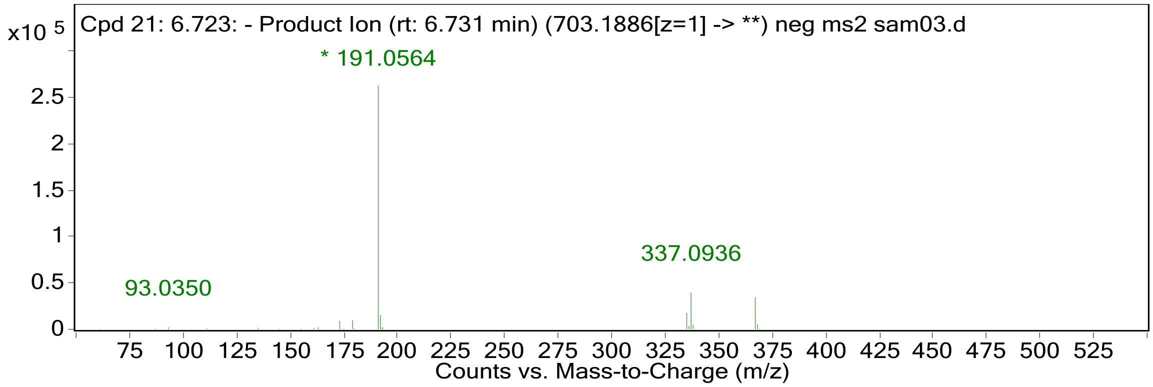

Compound Label	*m/z*	RT	Algorithm
Cpd 22: 6.735	367.1034	6.735	Targeted MS/MS

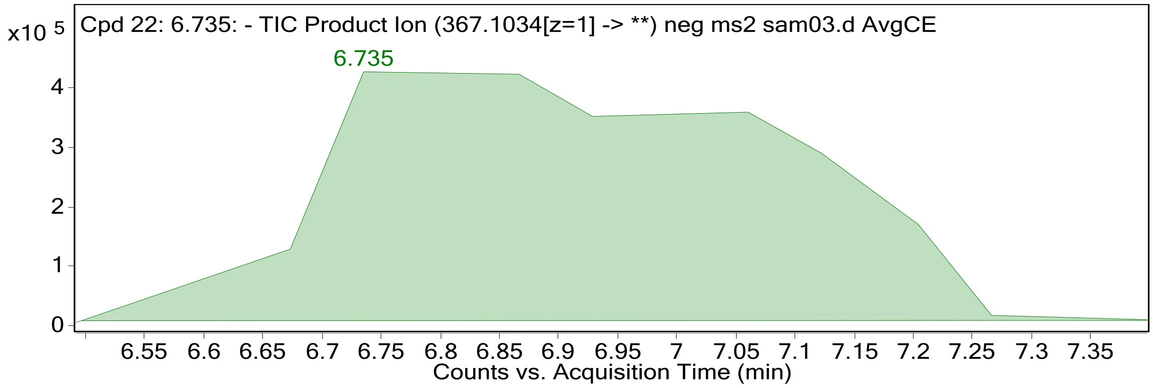

MSMS Spectrum

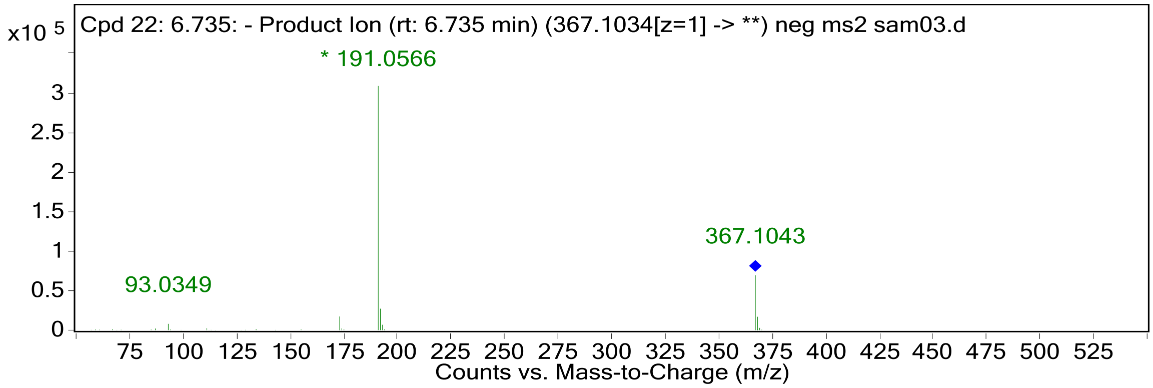

MSMS Spectrum

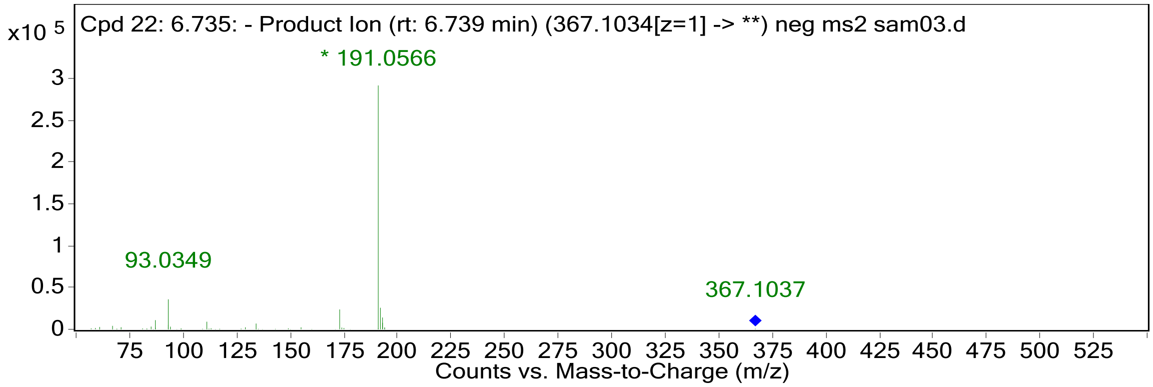

MSMS Spectrum

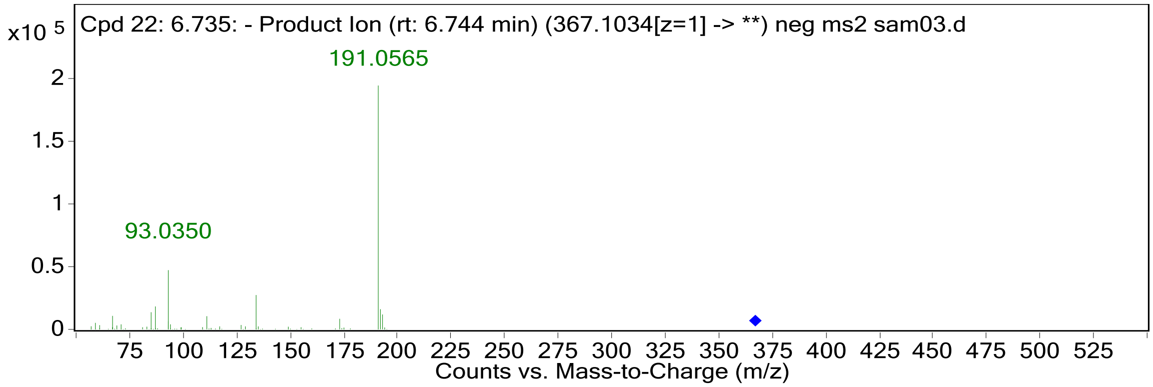

Compound Label	*m/z*	RT	Algorithm
Cpd 23: 7.159	735.2652	7.159	Targeted MS/MS

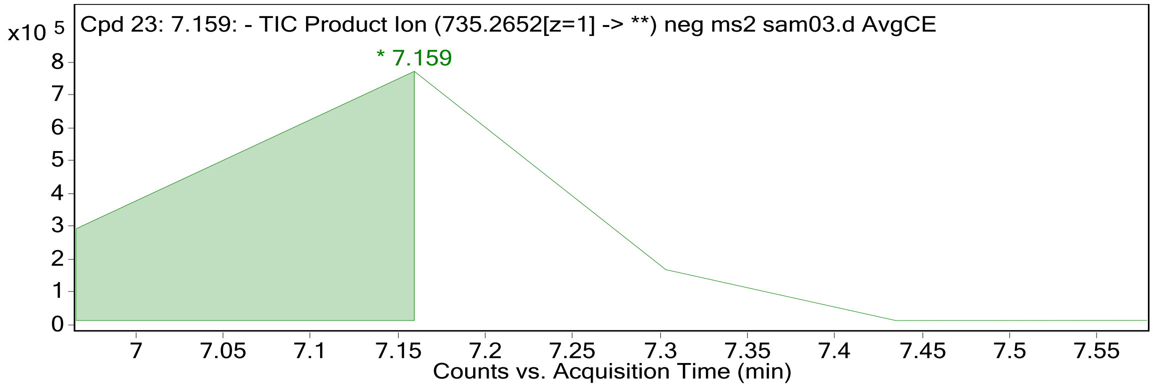

MSMS Spectrum

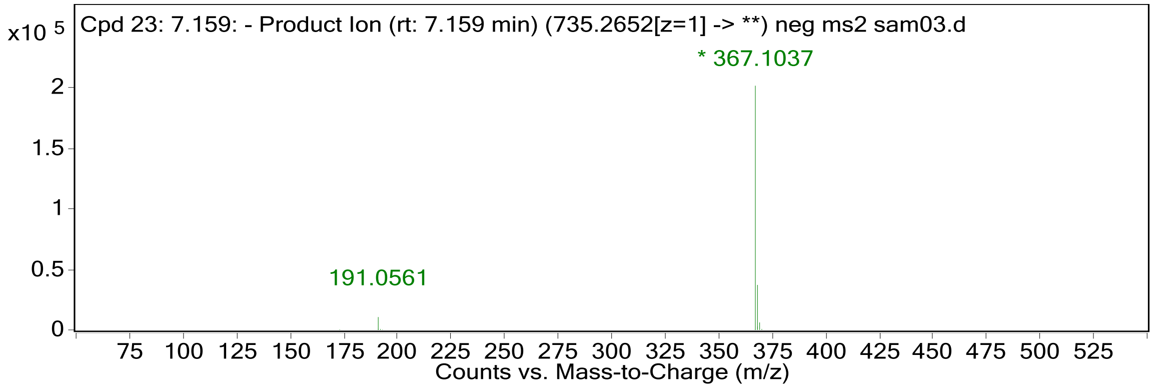

MSMS Spectrum

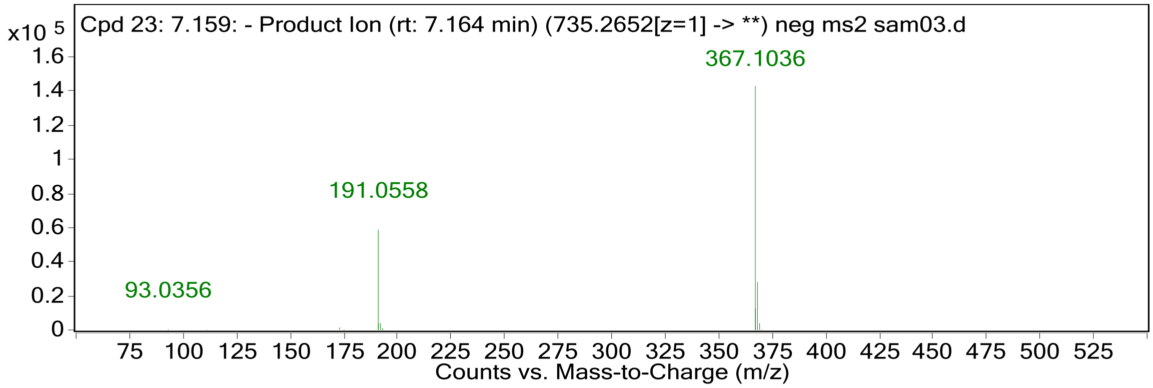

MSMS Spectrum

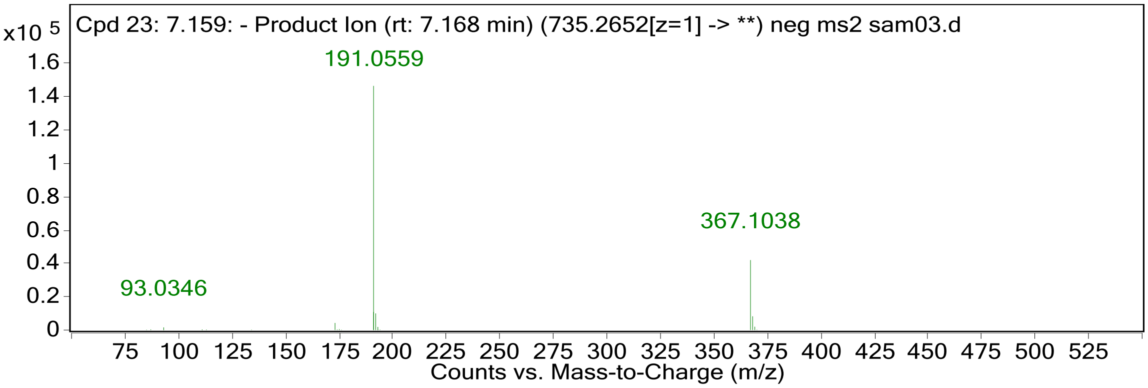

Compound Label	*m/z*	RT	Algorithm
Cpd 24: 7.678	609.1464	7.678	Targeted MS/MS

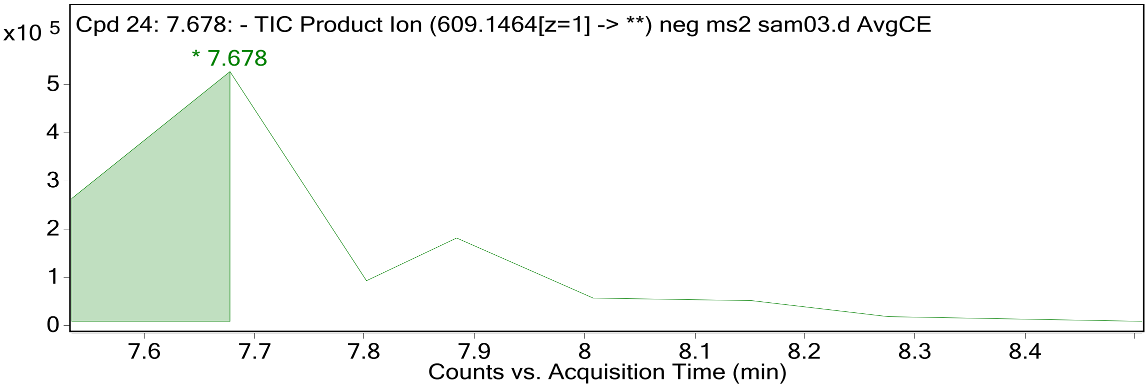

MSMS Spectrum

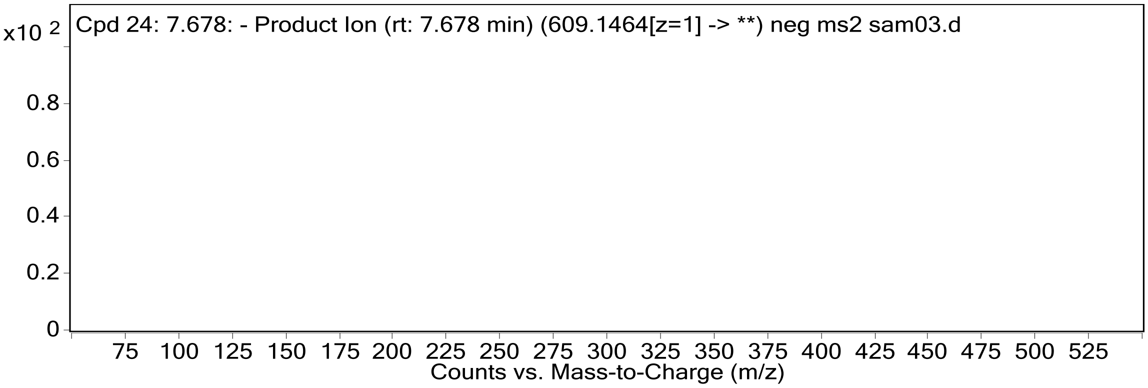

MSMS Spectrum

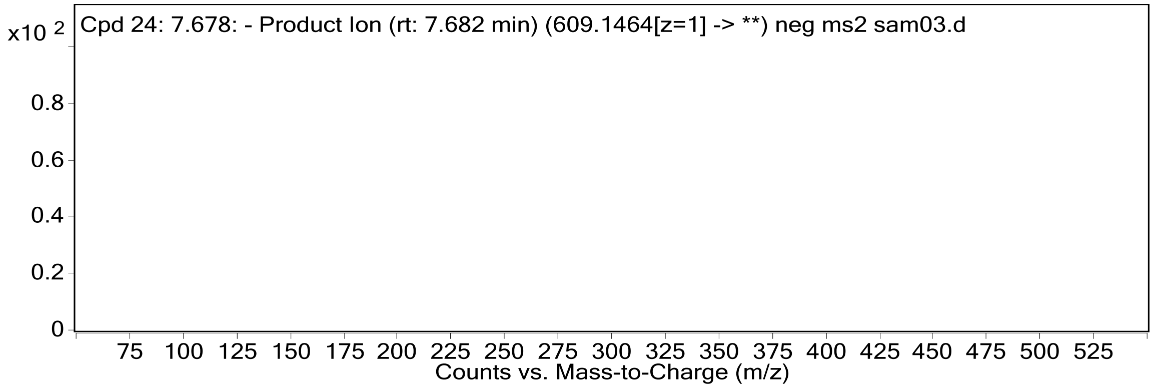

MSMS Spectrum

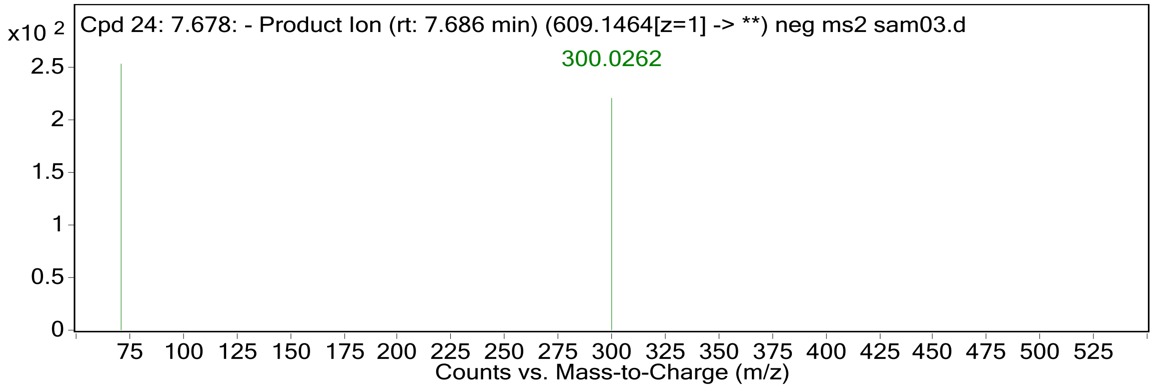

Compound Label	*m/z*	RT	Algorithm
Cpd 25: 7.909	607.2034	7.909	Targeted MS/MS

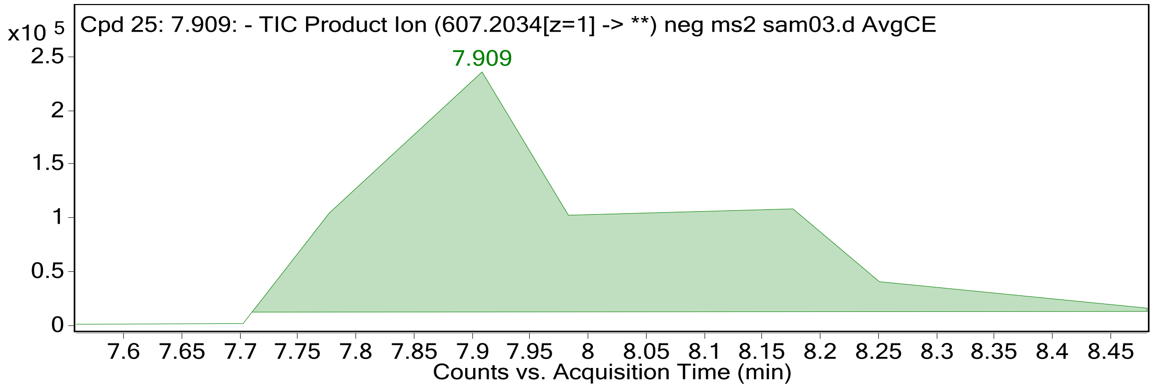

MSMS Spectrum

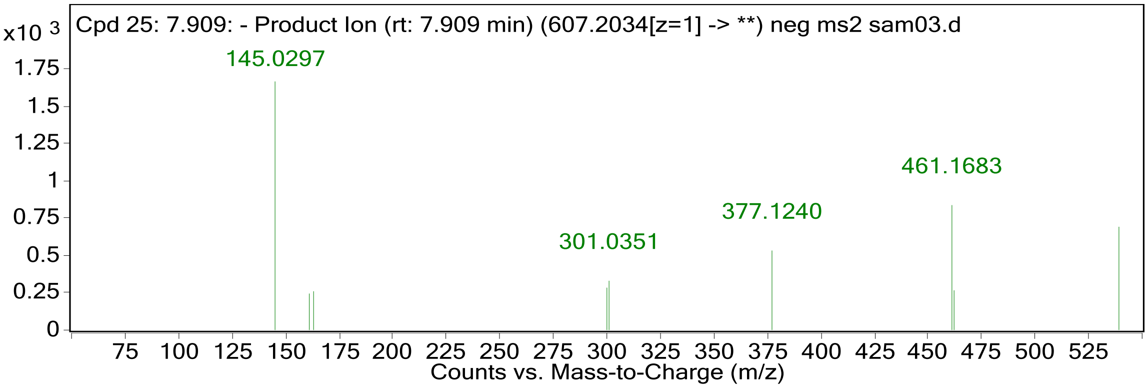

MSMS Spectrum

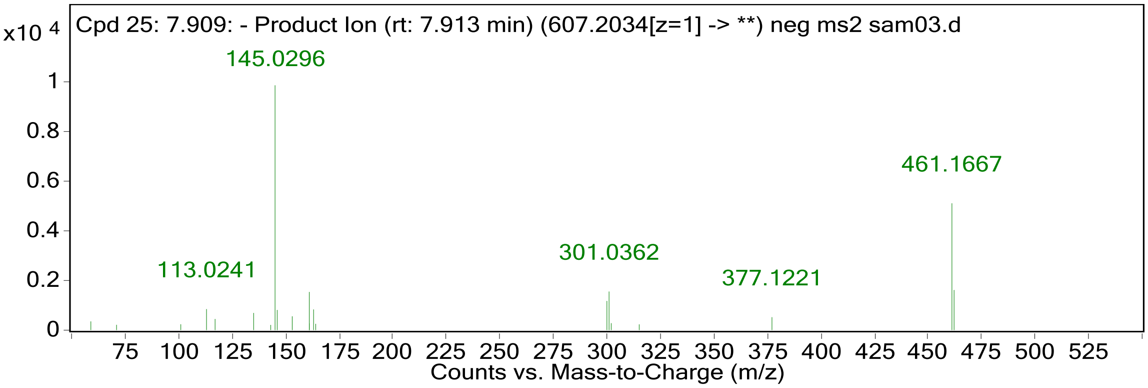

MSMS Spectrum

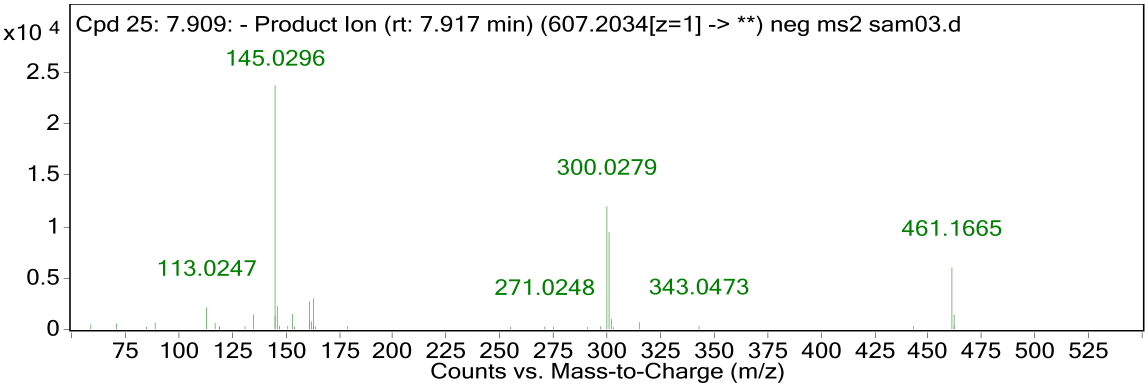

Compound Label	*m/z*	RT	Algorithm
Cpd 26: 7.921	623.1988	7.921	Targeted MS/MS

Qualitative Compound Report

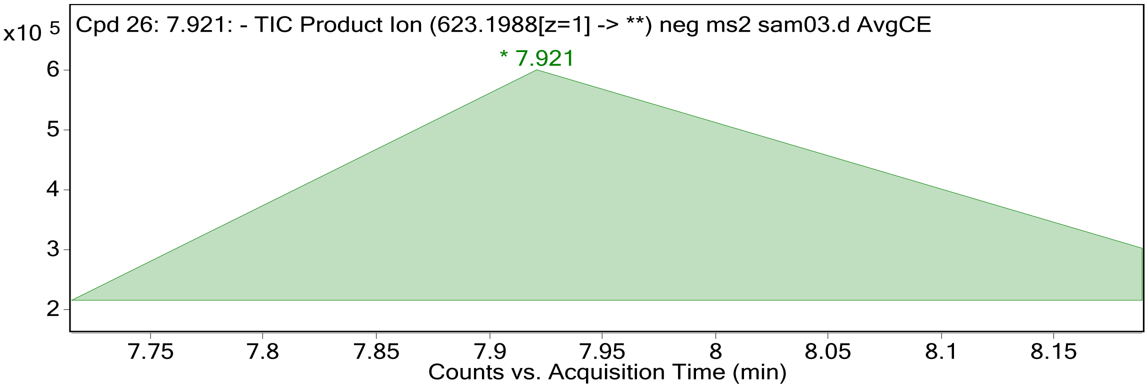

MSMS Spectrum

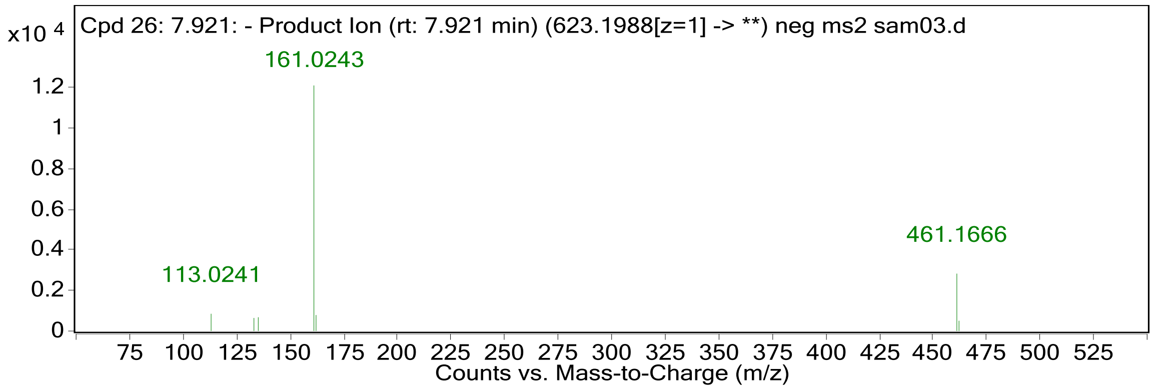

MSMS Spectrum

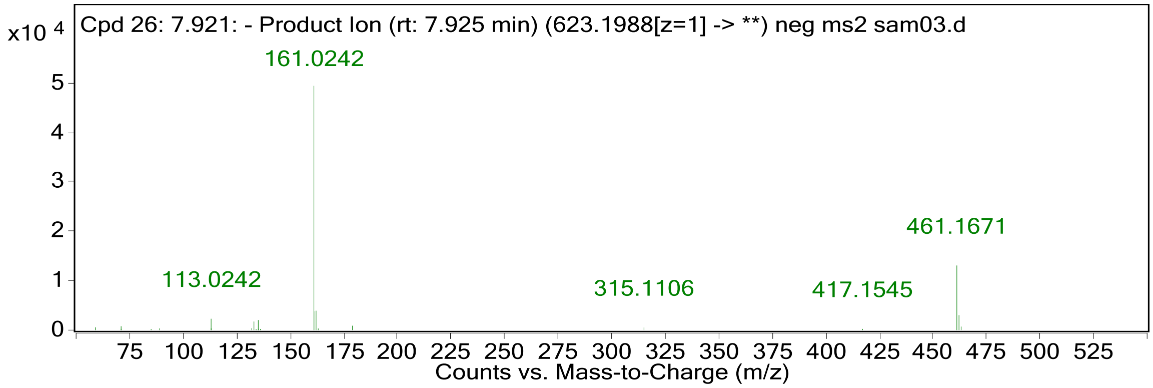

MSMS Spectrum

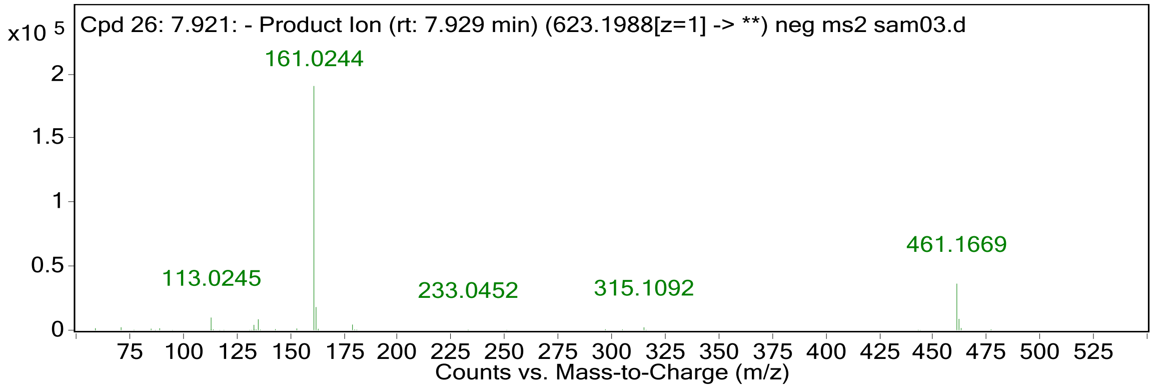

**Compound Label**	***m/z***	**RT**	**Algorithm**
Cpd 27: 7.995	377.1242	7.995	Targeted MS/MS

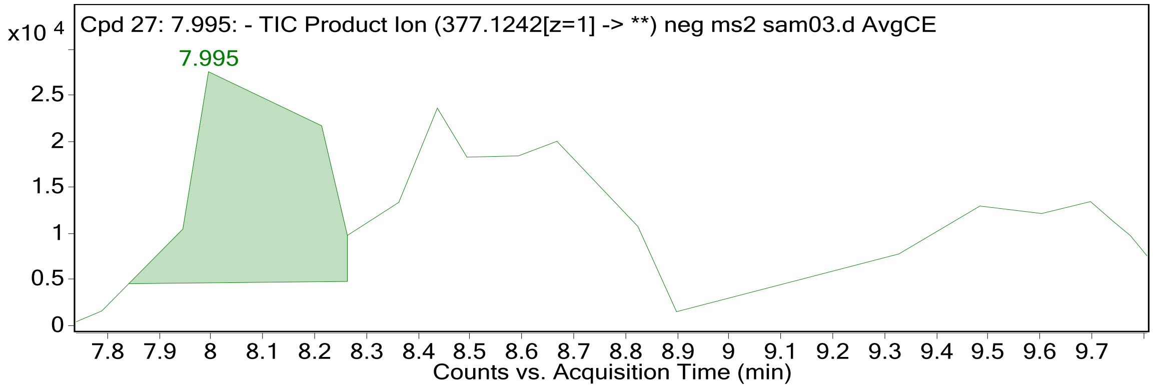

MSMS Spectrum

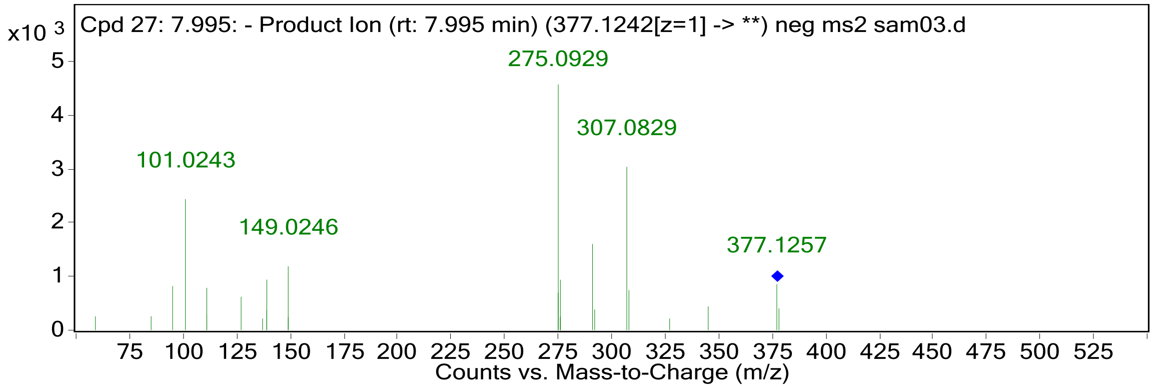

MSMS Spectrum

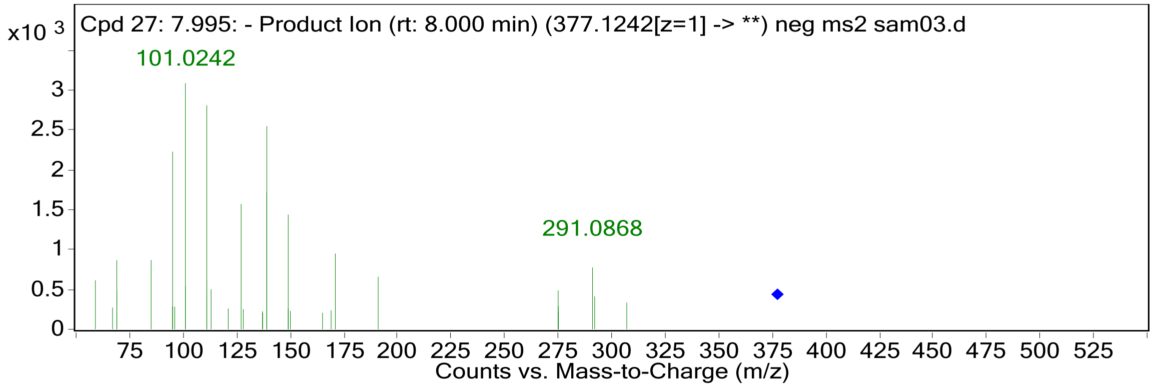

MSMS Spectrum

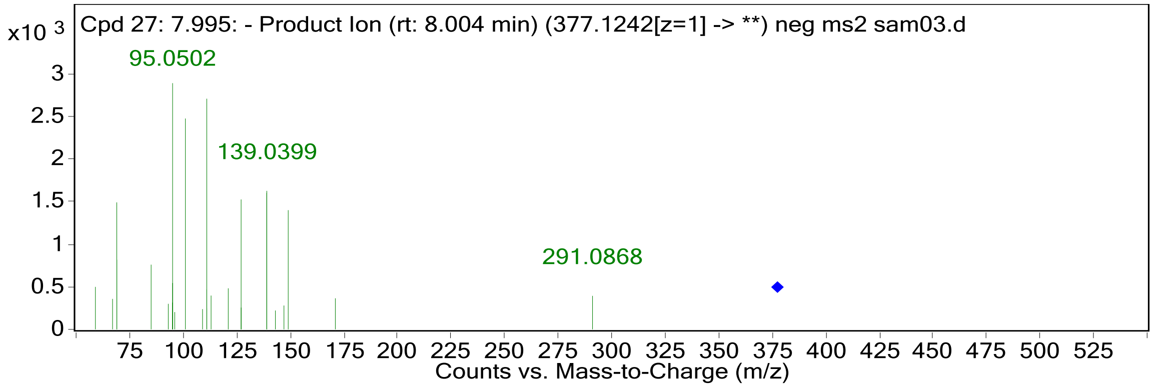

Qualitative Compound Report

Compound Label	*m/z*	RT	Algorithm
Cpd 28: 8.412	597.1832	8.412	Targeted MS/MS

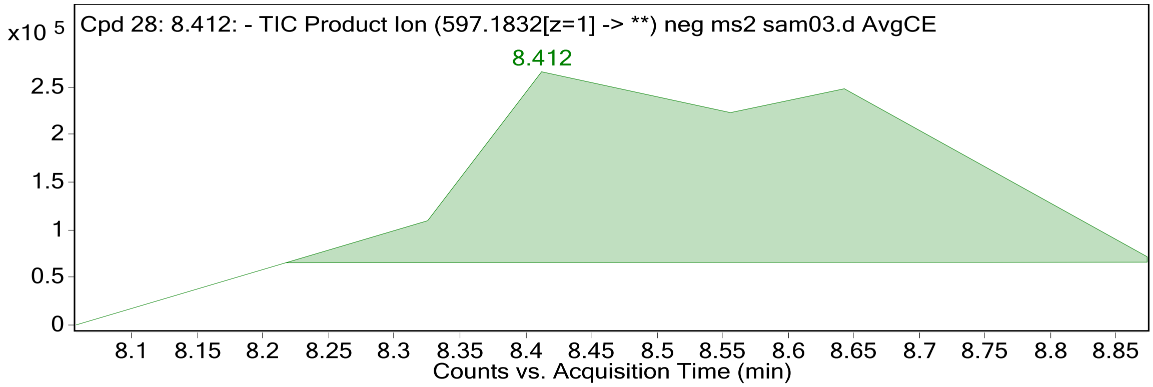

MSMS Spectrum

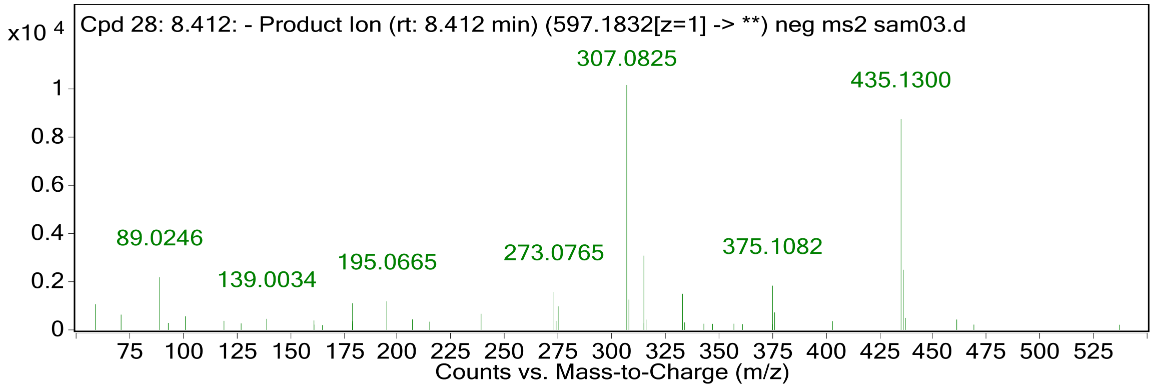

MSMS Spectrum

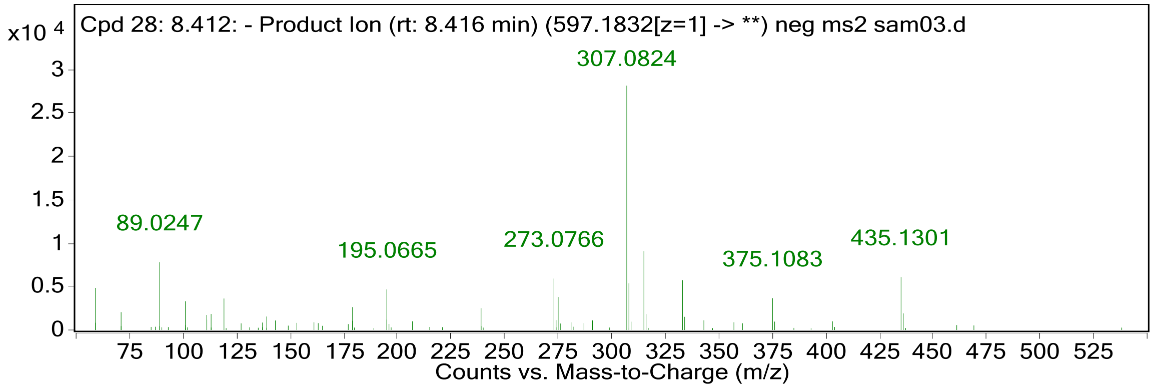

MSMS Spectrum

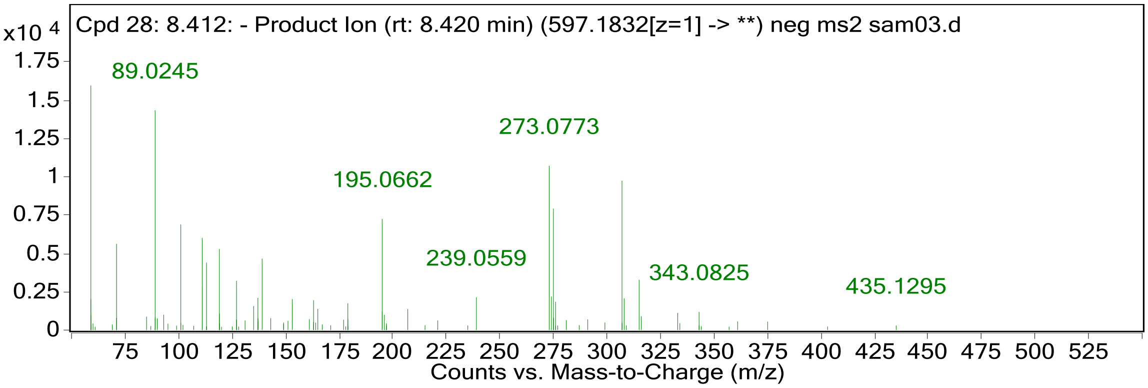

Compound Label	*m/z*	RT	Algorithm
Cpd 29: 8.449	539.1773	8.449	Targeted MS/MS

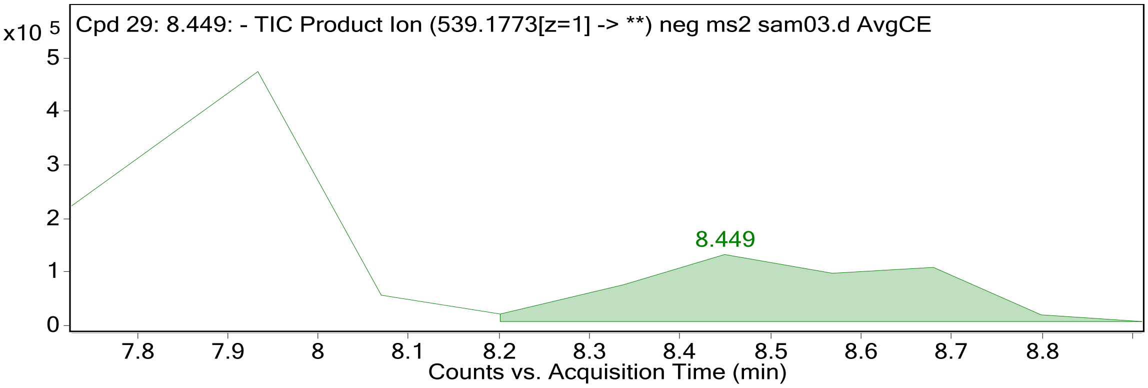

MSMS Spectrum

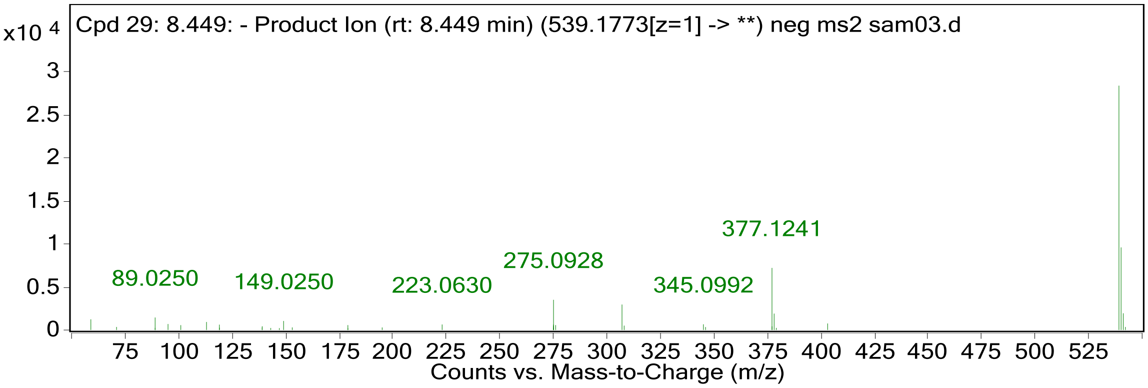

MSMS Spectrum

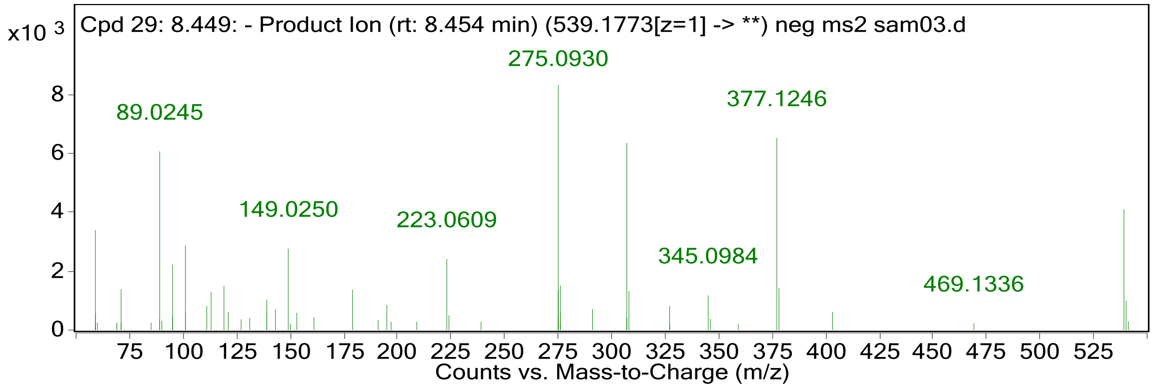

MSMS Spectrum

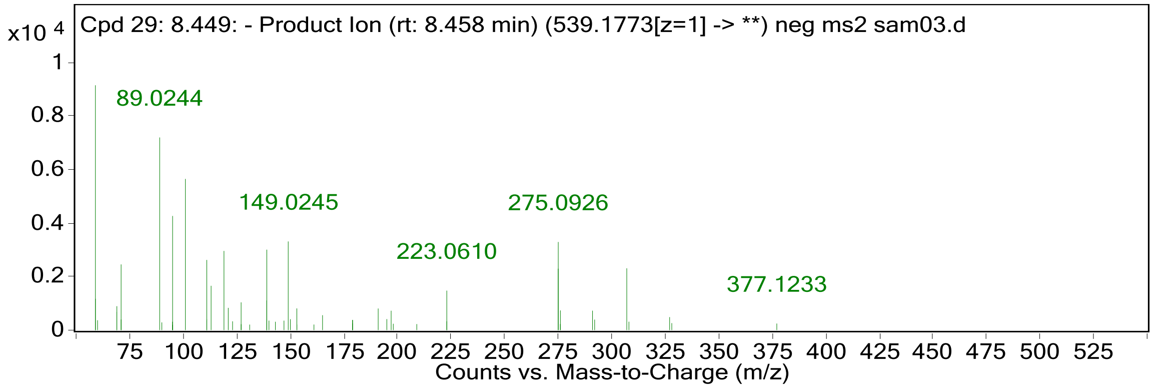

Compound Label	*m/z*	RT	Algorithm
Cpd 30: 8.544	693.2038	8.544	Targeted MS/MS

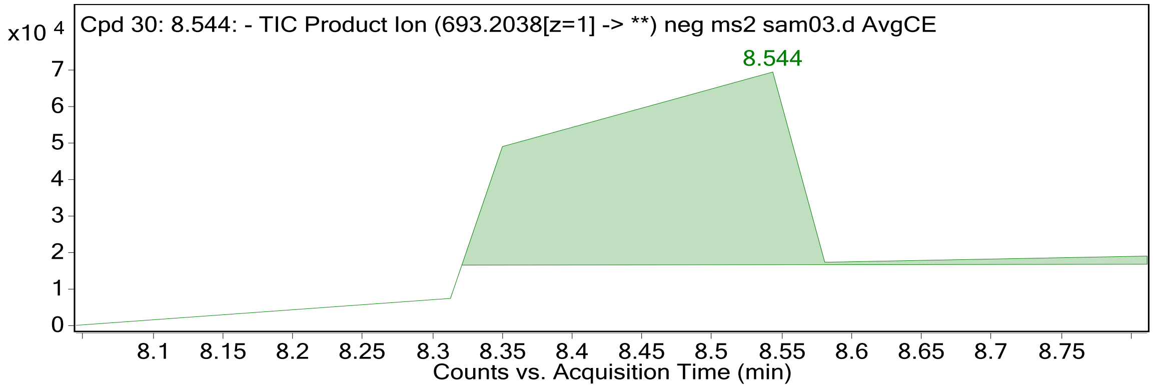

MSMS Spectrum

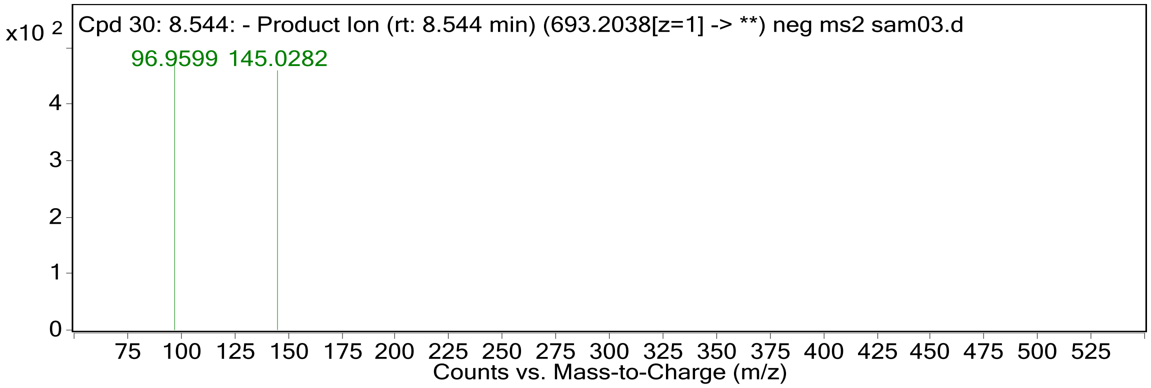

MSMS Spectrum

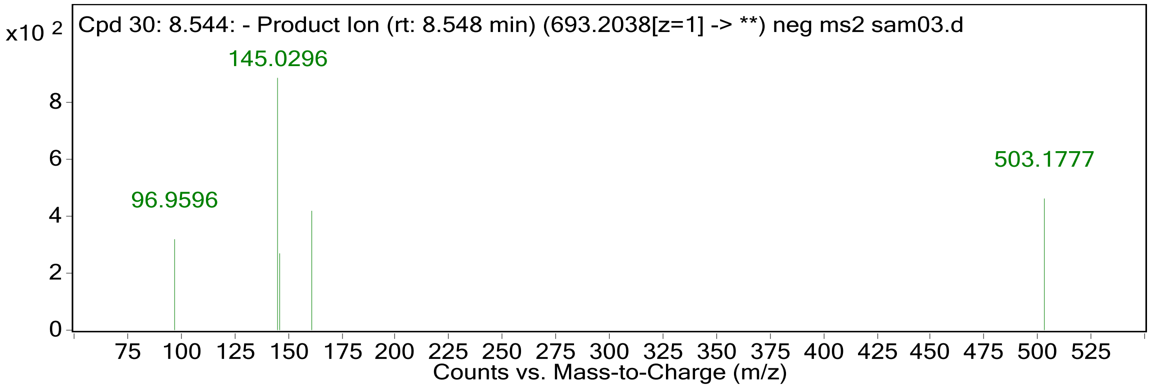

MSMS Spectrum

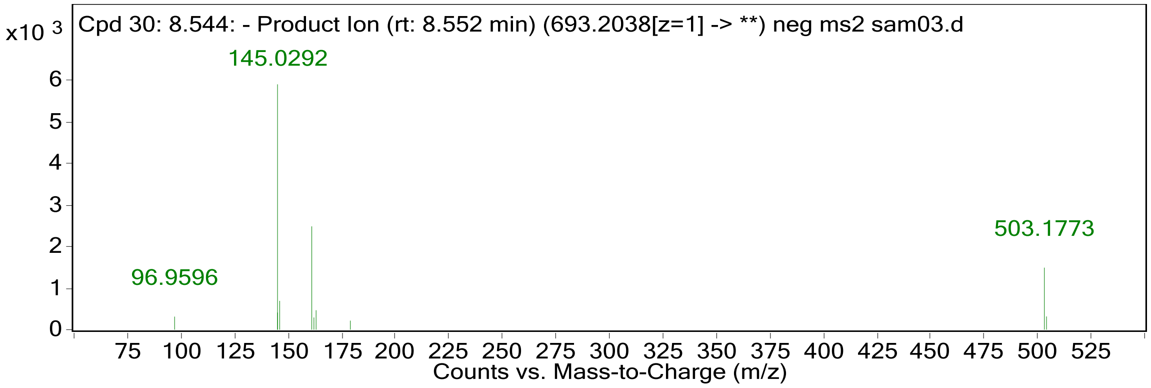

**Compound Label**	***m/z***	**RT**	**Algorithm**
Cpd 31: 8.606	591.2084	8.606	Targeted MS/MS

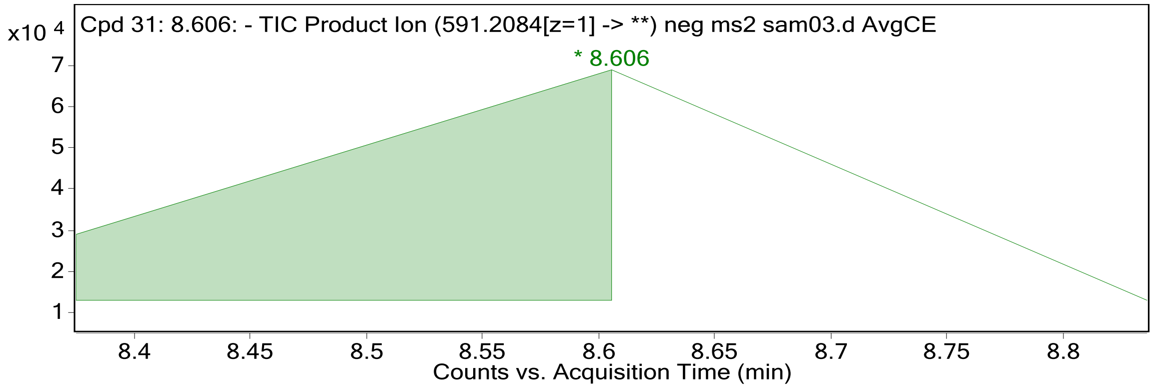

MSMS Spectrum

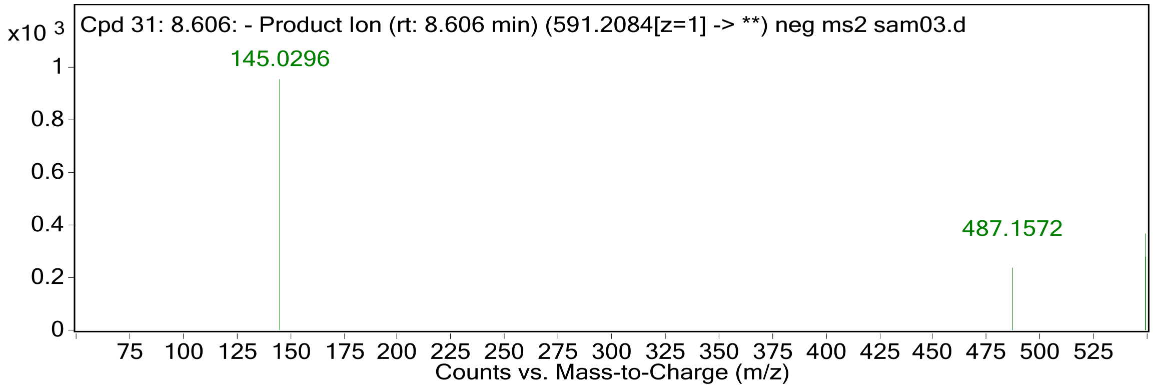

MSMS Spectrum

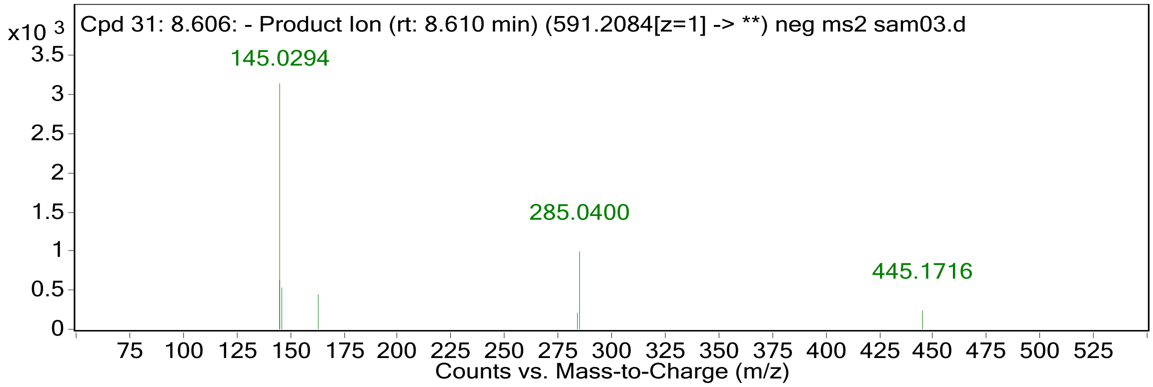

MSMS Spectrum

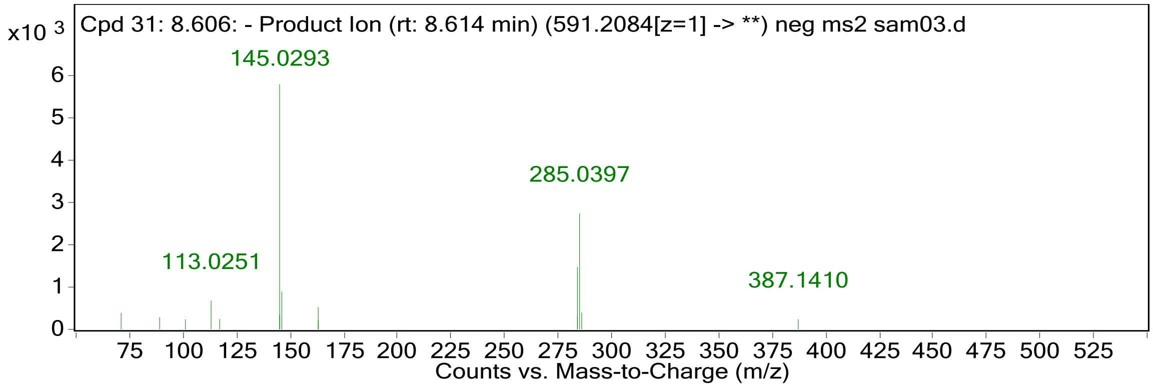

Compound Label	*m/z*	RT	Algorithm
Cpd 32: 8.618	435.1296	8.618	Targeted MS/MS

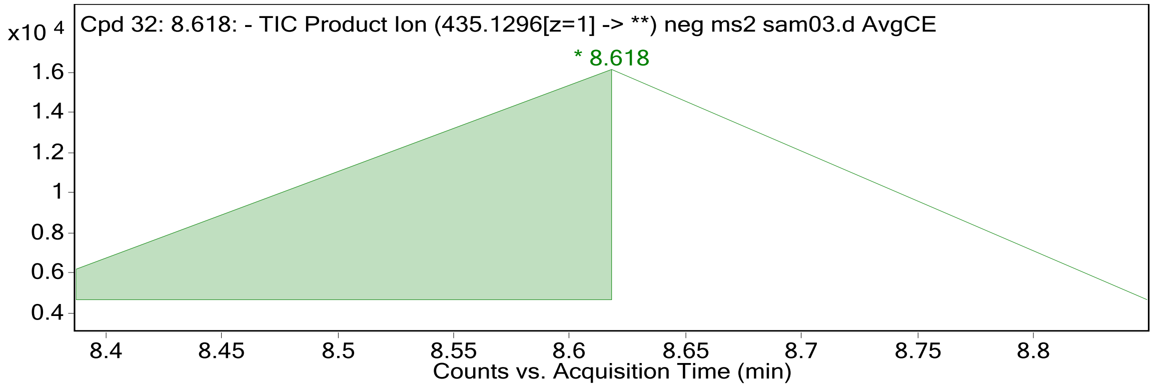

MSMS Spectrum

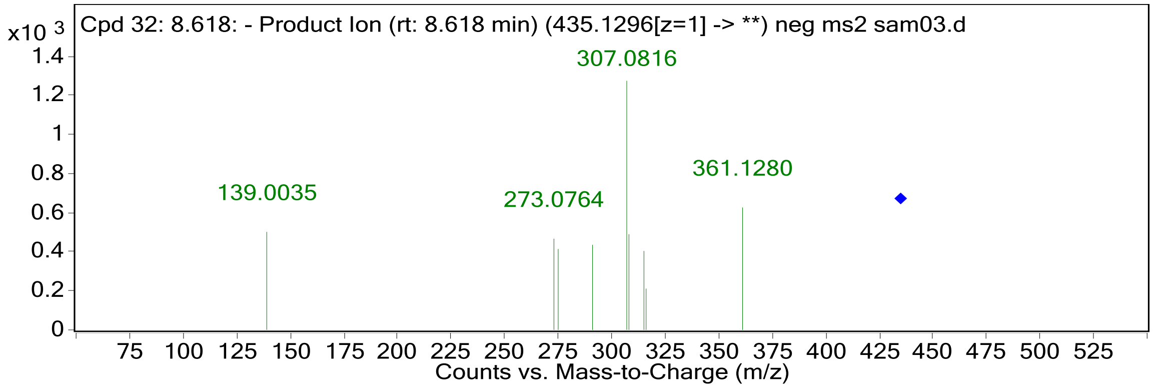

MSMS Spectrum

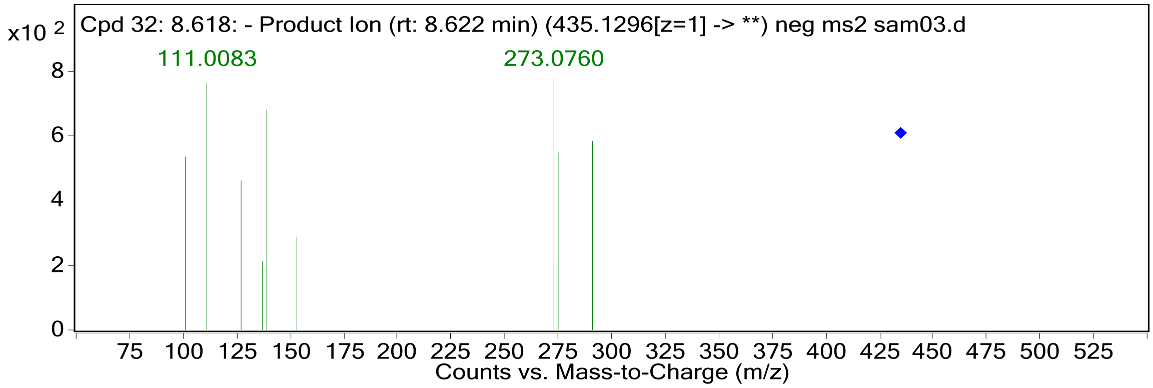

MSMS Spectrum

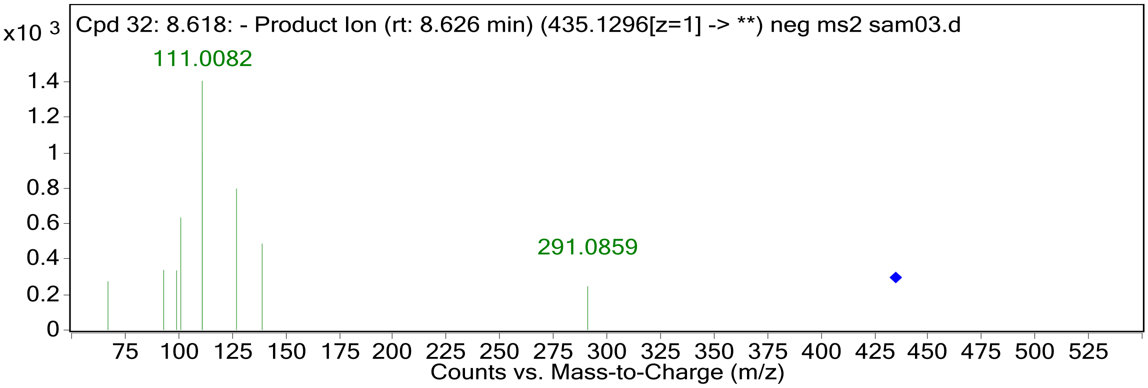

Compound Label	*m/z*	RT	Algorithm
Cpd 33: 8.668	377.1242	8.668	Targeted MS/MS

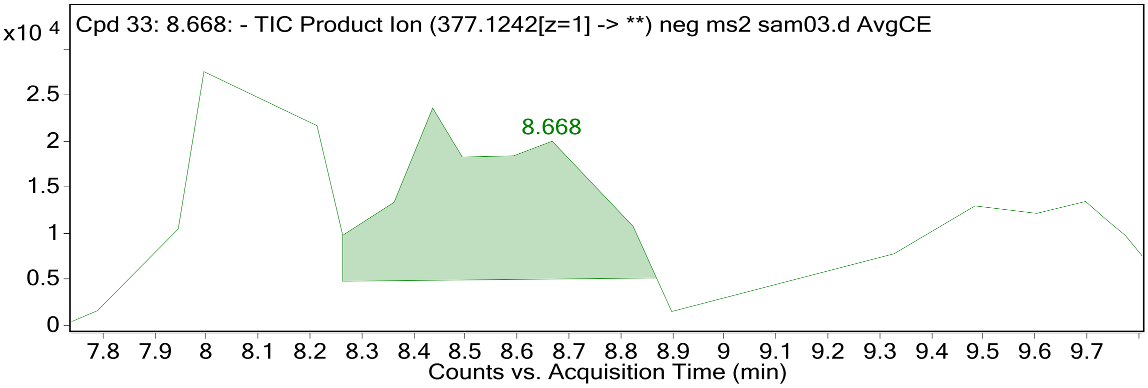

MSMS Spectrum

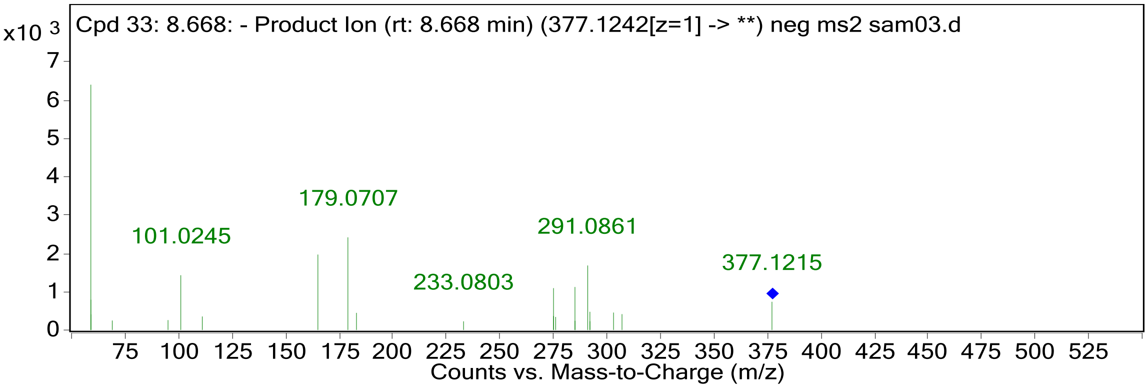

MSMS Spectrum

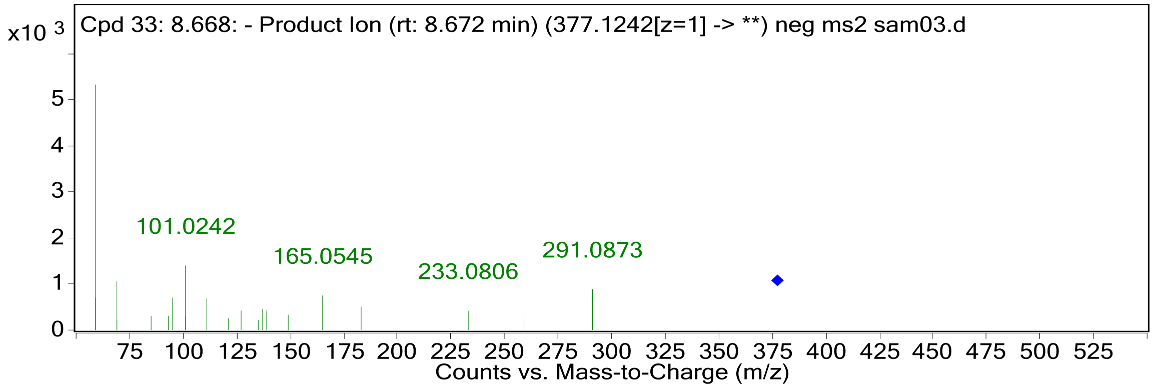

MSMS Spectrum

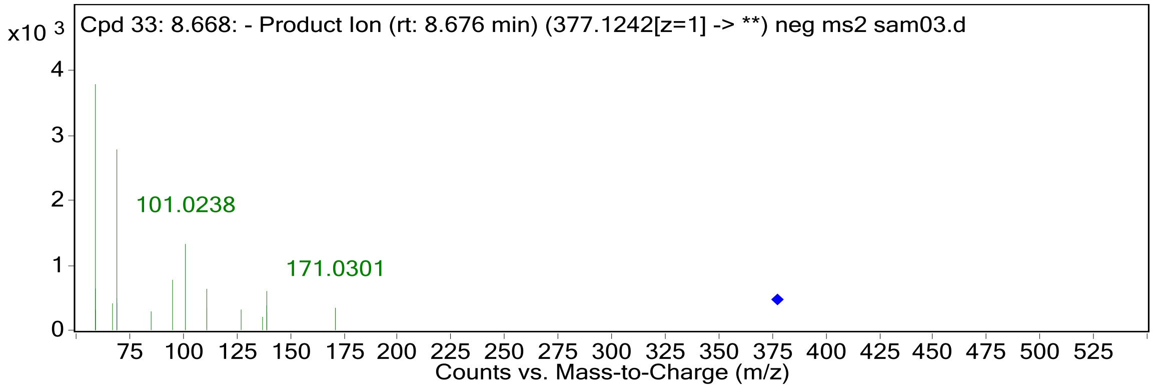

Compound Label	*m/z*	RT	Algorithm
Cpd 34: 8.886	1009.3201	8.886	Targeted MS/MS

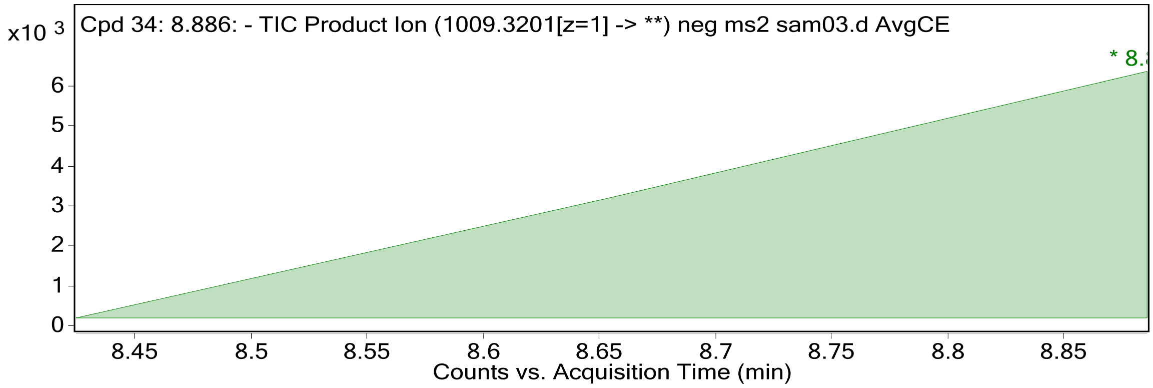

MSMS Spectrum

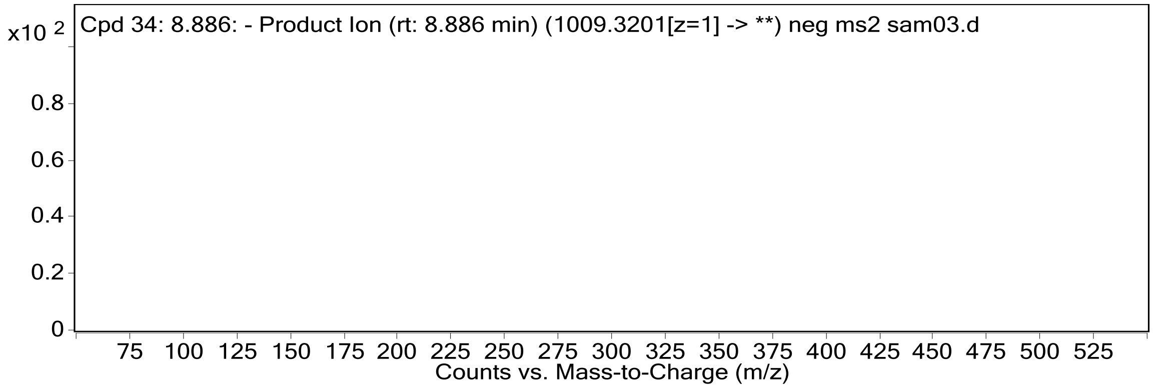

MSMS Spectrum

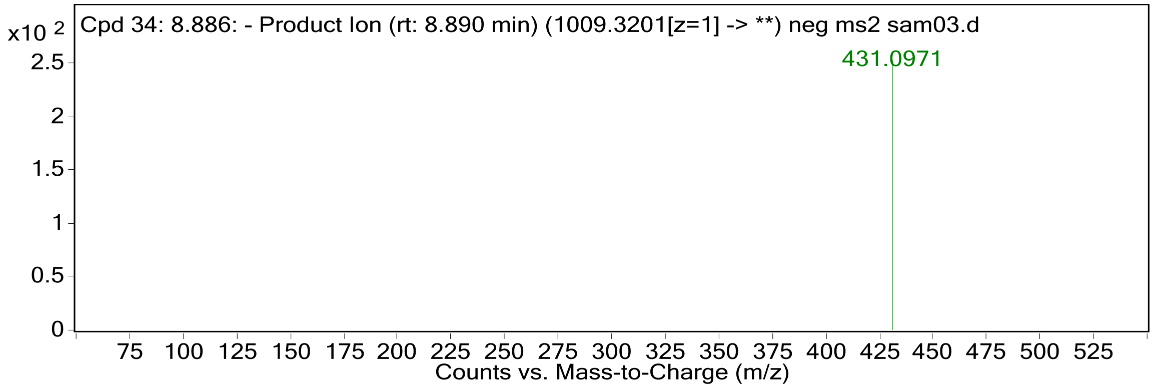

MSMS Spectrum

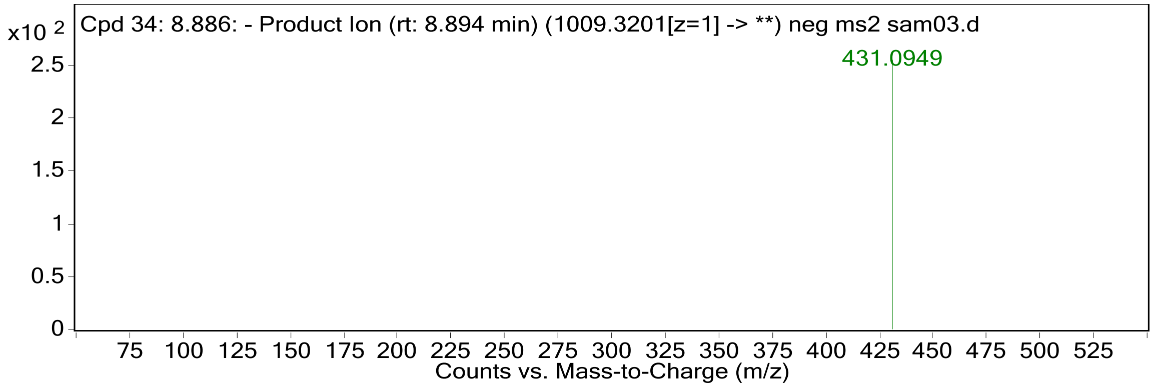

Compound Label	*m/z*	RT	Algorithm
Cpd 35: 8.923	531.1509	8.923	Targeted MS/MS

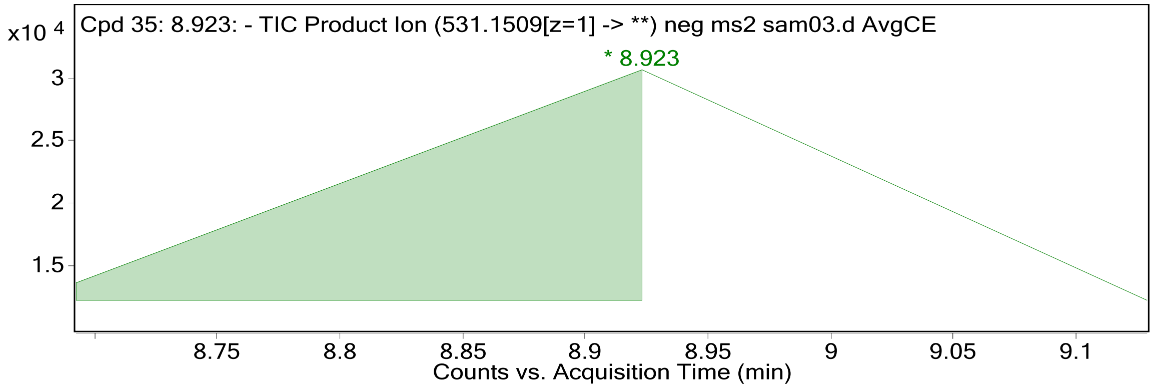

x10 4
Cpd 35: 8.923: - TIC Product Ion (531.1509[z=1] -> **) neg ms2 sam03.d AvgCE
* 8.923
3
2.5
2
1.5
8.75
8.8
8.85
8.9
8.95
9
9.05
9.1
Counts vs. Acquisition Time (min)

MSMS Spectrum

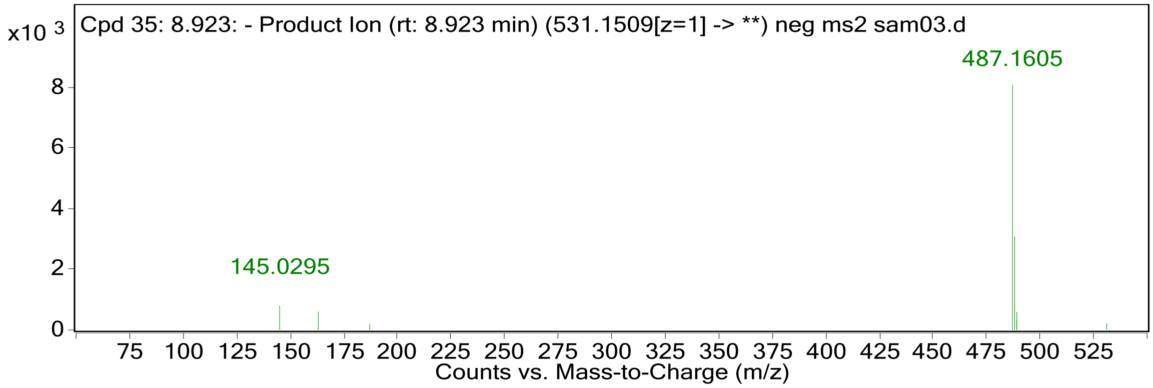

MSMS Spectrum

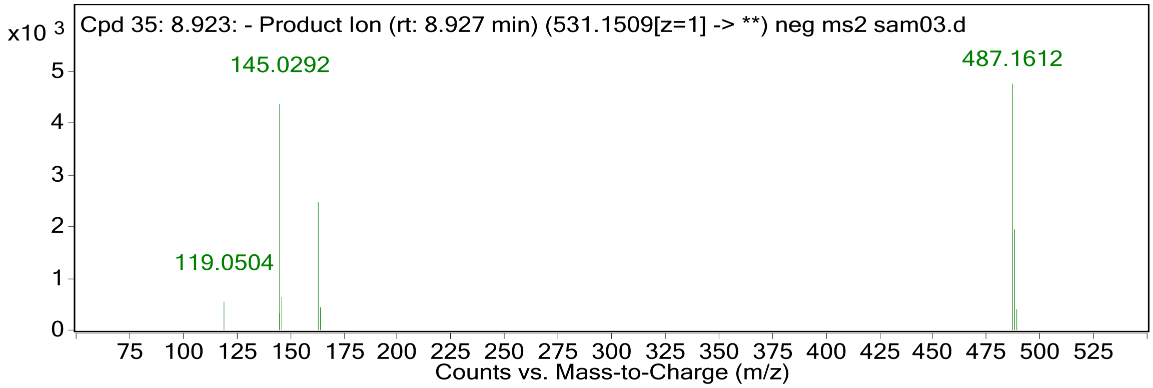

MSMS Spectrum

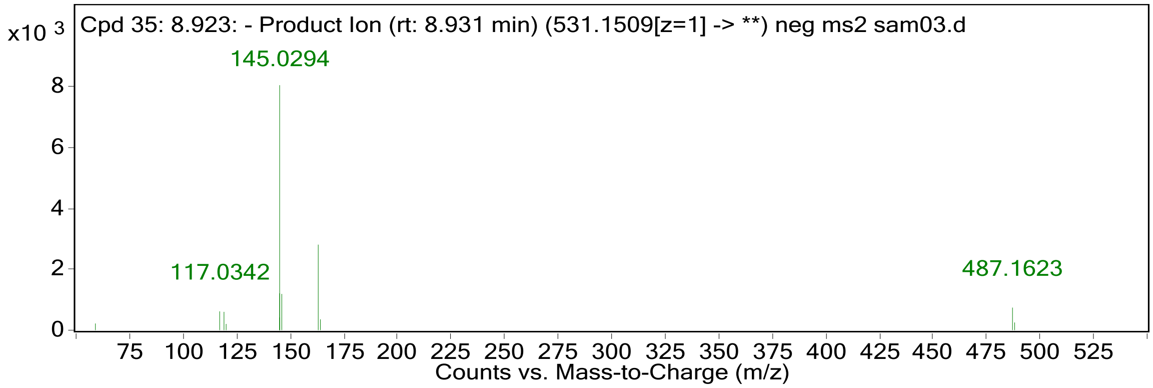

**Compound Label**	***m/z***	**RT**	**Algorithm**
Cpd 36: 8.936	487.1613	8.936	Targeted MS/MS

Qualitative Compound Report

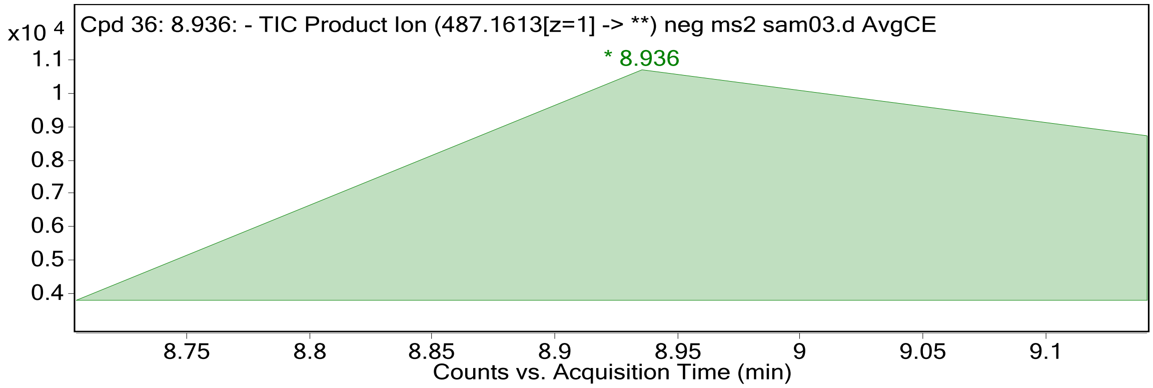

MSMS Spectrum

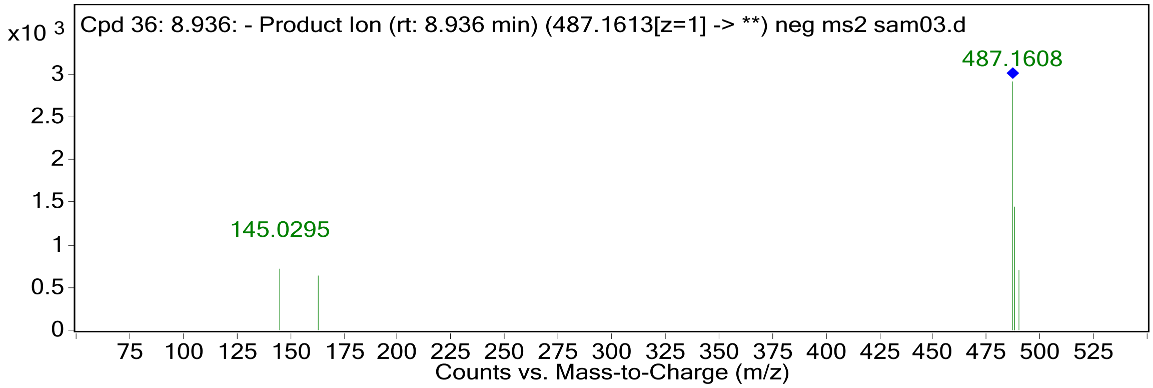

MSMS Spectrum

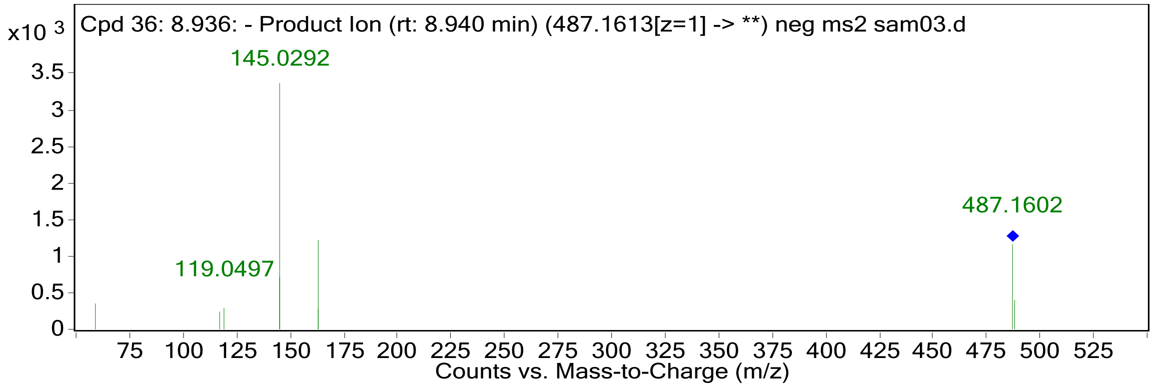

MSMS Spectrum

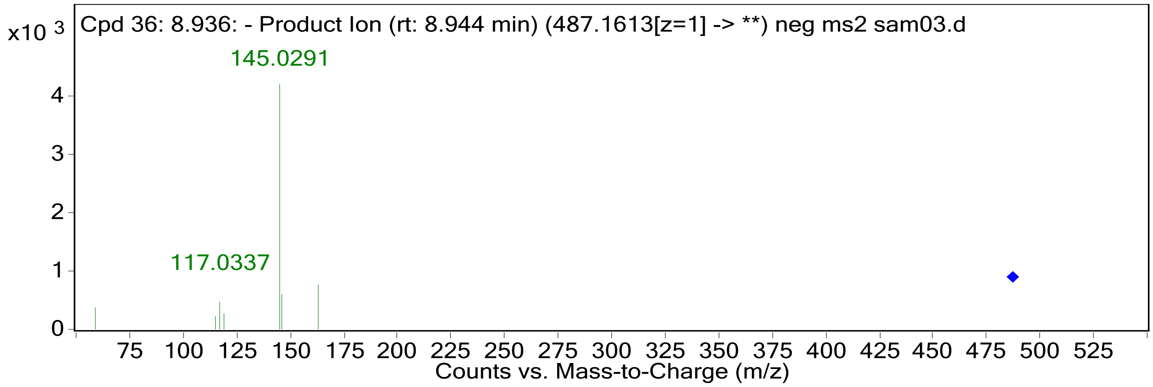

Compound Label	*m/z*	RT	Algorithm
Cpd 37: 8.960	677.209	8.96	Targeted MS/MS

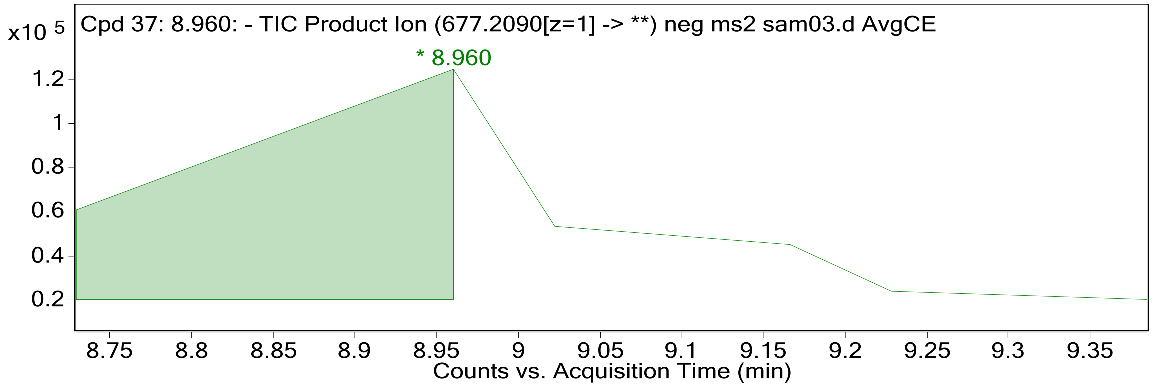

MSMS Spectrum

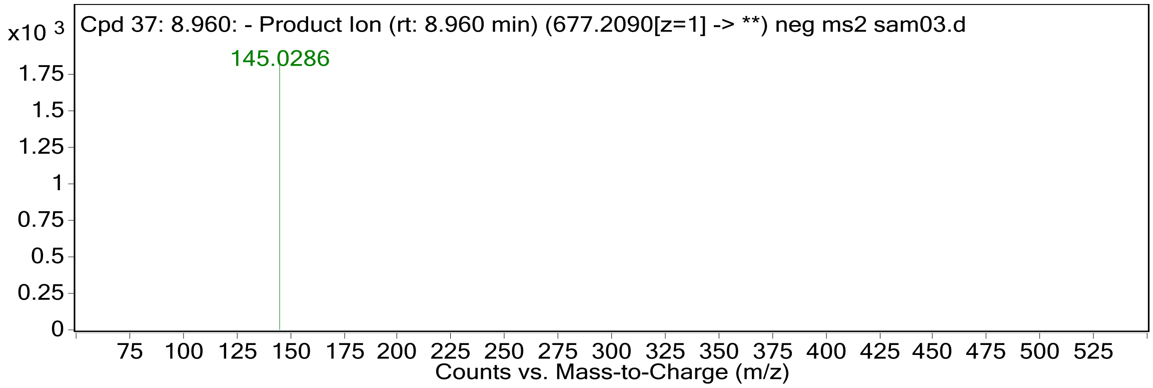

MSMS Spectrum

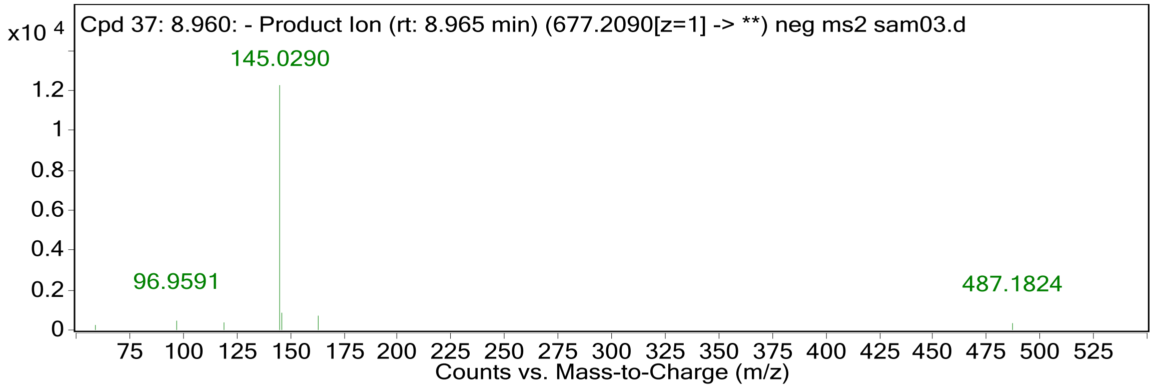

MSMS Spectrum

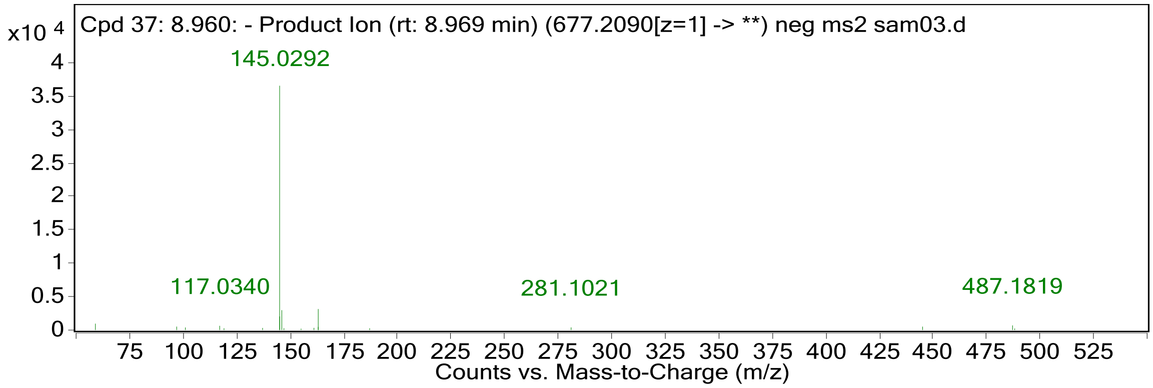

Compound Label	*m/z*	RT	Algorithm
Cpd 38: 9.084	601.2141	9.084	Targeted MS/MS

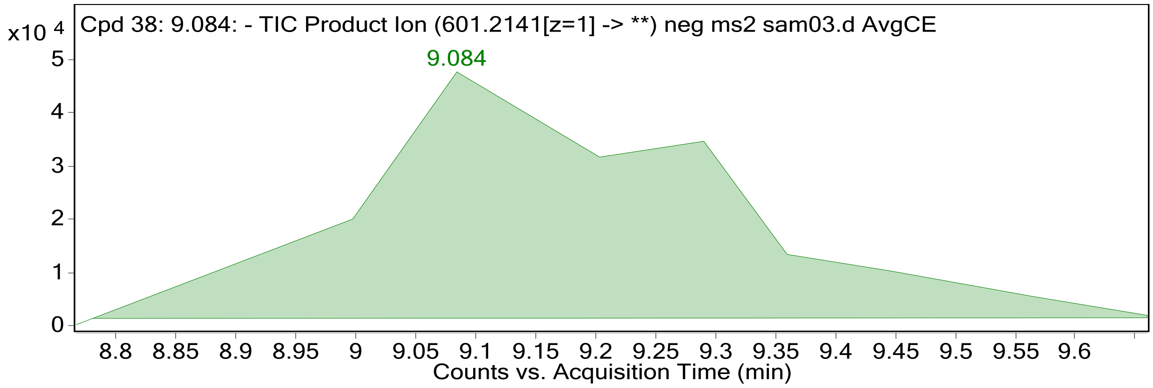

MSMS Spectrum

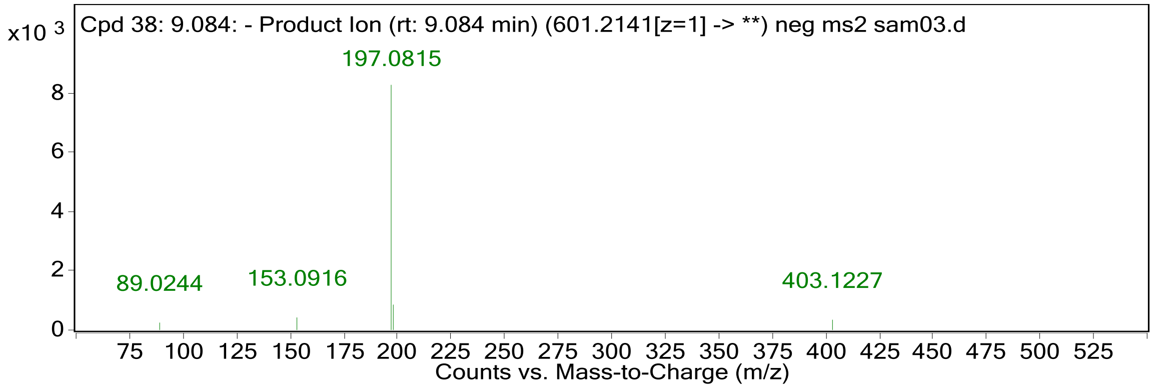

MSMS Spectrum

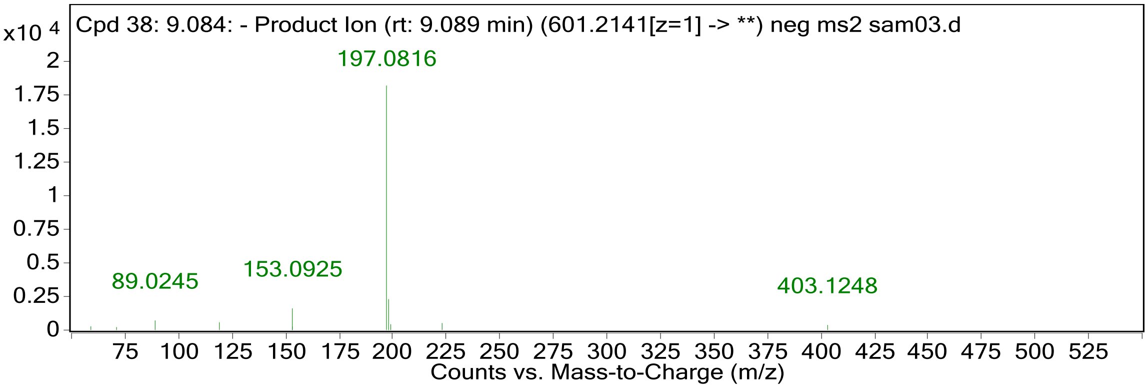

MSMS Spectrum

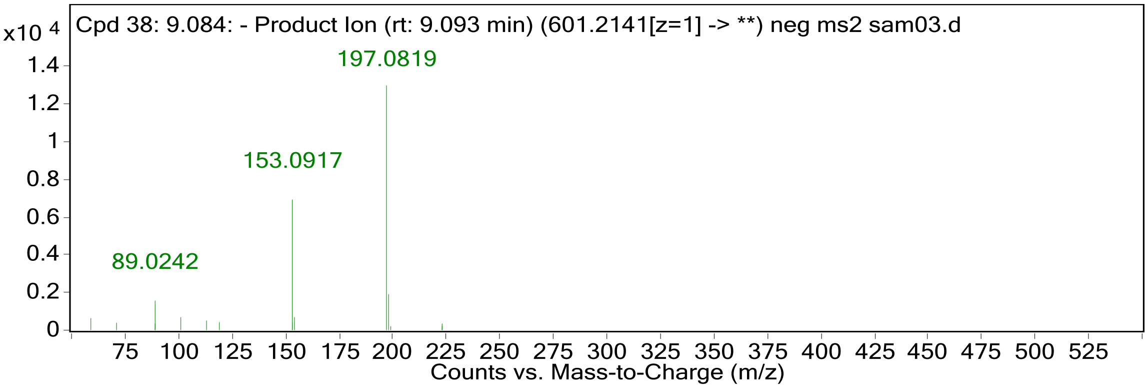

Compound Label	*m/z*	RT	Algorithm
Cpd 39: 9.154	579.2087	9.154	Targeted MS/MS

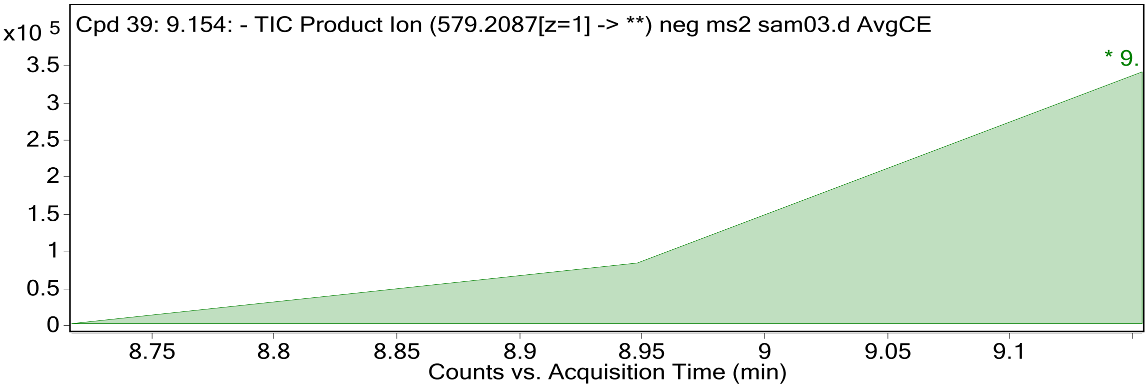

MSMS Spectrum

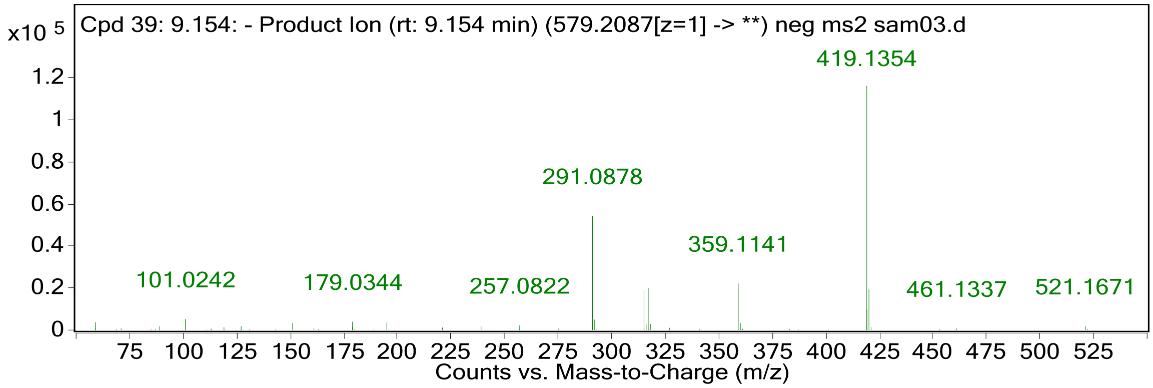

MSMS Spectrum

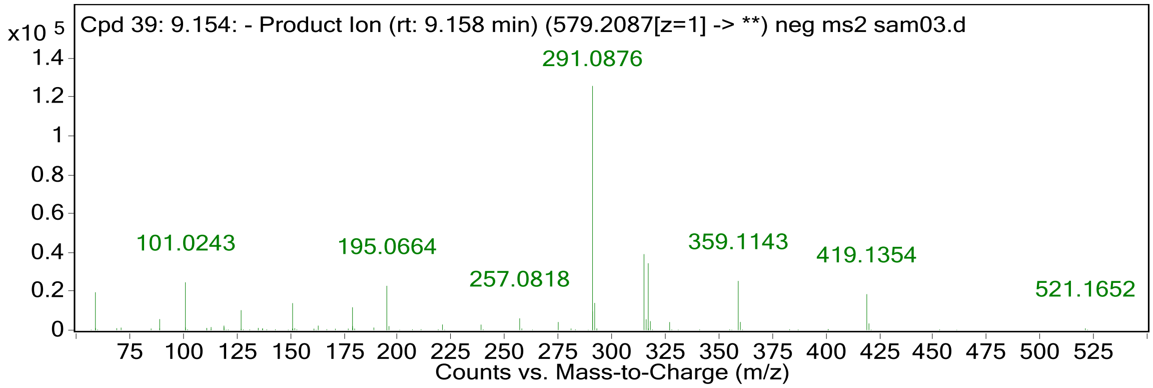

MSMS Spectrum

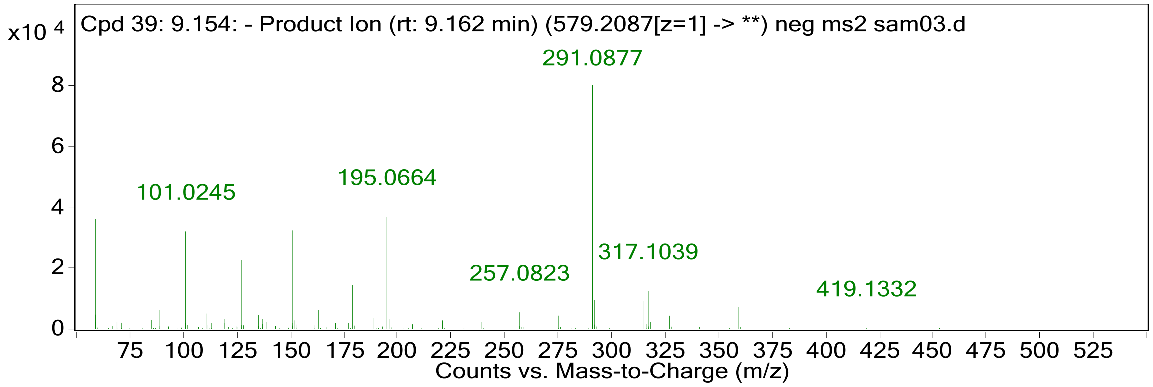

Compound Label	*m/z*	RT	Algorithm
Cpd 40: 9.253	471.1874	9.253	Targeted MS/MS

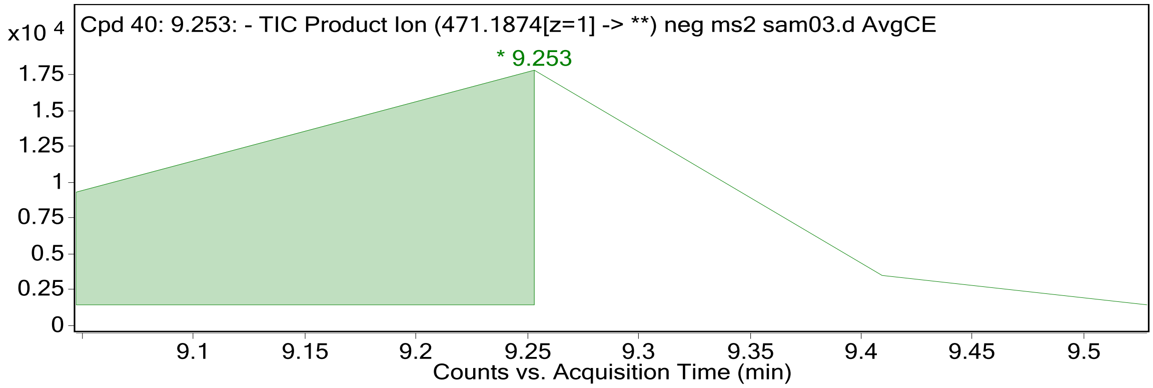

MSMS Spectrum

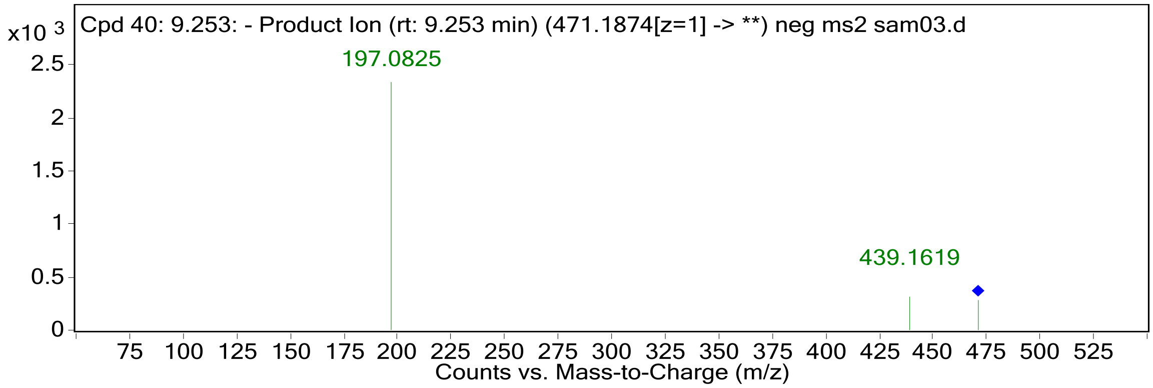

MSMS Spectrum

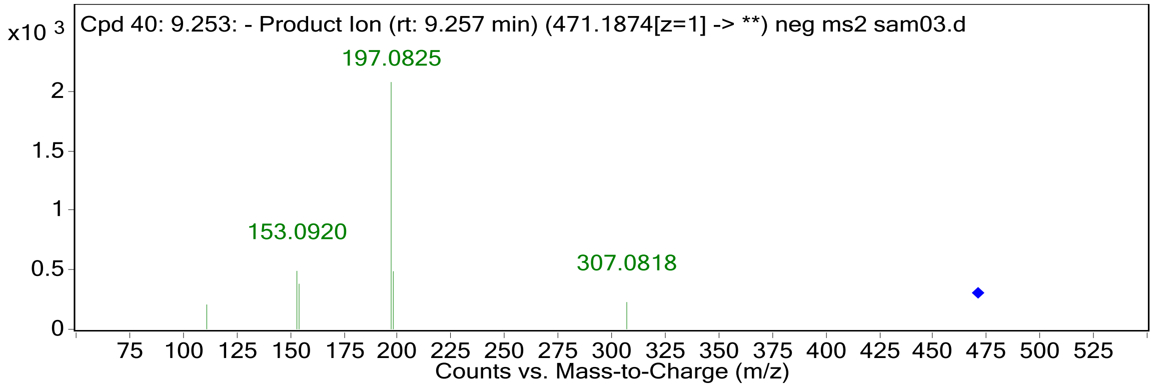

MSMS Spectrum

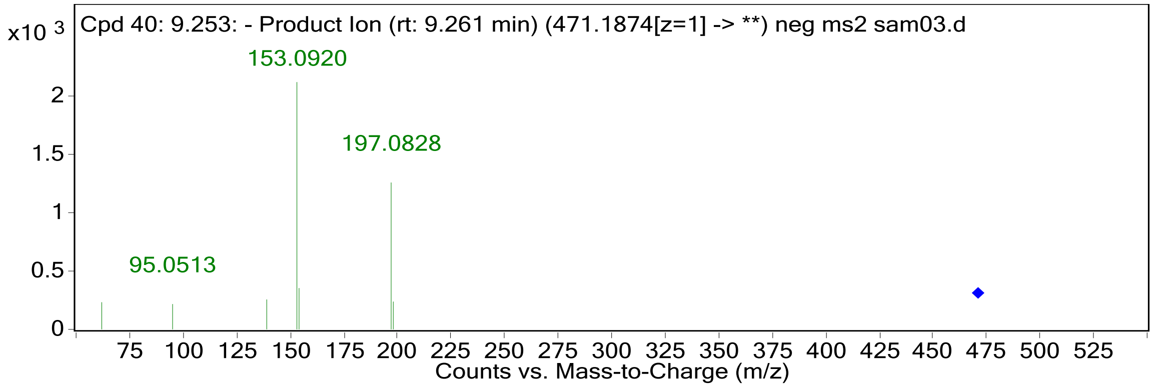

Qualitative Compound Report

Compound Label	*m/z*	RT	Algorithm
Cpd 41: 9.303	523.1825	9.303	Targeted MS/MS

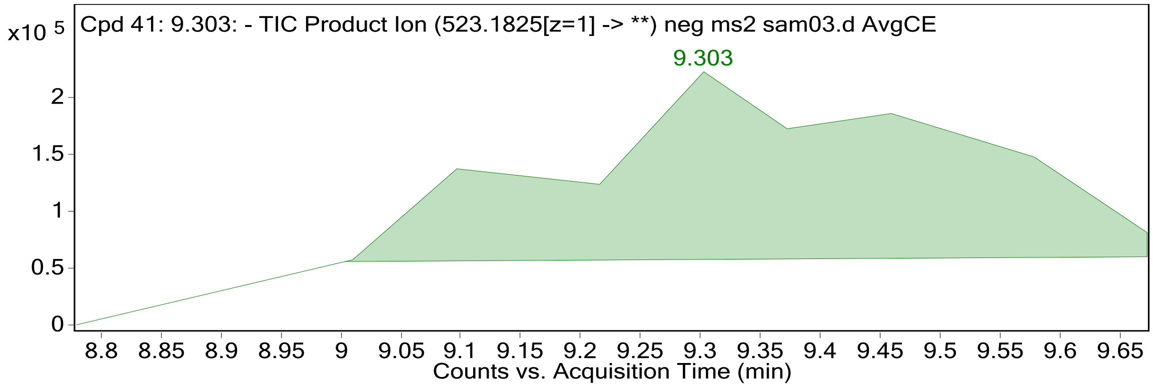

MSMS Spectrum

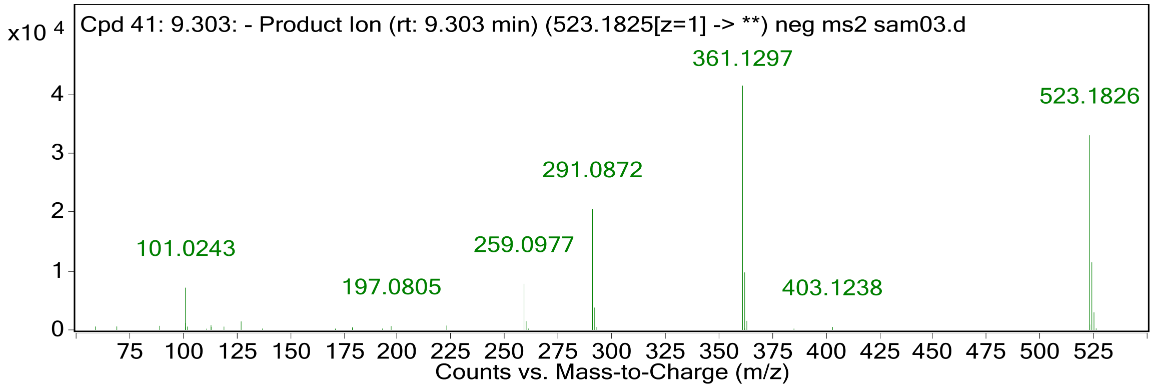

MSMS Spectrum

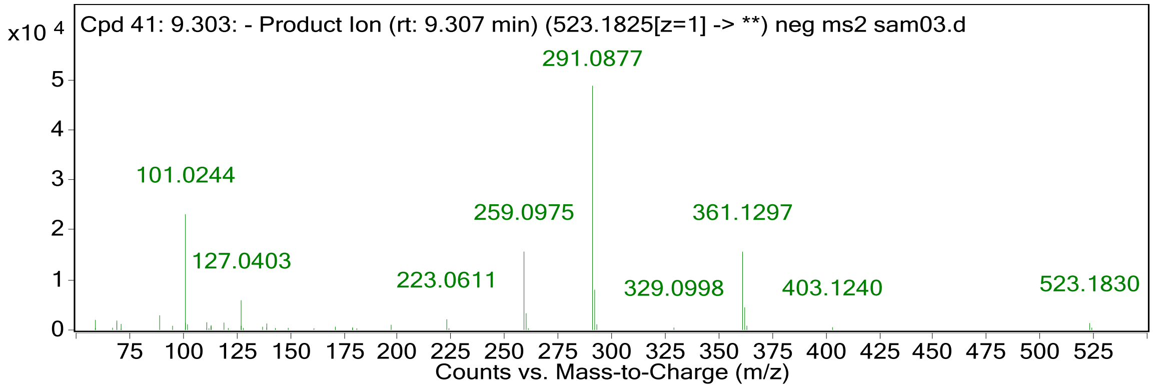

MSMS Spectrum

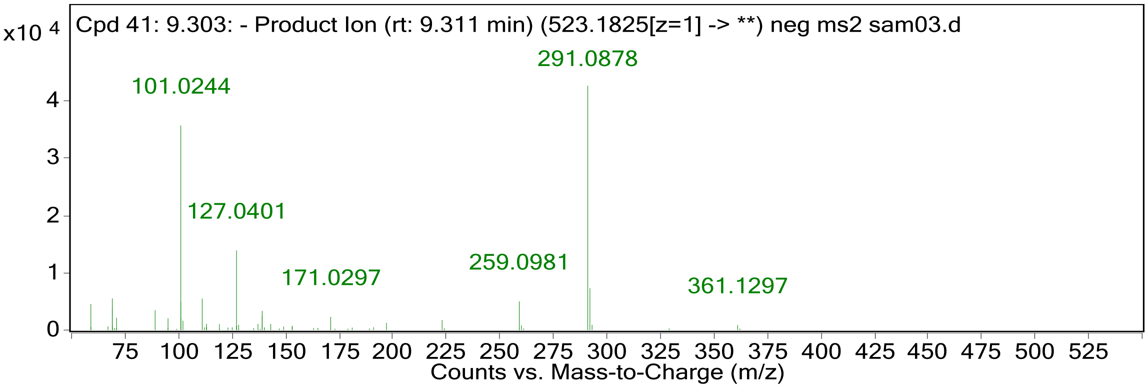

Compound Label	*m/z*	RT	Algorithm
Cpd 42: 9.340	581.1884	9.34	Targeted MS/MS

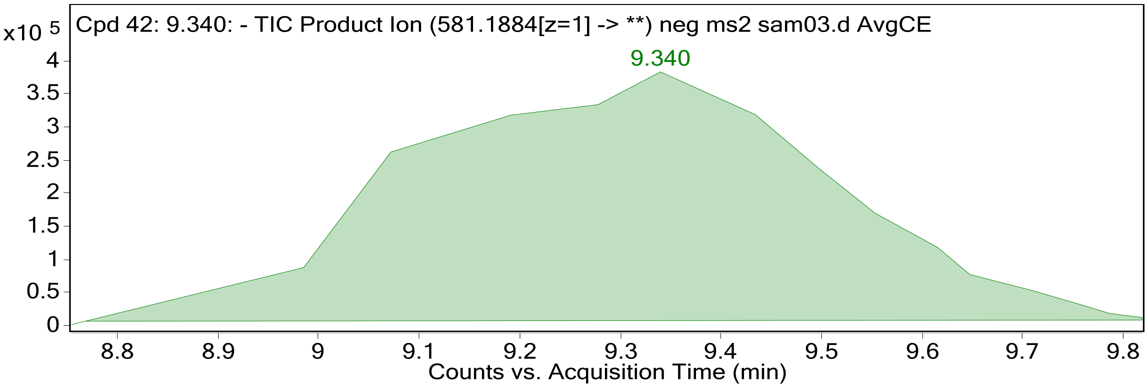

MSMS Spectrum

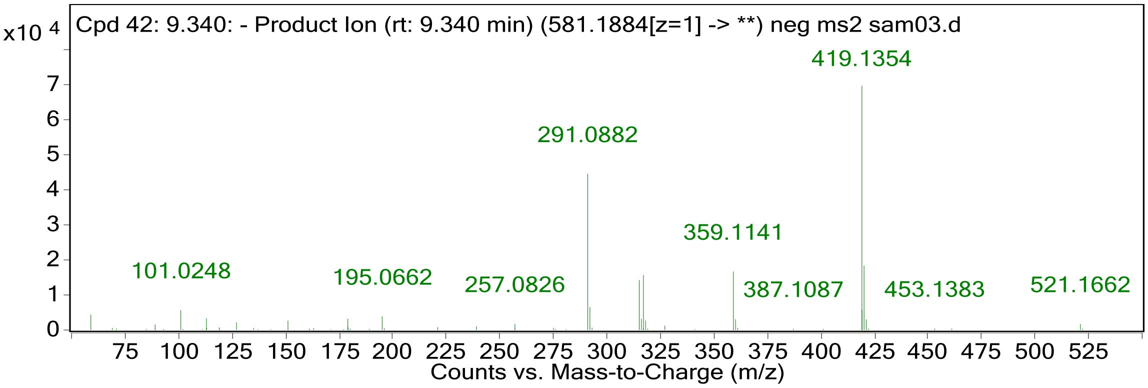

MSMS Spectrum

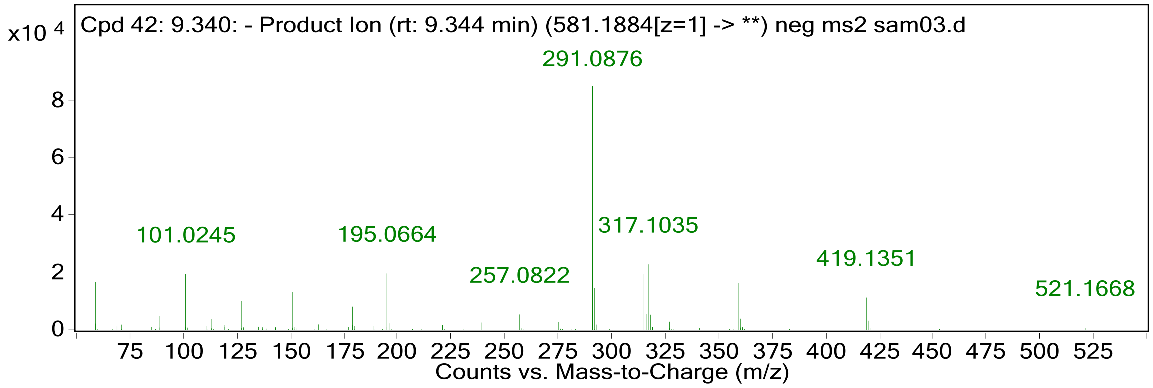

MSMS Spectrum

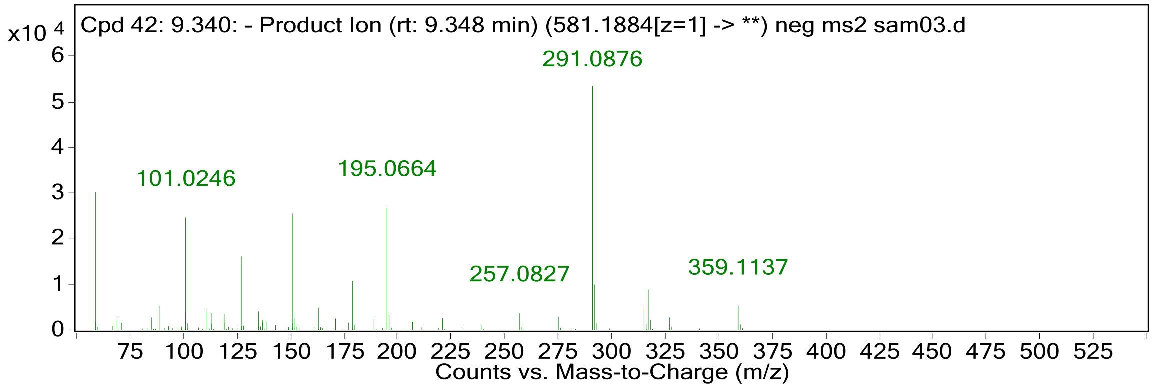

Compound Label	*m/z*	RT	Algorithm
Cpd 43: 9.397	361.1293	9.397	Targeted MS/MS

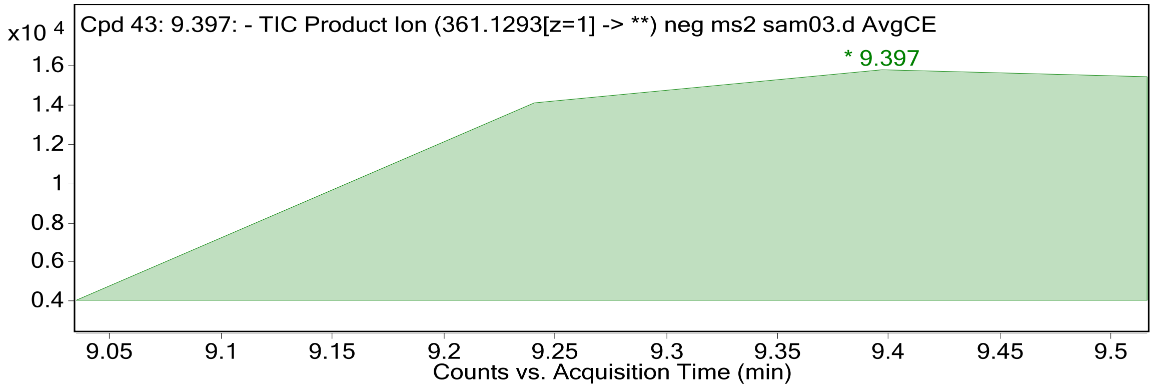

MSMS Spectrum

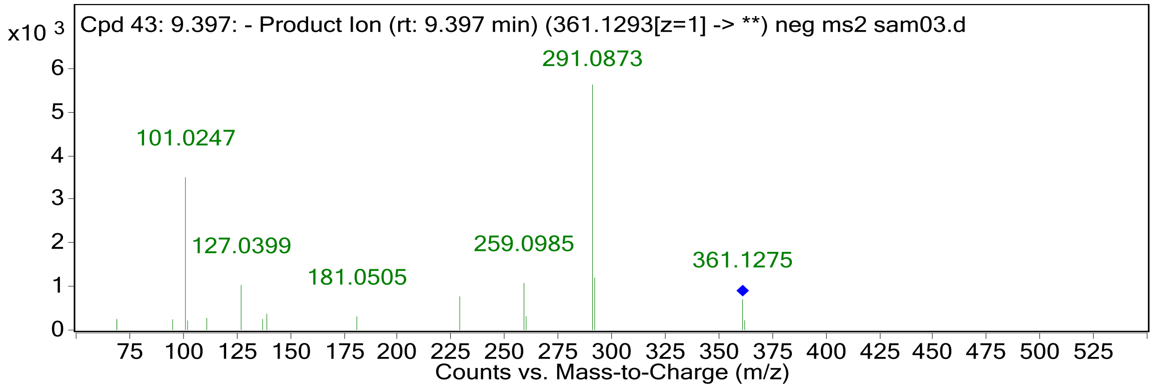

MSMS Spectrum

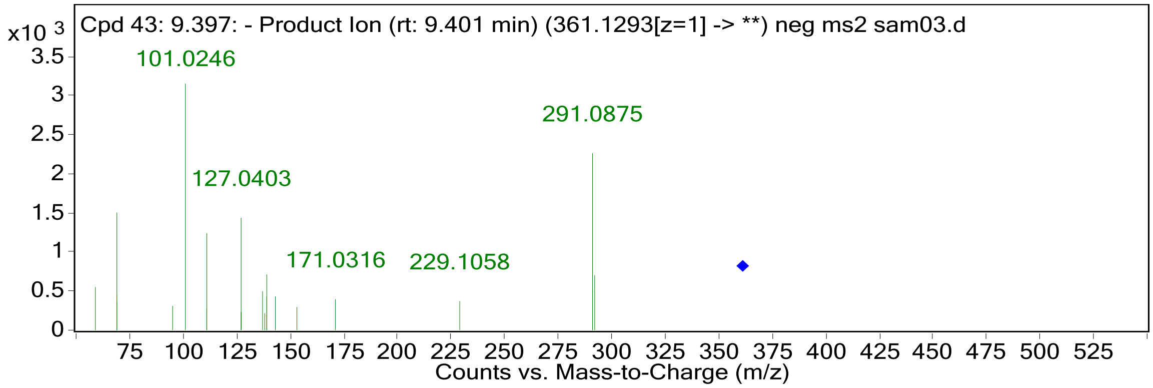

MSMS Spectrum

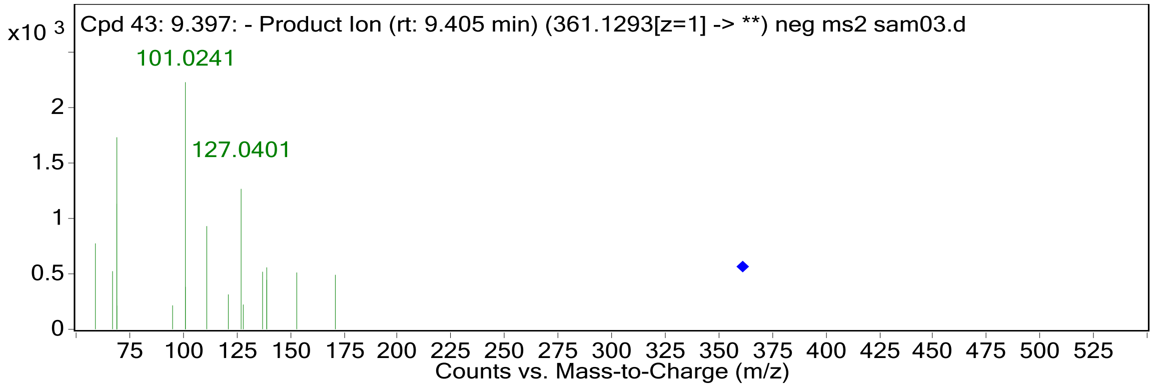

**Compound Label**	***m/z***	**RT**	**Algorithm**
Cpd 44: 9.697	377.1242	9.697	Targeted MS/MS

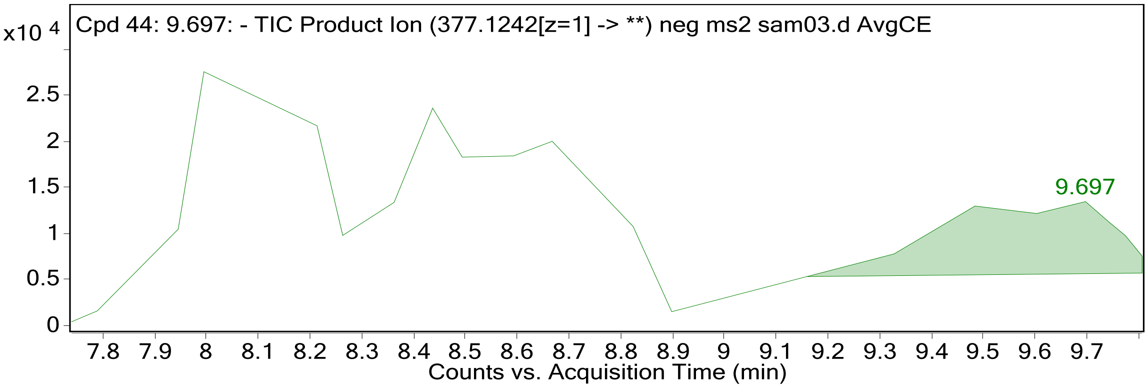

Cpd 44: 9.697: - TIC Product Ion (377.1242[z=1] -> **) neg ms2 sam03.d AvgCE
x10 4
2.5
2
1.5
1
0.5
0
9.697
7.8 7.9 8 8.1 8.2 8.3 8.4 8.5 8.6 8.7 8.8 8.9 9 9.1 9.2 9.3 9.4 9.5 9.6 9.7
Counts vs. Acquisition Time (min)

MSMS Spectrum

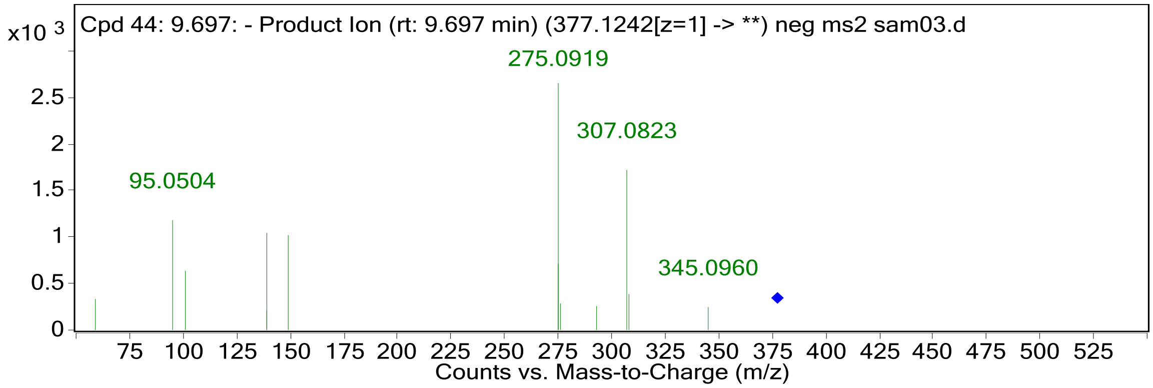

MSMS Spectrum

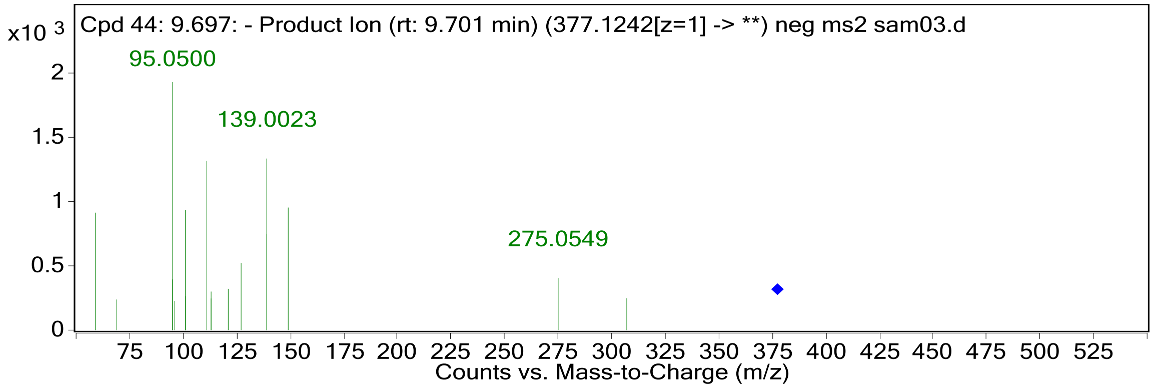

MSMS Spectrum

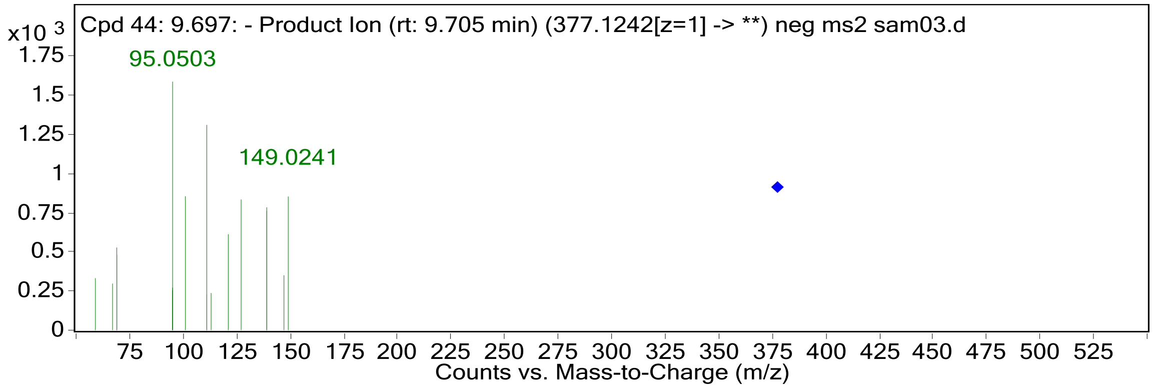

**Compound Label**	***m/z***	**RT**	**Algorithm**
Cpd 45: 12.010	219.1754	12.01	Targeted MS/MS

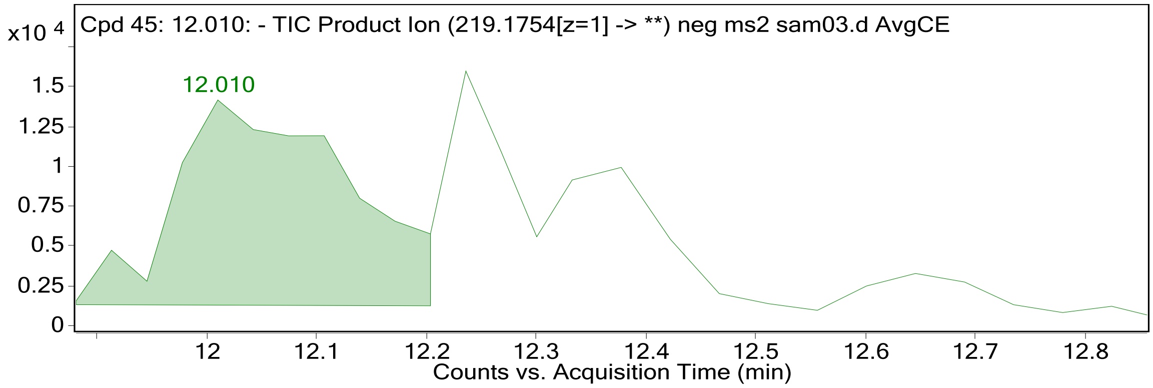

**Compound Label**	***m/z***	**RT**	**Algorithm**
Cpd 46: 12.236	219.1754	12.236	Targeted MS/MS

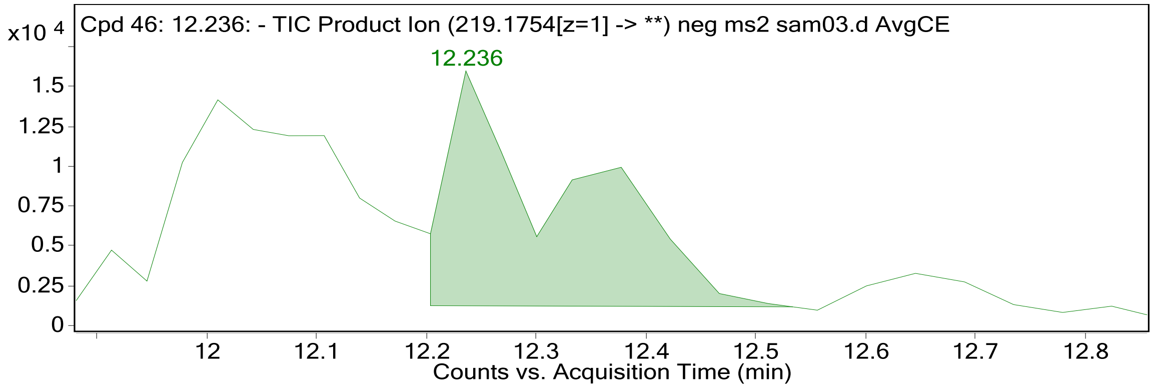

**Compound Label**	***m/z***	**RT**	**Algorithm**
Cpd 47: 12.321	487.3429	12.321	Targeted MS/MS

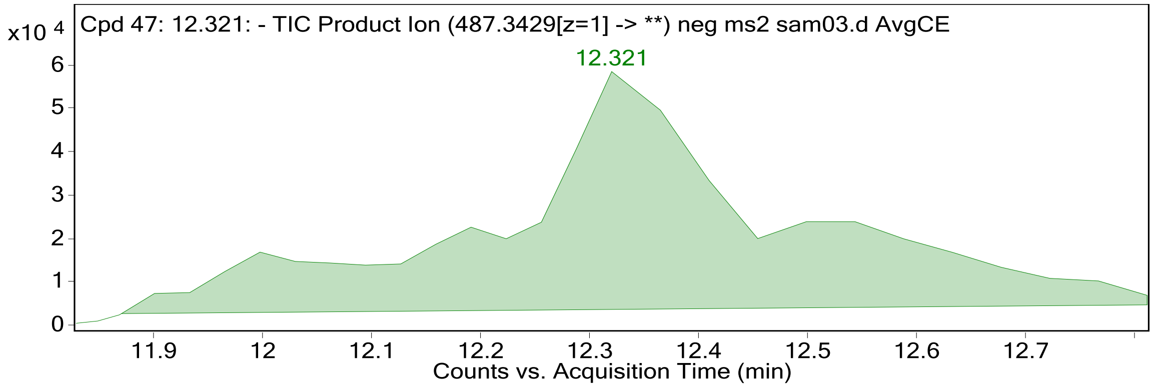

MSMS Spectrum

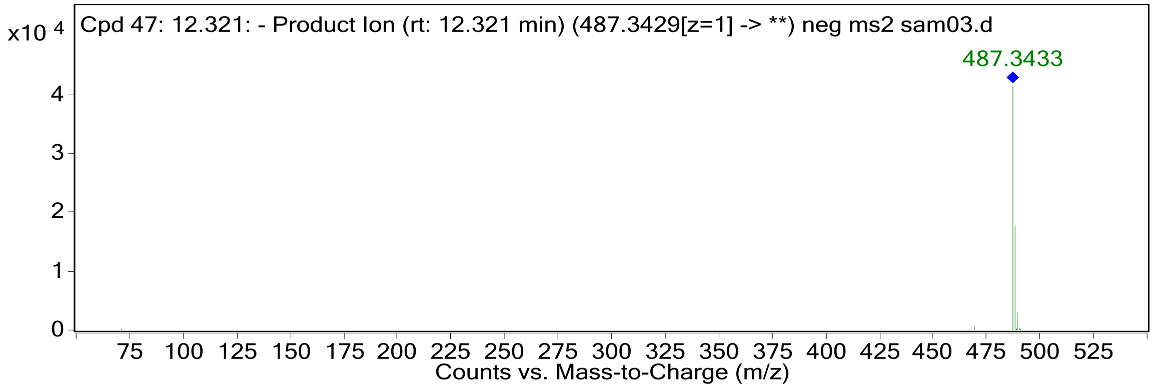

MSMS Spectrum

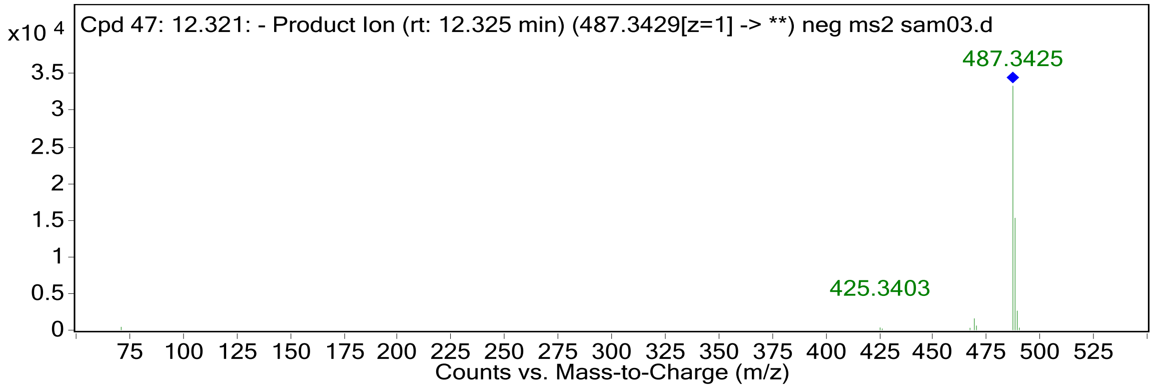

MSMS Spectrum

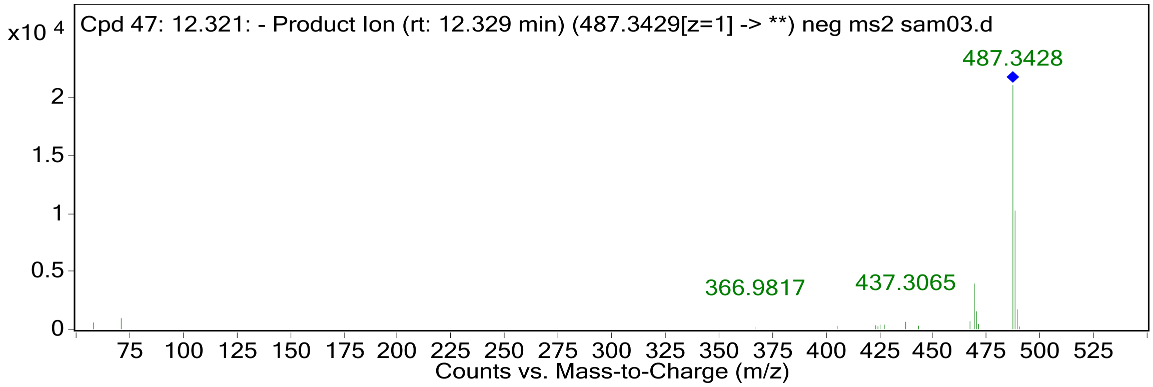

Compound Label	*m/z*	RT	Algorithm
Cpd 48: 12.837	633.3801	12.837	Targeted MS/MS

Qualitative Compound Report

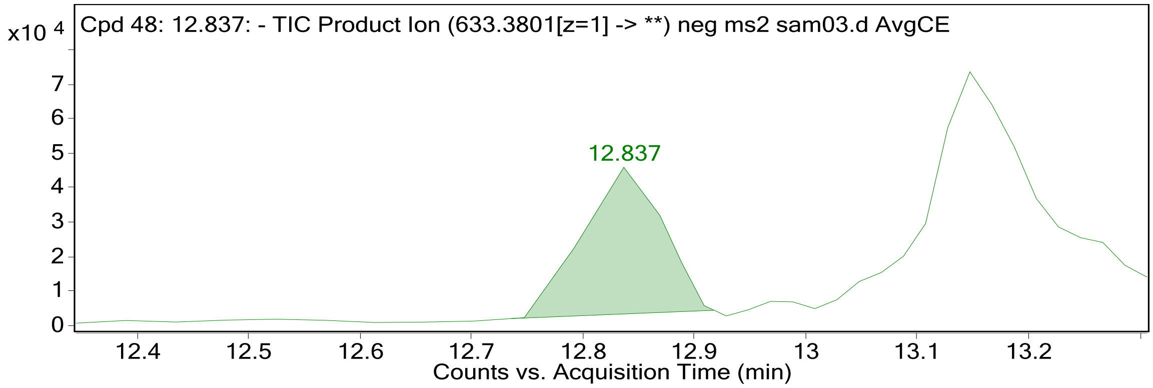

MSMS Spectrum

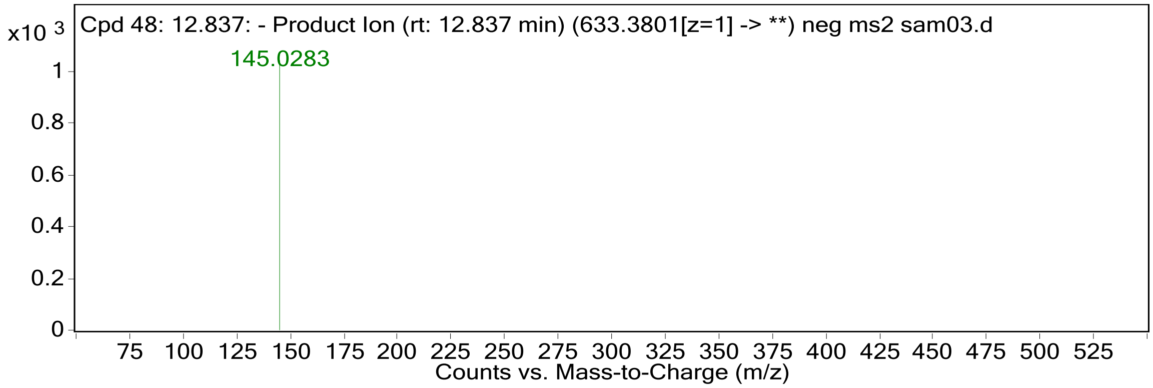

MSMS Spectrum

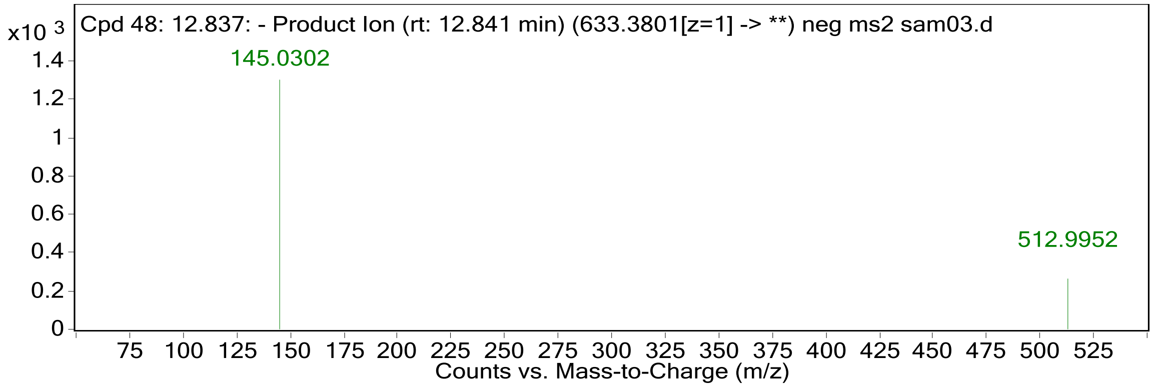

MSMS Spectrum

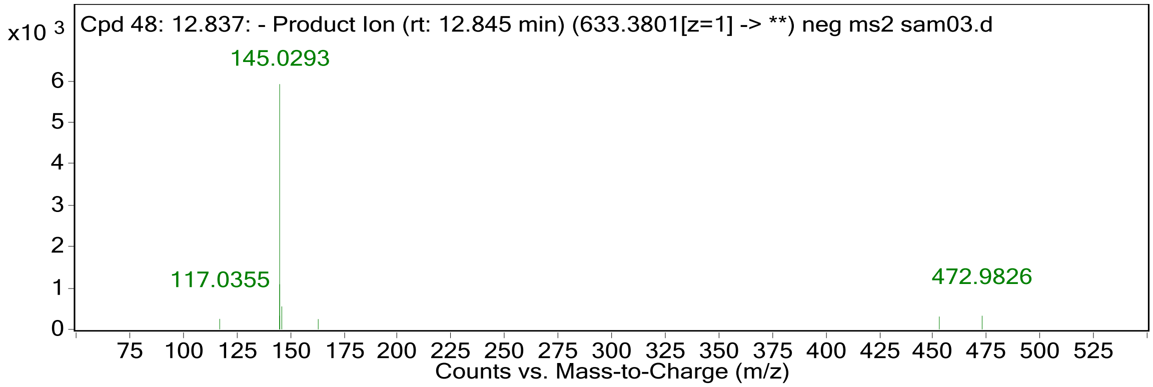

Qualitative Compound Report

Compound Label	*m/z*	RT	Algorithm
Cpd 49: 13.147	633.3801	13.147	Targeted MS/MS

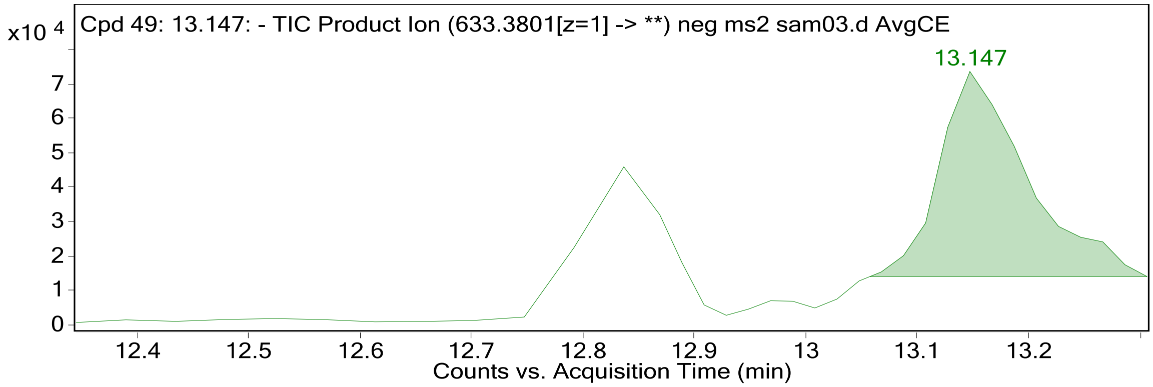

MSMS Spectrum

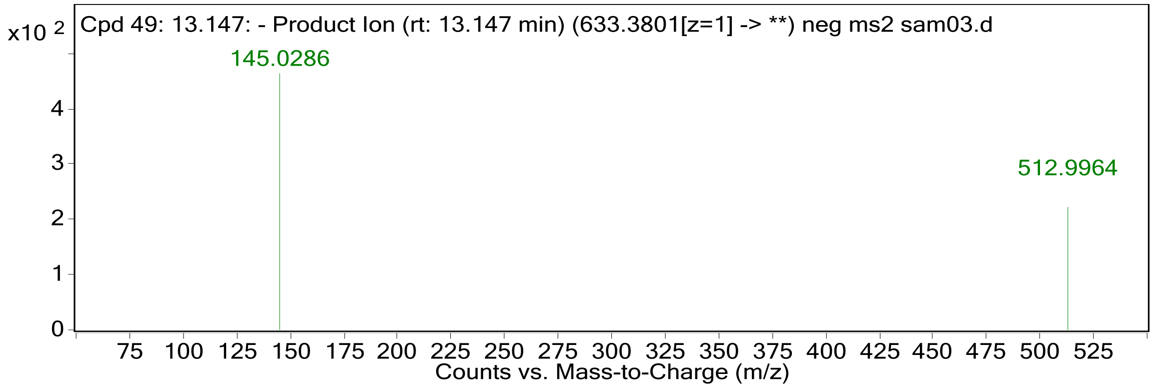

MSMS Spectrum

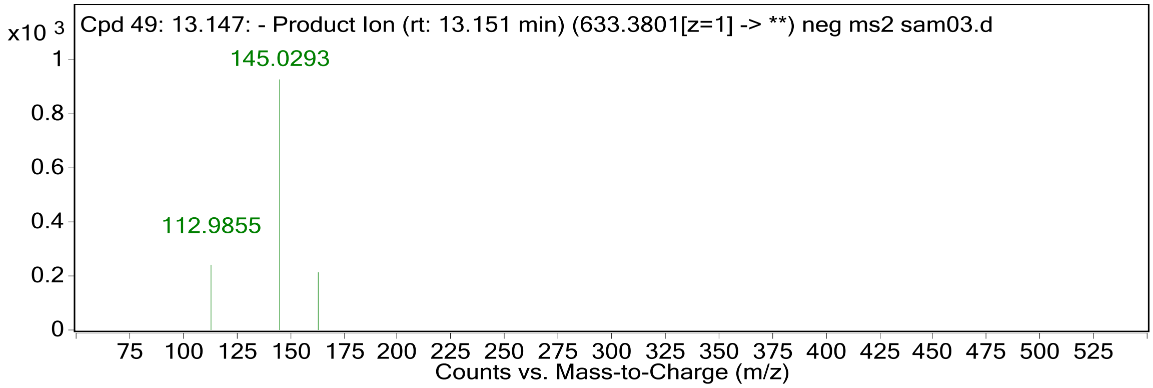

MSMS Spectrum

Qualitative Compound Report

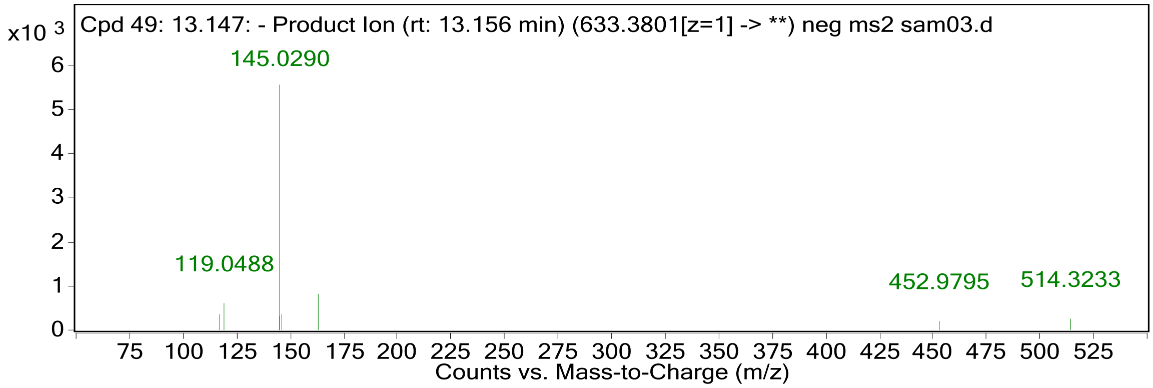

--- End Of Report ---
